# Supplementary material for: The vertebrate Aqp14 water channel is a neuropeptide-regulated polytransporter
Source: Commun Biol. 2019 Dec 11;2:462. doi: 10.1038/s42003-019-0713-y (PMC6906440; doi:10.1038/s42003-019-0713-y)
Supplement: Supplementary file 1 — Suplementary Information [file 42003_2019_713_MOESM1_ESM.pdf]

Supplementary Figure 1. Bayesian majority rule consensus tree of aligned aquaporin codons (exons 2-5/6) inferred from 25 MCMC generations. Posterior probabilities are annotated at each node, with the scale bar indicating the expected substitutions per site. The tree is rooted with lamprey *aqp14*. R4 represents the fourth round of whole genome duplication in salmonids and some cyprinids, with the salmonid *aqp14_1* pseudogenes † indicated in red, and accession numbers given in parentheses.

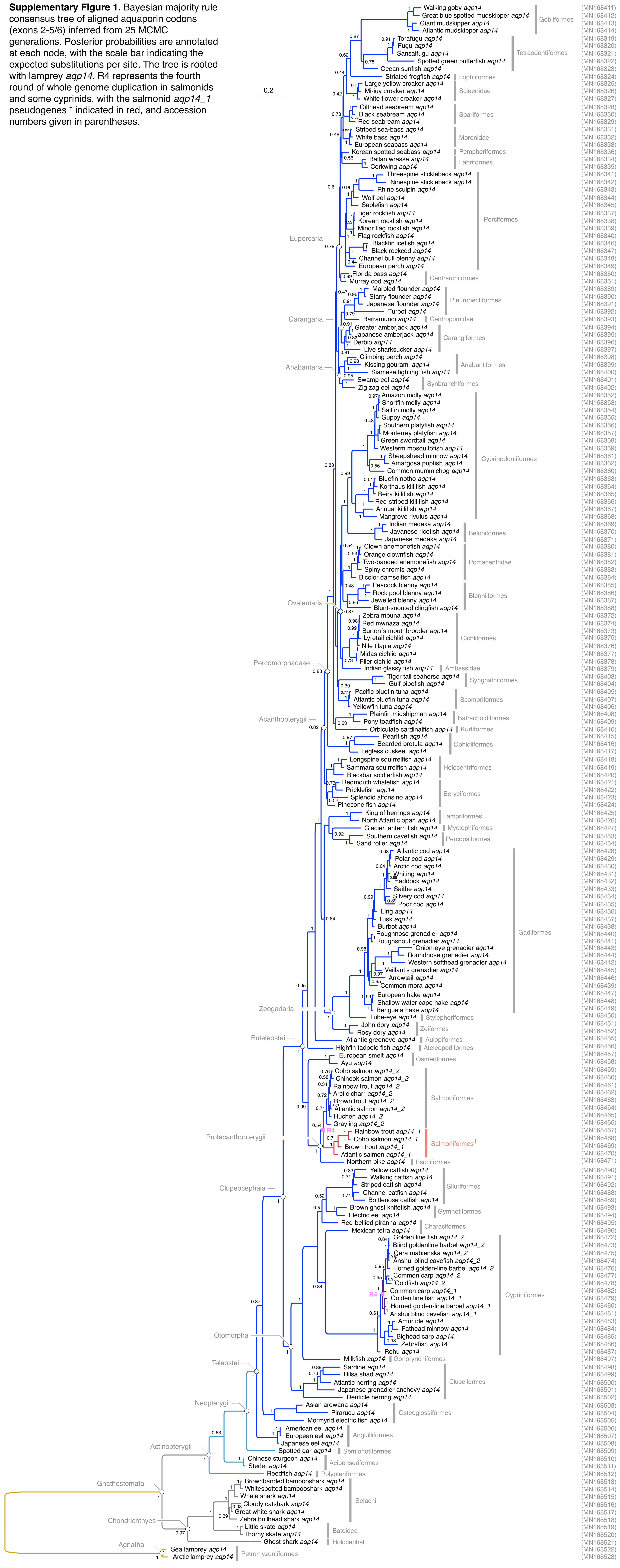

Figure 2a

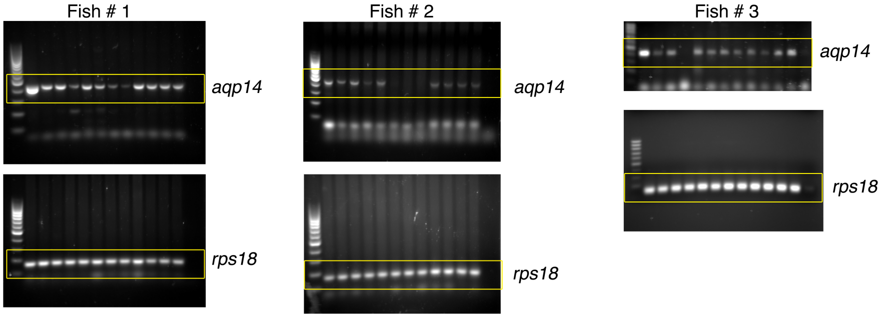

Figure 2b

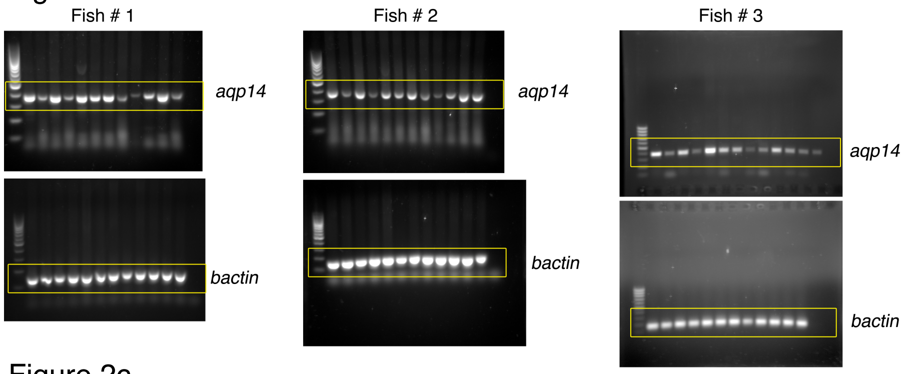

Figure 2c

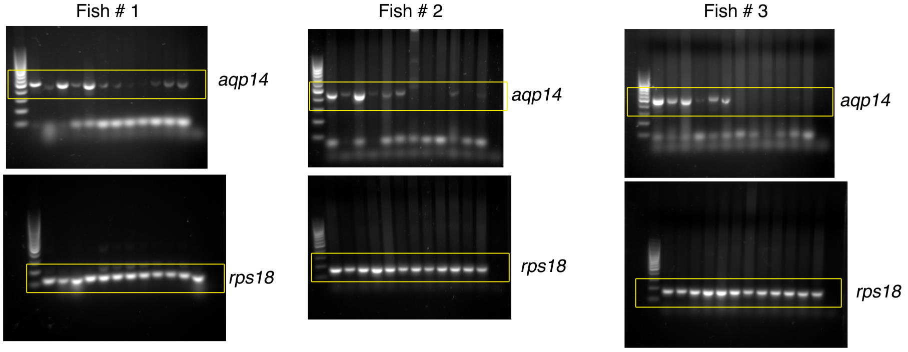

Supplementary Figure 2. Expression of *aqp14* in different teleost tissues. (a-c) Uncropped agarose gels from the RTPCR analysis of *aqp14* gene expression in different tissues from zebrafish (a), Atlantic salmon (b) and gilthead seabream (c) (N = 3 fish). The minus indicates absence of RT during cDNA synthesis. 18s ribosomal protein (*rps18*) was used as reference gene for zebrafish and seabream, while β-actin (*bactin*) was used for the salmon. The size (kb) of PCR products and molecular markers are indicated on the left.

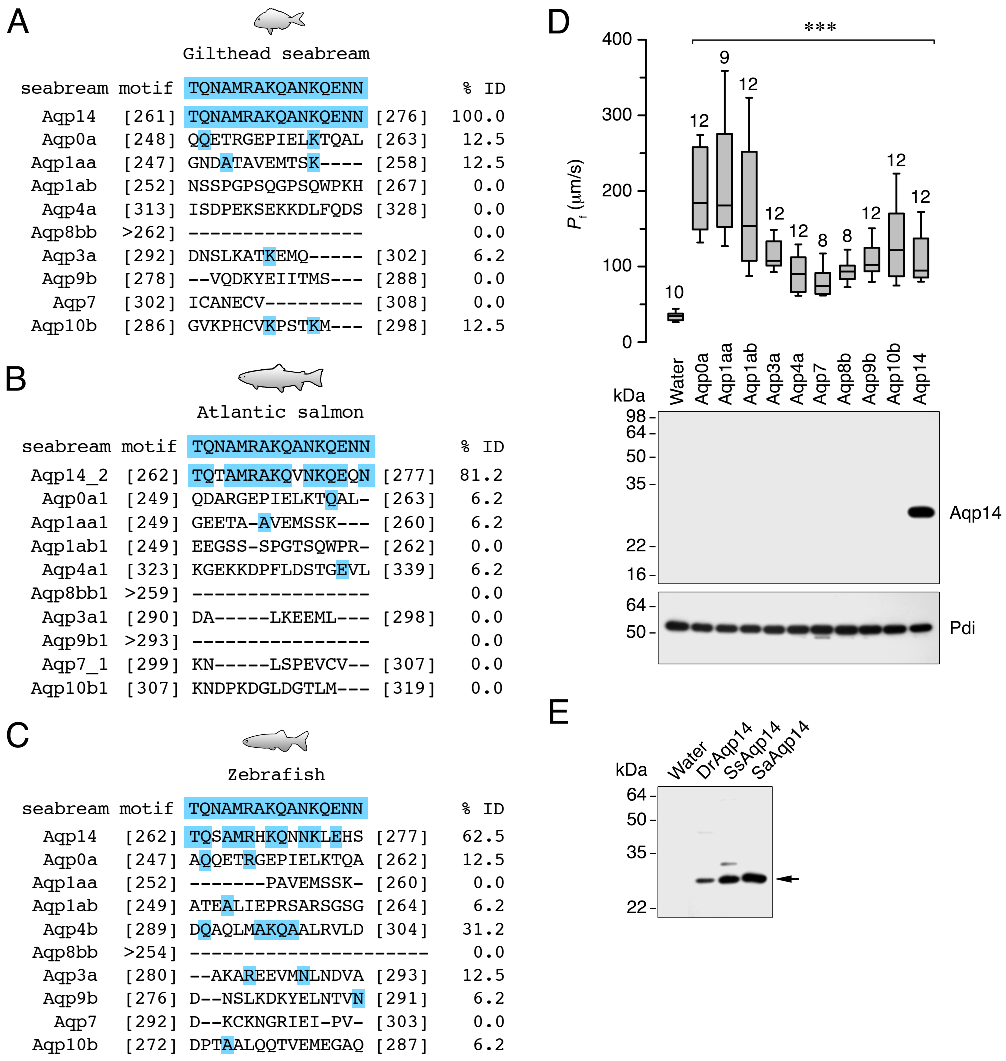

Supplementary Figure 3. Antiserum specificity of seabream Aqp14-Ctab. Alignment of the SaAqp14 C-terminal motif selected for immunization against (A) gilthead seabream, (B) Atlantic salmon and (C) zebrafish aquaporins illustrating the regional positions from the N-terminal methionine and % identity (% ID). Identical residues are highlighted in blue. (D) Osmotic water permeability (P_f, upper panel) and Western blot (lower panel) of *X. laevis* oocytes injected with water (control) or 1 ng of cRNA encoding the seabream channels. Antiprotein disulfide isormerase (Pdi: Sigma-Aldrich, P7496) was used for loading control. All channels displayed highly significantly different P_f ($P < 0.001$, n=8, 9 or 12 biologically independent oocytes, unpaired Student's *t*-test) with respect to control oocytes. Molecular mass markers (kDa) are on the left. (E) Western blot analyses of total membrane protein extracts from *X. laevis* oocytes injected with water or expressing zebrafish, Atlantic salmon or gilthead seabream Aqp14 (DrAqp14, SsAqp14 and SaAqp14, respectively) using an affinity purified antiserum against SaAqp14.

Figure 3d

α-DrAqp14 PDI

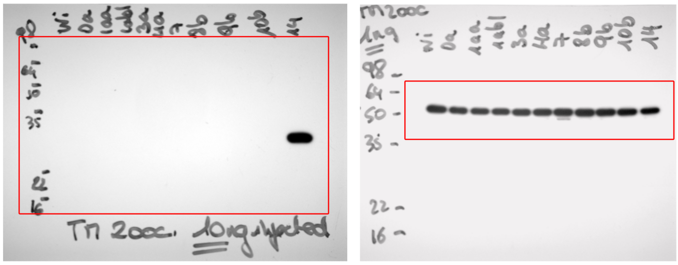

Figure 3e

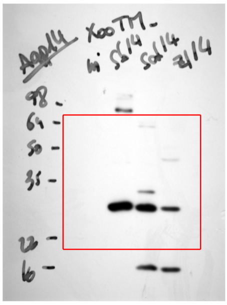

Supplementary Figure 3d, e. Uncropped Western blots shown in Supplementary Figure 3D, E

Figure 4d

DrAqp14 Preadsorbed

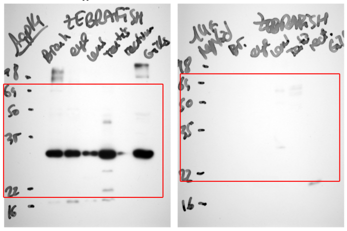

Figure 4e

SsAqp14 Preadsorbed

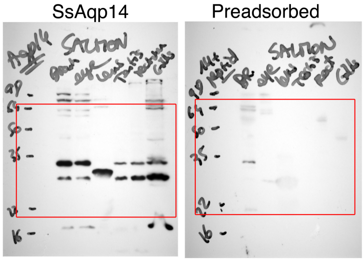

Figure 4f

SaAqp14 Preadsorbed

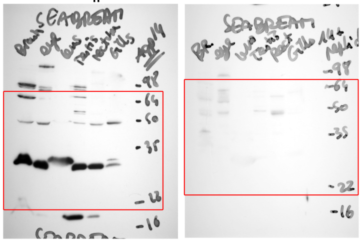

Supplementary Figure 4d, e, f. Uncropped blots shown in Figure 3D, E, F.

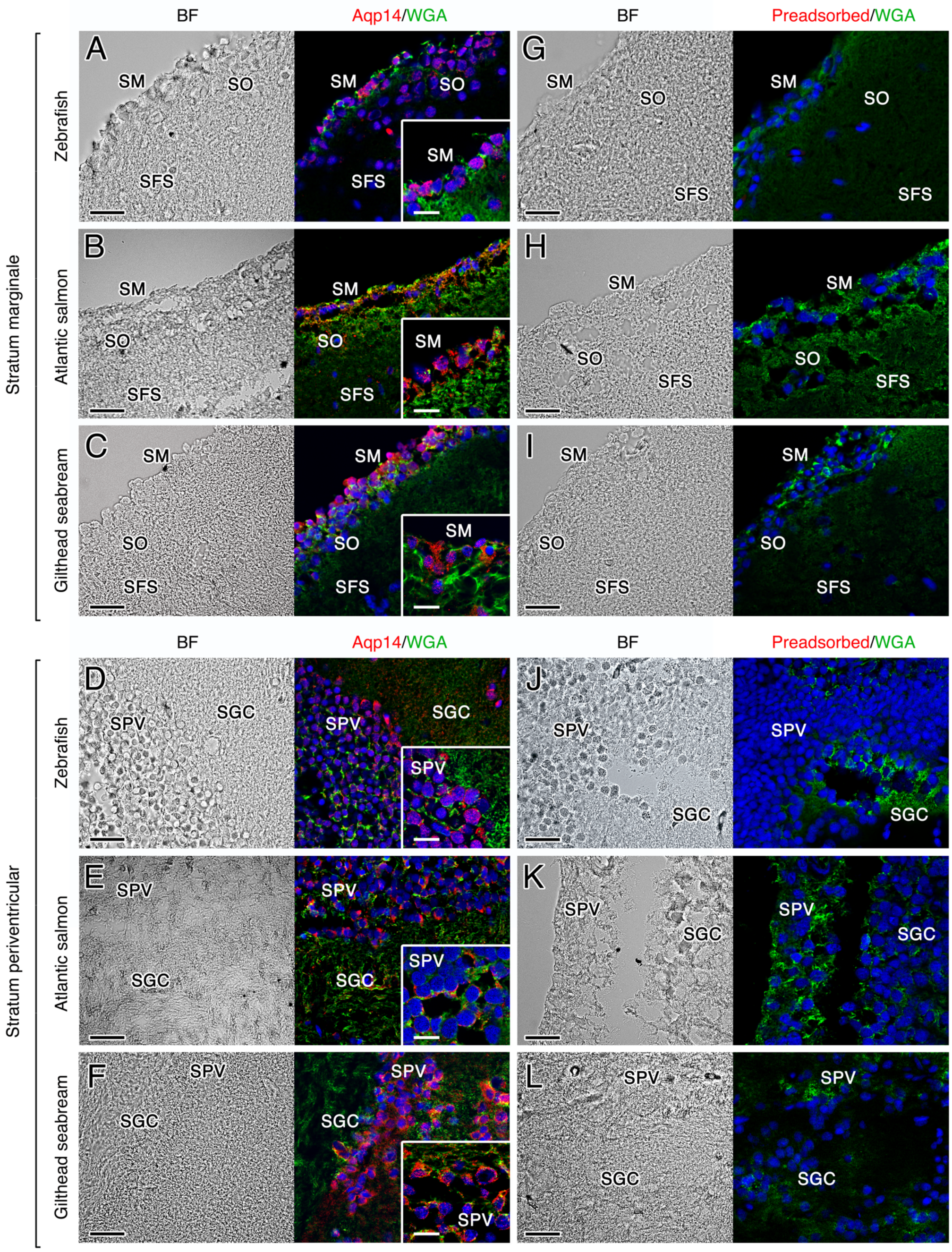

Supplementary Figure 5. Immunolocalization of Aqp14 in the brains of zebrafish, Atlantic salmon and gilthead seabream. (A-F) Representative brightfield (left panels) and immunofluorescence microscopy images (right panels and insets) of Aqp14 in the stratum marginale (A-C) and periventriculare (D-F) of the optic tectum. Sections were labeled with affinity-purified seabream Aqp14 antiserum (red) and counterstained with 4′,6-diamidino-2-phenylindole (DAPI; blue) and wheat germ agglutinin (WGA) (green). (G-L) Control sections incubated with preabsorbed antiserum were negative. Scale bars: 20 µm (10 µm insets). SM, stratum marginale; SO, stratum opticum; SFS, stratum fibrosum superficiale; SPV, stratum periventriculare; SGC, stratum griseum centrale.

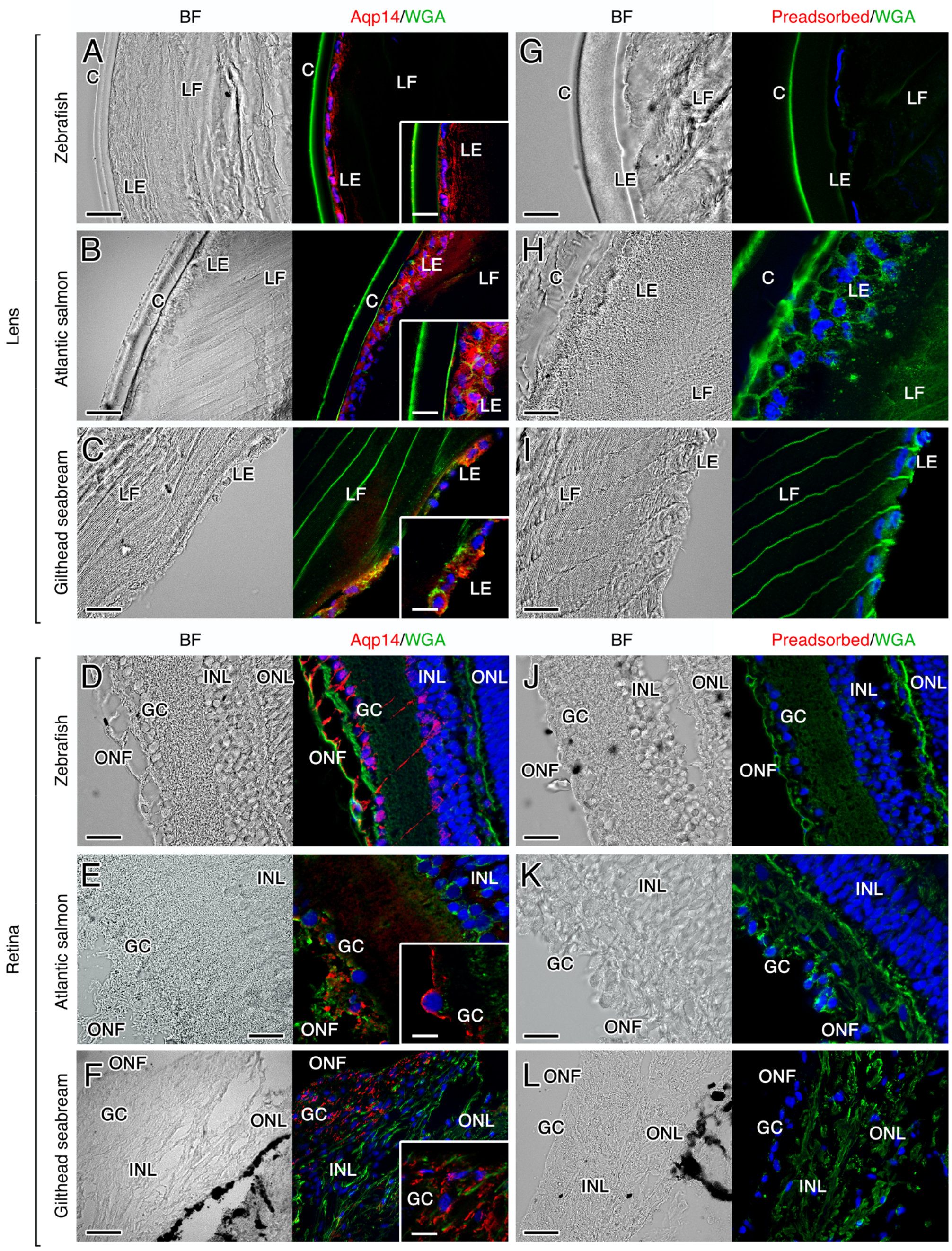

Supplementary Figure 6. Immunolocalization of Aqp14 in zebrafish, Atlantic salmon and gilthead seabream ocular tissues. (A-F) Representative brightfield (left panels) and immunofluorescence microscopy images (right panels and insets) of Aqp14 in the lens (A-C) and retina (D-F). Sections were labeled with affinity-purified seabream Aqp14 antiserum (red) and counterstained with 4′,6-diamidino-2-phenylindole (DAPI; blue) and wheat germ agglutinin (WGA) (green). (G-L) Control sections incubated with preabsorbed antiserum were negative. Scale bars: 20 µm (10 µm insets). C, capsule; LE, lens epithelium; LF, lens fiber; ONF, optic nerve fiber; GC, ganglion cells; INL, inner nuclear layer; ONL, outer nuclear layer.

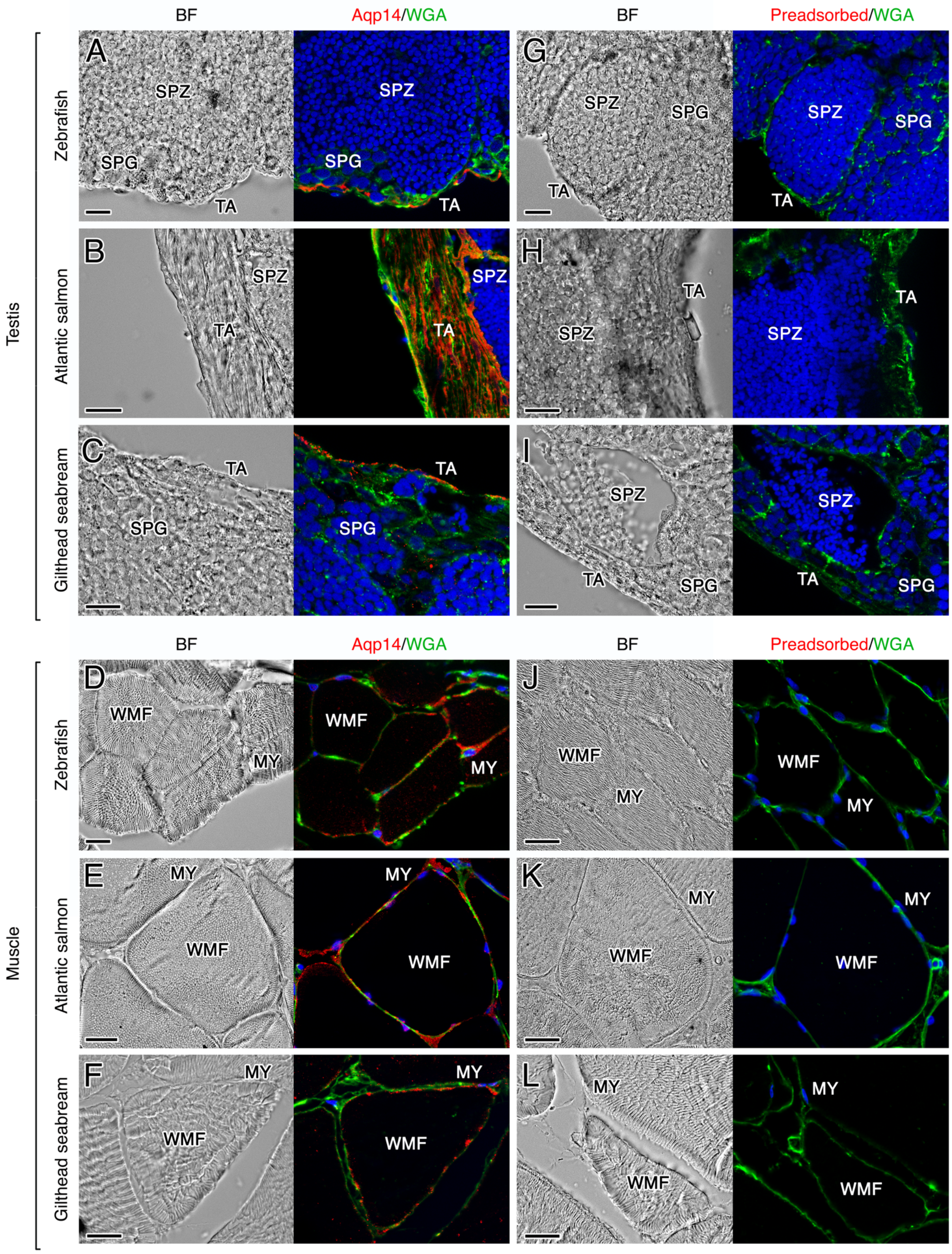

Supplementary Figure 7. Immunolocalization of Aqp14 in zebrafish, Atlantic salmon and gilthead seabream testis and muscle. (A-F) Representative brightfield (left panels) and immunofluorescence microscopy images (right panels and insets) of Aqp14 in the testis (A-C) and muscle (D-F). Sections were labeled with affinity-purified seabream Aqp14 antiserum (red) and counterstained with 4′,6-diamidino-2-phenylindole (DAPI; blue) and wheat germ agglutinin (WGA) (green). (G-L) Control sections incubated with preabsorbed antiserum were negative. Scale bars: 20 µm. TA, tunica albugínia; SPG, spermatogonia; SPZ, spermatozoa; WMF, white muscle fiber; MY, myocyte.

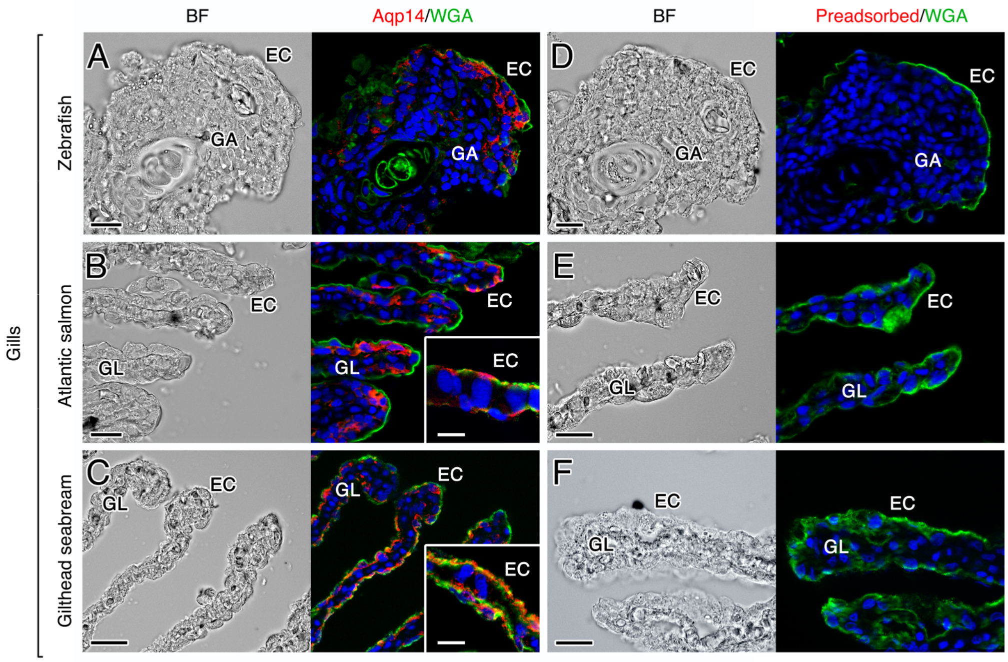

Supplementary Figure 8. Immunolocalization of Aqp14 in zebrafish, Atlantic salmon and gilthead seabream gills. (A-C) Representative brightfield (left panels) and immunofluorescence microscopy images (right panels and insets) of Aqp14. Sections were labeled with affinity-purified seabream Aqp14 antiserum (red) and counterstained with 4′,6-diamidino-2-phenylindole (DAPI; blue) and wheat germ agglutinin (WGA) (green). (D-F) Control sections incubated with preabsorbed antiserum were negative. Scale bars: 20 µm (10 µm insets). GA, gill arch; EC, epithelial cell; GL, gill lamellae.

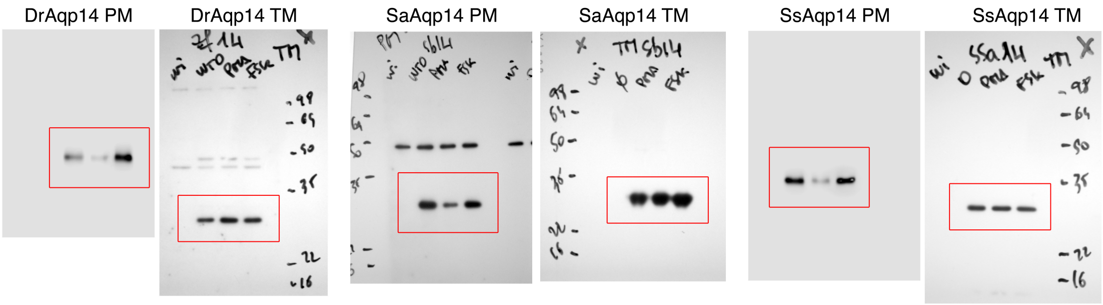

Supplementary Figure 9. Uncropped blots shown in Figure 6C.

Figure 10e

DrAqp14 TM

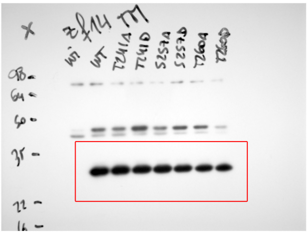

Figure 10g

SaAqp14 TM

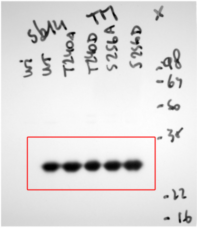

Supplementary Figure 10e, g. Uncropped blots shown in Figure 6E and G.

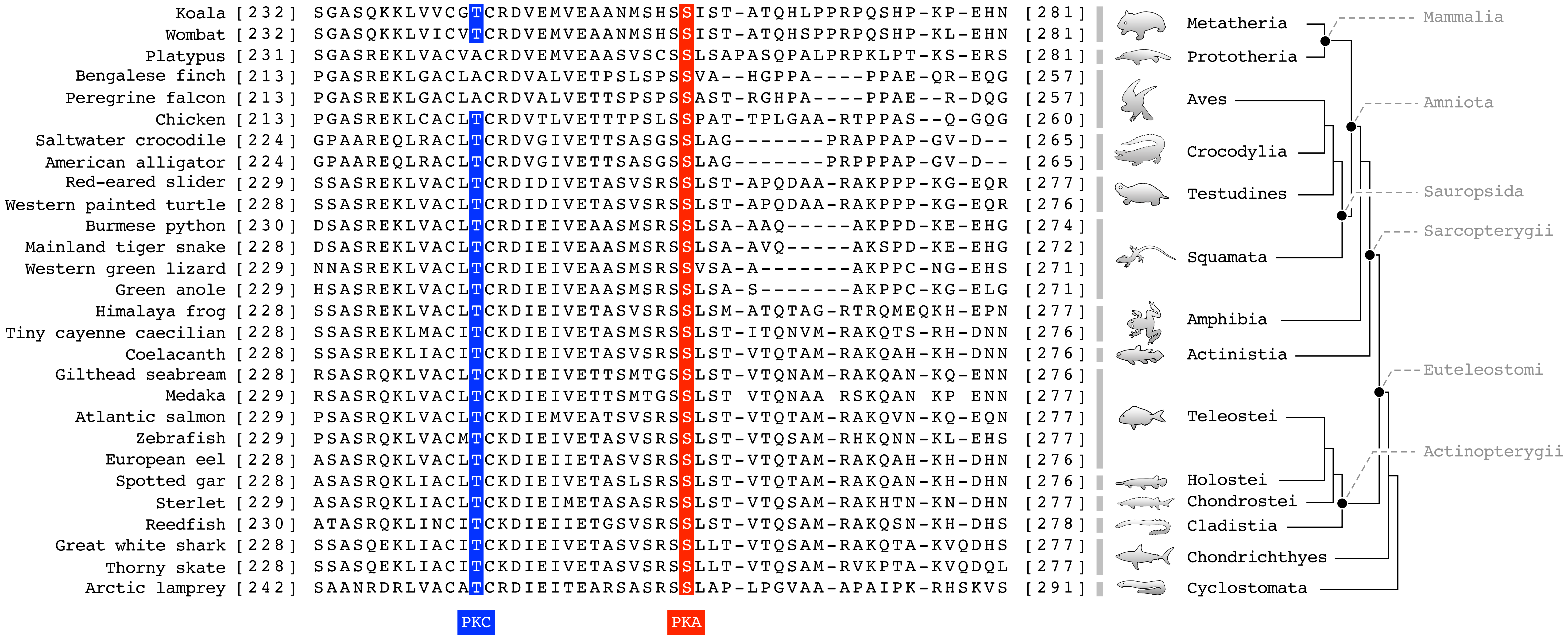

Supplementary Figure 11. Alignment of the C-termini of mammalian, sauropsid, amphibian and piscine Aqp14. Conserved Thr and Ser residues activated through the phosphokinase C (PKC) and phosphokinase A (PKA) signal transduction pathways are highlighted in blue and red, respectively. AQP14 accession numbers: Koala, MN168302; Wombat, MN168303; Platypus, MN168304; Bengalese finch, MN168305; Peregrine falcon, MN168306; Chicken, MN168307; Saltwater crocodile, MN168308; American alligator, MN168309; Red-eared slider, MN168310; Western painted turtle, MN168311; Burmese python, MN168312; Mainland tiger snake, MN168313; Western green lizard, MN168314; Green anole, MN168315; Himalaya frog, MN168316; Tiny cayenne caecilian, MN168317; Coelacanth, MN168318. Other accession numbers are provided in Supplementary Figure 1.

Supplementary Figure 12. Codon alignment used for maximum likelihood and Bayesian analys
platypus and zebrafish aquaporins.

```
>Platypus AQP6
------------------------------------------------------------
---------------ATGCGTCCGGGCCTCCCTTCGGTCGCCTTCTGCCGGGCGCTGGTG
GCCGAGTTCCTGGCCACGGGCTTGTACGTGTTCTTCGGCGTGGGGTCGGCCCTGTCCTGG
---------------------------CCCTCGGCCCTGCCCTCCGTGCTGCAGGTGGCC
ATCACCTTCAACCTGGCCACCGCCGTGGCCGTGCAGGTCACCTGGAAGAGCAGCGGGGCC
CACGTCAACCCGGCCGTGACCCTGGCCTTCCTGGTGGGCGCCCACATCTCGCTGCCCCGC
GCCCTGGCCTACGTGGTGGCCCAGCTGGCCGGGGCCACGGCCGGAGCCGCCGTGCTCTAC
GGGGTCACCCCGGGGGACGTC---------------------------------------
------------CGCGACAGCCTCGGGGTCAACGCCGTCCGGAGCAGCGTG---------
TCGCAGGGCCAGGCCGTGGCCGTGGAGCTGATCCTGACCCTGCAGCTGGTGCTTTGCGTC
CTGGCCTCCACCGACAGC---CGCCAGGCCCCG------GGC---TCCCCCGCAGCCATG
ATTGGCATCTCCGTAGCCCTGGGTCACCTCATCGGGATCTATTTCACCGGCTGCTCCATG
AACCCCGCTCGCTCCTTCGGCCCGGCGGTCATCGTG------------------------
---------------GGCAAATTTGCCGTC---CACTGGGTGTTCTGGGTAGGGCCTCTG
ACAGGGGCCATCCTGGCCTCCCTCCTCTACAACTTCATCCTATTCCCGGACCAG------
---AAGACCCTGGCTGAGCGTCTGGCCATCTTC------AAAGGCACGTGGGAGGCTGAG
---------------GGCCATCCGGAGGCCCACCCGGAGGCGGAGGCAGGGCCC------
------------------------------ACAAGG---AAGGAGAATGGCGATAGCGGC
GGCGTGGAAATCACCAATCTGGGG------------------CAGGTGGCC---------
------
>Platypus AQP5
------------------------------------------------------------
---------------ATGAGGAAGGAGGTGTGTTCCCTGGCCTTCCTCAGAGCCGTCTTC
TCCGAGTTCCTGGCCACCGCCATCTTCGTCTTCTTCGGCCTGGGCTCAGCCCTCAAGTGG
---------------------------CCGTCGGCGCTGCCCAGCATCCTGCAGATCTCG
TTGGCCTTCGGGCTGGCCATCGGCACGCTGGTGCAGGCCCTGGGCCCGGTCAGCGGAGCC
CACGTCAACCCGGCCATCAGCATCGCCCTGCTCCTGGGGAACCAGATCTCGCTGCTGCGG
GCCCTCTTCTACGTGGCCGCCCAGCTGGTGGGGGCCATCGTCGGGGCCGGGATCCTCTAC
GGCGTCACTCCTGTCAATGCC---------------------------------------
------------CGCGGGAACCTCGCCGTCAACTCTCTCAACAACAACACG---------
TCATTGGGCCAGGCCGTCGTGGTAGAGATGATCTTGACATTCCAGCTGGCCCTCTGCATC
TTCTCCTCCACGGACAGC---CGGCGGTCCGCGCCCCTGGGC---TCTCCGGCCCTGTCC
ATCGGCCTCTCCGTCACCCTGGGTCATCTGGTCGGGATCTACTTCACAGGCTGTTCCATG
AATCCCGCCCGCTCCTTGGGGCCGGCCGTCATCATG------------------------
---------------AAGCGCTTCAGCACCGCGCATTGGGTATTCTGGGTGGGTCCCATC
GTTGGGGCGGCCCTGGCTGCCTTGTTCTATTTCTACCTGCTGTTTCCCAACTCG------
---CTGAGCCTGGAGGACCGCGTGGCCATCGTC------AAGGGCTCCTACGAGCCCGAA
---------------GAGGACTGGGAAGAGCAGCGGGAGGACCAGAGGGAG---------
------------------------------GAGCGG---AAAAAGACC------------
---ATGGAGCTGACCTGCCAG---------------------------------------
------
>Platypus AQP2
------------------------------------------------------------
------------------ATGTGGGAGCTCCGGTCGGTCGCCTTCTCCCGCGCGGTCCTG
GCCGAGTTCCTGGCCACCCTCCTCTTCGTCTTCTTCGGGCTGGGTTCGGCCCTGGACTGG
---------------------------CCGTTGGCCCTGCCCAGCGTGCCGCAGATCGCG
```

```
CTGGCCTTCGGGCTGGCCATCGGCACGCTGGTGCAGGCCCTGGGCCACGTGAGCGGCGCC
CACATCAACCCGGCCGTGACCGTGGCCTGCCTGGTGGGCTGCCGCGTCTCCGTCCTGCGA
GCCGCCTGCTACGTGGCCGCCCAGCTGCTGGGGGCCGTGGCCGGAGCCGCCCTGCTCCAC
CAGCTCACCCCGCCGGACATC---------------------------------------
------------CGTGGAAACCTGGCCATCAACAAGCTGAGCAATAACACG---------
ACGTCGGGACAGGCGGTCACAGTGGAGCTGTTCCTGACGCTGCAACTCGTCCTCTGCGTC
TTCGCCTCCACCGACGAG---CAGCGCGGCGACACCTTGGGC---TCCCCCGCCCTCTCC
ATCGGCTTCTCCGTGGTCCTCGGCCACTTGTTGGGGATCCACTACACCGGCTGTTCCATG
AACCCTGCCCGCTCCCTGGCGCCGGCTGTCATCGTG------------------------
---------------GGCAAGTTTGACGAT---CACTGGGTATTCTGGATCGGGCCGCTG
GTGGGGGCCATCCTGGGCTCCCTCATCTACAACTACCTCCTGTTCCCGCACGCC------
---CTGAGCCTGTCCGAGCGCCTGGCCACGCTC------AAGGGGTTG---GAGCCGGAC
---------------GTGGACTGGGAGGAG---------------AGGGAGGCC------
------------------------------CGGAGA---CGCCAGTCG------------
---GTGGAGCTCCACTCGCCCCAG------------------ACCCTGCCTCGGGGGAAC
AAAGTC
>Platypus AQP0
------------------------------------------------------------
------------------ATGTGGGAGCTGCGTTCGGTGTCCTTCTGGAGGGCCATCTCG
GCAGAGTTCCTGGCCAGCCTGGTCTATGTCTTCTTCGGGCTGGGGGCGTCGCTGCACTGG
---------------------------GCCCCGGGGCCGCTGAACGTCCTGCAGGTCTCG
CTGGCCTTCGGGCTGGCGCTGGCCACCCTGGTGCAGACCCTGGGCCACGTCAGCGGCGCC
CACGCCAACCCGGCCGTCACTTTCGCCTTCCTCCTGGGCGCCCAGGTGTCCCTGCTCCGG
GCAGTCTGCTACGTGGCAGCCCAGCTGCTGGGGGCCGTGGCCGGTGCGGCCGTACTCTAC
GGGGTCACGCCCCCCGCCGTC---------------------------------------
------------CGCGGGAACCTGGCCCTCAACATGCTGCACCCCGGGGTA---------
AGCACGAGTCAGGCCACCGTGGTGGAGATCATCGTGACGCTGCAGTTCGTGCTCTGCGTC
TTCGCCAGCTACGACGAG---CGGCGAGATGGGCGCCTGGGC---TCCGTGGCCCTGGCC
GTCGGCTTCTCCCTCACCCTGGGGCATCTCTTCGGGATGTACTGCACCGGTGCTGGCATG
AATCCTGCCCGTTCCTTCGCCCCTGCCATCCTCACC------------------------
---------------AGGAACTTCACCAAC---CACTGGGTATACTGGGTGGGGCCCATC
ATCGGTGGGGCCTTGGCTGTCTTCCTGTACGACTTCCTCCTCTTTCCCCGGCCC------
---AAAGGGGTGTCTGAGAGACTGTCCATCCTC------AGGGGCGAC---CGGCCCAGT
------------------GCCCCTGAGGGG------------CCAGGAGAGGCC------
------------------------------TTGTCC---GGGGACCCG------------
---GTGGAACTCAAGACCCAG---------------------GCCTTG------------
------
>Zebrafish aqp0a
------------------------------------------------------------
------------------ATGTGGGAGTTCCGGTCCATGTCTTTTTGGCGGGCTGTGTTT
GCCGAGTTTTACGGCACCATGTTCTTTGTGTTTTTTGGTCTGGGAGCTGCTCTCCGCTGG
---------------------------ACCACCGGGCCACACAATGTGCTTCAAGTGGCC
TTCTGCTTTGGGCTGGCAGCTGCCACGTTCATCCAATCCATCGGCCACATCAGTGGTGGC
CACATCAACCCGGCGGTCACTTTTGCATACCTGATTGGCTCCCAGATGTCCCTGTTTCGT
GCCTTCTTCTACATCTGTGCTCAGTGCTTGGGTGCATTGGCTGGCGCCGCTGTGCTTTAT
GGGGTCACGCCAACCAATATG---------------------------------------
------------AGAGGAAATCTGGCCTTAAATACGCTTCAGCCTGGCATC---------
AGTATGGGAATGGCCACTACCATAGAAATATTCCTGACCCTGCAGCTTGTGGTTTGTGTG
TTCGCTGTGACCGATGAG---AGGAGAAACGGGCGACTGGGG---TCTGCTGCCCTGTCC
ATTGGCTTTTCTGTGCTAGTGGGACACCTGCTGGGGATGTATTACACTGGAGCCGGAATG
AACCCCGCCAGGTCTTTTGCCCCTGCTGTGCTCTAT------------------------
```

---------------AGGAACTTTATTAAC---CACTGGGTGTATTGGGTGGGCCCTATG
ATCGGCGCTGCCATGGGAGCTCTGCTCTACGACTTCATGCTGTTCCCACGCGTG------
---CGGGGTCTGTCCGAGAGACTGGCTGTGCTC------AAGGGAAAC---AAACCAACA
------------------GAGCCTGAAGCC------------CAGCAGGAG---------
------------------------------ACCCGA---GGAGAGCCA------------
---ATCGAGCTCAAAACTCAA------------------------GCATTA------------
------
>Zebrafish aqp0b

------------------ATGTGGGAGTTTCGCTCTATGATGTTTTGGCGGGCGGTGTTT
GCCGAGTTTTTCGGCACCATGTTCTTCGTGTTCTTCGGGATGGGTGCCGCTCTACGCTGG
---------------------------ACCACCGGGCCGTACCATGTCTTCCACACTGCT
CTCTGCTTTGGTTTTGCTGCTGCCACACTCATTCAATCCATCGGCCACATCAGCGGAGGA
CACATTAATCCGGCTGTCACCTTTGCATACTTAGTCGGCTCTCAGATGTCTGTGTTCAGA
GCTTTCTTCTACATTTGTGCTCAGTGTCTGGGGGCCATGGCAGGAGCCGCGGCGCTCTAC
GGAGTTACACCCAACAACATG---------------------------------------
------------AGGGGCACGATGGCACTTAACACGCTTCAGCCTGGCATG---------
AGTCTGGGAATGGCCACCACAGTGGAGGTGTTCCTCACTATGCAGTTGGTGGTCTGCGTT
TTCGCTGTCACTGATGAA---AGAAGAAATGGACGTCTGGGT---TCTGCTGCTCTGTCC
ATTGGATTCTCTGTTACTATGGGCCACCTGATGGGGATGTACTACACGGGAGCTGGAATG
AACCCAGCCAGGTCTTTCGCCCCTGCTGTAATCACC------------------------
---------------AGGAATTTCATCAAC---CACTGGGTGTACTGGGTGGGTCCGATG
ATTGGTGCTGCTATGGGTGCCATCTTCTATGACTTCTTCCTGTTCCCCCGTATG------
---CGGGGCTTCTCTGAGCGGCTAGCCACGCTG------AAGGGCAGT---CGACCCCCA
------------------GAGGCCGAGAAC------------CAGCAGGAG---------
------------------------------ACCCGC---GGGGAGCCC------------
---ATCGAGCTCAAGACTCAA------------------------ACCCTA------------
------
>Platypus AQP1

---------------ATGGCCAGCGAGTTCAAGAAGAAGCTCTTCTGGCGCGCCGTGGTG
GCCGAGTTCCTGGCCATGACCCTGTTCGTGTTCATCAGCATCGGTTCCGCACTGGGCTTC
CAGTACCCTCTGGCCAGGAACCGGACGAGCGGCGCCGTCCAGGACAACGTGAAGGTGTCC
CTGGCCTTCGGTCTGAGCATCGCCACGATGGCCCAGAGCGTGGGGCACATCAGCGGGGCG
CACCTGAACCCGGCCGTGACCCTGGGCCTGCTGCTCAGCTGCCAGATCAGCGTCCTGCGA
GCCGTCTTCTACGTGGTGGCGCAGTGCATCGGGGCCATCGTGGCCACCGCCATCCTCTCG
GGCATCACC---
---------------TCCGCGCTCCCCGAGAACACCCTGGCGGAGGGGATT---------
AACTCCGGGCAAGGCCTGGGCATCGAGATCATCGGCACGCTGCAACTTGTGCTGTGCGTC
CTGGCCACCACGGACCGG---CGCAGGAACGACGTGGGTGGC---TCCGCCCCGCTGGCC
ATTGGGCTGTCCGTGGCGCTGGGCCACCTCCTCGCGATCGACTACACCGGCTGCGGCATC
AACCCTGCCAGGTCCTTCGGCTCGGCCGTGATCACC------------------------
---------------AACAACTTCACTGAC---CATTGGATCTTCTGGGTCGGCCCCTTC
CTCGGGGGCGCCTTAGCTGCGCTCATCTACGACTTCGTCCTGGCCCCGCGCAGC------
---TGCGACCTCACCGACCGCGTCAAGGTGTGGGCC---AGCGGTCAGATGCAG------
------------------GAGTACGACCTG------------GACGCCGAG---------
------------------------------GAGATG---AGCTCCAGG------------
---GTGGAGATGAAGCCCAAG--

>Zebrafish aqp1aa

------------------ATGAACGAGCTGAAGAGCAAGGCTTTCTGGCGGGCCGTCCTG
GCCGAGCTGCTGGGAATGACCCTGTTCATCTTCCTCAGCATTACAGCAGCTGTGGGA---
------------------AACGCCAACACTCAAAACCCAGACCAGGAGATCAAGGTGGCG
CTGGCTTTCGGGCTGTCCATCGCCACCCTCGCCCAGAGCCTGGGACACATCAGCGGAGCT
CACCTGAACCCTGCCGTGACCCTGGGTCTGCTGGCCAGCTGTCAGATCAGTCTGCTGAGG
GCCGTCATGTATATTCTGGCCCAGATGATCGGGGCGACTGTGGCCAGCGCTATAGTGCTC
GGGGTCTCCAAA--
------------GGGGACGCCCTGGGACTGAATCAAATCCACACAGATATT---------
TCAGCAGGTCAAGGTGTTGGAATTGAGCTCCTGGCCACCTTCCAGCTGGTGTTGTGTGTT
TTAGCAACTACAGACAAA---AGGCGGCGGGACGTGTCGGGC---TCCGCTCCTCTGGCC
ATCGGCCTCAGTGTTTGCCTGGGACATCTGACAGCCATCAGCTTCACGGGATGTGGAATC
AATCCTGCTCGAACATTCGGACCAGCAATGATTCGT------------------------
---------------CTAGATTTCGCCAAC---CACTGGGTCTACTGGGTCGGGCCCATG
TGTGGAGGTGTGGCGGCTGCGCTAATCTATGACTTTCTGCTTTACCCAAAAATG------
---GATGATTTCCCTGAGCGTGTGCGAGTGCTGGTG---TCCGGTCCGGCCACT------
------------------GATTATGAGGTC------------AACGGTACT---------
------------------------------GACGAT---CCCCCTGCA------------
---GTAGAGATGTCCTCAAAG---------------------------------------

>Zebrafish aqp1ab

---------------ATGGCACGAGAGCTTAAAAGCTGGTCGTTCTGGCGGGCCGTGCTG
GCCGAGTTTGTTGGCATGACCATATTTGTTTTCATTGGCATCGCCTCGGCTATTGGA---
------------------AATAAGCATAACAGATATCCCGACCAAGAGGTGAAGGTGGCT
TTAGCTTTCGGTCTGGCCATCGCCACGTTGGCGCAGAGTTTAGGGCACATCAGCGGAGCC
CACCTGAACCCGGCCATCACTTTAGGGCTCTTGGTTAGCTGTCAGATCAGTTTCTTCAGG
GCCTTCATGTATATCATTGCACAGATGTTGGGAGCTGTGCTGGCGAGCGGCATTATGTTC
AAAGTTAGTCCCGACCCC--
------------GATACAACTCTCGGACTCAATATGCTGGGTAACGGTGTG---------
AAAGTTGGTCAGGGATTTGCCATTGAGCTTTTTACCACCTTTCAGCTGGTTCTGTGCGCT
TTGGCCACGACGGATAAA---AACCGAACTGATGTTTCCGGC---TCCGCACCCCTCGCC
ATCGGGCTTTCTGTTGGTTTGGGGCACCTGGTAGCAATCAGCTACACTGGATGTGGCATC
AACCCCGCTCGATCTTTCGGACCAGCCGTTGTCCTC------------------------
---------------GAGTCTTTTAAAAAT---CACTGGATTTACTGGATTGCCCCCATG
TGTGGTGGAGTGGCTGCTGCGCTCATTTACGACTTCTTGCTCTTCCCCAAAAGA------
---GAAGCACTTCGCAAGCGTATGAATGTCCTG------AAAGGCACCGCTGACCCA---
------------------GACCCGTCCGCC------------------------------
---------------------------------------ACAGAAGCACTA---------
---ATCGAACCCCGAAGCGCCAGATCCGGTTCTGGT------CAGTGGCCCCGGCCC---

>Zebrafish aqp15
--ATGAAA
CCATTGTTCAGTTTGATCCTGACAGACATACTGTCTATCTTGTTTTTACGTGACGTGTTT
TGTGAATTTTTAGGAACCGTTTTTTTTCTCTTCATCAGCCTCTCTTCTGCCATTCTGTGG
------------------CCA---CAT---GCTTCCACACCGGACCCACTGCATGTTTCT
TTGGCTTTTGGAGTATCGGTGGCCCGGGCTGGTGTCTGCCTGGGTGAGGTG---------
CATCTCAACCCAGTAATTACCCTGGCATTGGTTGCAGGCTTGAGGGTGAGCCCTTGGAGG
GGAGTCCTGTTGGTTGGAGCCCAGCTGCTTGCTGCTCTCAGTGCCTGTGCTATCCTTTTA
GTCATTGCACCA--
---------------ACTACC---------CAACAGGTGGCCCCGGGGGTG---------
TATCTGTATCAGGCTCTGTTAGTG---ACGGCTGTTACATTCCAGCTAGTGCTGTGTGTC

CAGGCTGCCACTCATCCC------AAATCCGCATTTTCCTCC---AATCCTCCTGCTGTC
ACCGGCCTGTCGGACACCCTCGGTCATCTCATGGCGATCGGCTTCACCGGCTGTGGAATG
AATCCTGCCAGGTCATTTGGTCCAGCTGTGCTAACT------------------------
---------------ATGAACTTCCACAAT---CATTGGGTGTATTGGGTTGGGCCTTGC
TCTGGCTCTCTCCTAACCTGGTTTCTGCATGACCTGCTGCTTCGTCCTTGCTGG------
---AGCTGCTTTGGCGACTGGGTGACCGAATAC------AAAGAGACGTTCCTGAAA---
------------------GACCTTTCCAAG------------AAGCCCAGG---------
------------------------------AATCCT---GAACCAAAT------------
---ATGGAAGCA--

>Platypus AQP4
ATGAGTGACAGACCGGGATCAAGGCACTGGGGGAAGTGCGTGCCTCTCTGCAAGCCCAAG
AGCAACATCATGGTGGCATTCAAAGGGGTGTGGACGCAGCCTTTCTGGAAGGCGGTGGCC
GCGGAGTTTCTGGCCATGGTCATTTTCGTCCTCCTCAGCCTGGGCTCGACGATCAACTGG
------------GGC---GGGAGCGAGCAGCCGTTGCCCACGGACCTGGTCCTCATCTCG
CTCTGCTTCGGGCTGAGCATCGCCACGATGGTCCAGTGTTTCGGGCACATCAGCGGGGCC
CATGTGAACCCGGCGGTGACGGTGGCCATGGTGTGCACGAGGAAGATCAGCATCGCCAAG
TCAGTCTTCTACGTCGCGGCGCAGTGCCTGGGGGCGGTGGTCGGTGCTGGCCTCCTCTAC
CTCGTCACTCCTCCCGGTGTC---------------------------------------
------------CTGGGCGGACTCGGAGTCACTACGGTACATAAAAACCTT---------
ACTGCTGGTCACGGACTCCTAGTGGAGTTGATAATCACATTTCAGCTGGTGTTTACCATC
TTTGCCAGCTGTGATCGC---AACCGAACGGACGTCACTGGC---TCAGTCGCATTAGCC
ATCGGGTTTTCCGTTGCCATCGGGCACTTATTTGCGATCAATTATACGGGCTCCAGCATG
AACCCTGCCCGGTCCTTTGGACCTGCTGTTATCATG------------------------
---------------GGAAACTGGGAGAAC---CACTGGGTATACTGGGTGGGACCGATA
ATAGGAGCCGTCCTGGCTGGTGGTCTCTATGAGTACGTCTTCTGTCCTGATGCT------
------GAGCTCAAACGTCGCCTGAGGGAGGCCTTCAACAAGGCCGCCCAACCCGCCAAA
GGCCACTACGTGGAGGTGGACAACAGCCAGAACCAAGTGGAAACGGATGACCTGATCTTG
AAACCCGGCCTCGTGCACGTCATTGACATCGACCGGGGAGAGGAGAAG---------AAG
GGGAAAGAGACGTCCAGCGAG---------------------GTGCTGTCT---------
TCCGTA
>Zebrafish aqp4b
ATGACAAGCTGTGGAGCCCTGGACACATTCAGGAGATGCGTGTCGTCTTGCTCATGCAAT
AACAGTATCATGGCAGCCTTTAAAGGTGTGTGGACTCAGGAATTCTGGCGGGCGGTTTCC
GGAGAGTTTTTGGCCATGATAATATTCGTGCTGCTCAGTCTTGGATCCACCATCAACTGG
------------GGAGCAAAACAGGAGAACCCTCCACCCGCCGACCTTGTCCTCATCTCC
CTTTGCTTTGGCTTGTCCATCGCAACCCTCGTCCAGTGTTTCGGGCACATCAGCGGCGCC
CACATCAACCCGGCCGTTACTGTGGCAATGGTTGCTACACGGAAGCTGAGTCTGGCGAAG
GGTGTGTTTTATTTGTTGGCACAGTGTTTGGGGGCAGTAGTGGGAGCAGCCATCTTGTAT
GGAGTGACACCGGCGTCAGTG---------------------------------------
------------AGAGGAGGAATGGGAGTGACGTCTGTTAATGAAGAGATC---------
TCCGCTGGTCATGCTATTGTGATAGAGCTCATAATCACTTTTGAGCTTGTTTTCACTGTC
TTTGCTACCTGTGACCCC---AAGCGTAATGATCTCAAAGGT---TCGGCAGCACTGGCT
ATTGGTCTGTCTGTGTGCATCGGCCATCTCTTTGCGATCCCGTACACTGGGGCCAGTATG
AATCCAGCTCGCTCTTTTGGTCCCGCAGTCATCATG------------------------
---------------GTAAAATGGCAGGAC---CATTGGGTGTACTGGGTGGGCCCTTTA
ATAGGAGGAATCCTTGCTGCAGCTGTATATGAATACCTCTTCTGTCCTGACCCT------
------GACCTGAAGCGCCGCTATGCTGACGTCCTCTCCAAGAGCCCCTTCCAGATGGAG
CCATATCGAGTG---GTGGACACAGACTCGTACCCCAGCGATCAGGCTCAGCTCATGGCC
AAACAGGCGGCACTCAGAGTGCTGGATTTGGAGAAG---AAGGAGAGA------------
------GAGTCCACCGGAGAG---------------------GTCCTGTCA---------

TCCGTA
>Platypus AQP14

---------ATGGCTACCCGGGAGGGCCTACGAAGCTGCCGACTCTGGCGGGCGGTGCTG
GCCGAGGTGGTGGGCACCTTTGTGTTTGTTGGGGTGGTTCTGGGGGCCTCCTGCCCCATA
------------GGGGGGCCT------CCAGCTCTGCCCCCGCCCGCCCTTCAGCCAGCC
CTGGCCGCAGGACTGGCGGGGATGGGACTGGCGCAGAGCTTTGGGGAAGTAAGTGGAGCC
CAGGTCAATCCCGCCCTGACCCTGGCCTTGGCCTTGACCCGCCGGCTGGACCCCCTGAGT
GCCGCCACCTACGTCCTGGCCCAGAGTCTGGGCGCTGTCCTGGCCTCTGCTGCCTTCTAC
CTGGCACTGCCCCGCTCTGCC---------------------------------------
------------ACTGCCCAGCTGCTCCTGCTTCAGGTGAGCGGTGAGGCC---------
CAGGCCGGGCAGGCCCTGGGCATGGAGATCTTCTCCACCTTCCAGCTGGCTCTCACCATC
TTCGCCACAGCTGACCAG---CGGCGGCGGGAGACAGGCCAA---CTGGGCAGCCTGGCC
GTGGGCTTCTCGGTGACCGCCGGGGCCCTGGCGGCGGTGCCATTCTCTGGAGGCAGCATG
AACCCTGCCCGCTCTCTGGGGCCTGCTGTGCTGACT------------------------
---------------GGCATCTGGGACTGC---CACTGGGTGTACTGGCTGGGCCCGGCC
TTAGGGGCCACCATTGGCGGCCTCTGCTATGAACTCCTCTTCTCCTCCGGGGCC------
------TCGAGGGAGAAGCTGGTGGCCTGTGTCGCCTGCCGAGACGTGGAGATGGTGGAA
GCCGCCAGCGTG---TCCTGCTCCTCCCTCTCCGCCCCTGCCTCACAGCCCGCCCTGCCC
CGCCCC------------------------AAGCTC---CCCACCAAG------------
---TCTGAACGCAGC---

>Zebrafish aqp14

---------ATGGCAATAAAAGAGGAGCTGCGGAGCCGTCAGTTTTGGCAGGGGATCCTG
GCAGAGGTTCTTGGTTCTCTGGTGTTTGTATCTGCTGTTTTGGGCTCTTTAGTGCCGGGG
------------------CCG------GATGGGGTGTCCCCGGGACCCATCTACCCTGCA
CTGGCTGCTGGTATGGCAACTGTGGTTCTGGGATATTGCTTTGGTGAAATCAGCGGGGCT
CAGGTGAATCCCGCAGTGACGGTGGCTCTCTTGGCCACCCGTAAGGTGGATGTGTTGAGG
GCTGTGGTGTATCTGGTGGCTCAGTGTTTGGGTGGGATCCTCGCAACTGGCCTCATGTAC
CTCTCACTGCCTCTGAAGTCA---------------------------------------
------------ACCGCACAGAACTACATCAACAAGGTCCCAGTGGAGATG---------
AACGCAGGTCAAGCTCTTGGAATGGAGATGCTTGCTACGTTTCTTCTGGGTTTCACTGTG
TTTTCTGTGGAAGATCAA---CGCAGGAGAGAAATAAATGAA---CCTGGAAACTTAGCC
ATTGGCCTTGCTGTAACCACTGCGATCTTTATTGCTGGGAGATTTTCTGGTGCCAGCTTG
AATCCCGCTCGCTCCCTTGGTCCTGCTATAATACTG------------------------
---------------GGATATTGGGAACAT---CACTGGGTGTACTGGATCGGGCCAATA
TTAGGTGCAGTCCTAGCTGGAGTCTCTCATGAGTTTATTTTCGCACCGAGCGCA------
------TCCAGACAGAAGCTCGTGGCCTGCATGACCTGTAAGGACATTGAGATAGTGGAA
ACGGCCAGTGTG---TCTCGATCGTCACTGTCCACAGTTACACAGAGCGCC------ATG
AGACAC------------------------AAACAA---AACAACAAG------------
---CTGGAGCACAGC---

>Platypus AQP8
ATGTGCGACGTGGAATTGGGAGGGATGAAGAAGGAG---------------CCCGTCAGC
GAAGGCCAGTCGGGGCCCGCCGAGCCTCACTGGTTTGAGCGGAACGTGCAGCCCTGCCTG
GCGGAGGCGGTCGGTTCCGCCCTCTTCGTCTTCATCGGGTGCCTGTCCGTCATCGAG---
----------------------------AACGCGGACGGGACGGGCAGGCTGCAGCCGGCC
CTGGCGCACGGACTGTCCCTTGGCCTCATCATCGCCATTTTGGGAAACATCAGTGGTGGC
CACTTCAACCCAGCCGTTTCCTTGGCAGCCATGCTGATTGGAGGATTGAATTTCATGATG
CTCTTCCCTTACTGGATCTCACAGCTTTGCGGAGGGCTGATAGGAGCTGCTTTGGCAAAG

GTGGTGAGCACAGAGGACCGATTT------------------------------------
------CTGAACGCCTCGGGAGCAGCTTTCGGGACGATCAGCGCGGACGAA---------
CAGGTGGGAGGGGCGCTGCTGGCAGAGACCATCATGACGCTCTTTCTGATCCTGGCCGTC
TGCATGGGAGCCATC------AACGAGAAAACCAAGAGCCCG---CTGGCCCCGTTCTGT
ATTGGATTCACTGTCGGGGCCGACATTTTGGCCGGGGGGGCTATATCGGGAGCCTGTATG
AATCCCGCCAGAGCCTTTGGACCCGCGGTGATGGCC------------------------
---------------AACTACTGGGACTAC---CACTGGGTCTACTGGGTCGGCCCCTTG
ATGGCCAGCCTGATTGCGGCAGTGCTAATAAGGTTCTTCATCGGGGACAGAAAA------

---ATTCGCCTGTTCTTGAAA---------------------------------------

>Zebrafish aqp8aa
ATGACCCCGGCAGAGTCCAAGTCTGAGCTCTTCACTGTGGCCACGGGCGATGGGGGAGAC
AACCACCAGAACCAGCCGAAGAAGCTGCCCTTCTTCGAGCACTACATCCAGCCGTGTCTG
GCAGAGGTCGTCGGCTCTTTCCTCTTCATGTTTGTGGGCTGCGTGTCCGTCATGGGC---
---------------------------AACGTGGGCATCAGCGGGAGCATCCAGCCCGCC
CTGGCACACGGACTAGCACTGGCCATAGCAATAGCAATTTTTGGGGAAATCAGTGGTGGT
CACTTCAATCCAGCCGTGTCTGTGTGTGTTTACCTCATTGGAGGAATGGAGGTGATCCTC
CTGGTGCCATACATCATCTCACAGATGTTAGGTGGAGTGATTGCTGCCAGCCTTGCCAAA
GCTGTGACTACAAATGATGCATTC------------------------------------
------AGCAATGCAACAGGAGCAGCGTTTAATGCCATTCCGTCGTCTGAC---------
GGCATTGGAGCTGCCACTATGGCTGAGATGATCATGACGCTTTTCTTGACTATTGTCGTC
AGCATGGGCGCTGTG------AACGGAAGAACCAAAAGTCAA---CTTGCTCCTTTCTGC
ATTGGACTCACTGTGACGGCAAATATCTTGGCTGGGGGAGGAATATCAGGAGCATGTATG
AATCCTGCACGAGCCTTTGGGCCAGCAGTAGTGTCC------------------------
---------------GGCCACTGGACGCAC---CACTGGATTTATTGGGTTGGACCTCTT
ACCGGCGCTCTGGTCACTGTCAGCATTGTGAGATTGGTAATGGGTGACAAGAAG------

---GTCCGTGTTATTTTCAAA---------------------------------------

>Zebrafish aqp8ab
ATG---CCG------GTGGAAGCAGAGAAGCTGGAGCTCGAAGAACTGGACAAAACCTTG
TTAAACAGGGACAAACCAAAACCTCAGAGCAAATACGAAAGAATATTCCAGCCATGCATT
GCTGAGCTGGTGGGCACAACATTTTTTGTCTTCATTGGCTGTGTGTCTGTTATTGAA---
---------------------------AATGTGGAAGCAGCTGGAAGACTGCAGCCAGCA
CTTGTGCACGGGCTGGCAGTGGCTGTGCTGGTGGCATGTATGGCGGAAATCAGCGGATCC
CATTTCAACCCTCCGTTCACCATCGCTATCTGGTTATGTGGAGGAATGCAGCTGACGATG
GTTGTCCCGTATCTTATCAGTCAGCTTATCGGAGGTGTACTAGGAGCTGCAATGTCAAAG
GTTATGACTTCAGATGAAAACTAC------------------------------------
------GCGAACGCTACTGGAGCTGCATTTGCTGTTCTGAAATCAGATGAG---------
CAGCTGGGGAAGGTGGTGTTTGCTGAAATGGCCATGACGTGTCTGGTGACTCTGGTGGTC
CTGATGGGAGCTGTT------AACGGAAAAAGCAAAAGCCCA---ATGGTGCCCTTCATG
GTGGGCTGCACCGTTATTGTCAATATTCTAGCAGGAGGAGATGTTTCTGGCACCTGTTTG
AACCCTGCGAGAGCTTTTGGACCTGCACTCATGGCA------------------------
---------------AACCACTGGACTTAT---CACTGGGTTTATTGGGTCGGTCCGTTG
GGAGGTGGTCTGGTGGCTGCCGCTCTTATGAGGCTTCTGCTTGGAGATGAAAAG------

---CTACGGGTTGTAATGAAA---------------------------------------

>Zebrafish aqp8b
ATGGCAGAT---------------GACAAAATGGAGAAAGCGGCGATGGATAAAATGGTG
---CAGGAGACGGAGATGGAGGAGCCCGGGCTGTTTGAGCAGCTCGTCCAGCCGTGTATG
GCGGAGCTGGTGGGCACCGCTTTCTTTGTGCTGATGGGCTGTTTGTGTGTGATCGAG---
---------------------------AGCGCGCAGGAGGGGCACACACTGCAGGCCGCG
CTCGTGCACGGGCTCGCGTTGGCGGTGGTCATTGGATGTATGGTGGAGATCAGTGGTTCA
CATTTCAATCCATCATTCACCATCGCCGTCTTCCTCAGTGGTGGCCTGGAGCTGAAAATG
GTGTTGCCGTACCTGATCTCTCAGGTCTCTGGAGGTCTGCTTGGTGCAGTCATGGCCAAG
GGAATGACATCTAGCGAGAAATAT------------------------------------
------GCACAAGCTCAAGGTGCTGCATTTACAGTCCTCCAGGCTGATGAT---------
CACATTATGAAGGCGTTGTTTGCAGAAGCTGCTATGACATGTCTGGCTACCTTGGCGGTC
TTATTGTCAGCGGTC------AATGGCAAGAGCAAAAACCAC---ATGTTTCCATTTCTG
GTGGGCTGCACAGTCATGGTTAATGTGCTGGCAGGGGCCAATGTGTCTGGTGCTTGTCTG
AATCCTGTAAGAGCTCTGGGTCCTGCCGTGCTGACC------------------------
---------------AACTACTGGACTCAC---CACTGGATCTACTGGGTGGGTCCCATC
ACCGGTGGACTCATTGCTGCTGCATTAGTCAGGCTTTTCCTAGGTGACAACGAT------

---ACCCGTGTAGTGATGAAG---------------------------------------

>Platypus AQP11
ATGGCCGGC------------------------------------ACCGTGGTCCTGGTG
------GGCGTCGTGCGGGGCCTGTGGGAGCCCGAGGCCCGTCGCCTCAGGCCCTTGGTC
CTGGAGCTGCTGGCCACC------TTCCAGCTGTGCGCCTGCACCCACGAGCTCGTCGTC
------------------CAGGCCCGCCTCGACCCC---------------TCGCCCCAC
CTGGGCCTCACGCTCTCCTACCTCTCGTCGCTGGTGCACGGGCTGACCCTGCCGGGCGCC
CTGGCCAACCCCTGCGCCGTGCTGCACAAGGTGGCGGCCGAAGGACTGGCCGTAAGGACG
GCCCTGCTCAAGGTGGCGGCCCAGGTGGCGGGGGCCCTGCTGGCCCGGGCCTACGCGCTC
TCCGTGCGGAGCCTGGGCCTGGCGGAG---------------------------------
---CGGCACCGGCCTGACCGG------CGGCCCGCCTGCCGGCCGCCCCTG---CCCGCC
GACGTGCCCCGGGCCTGCCTGGTGGAGGCCATCTGCTCCTTCATCTACCACAGCGCCGTC
GTCCAGTTCAGGGAC---------------GTGCGGCCCAAG---CTGCGCGTCCACCTG
TTGGCCGCCCTCGTCACCTTCCTGGTCTTCGCAGGTGGAAGTATAACGGGGGCCGTGTTT
AATCCAGCCCTGGCTCTCTCGCTGCACATGAAGTGCTTC---------------------
------------CAAGGCGAATTCCACAACTTCGCCATCGTGTACTGGGTAGGCCCCACG
GCAGGTATAATAATAATAATGTTGGTATTTGTT---------------------------
------------------AAGCGCTTACTATGG---------------------------
---------------GCCGAGCACTGCTCTAAGCGC------------------------

>Zebrafish aqp11b
ATGGCTGAT------CTCACCGTCTCTCTATCCATCCTGGTGGGGATTGTAGTTTTAAGC
GAGTTCGCGAGAAGGACGGCTTTATATCTATTCCCGAATCGAGAATGGATAATCTACATG
TTGGAGTTTATTTCTACC------TTTCAGCTGTGCGCGTGCACGCATGAGCTGAAGCTG
------------------CTGGCGGAGCTGGGC------------GGACTGGACCCGCAG

ACCGGACTGACCCTCACTTTCATCATCTCGGTGGTTCATGGGTTCTCATTCCGCGGCGCG
ATCTGCAACCCTACCGGCGCGCTGGAGCTGCTGAGCCGCGGGACTCTGCCGTGGGGATGC
GCGTTGGCCAGGGTGTCCTGCCAGCTGACCGCGGCGGTGGTGTCTCGGTGGGTGATGCCC
GTGGCCTGGGCTCTGGCTCTCTCTGAC---------------------------------
---CTGCACCAAAGACACTCTCTGACGGGTTTTAGATGCTCAAGCAGCCCCGTGAATGCC
CCGGTGCTGCAGGCCGCCGCGGTAGAGCTGAGCTGCGCATTTGTGATGCACACCGCGGTC
AATAACGCGGAGAAG---------------CTGGAGGAGAAA---TACCGCGTCCCGGCG
GTGGCGGCGCTCATCACCACTCTAGTCTATGCGGGTGGACGTCTCACAGGCGCTGTGTTC
AACCCAGCACTGGCATTCTCTATACAGTTCCCCTGTCCT---------------------
------------GGAAACACGTTCACAGAGTACAGCTTCGTGTACTGGATGGGACCAATA
CTAGGTATGTCAGCCTCTCTGCTGCTTTCTGATAAACTGATC------------------
---CCGGCCATTTCTGGAAAAAGCACAATTCCA---------------------------
---------------CAGCAGCTGAATTCTAATGGACTCAAGAAGAAAAAGATGAAG---

>Platypus AQP12
ATGGCCGGC------TTGAACGTGTCCCTGTCCTTCTTTTTCATCGTGGTGGCCGTCTGC
GAGGCGGCCAGGAGGCTCTCCAAGAGGTCCCTCCCGGCCCCGGTGTACGGCAACCTGGCC
AGAGAACTGATCGGCGCC------TTCCAGCTGGGGGCCTGCTGCCTGGAGCTCCGCATG
------------------CTGGTGGTCATCGGCCCGTGGGGGGGCGGCTACGGCCCCGAC
GTCATCCTCACCCTCCTCTTTCTCCTCTTCCTGGTCCACGGCCTGACTTGGGACGGAGCC
TCGGCCAACCCGATCACCTCCCTCCGGGAGTTCCTGACGGCGGAGGCTTCGGCACCGGTG
GCGGCGGTCCGGGCCGCGGCTCACTTGGCGGGGACGTACGCCGGCGACGCCCTGACCCGG
CTCTACTGGTCCTGGGAGCTGACGGAC---------------------------------
---TTCCACCTGATCCAGAACCTGATGGGCCGGGAGTGCAGCTCCGCCCTG---CAGACG
GCCGTGGCCTGGGGGGTCCTGGTGGAAGCCACCTGTGCCTTCTTCTACCACCTGACCCTC
CTCTGCTTCCAACAC---------------AGCCGTCCCCTC---TACAGGGCCCCGGCC
GTGGCCCTGATCGTCACCCTCTTGGCTTACACAGCCGGCCCCTTCACCTCGGCCTTCTTC
AATCCCGCCCTGGCGTCCGCCGTCACTTTCCACTGCTCC---------------------
------------GGAAACACCCTGCTTGAGTACGTCCAGGTCTATTGGCTCGGGCCCGCC
GCAGGTATGGTCACGGCCCTCCTGCTGCACCAGGGCAACATC------------------
---CCTCGACTCTTCCGGAGGAACCTCCTCTAC---------------------------
---------------TCTCAGAGGAGCAAGTACCGCGCCCCCAAGGCCAAGCTGGGCCCG
AGCCCGGGC------------------CCCCAGCCC---GCTGGGAAG------------
---CAGGAGAAAACCAGGAAGAAGAGGGCCGGCGGAGAGCCGGGCCTCAGA---------
GCCCAG
>Zebrafish aqp12
ATGTCGGGC------CTGAACTCTTCTCTGGGCTTCTTCCTGGCGGTGGTGGGTTTGAGT
GTGTCGGGCCGTCTTCTGCTGCGCCGA---------------------TGGACCGTCCTG
CTGGAGTTAGTCTCCGCG------TTCGCTCTGTGCGCCTGCAGACTGGAGGTGGACACC
------------------ATCGCAGAGGTGGGACAGTGGGCCGGAGCACTCGGGCCAGAT
GTTGCGGTCACAATGCTGTTCCTGTCCATCGCCGTACACACCGCCGTCATGCAGGACGTG
AGTGGAAACCCAGCGGTGACACTGCTGCGGCTCCTGCAGAGGGATGTCAGCGTAGTGGTG
GCAGTTCTGAGCATAGCGGCGCAGCTGATTGGAGCGTTTCTGGCTCTGGAAGTGGCCGGC
AGGTTCTGGGCGATGGAGATGAGCGAT---------------------------------
---ATGCACATGATCAAGAACCTGATGATGTCCGAGTGCAGCACGTCTCTG---AGGGTC
TCCACAGCTCTCGGGGTCTCCACTGAAGCTCTCGCTGCACTGCTGCTGCTCCTGTTGCAC
CTCGTCCTGAAGAAC---------------AGATCACAGATG---CTGAAGGTCCCTGCG
CTCTCTGTGGCGCTCACGCTCATCGCTTACACCGCAAACAACTACACATCTGGATATGTG
AATCCGGCCCTGGCCTACGCTGTGACCCTCACCTGTCCT---------------------

```
------------GGACACTCGTTCCTGGTGTATTCACTGGTCTACTGGCTCGGACCACTC
ATTGGTGTGTTTCTTGCAGTCTTCCTGTATTTGGGAAATATC------------------
---CCACTGCTGTTCAGCAAGAATCTGCTCTAC---------------------------
---------------TCCAAGAAAAATCGCTTCCGGCTGCCGAAAGGAAAA---------
------------------------------------------ACCAAT------------
---GATGAGAAGAGCAGC------------------------------------------
------
>Platypus AQP9
ATGAAGGAC----------------------------------------TCGGAGGGAAG
AGCGAAAGGACCTTCAAGGAGAGGCTCGTCCTCAAGAGCCGCCTGGCGAAGGAGAGTCTC
TCCGAGTTCCTGGGGACCCTCATCATGATCGTGCTCGGGTGTGGGTCCGTGGCCCAAGCC
------------------GTCCTCAGCCGGGGGCAGCTGGGCAACATAGTGACCATCAAT
GTGGGTTTTGCCATGGCCGTGGTCATGGCCATTTATGTGACCGGGGGCATATCCGGCGGC
CACATCAACCCGGCCGTGTCCTTCGCCATGTGCCTCTCTGGCCGGATGTCTTGGCTCAAG
CTGCCATTCTACGTGGCGGCCCAGTTCCTCGGGGCTTTCACCGGGGCCGCCTTGCTCTTC
GGCATCTACTACGATGCCCTCATGGCCTTCGCCGAA------GGAGAGCTACTCATCACC
GGAGAAAATGCCACGGCCTTCATCTTTGCCACATACCCGGCCGGGTACCTG---------
TCCCCGGCAAACGCGTTCGTGGACCAAGTGGCGTCCACCACTTTCCTGATCCTGGTGATC
TTCGCCGTCTTCGACACCAGGAACCTGGGGGTGCCCAGGGGC---CTGGAGCCGATCGTC
ATCGGACTGCTCATCGTTGTTCTTGCGTCCTCTCTGGGGCTGAACAGCGGCTGTGCCATG
AACCCGGCCCGGGACCTGGGCCCCAGGGTCTTCACCGCGGTGGCCGGGTGGGGGCTGGAG
GTCTTCACGGCTGGAAACCACTTC---------TGGTGGATCCCGGTGGTGGGGCCGATG
GTGGGAGCAGTCTTGGGCGTGCTGGTCTACCAGCTGTGCATCGCCATCCAGCTC------
---CCCCAGCCGGAG---------------------------------------------
---------------GCGGAGCAGGAGGGACAGGTCATGACCAAGATGGAGAAA------
------------------------------------------------------------
---TACGAACTCAGCGTCATGATG------------------------------------
------
>Zebrafish aqp9a
------------------------------------------------------------
------------ATGAAGCAGCACTGCGCGCTCAAACAGCGGCTCTTCAAGGAGTTTCTG
GCCGAGTTTTTGGGGACCTTCGTGCTGGTGCTGTTCGGCTGTGGGTCGGTGGCTCAGACA
------------------GTGTTGAGCAGAAACACACTCGGTGAACCGCTGACCATCCAC
ATCGGCTTCAGCACCGGCCTCATGATGGGCGTCTATGTGTCCGGCGGCGTCTCAGGCGGG
CACCTGAACCCGGCCGTGTCTCTGGCGATGGTGATTCTGGGGAAGCTGAAGATCTGGAAG
TTCCCGGTGTACGTGATCGCGCAGATGCTCGGAGCGTTTGCAGGAGCGGCCGCTGTGTTC
GGCCTGTACTACGACGCCTTCATGGAGTTCACCAGC------GGGATCCTGTCAGTGACG
GGCATTAACGCCACAGGACACATCTTCTCCTCGTACCCGGGCAGACACCTG---------
ACGGTCCTGGGCGGGTTCGTGGATCAGGTGGTGGGCACAGGGATGCTGGTGCTCTGTATT
CTCGCTATAGTGGACGGCAGGAACATCGGCGCCCCCAGAGGT---GTGGAGCCGCTGGCT
GTGGGTGTGGTGCTGCTGGGCATCAGCGTCTCCATGGGCCTGAACTGCGGATACCCCCTG
AACCCGGCCCGAGACCTGGGGCCCAGACTCTTCACCGCACTCGCCGGATGGGGGATGGAG
GTGTTCAGCACTGCAGATTACTGG---------TGGTGGATCCCAGTTGCCGGGCCGCTG
GTGGGGGGTGTCGTCGGAGCGGTGATCTACTTCCTGTTAATCGAGCTTCATCAC------
---TCCAATCACAACGACACACCACAGGAGGAGCCTGAGGAG------------------
---------------GAGGAGGATGAGGATGAAGAAGAGGAC------------------
------------------------------AGCAGCCTGAAGGACAAA------------
---TACGAGATGATCAACATGAGC------------------------------------
------
>Zebrafish aqp9b
ATGGAG---------------------------------------------TATCTCGAG
```

AACATCCGAAATCTGAGGGGGAGATGCGTCCTGAGGCGCGACATCATCCGAGAGTTTCTG
GCAGAATTACTCGGGACATTCGTGTTAATACTTTTCGGTTGCGGTTCAGTGGCCCAGACT
------------------GTCCTCAGCAGAGAAGCAAAAGGACAGCTTCTCACCATCCAT
TTTGGCTTTACTCTAGGGGTGATGCTGGCCGTCTACATGGCAGGAGGCGTGTCAGGAGGA
CATGTGAACCCTGCTGTTTCTTTGGCTATGGTTGTCCTGAGGAAACTCCCACTAAAGAAG
TTCCCTGTGTATGTGTTGGCCCAATTTCTAGGTGCCTTTTTTGGGTCTTGTGCCGTCTAC
TGTCTTTACTATGATGCCTTTACAGAATTTGCTAAT------GGAGAGCTAGCTGTAACT
GGCCCAAATGTCACAGCAGGTATCTTTGCATCATATCCACGTGAAGGACTC---------
TCATTGTTAAATGGATTCATTGATCAGGTGATTGGTGCAGGTGCCCTGGTCCTCTGTATT
TTAGCTGTTGTAGATAAGAAGAACATTGGAGCACCTAAAGGA---ATGGAGCCTCTGCTT
GTCGGTCTGAGCATCCTGGCTATTGGAGTGTCAATGGCACTAAACTGTGGATATCCTATA
AACCCTGCCAGAGACTTGGGACCTCGGCTGTTCACTGCCATTGCAGGATGGGGATTAACG
GTGTTCAGTGCTGGCAATGGCTGG---------TGGTGGGTTCCAGTGGTGGGGCCAATG
GTGGGCGGAGTGGTTGGTGCTGCTATCTACTTCCTGATGATCGAGATGCATCAC------
---CCTGAGAACGACAAGAACCTGGAAGACGAC---------------------------

------------------------------AACAGCCTTAAAGACAAA------------
---TATGAGCTGAACACCGTCAAC------------------------------------

>Platypus AQP13

------CAGAGTGCCCACCTGCGCTGTGGCCTGGCT---GAGTTCTGG------------
------------GGGACTTTTGTGCTGATTGTACTCGGCTGCGGCTGGGTGGCCCAGGCT
------------------------------GAGCTGCGAGGCTGG---------------
------------TTCATGGTCTCTGCGGGAGCCTACATAGGCTCCGGAGCCTCAGGCACT
CATCTGAACCCCGCCGTGTCCCTGGCCATGTGTTTCCTGCGGCGGCTGGACTGGAACCTC
CTGCCCACTTTGTGCTTAGCCCAGCTGGCCGGGGCCTTCTGCGGCGCAGCCACCGTCTTC
GTCTGGCACTACGACGGTCTCCAGGCCCTGAGCGCT------GGTGCCTGGACAGTGGCG
GGACCCAATGCCACAGCTGGGATCTTTGCTTCCTACCCCTCCGGGCAGCAG---------
AGTTCACTCAGCTGCTTTGCTGACCAGGTGATGGCGTCGGCAGCTTTTCTCACCTGTGTC
CTGGCCGTGCTG---GGCGAGGGAGTCCCAGCCCCCCGCCACAAGCTGCAGCCCCCCGCC
CAGCGGCTGGCCCTGTTCCTAGTAGGCTCGGCCTTGGGCTCGAGCTGTGGGTGCCCCATC
AACCCCGCCGAGGACCTGGGCCCGCGGGCGTTCGCGGCAGTGGCCGGCTGGGGGCTGGAA
GTCTTCAGGGTTGGAAACCACTGG---------TGGTGGATCCCCGTCCTGGGGCCCCTG
GTGGGCGCCCTGCTCGGGGCCTCCGTGTAC------------------------------

>Platypus AQP3
ATGGGCCGG--CAGAAGGAG
CTGGTGTCGCGCTGCGGGGAGATGCTGAGGATCCGCTACAAGCTGTTGCGGCAGGCCCTG
GCCGAGTGTCTGGGTACCCTCATCCTCGTGATGTTCGGCTGCGGCTCCGTGGCCCAGGTG
------------------ATACTTAGTCGTGGCACCCACGGCGACTTCCTCACCATCAAC
CTGGCCTTTGGCTTTGCTGTCACCCTGGGAATCCTCATTGCCGGTCAGGTCTCAGGTGCC
CACTTGAACCCGGCCGTGACATTTGCACTGTGTTTTCTGGCCCGGGAACCCTGGATCAAG
CTGCCCATCTATGCCTTGGCACAGACCCTAGGTGCGTTCCTGGGTGCTGGCATCATCTTC
GGCCTCTACTATGATGCCATCTGGGCCTTTGCGGAC------AACCAGCTGATTGTGTCC
GGCCCCAACGGCACTGCTGGGATCTTTGCAACCTACCCGTCCGGTCACTTG---------
GACACCCTCAACGGCTTCTTTGACCAGTTCATCGGCACGGCCTCCCTCATCGTCTGTGTC

CTGGCCATCGTGGATCCTTACAACAACCCCGTCCCCAGGGGC---CTGGAGGCCTTCACC
GTTGGTTTTGTCGTCTTGGTCATCGGCACCTCCATGGGCTTTAACTCTGGCTATGCCGTC
AATCCCGCCCGAGACTTCGGCCCCCGCCTCTTCACCGCCATCGCTGGATGGGGCTCTGAG
GTCTTCACGACGGGCAAGCACTGG---------TGGTGGGTGCCCATCGTCTCACCCCTT
CTGGGCTCCATCGCCGGAGTCTTCGTCTACCAGCTGATGATTGGCTGCCACATG------
---GAGGCCCCGCCCCCATCC---------------------------------------
---------------ACCGAGCAGGAGAACGTCAAGTTGGCCAACGTCAAGCAC------
---------------------------------------AAGGAGCGGATC---------

>Zebrafish aqp3a
ATGGGTTGG--CAGAAAAGC
GTTCTGGATAAGCTTGCGCAGACTTTCCAAATCCGCAACAAGTTACTGCGCCAGGGATTG
GCTGAATGCTTAGGAACTCTCATCCTGGTGATGTTTGGCTGTGGTTCATTGGCCCAGTTG
------------------AAACTAAGCGAAGGTTCTCATGGTCTCTTTCTCACTGCAAAC
CTTGCTTTTGGGTTTGGTGCTACTCTTGGAATCTTGGTTTGCGGCCAGGTGTCAGGCGGA
CATTTAAATCCTGCTGTTACATTTGCTCTCTGCCTCTTGGGAAGAGAAAAATGGAGAAAG
TTTCCTGTGTACTTTCTGTTCCAAACACTCGGATCCTTCTTGGGTGCTGCTATTATCTTT
GCCGAATACCATGACGCAATTTATGATTATGCTGGAGAATCAAATGAGTTGCTTGTACTG
GGTGAAAAAGAAACAGCTGGGATTTTTGCTACATACCCAAGCAAATATCTC---------
ACCCCCCTAAATGGATTTTTTGACCAGGTGATAGGCACAGCATCCCTGATTGTGTGCATC
CTGGCCATTGTGGACCCCTACAACAACCCGATCCCTCAAGGT---CTTGAGGCCTTCACA
GTGGGATTCAGCGTCCTTATCATTGGTCTCTCCATGGGCTTCAATTCTGGCTATGCAGTA
AACCCAGCTCGAGATTTTGGACCTCGTCTTTTTACTGCCATGGCTGGTTGGGGTAGTGAA
GTCTTCACAGCCAGGGATTATTGG---------TTTTTGGTGCCCATCTTTGCTCCGTTC
ATTGGAGCCGTTATTGGTGTGATTGTGTACCAGCTGATGGTGGGATGGCATGTG------
---GAAGGAGAGGCACGAGATAAGAAAGCTAAA---------------------------
---------------GCTAGAGAGGAGGTGATGAACCTCAATGACGTCGCCAGC------
---------------------------------------AAGGAA---------------

>Zebrafish aqp3b
ATGGGAAGA--CAGAAGGTA
ATCCTGGAAAAGATGGCTCGGATCTTTCAGATTCGGAACATGCTGATGAGACAAGCACTG
GCAGAATGCCTGGGCACCCTCATTCTAGTGATGTTTGGTTGTGGTGCTCTTGCCCAGCAT
------------------ATTTTAAGCGGAGGCTCTCATGGAATGTTTCTGACAGTGAAT
TTTGCATTTGGATTCGCTGCTACATTGGGAATCCTGGTTTGTGGGCAAGTCTCAGGAGGT
CACATAAACCCTACTGTGACCTTTTCTCTCTGTTTGTTGGGGAGGGAGCCCTGGAGGAAA
TTTCCCGTTTACTTTCTGGCCCAGACTGTGGGGGCTTTTCTTGGAGCTGGAATAATATTT
GGCATGTATTTTGATGCAATTTGGAAATTTGGACAA------GGTTCTCTTGATGTTGAT
GGGGTAAATGCAACTGCTGGAATCTTTGCTACGTACCCTTCTAAACACCTT---------
ACTTTGCTAAATGGATTCTTTGATCAGATGATCGGCACGGCAGCTCTGATCGTGTGTATC
CTTGCCATTGTTGACCCTTACAATAACCCCATCCCGCAAGGA---CTGGAGGCCTTCACT
GTGGGCTTTGTGGTTCTGGTGATTGGTCTGTCTATGGGATTTAACTCAGGCTATGCTGTA
AACCCAGCCAGAGACTTGGGACCACGGATCTTCACTGCAATTGCTGGATGGGGCTCAAAA
GTGTTCTCAGCGGAGTCTTACTGG---------TCCTTTGTGCCAGTCTTTGCCCCATTC
ATTGGTGCTGTGTTCGGTGTGATGGTGTATCAGTTGATGGTGGGATGCCATGTG------
---AAAGGAGAAGAAAGAGATAAAAGAGAAGCGGTGGAAAGA------------------
---------------GAAGAGAAGGAAAGACTCAAACTATCTGCCGTTTCTGAT------
---------------------------------------AAAGATGCAGCA---------

>Tasmanian devil AQP10
ATGACT--CAATTCACA
TTTTTGGCCAAGGTTAGGGCCCGGATCCGAATCCGTAATCTTCTAGTGCGACAATGCTTG
GCTGAATTTTTGGGTGTCTTTGTACTTATGATCATCACACAGGGGGCAGTGGCTCAGGCT
------------------GTTACCAGTGATGAATCCAAAGGCAACTTCTTCACCATGTTC
TTGGCAGGCGCCCTGGCTGTAACGATTGCCATCTATGTGGGAGGCAATGTCTCAGGAGCC
CACCTGAATCCTGCTTTCTCCTTGTCCATGTGCCTAATGGGTCGCCTACCTTGGACCAAA
TTTCCAATATATATTCTGGTACAGCTACTTTCTGCCTTCTTTGCCTCGGGAGCAACATAT
GTCCTCTACTATGATGCTCTCCAGAACTATACTGGT------GGAAACCTGACAGTGACT
GGCCCCAAGGAGACAGCCTCCATCTTTGCCACCTATCCAGCCCCTTATCTG---------
TCCCTGGAAAATGGCTTCCTGGACCAGATTCTAGGTACTGCTATGCTGATTGTGGGCATC
TTTGCCATTATAGACACCAAGAACAAGGGAGTGCCTGCAGGA---TTGGAGCCAGTAGCA
ATTGGTCTGCTGATTCTGGCCATTGGCCTCTCCATGGGTGCCAACTGCGGTTACCCACTG
AACCCTGCTCGGGACCTCGGACCTCGGCTCTTCACCTATGTGGCTGGCTGGGGACCTGAG
GTCTTTAGTGCTGGCAATGGTTGG---------TGGTGGGTGCCTGTGGTGGCTCCCATG
GTAGGTGCTGTGTTGGGCACTGCCACATACCAGTTGCTGGTGGGTTTGCATCAT------
---CCTGAAGATTTGGAGCCTGCCCCAGAAGAAAGATTC---------------------

>Zebrafish aqp10a
ATGAGTCAG---
---ATGAAGAAGATCATGAAGAGGATGAAGGTGAAAAATGAACTGGCACGACAGATTATG
GGAGAGATCTTGGGCACTTTTGTTCTTCTGTTGTTTGGTTGTGCTGCAGCGGCTCAGGTG
------------------AAAACCAGCAGAGAAACAAAGGGGCAGTTTCTGTCTGGTAAC
ATCGCCTTCTCTGTAGGTGTCATGTCTGCCATGTACCTCTGCAGGGCAGTATCAGGAGCT
CATCTAAACCCAGCTGTGTCTCTGAGTTTCTGTGTATTGGGAGACCTGGCCTGGATAAAG
CTGCTACCATATTCTCTCGCTCAAATTTTAGGGGCTTACCTTGCTTCAGGGCTTGTCTAT
CTCATCTACCATGATGCCATCATGGAGTTCAGTGGT------GGAGTTCTGACCGTATTT
GGCCCTAATGAAACAGCCAGTATCTTCGCCACTTACCCAACCGATGTAGTA---------
TCAGTGCAGACCAATTTCCTGGATCAGGTGGTTGGCACAGCCATGTTGATGCTGTGCATT
TTGCCTCTGAATGATAAGAGAAACGCCCCAGCTCCTGAAGCG---CTGCTCCCACCCATT
GTAGCCACTGTTGTTCTAGGGATTTCCATCTCAATGTCTGCTAATTGTGGAGCAGCCATA
AATCCAGCACGTGATCTTGGTCCACGACTCTTTACCTTTACAGCAGGCTGGGGCACTGAA
GTCTTTACGTGCTATGACTACTTC---------TTTTGGATCCCATTGGTGGCTCCTATG
GTAGGGGGTGTCCTGGGCTCCATCATTTATTTGGTTTTCATCCAGTGGCATCTG------
---CCTGAGCTTGAAGATGAATCTGAATCTGAGGAG------------------------
---------------ATGAATGATCAAACAAAAGTCATGGAGCACAACAACAAA------
--AAAGATGAGATATAC------
---CTTAAAATGTCTTCAATT---------------------------------------

>Zebrafish aqp10b

---ATGGACCGTCTGCTGAGGAGATGCCGAATCAAGAGTCGTCTGCCCAGAGAATGTCTG
GCGGAGTTTTTCGGAGTCTATGTTTTAATACTGTTCGGGTGTGGATCAGTGGCTCAGGTC
------------------ACCACCTCTCAGAATACCAAGGGAGAGTACCTGTCAATCAAC
CTGGGCTTCGCACTGGGAACCACATTTGGCATCTACATTGCAAAAGGAGTGTCAGGAGCT
CACCTGAATCCAGCGGTTTCCCTCAGTTTGTGTGTTTTGGGCAGGTTTTCGTGGACTCGT
CTTCCTTTCTACGTGTGTTCGCAGCTCTTCGGTGCATTTCTGGCTGCAGCAACGGTTGCC

CTGCAGTATTATGACGCCATAATGGATTTCACTGGA------GGGCATCTGACAGTCAGT
GGTGCCACGGCTACAGCGGGCATCTTCTCAACTTATCCAGCAGATTATCTG---------
AGTCTGTGGGGAGGAGTGGTAGACCAGATCATTGGCACGGCTGCTCTGCTGGTGTGTGTT
CTTGCATTGGGAGATGCTCATAACACACCTGCACCTGCAGGT---CTGGAGCCTGTACTT
GTCGGAGCCGCCGTGCTGGTGATCGGGATCTCCATGGGATCTAACAGCGGATATGCCATC
AATCCAGCCAGAGACTTCGGCCCAAGACTCTTCTCCTACATCGCAGGCTGGGGAGACGAG
GTGTTCAGGGCTGGACATGGATGG---------TGGTGGGTGCCTATAATCGTAACGTGT
GTCGGGGCTCTTCTGGGATCATTACTGTACGAGCTGCTGATTGGAGTTCATCAT------
---CCTGACTCAGAGGCAGTGGATCATGAAGACCCGACAGCG------------------
---------------GCGCTCCAGCAAACTGTCGAGATGGAGGGTGCGCAGAGT------
---------------------------TTTGACACCATTAAAGAAAACAAAAAGAGCGGG
ATCTTTTCTATAACCTCAGCAGACGTAGGG------------------------------

>Platypus AQP7
ATGGCC--
---GTCCAGAAGTTCTTGCTATCGTTCCTGACGGAGAACAAGCTGACCAGAGAGTTCCTG
GCAGAAATGCTGAGCACATTTGTCCTCATGGTGTTCGGGCTGGGGTCTGTGGCGCAGGTG
------------------GTGCTGGGAAAGCGGGAGTTCGGGGATTTTCTGAGCATTAAC
TTGGGCTTTGGCTTCGGCGTCACCATGGGCATACATGTGGCTGGAGGCATCTCGGGTGCT
CATATGAATGCTGCCATCACCTTTGCCTCCTGTGTCCTGGGCCAGCTACCATGGAAGAAG
TTACCCGTCTATGTGCTGGGCCAGTTTCTGGGTTCTTTCTTGGCTGCTGGCACCGTCTAC
TTGCTCTACAAAGATGCTCTCTATTCCTTCTCGGGA------GGAAACTTGACGGTGACG
GGGCCTAATTCAACAGCGGGGATCTTCTCAACTTACCCTGCTCCTTACATG---------
GACCTGCTTGGGGGATTTGTGAATGAGCTCATCGCCACGGCAATGCTCCAGCTCTGCATT
CTCGCCATTACGGACAAGAAGAACAGTGCTGCGCTGGATGGC---ACGCAGGCCCTCATC
ATCGGCCTCCTGGTGGCCGTCATTGGCATGTCCCTGGGCATGAACACGGGTTACGCCATC
AATCCTTCCCGGGACTTGCCACCCCGCATCTTCACCGCCATTGCGGGCTGGGGCATGGAT
GTCTTTCGGGCCGGCAATAGCTGG---------TGGTGGGTGCCCTTGATTGCCCCGACG
ATTGGCAGCGTCCTTGGAGCTCTGATTTACAAGATCCTCATCGACCATCACAACCGCCCG
GCCCCACAGCCGGAGTCCTGCATGACCTCGCCG---------------------------
---------------GCAGAGCCGGAG---------------------------------
---------------------------------------CCGGAGGCCACCTGCTTAGGC
---ATGGAGATGAAGGCG--

>Zebrafish aqp7
ATGGAAGAT---------------------------------GGCAGCATTCAAGGCCGC
ATGGCTCCAAATGTTGGATCCATGTTGAAGATCAAGAATGAATACATTCGAGTGGCTTTG
GCAGAAAGCCTCTGCACATTCATCATGATGGTGTTTGGCCTTGGCACTGTTGCACAAGTG
------------------GTTACAGGAGAAGGTTATTTTGGTGAATATCTCAGCATTAAT
ATAGGCTTTGGGCTGGCAGTGGCTATGGGTGTGCATGTTGGTGGAAAAGTGTCAGGAGCT
CATATGAACGCAGCTGTTTCATTCACAATGTGCGTGTTTGGCCGATTGCGCTGGAAGATG
CTGCCGCTGTATGCTTTCGCTCAGTTTCTGGGTTCATTCCTTGCCGCCGGGACCATTTTT
TCACTTTATTATGATGCCATAAATCATTTCTGCGGG------GGTAATTTGACTGTGTCC
GGACCCAAAGCAACAGCTGGGATCTTCGCCACATATCCAGCACCCTATATC---------
TCAGTCTACACTGGATTCTTTGATCAGGTTGCTGGCACGGGCCTGCTGTTGTTGTGTCTG
ATGGCTCTGTCAGACCAAAGGAACCAGCCGCTGGTGTCTGGA---GGTGAAGCCGTCGGT
GTGGGGCTTCTAGTGATGCTCATCGGCATCTCTATGGGGAGCAACAGCGGTTACGCCATC
AATCCCACACGGGACCTGGGGCCACGGCTCTTCACACTCATAGCAGGATGGGGCACAGAG
GTTTTTAGGGCAGGCAATTGCTGG---------TGGTGGGTACCCTTGGTGGCTCCTTTT
ATTGGAGGAGTTTTAGGGGCTTTAATCTACAAAGCACTTGTAGAACTACACCAC------
---CCTGATCTTAAAAACACTACAACACGGCCAGCA------------------------

```
---------------GTAGATCCTGAATGCATTCCTCTGGACAAGTGCAAGAAC------
---------------------------------------------------------GGC
AGAATAGAGATACCTGTG------------------------------------------
------
```

Supplementary Figure 13: Codon alignment used for Bayesian analyses of aqp14 sequences.

>MN168319
GAGATACGCAGTCGTCAGTTCTGGCGAGCAATGTTCTCAGAGCTGCTCGGTACTCTGGTG
CTGGTGAGCGTGGTTCTGGGCGCCTCCGTTCCGGGCCCCGGGGAGGCCCCCGTGGGACCC
TTGTACCCGGCGGTGGCAGTGGGCGTGGCCATCGTGGCGCTGGCGCACTGCTTTGGGGAG
ATGAGCGGCGCGCAGGTGAACCCCGCCCTCACTCTGGCCCTTTTGGCCACTCGGAGGCTG
GACGTCCTCAGGGCTCTCGTTTATATCGCCGCCCAGTGTCTGGGGGCCTGTTTAGGGGCC
GGGGCCCTCTACCTGGCCCTGCCGCTCAAGACCACAGCAGAACACTTCGTCAACAAGGTC
CCGATGCAGCTGAACGCGGCTCAGGCTCTGGGCGTTGAGGTCCTGTGCACCTTCCAGATG
GTCTTCACCGTTTTCTCCGTGGAGGAGCAGCGGCGGAGGGAGAACCCGGAACCAGGAAAT
CTGGCCATTGGATTCGCCCACTCTGCCGGAGTGCTGCTGGGGGCCCGGTTCTCTGGCGGA
AGCATGAACCCTGCGCGTTCTCTGGGTCCAGCCATCGTCGCCGGCTTCTGGGAAAACCAC
TGGGTCTACTGGTTCGGGCCGGTGATCGGAGCCATCTTGGCCGGCGTCTCCCACGACTTC
TTCTTCGCTCGCAGTGCGTCTCGCCAGAAGCTGGTGGCGTGTCTCTCCTGCAAGGACATC
GAGATCGTGGAGACCGCCAGCATGACGGGCTCGTCCCTGTCCACCGTGACCCAGAACGCC
ATGAGAGCCAAGCAGGGCAGCAAG---CAAGACAACAAC
>MN168320
GAGATACGCAGTCGTCAGTTCTGGCGAGCAATGTTCTCAGAGCTGCTCGGTACTCTGGTG
CTGGTGAGCGTGGTTCTGGGCGCCTCCGTTCCGGGCCCCGGGGAGGCCCCCGTAGGACCC
TTGTACCCGGCGGTGGCAGTGGGCGTGGCCATCGTGGCGCTGGCGCACTGTTTTGGGGAG
ATGAGCGGCGCGCAGGTGAACCCCGCCCTCACTCTGGCCCTTTTGGCCACTCGGAGGCTG
GACGTCCTCAGGGCTCTCGTTTATATCGCCGCCCAGTGTCTGGGGGCCTGTTTAGGGGCC
GGGGCCCTCTACCTGGCCCTGCCGCTCAAGACCACAGCAGAACACTTCGTCAACAAGGTC
CCGATGCAGCTGAACGCGGCTCAGGCTCTGGGCGTCGAGGTCCTGTGCACCTTCCAGATG
GTCTTCACCGTTTTCTCCGTGGAGGAGCAGCGGCGGAGGGAGAACCCGGAACCAGGAAAT
CTGGCCATTGGATTCGCCCACTCTGCCGGAGTGCTGCTGGGGGCCCGGTTCTCTGGCGGA
AGCATGAACCCTGCGCGTTCTCTGGGTCCAGCCATCGTCGCCGGCTTCTGGGAAAACCAC
TGGGTGTACTGGTTCGGGCCGGTGATCGGAGCCATCTTGGCCGGCGTCTCCCACGACTTC
TTCTTCGCTCGCAGTGCGTCTCGCCAGAAGCTGGTGGCGTGTCTCTCCTGCAAGGACATC
GAGATCGTGGAGACGGCCAGCATGACGGGCTCGTCCCTGTCCACCGTGACCCAGAACGCC
ATGAGAGCCAAGCAGGGCAGCAAG---CAAGACAACAAC
>MN168321
GAGATACGCAGTCGTCAGTTCTGGCGAGCAATGTTCTCAGAGCTGCTCGGTACTCTGGTG
CTGGTGAGCGTGGTTCTGGGCGCCTCCGTTCCGGGCCCCGGGGAGGCCCCCGTGGGACCC
TTGTACCCGGCGGTGGCAGTGGGCGTGGCCATCGTGGCGCTGGCGCACTGTTTTGGGGAG
ATGAGCGGAGCGCAGGTGAACCCCGCCCTCACTCTGGCCCTTTTGGCCACTCGGAGGCTG
GACGTCCTCAGGGCTCTCGTTTATATCGCCGCCCAGTGTCTGGGGGCCTGTTTAGGGGCC
GGGGCCCTCTACCTGGCCCTGCCGCTCAAGACCACAGCAGAACACTTCGTCAACAAGGTC
CCGATGCAGCTGAACGCGGCTCAGGCTCTGGGCGTCGAGGTCCTGTGCACCTTCCAGATG
GTCTTCACCGTTTTCTCCGTGGAGGAGCAGCGGCGGAGGGAGAACCCGGAACCAGGAAAT
CTGGCCATTGGATTCGCCCACTCTGCCGGAGTGCTGCTGGGGGCCCGGTTCTCTGGCGGA
AGCATGAACCCTGCGCGTTCTCTGGGTCCAGCCATCGTCGCCGGCTTCTGGGAAAACCAC
TGGGTGTACTGGTTCGGGCCGGTGATCGGAGCCATCTTGGCCGGCGTCTCCCACGACTTC
TTCTTCGCTCGCAGCGCGTCTCGCCAGAAGCTGGTGGCGTGTCTCTCCTGCAAGGACATC
GAGATCGTGGAGACGGCCAGCATGACGGGCTCGTCCCTGTCCACCGTGACCCAGAACGCC
ATGAGAGCCAAGCAGGGCAGCAAG---CAAGACAACAAC
>MN168322
GAGATGCGCAGTCGGCAGTTCTGGCAAGCCACGTTCTCGGAGCTGCTCGGGACTTTGGTG
CTGGTGAGCGTGGTTCTGGGCGCTTCGGTGCCCGGACCCGGGGAGGACCACGTGGGACCC

CTGTACCCGGCCATCGCAGTGGGCGTGGCCATCGTGGCGCTGGCCCACTGTTTTGGAGAA
ATGAGCGGAGCGCAGGTCAACCCGGCCCTCACTCTGGCCCTTTTGGCCACTCGGAGGCTG
GACGTCCTGCGGGCTCTGGTCTACATCACCGCCCAGTGCCTGGGGGCCTGCCTAGGGGCC
GGGGCCCTCTATTTGGCGCTGCCTCCGGAGACCACAGCCGAACATTTTGTCAACAAGGTG
CCCCTGCAGCTGAACGCGGCTCAGGCTCTGGGCGTGGAGGTGCTGTGCACCTTCCAGATG
GTCTTCACCGTCTTCTCCGTGGAGGAGCAGCGAAGGAGGGAGAACCCGGAGCCGGGGAAC
CTGGCCGTTGGGTTCGCCCACTCCGCTGGAGTGCTGCTGGGGGCTCGCTTCTCTGGCGGA
AGTATGAACCCCGCGCGTTCTCTGGGTCCCGCCATCGTCGCCGGCTTCTGGGAAAACCAC
TGGGTTTACTGGTTCGGCCCCGTGGTCGGGGCCGTCCTGGCCGGCGTGTCCCACGACTTC
CTCTTCGCCCGCAGCGCCTCGCGCCAGAAGCTGGTGGCCTGCCTCTCCTGCAAGGACATT
GAGATCGTGGAGACGGCCAGCATGATGGGGTCTTCCCTGTCCACCGTCACCCAGAACGCC
GGCAGGGCCAAGCAGGCCGGCAAA---CCGGACGGCAAC
>MN168323
GAGCTACGTAGTCGGCAGTTCTGGCGTGCCATGTTAGCTGAACTTCTCGGCACCTTGGTG
CTAGTGAGTGCCGTGCTGGGTGCCTCCGTGCCCGGCCCTGGAGAGGCCCCCGGGGGCCCC
ATGTACCCGGCGGTAGCGGTGGGTGTGGTGATTGTCGCACTGGCGCACTGTTTTGGCGAA
ATAAGTGGAGCACAGGTGAACCCTGCACTCACTCTGTCCCTGCTGGCCACGCGGAGGCTG
GATGTTCTCCGGGCCCTTGTTTATATCGCTGCACAGTGCTTGGGGGCCTCTTTAGGAGCC
GGAGCCCTCTACTTGGCCCTGCCACTCAAAACCAATGCAGACCACTTTGTCAACAAGGTT
CCCCTACAGCTGAACGCAGCCCAGGCTCTGGGCATAGAGGTTCTGTGCACCTTCCAGATG
GTCTTCACTGTGTTCTCAGTGGAGGATCAGCGACGGAGGGAAAGTCCAGAACCAGGAAAC
CTGGCCATTGGAGTAGCACACTCTGCCGGGGTGCTAATAGGGGTGAGGTTCTCTGGTGGA
AGCATGAACCCTGCACGTTCTCTGGGCCCGGCCATCATCACTGGCTTCTGGGAGAACCAC
TGGGTGTACTGGATCGGGCCCGTGCTCGGTGCCGTACTGGCAGGAGTGTCCCACGAGTTC
TTCTTTGCGCGCAGTGCGTCTCGTCAGAAGCTGGTGGCGTGTCTGACATGTAAGGACATC
GAGATTGTGGAGACGACCAGCATGACGGGATCGTCGCTGTCCACAGTCACGCAGAACGCC
ATGAGGGCCAAGCAGGCCAACAAA---CAGGACAACAAC
>MN168324
GAGCTGCGTTCTCGTCAGTTCTGGCGTGCCATCTTAGCCGAGCTGCTTGGCACCCTGGTG
TTAGTCAGTGCCGTGCTGGGCGCCTCTCTGCCGGGCCCTGGAGAGGCCCCCGGGGGCCCG
CTGTACCCAGCTGTAGCTGTGGGTGTGGTGATCGTTTCGCTGGCGCACTGTTTTGGAGAA
ATTAGCGGCGCGCAGGTCAACCCCGCCGTGACCCTGGCCCTCCTGGCCACCCGGAGGATG
GACGTCCCCAAAGCCCTGGTCTACATCGCTGCTCAGTGCGTGGGGGCCCTTCTGGGAGCC
GGGGCCCTCTTCTTGGCCCTTCCACACAAATCCACAGCAGAACACTTTGTGAACAGGGTC
CCCCTGGAGCTGAACGCGGGTCAGGCTCTGGCCGTGGAGGTTCTCTGCACCTTCCAGATG
GTCTTCACCGTGTTCTCAGTGGAAGAGCAGCGACGGAGAGAAAGCACGGAACCCGGGAAC
CTGACCATCGGATTGGCTCACACAGCTGGAGTCCTGATCGGGGTA------------GCG
TCCATGAATCCTGCTCGTTCTCTGGGCCCAGCCATCATCACGGGCTTCTGGGAGAACCAC
TGGGTATACTGGCTCGGACCGGTGCTCGGCGCCGTGCTGGGGGGCGTCTCCCACGAGTTC
TTCTTCGCTCAGAGCGCGTCCCGCCAGAAGCTGGTGGCCTACCTGACCTGCAAGGACATC
GAGATCGTGGAGACGACCAGCATCACCGGGTCGTCCCTGTCCACCGTCACGCAGAACGCC
ACCAGGGCCAAACACGCCAACAAG---CAGGACAAC---
>MN168325
GAGTTACGCAGTCGGCAGTTCTGGCGTGCCATACTTGCAGAGCTGCTCGGCACCCTGGTG
TTAGTAAGCACCGTTCTGGGTGCCTCTGCGCCCGGCCCTGGGGAGGCGCCTGGGGGACCC
CTGTACCCAGCAGTGGCAGTGGGTGTGGTTATTGTTGCACTGGCACACTGTTTTGGAGAA
ATAAGTGGAGCACAGGTGAACCCAGCTGTGACTCTGGCTCTGTTGGCCACTCGGAAGGTG
GATGTTCTCAGGGCTCTGGTTTATATCGCTGCACAGTGTTTAGGGGCCTCTTTAGGAGCC
GGGGCCCTCTACTTGGCCCTGCCACTCAAAACAAGCGCAGACCACTTTGTCAACAAGGTT
CCTATAGAGCTGAACGCAGCCCAGGCTGTGGGCATCGAGGTTTTGTGCACCTTCCAGATG
GTCTTCACTGTGTTCTCTGTGGAGGATCAGCGACGGAGGGAATGTACAGAACCAGGAAAC

CTGGCCATTGGATTAGCACACACTGCTGGTGTACTGATCGGGGCTCGGTTCTCTGGTGCC
AGCATGAATCCTGCACGTTCTCTGGGTCCAGCCATCATCACTGGCTTCTGGGAAAACCAC
TGGGTTTACTGGATCGGACCAGTGGTTGGTGCTGTACTGGGTGGAATGTCCCACGAGTTC
TTCTTTGCTCGCAGTGCATCTCGCCAGAAGCTGGTCGCATGTTTGACCTGTAAGGACATT
GAAATCGTGGAGACGACCAGCATGACCGGCTCATCTCTATCCACAGTCACACAGAACGCC
ATGAGAGCCAAGCACACCAACAAA---CAAGAAAACAAC
>MN168326
GAGTTACGCAGTCGGCAGTTCTGGCGTGCCATACTTGCAGAGCTGCTCGGCACCCTGGTG
TTAGTAAGCACCGTTCTGGGTGCCTCTGCGCCCGGCCCTGGGGAGGCACCTGGGGGACCC
CTGTACCCAGCAGTGGCAGTGGGTGTGGTGATTGTTGCACTGGCACACTGTTTTGGAGAA
ATAAGTGGAGCACAGGTGAACCCAGCTGTGACTCTGGCTCTGTTGGCCACTCGGAAGGTG
GATGTTCTCAGGGCTCTTGTTTATATCGCTGCACAGTGTTTGGGAGCCTCTTTAGGAGCC
GGGGCCCTCTACTTGGCCCTACCGCTCAAAACAAACGCAGACTACTTTGTCAACAAGGTT
CCTATAGAGCTGAACGCAGCCCAGGCTGTGGGCATCGAGGTTTTGTGCACCTTCCAGATG
GTCTTCACTGTGTTCTCCGTGGAGGATCAGCGACGGAGGGAATGCACAGAACCAGGAAAC
CTGGCCATTGGATTAGCACACACTGCTGGTGTACTGATCGGGGCTCGGTTCTCTGGTGCC
AGCATGAATCCTGCACGTTCTCTGGGTCCAGCCATCATCACCGGCTTCTGGGAAAACCAC
TGGGTGTACTGGATCGGACCAGTGGTTGGTGCTGTACTGGGAGGAGTGTCCCACGAGTTC
CTCTTTGCTCGCAGTGCATCTCGCCAGAAGCTGGTCGCATGTTTGACCTGTAAGGACATC
GAGATCGTGGAGACGACCAGCATGACCGGCTCATCGCTATCCACAGTCACACAGAACGCC
ATGAGAGCCAAGCACGGCAACAAA---CAAGAAAACAAC
>MN168327
GAGTTACGCAGTCGGCAGTTCTGGCGTGCCATACTTGCAGAGCTGCTTGGCACCCTGGTG
TTAGTAAGCGCCGTTCTGGGTGCCTCTGCGCCCGGCCCTGGGGAGGCGCCTGGGGGACCC
CTGTACCCAGCAGTGGCAGTGGGTGTGGTGATTGTTGCACTGGCACACTGTTTTGGAGAA
ATAAGTGGAGCACAGGTGAATCCGGCTGTCACTCTGGCTCTGTTGGCCACTCGGAAGGTG
GATGTTCTCAGGGCTCTTGTTTATATCGCTGCACAGTGTTTGGGGGCCTCTTTAGGAGCC
GGGGCCCTCTACTTGGCCCTACCGCTCAAAACAAGAGCAGACCACTTTGTCAACAAGGTT
CCTATAGAGCTGAACGCAGCCCAGGCTGTGGGCATCGAGGTTTTGTGCACCTTCCAGATG
GTCTTCACTGTTTTCTCCGTCGAGGATCAGCGACGGAGGGAAAGCACAGAACCAGGAAAC
CTGGCTATTGGATTAGCACACACTGCTGGTGTACTGATCGGGGCTCGGTTCTCTGGTGCC
AGCATGAATCCTGCACGTTCTCTGGGTCCAGCCATCATCACCGGCTTCTGGGAAAACCAC
TGGGTGTACTGGATCGGACCAGTGGTTGGTGCTGTACTGGGTGGAGTGTCCCACGAGTTC
CTCTTTGCTCGCAGTGCATCTCGCCAGAAGCTGGTCGCATGTTTGACCTGTAAGGACATC
GAGATCGTGGAGACGACCAGCATGACCGGCTCGTCGCTGTCCACAGTCACACAGAACGCC
ATGAGAGCCAAGCACGCCAGCAAA---CAAGAAAACAAC
>MN168328
GAGCTCCGTAGTCGGCAGTTCTGGCGTGCCATCCTCGCAGAGCTGCTCGGCACCCTGGTG
TTAGTGAGCGCCGTGCTGGGAGCCTCTGTGCCCGGCCCTGGAGAGGCCCCTGGGGGACCC
CTGTACCCAGCAGTGGCAGTGGGTGTGGTGATTGTTTCACTGGCACACTGTTTTGGAGAA
ATAAGTGGAGCACAGGTAAATCCTGCAGTGACTCTGTCTCTGTTGGCCACTCGGAAGATT
GAATTTCTCAGGGCCCTTGTTTACATCACTGCACAGTGTTTGGGGGCCTCTTTAGGAGCT
GGGGCACTCTACTTGGCCTTGCCACTGAAAACCACTGCAGACCACTTCGTCAACAGGGTT
CCTATGGAGTTGAATGCAGCACAGGCTCTGGGCATAGAGGTGTTGTGCACCTTCCAGATG
GTTTTCACTGTGTTCTCAGTGGAGGATCAGCGACGGAGGGAATGCACAGAACCAGGAAAC
CTGGCTATTGGATTAGCACACACCGCTGGAGTGCTGATAGGGGCAAGGTTCTCTGGTGCA
GGTATGAACCCTGCTCGTGCTCTGGGTCCGGCCATCATCACGGGCTTCTGGGAAAACCAC
TGGGTTTATTGGCTCGGACCAGTGATTGGTGGCATACTGGCTGGAGTGTCCCACGAGTTC
TTCTTTGCACGCAGTGCGTCACGCCAGAAGCTGGTGGCGTGTCTGACCTGCAAGGATATC
GAGATCGTGGAGACGACCAGCATGACCGGATCGTCGCTGTCCACGGTCACACAGAACGCC
ATGAGAGCCAAGCAGGCCAACAAA---CAAGAAAACAAC

>MN168329
GAGCTCCGTAGTCGGCAGTTCTGGCGTGCCATGCTCGCGGAGCTGATCGGCACCCTGGTG
TTAGTGAGCACCGTGCTGGGAGCCTCTTTGCCCGGCCCTGGAGAGGCCCTCGAGGGACCC
CTGTACCCAGCAGTGGCAGTGGGTGTGGTGATTGTTGCGCTGGCACACTGTTTTGGAGAA
ATAAGTGGAGCACAGGTAAATCCTGCGGTGACTCTGTCTCTGTTGGCCACCCGGAGGATT
GATGTTCTCAGGGCTCTTGTTTATATCACTGCACAGTGTTTGGGGGCCTCTTTAGGAGCT
GGGGCCCTCTACCTGGCCCTGCCACTGAAAACCACTGCAGACCACTTCGTCAACAGGGTT
CCTATAGAGTTGAATGCGGGCCAGGCTCTGGGCATAGAGGTGTTGTGCACCTTCCAGATG
GTCTTCACTGTGTTCTCAGTGGAGGATCAGCGACGGAGGGAATGCACAGAACCAGGAAAC
CTGGCTATTGGATTAGCACACACTGCTGGAGTGCTAATAGGGGCGAGGTTCTCTGGTGCA
GGTATGAACCCTGCTCGTGCTCTGGGTCCTGCCATCATCACCGGCTTCTGGGAAAACCAC
TGGGTGTATTGGATCGGACCAGTGACCGGTGCTGTACTGGGTGGAGTCTCCCACGAGTTC
TTCTTTGCGCGCAGTGCGTCACGC------------------------------------
------------------AGCATGACCGGATCATCGCTGTCCACAGTCACACAGAACGCC
ATGAGAGCCAAGCAGGCCAACAAA---CAAGAAAACAAC
>MN168330
GAGCTCCGTAGTCGGCAGTTCTGGCGTGCCATCCTTGCAGAGCTGCTCGGCACCCTGGTG
TTAGTGAGCGCCGTGCTGGGAGCCTCTGTGCCCGGCCCTGGAGAGGCCCCTGGGGGACCC
CTGTACCCAGCAGTGGCAGTGGGTGTGGTGATTGTTGCGCTGGCACACTGTTTTGGAGAA
ATAAGTGGGGCACAGGTAAATCCTGCAGTGACTCTGTCTCTGTTGGCTACTCGGAAGATT
GAATTTCTCAGGGCCCTAGTTTATATCACTGCACAGTGTTTGGGGGCCTCTTTGGGAGCT
GGGGCCCTCTACTTGGCCCTGCCACTGAAAACCACTTCAGACCACTTCGTCAACAGGGTT
CCTATAGAGCTGAATGCAGCGCAGGCTCTGGGCATAGAGGTGCTGTGCACCTTCCAGATG
GTCTTCACTGTGTTCTCAGTGGAGGATCAGCGACGGAGGGAATGCACAGAACCAGGAAAC
CTGGCTATTGGATTAGCACACACCGCTGGAGTGCTAATAGGGGGGAGGTTCTCTGGTGCA
GGTATGAACCCTGCTCGTGCTCTGGGTCCGGCCATCATCACTGGCTTCTGGGAAAACCAC
TGGGTGTATTGGCTCGGACCAGTGATCGGTGCTGTACTGGCTGGAGTGTCCCACGAGTTC
TTCTTTGCACGCAGTGCGTCACGCCAGAAGCTGGTGGCGTGTCTGACCTGTAAGGATATC
GAGATCGTGGAGACGACCAGCATGACCGGATCGTCGCTGTCCACGGTCACACAGAACGCC
ATGAGAGCCAAGCAGGCCAACAAA---CAAGAAAACAAC
>MN168331
GAGCTACGTAGTCGGCAGTTCTGGCGTGCCATACTTGCAGAGCTGCTCGGCACCCTGGTG
TTAGTGAGTGCCGTGCTGGGTGCCTCTGTGCCTGGCCCAGGAGAGGCACCTGGGGGACCC
ATGTATCCAGCAGTGGCAGTGGGTGTGGTGATTGTGGCACTTGCACACTGTTTTGGAGAA
ATAAGTGGAGCACAGGTCAACCCTGCAGTCACTCTGTCTATGTTGGCCACACGGAGGCTG
GATGTTCTCAGGGCCCTCGTTTATATCACTGCACAGTGTTTGGGGGCCTCTTTAGGAGCT
GGGGCCCTCTACCTGGCCCTGCCGCTCAAAACCACCGCAGACCACTTTGTCAACAGGGTT
CCTATAGAGTTGAATGCAGCACAGGCTCTGGGCATAGAGGTTTTGTGCACCTTCCAGATG
GTCTTCACCGTGTTCTCAGTGGAGGATCAACGACGGAGGGAAAGCCCAGAACCAGGAAAC
CTGGCCATTGGATTAGCACACACTGCTGGAGTGCTAATAGGGGTAAGATTCTCTGGTGCG
AGTATGAACCCTGCACGTTCTCTGGGTCCAGCCATCATCACTGGCTTCTGGGAAAACCAC
TGGGTGTATTGGATCGGACCAGTGCTCGGTGCTATACTGGCTGGAGTGTCCCACGAGTTC
TTCTTTGCACGCAGTGCATCTCGCCAGAAGCTGGTGGCGTGTCTGACCTGTAAGGATATC
GAGATTGTGGAAACGACCAGCATGACTGGATCGTCACTGTCCACGGTCACACAAAACGCC
ATGAGAGCCAAGCAGGCCAACAAA---CAAGAAAACAAC
>MN168332
GAGCTACGTAGTCGGCAGTTCTGGCGTGCCATACTTGCAGAGCTGCTCGGCACCCTGGTG
TTAGTGAGTGCCGTGCTGGGTGCCTCTGTGCCTGGCCCAGGAGAGGCACCTGGGGGACCC
ATGTATCCAGCAGTGGCAGTGGGTGTGGTGATTGTGGCACTTGCACACTGTTTTGGAGAA
ATAAGTGGAGCACAGGTCAACCCTGCAGTCACTCTGTCTATGTTGGCCACACGGAGGCTG
GATGTTCTCAGGGCCCTCGTTTATATTACTGCACAGTGTTTGGGGGCCTCTTTAGGAGCC

GGGGCCCTCTACCTGGCCCTGCCTCTCAAAACCACCGCAGACCACTTTGTCAACAGGGTT
CCTATAGAGTTGAATGCAGCACAGGCTCTGGGCGTAGAGGTTTTGTGCACCTTCCAGATG
GTCTTCACCGTGTTCTCAGTGGAGGATCAACGACGGAGGGAAAGCCCAGAACCAGGGAAC
CTGGCCATTGGATTAGCACACACTGCTGGAGTGCTAATAGGGGGGCGGTTCTCTGGTGCG
AGTATGAACCCTGCACGTTCTCTGGGTCCGGCCATCATCACTGGCTTCTGGGAAAACCAC
TGGGTTTATTGGATCGGACCAGTGCTCGGTGCTATACTGGCTGGAGTGTCCCACGAGTTC
TTCTTTGCGCGCAGTGCATCTCGCCAGAAGCTGGTGGCGTGTCTGACCTGTAAGGATATC
GAGATTGTGGAAACGACCAGCATGACCGGATCGTCACTGTCCACAGTCACACAAAACGCC
ATGAGAGCCAAGCAGGCCAACAAA---CAAGAAAACAAC
>MN168333
GAGCTACGTAGTCGGCAGTTCTGGCGTGCCATACTTGCAGAGCTGCTCGGCACCCTGGTG
TTAGTGAGTGCCGTGCTGGGTGCCTCTGTGCCTGGCCCAGGAGAGGCACCCGGGGGACCC
ATGTATCCAGCAGTGGCAGTGGGTGTGGTGATCGTGGCACTTGCACACTGTTTTGGAGAA
ATAAGTGGAGCACAGGTCAACCCTGCAGTCACTCTGTCTATGTTGGCCACTCGGAGGCTG
GATGTTCTCAGGGCCCTCGTTTATATCACTGCACAGTGTTTGGGGGCCTCGTTAGGAGCC
GGGGCCCTCTACCTGGCCCTGCCGCTCAAAACCACCGCAGACCACTTTGTCAACAGGGTT
CCTATAGAGTTGAATGCAGCACAGGCTCTGGGCATAGAGGTTTTGTGCACCTTCCAGATG
GTCTTCACGGTGTTCTCAGTGGAGGATCAACGACGGAGGGAAAGCCCAGAACCTGGAAAC
CTGGCCATTGGGTTAGCACACACTGCCGGAGTGCTAATAGGGGTACGGTTCTCTGGTGCG
AGTATGAACCCTGCACGTTCTCTGGGTCCGGCCATCATCACTGGCTTCTGGGAAAACCAC
TGGGTGTATTGGATCGGACCAGTGCTCGGTGCTATACTGGCTGGAGTGTCCCACGAGTTC
TTCTTTGCACGCAGTGCGTCTCGCCAGAAGCTGGTGGCGTGTCTGACCTGTAAGGATATT
GAGATTGTGGAAACGACCAGCATGACCGGATCGTCACTGTCCACAGTCACACAAAACGCC
ATGAGAGCCAAGCAGGCCAACAAA---CAAGAAAACAAC
>MN168334
GAGCTACGCAGTCGTCAGTTCTGGCGTGCCATGATGGCAGAGCTGCTGGGCACACTGGTT
TTAGTGAGTGCTGTGCTGGGGGCATCAGTTCCGGGCCTAGGAGAGGGCCCTGTGGGACCC
CTTTACCCAGCTATAGCAGTGGGAGTGGTGATTGTTGCAGTGGCACACTGTTTTGGAGAA
ATAAGTGGGGCGCAGGTGAACCCTGCAGTGACTCTGTCTCTGTTAGCCACTCGGAGGCTG
GATGTTCTCCGGGCCTTTGTTTATATAGCTGCACAGTGTTTGGGGGCTTCTTTGGGAACT
GGGGCCCTCTACCTGGCCTTGCCACTCAAAACCACAGCAGAGCACTTCGTCAACAGGGTT
CCGTTAGAGCTGAATGCAGCGCAGGCTCTGGGCATAGAGGTTTTATGCACCTTCCAGTTG
GTCTTTACTGTGTTTTCTGTAGAGGCTCAGCGACGAAGGGAAAGTCAAGAACCAGGAAAC
CTCGCCATCGGATTTGCCCACACAGCCGGAGTGCTAATAGGGGTAAGATTCTCTGGTGGG
AGTATGAATCCTGCACGTTCTCTGGGTCCAGCTATCATCACTGGCTTCTGGGAAAACCAC
TGGGTGTATTGGATCGGGCCAGTGCTCGGTGCTATACTGGCCGGAGTTTCCCATGAGTTC
TTCTTTTCGCTCAGTGCATCTCGCCAGAAGCTGGTGGCCTGTCTGACCTGTAAGGATATT
GAGATTGTTGAGACAGCCAGCATGACCGGATCATCACTTTCAACTGTCACACAGAACGCC
ATCAGAGCCAAACAGGCCAACAAG---CAAGAAAACAAC
>MN168335
GAGCTACGCAGTCGTCAGTTCTGGCGTGCCATGATGGCAGAGCTGCTGGGCACACTGGTT
TTAGTGAGCGCAGTGCTGGGGGCTTCAGTTCCGGGCCTGGGAGAGGGCCCTGTGGGACCC
CTTTACCCAGCTATAGCAGTGGGAGTGGTGATTGTTGCAGTGGCACACTGTTTTGGAGAA
ATAAGTGGAGCGCAGGTGAACCCTGCAGTAACTCTGTCTCTGTTAGCCACTCGGAGGCTG
GATGTTCTCCGGGCCCTTGTTTATATAGCTGCACAGTGTTTGGGGGCTTCTTTGGGAACC
GGGGCCCTCTACCTGGCATTACCACTCAAAACCACAGCAGAGCACTTCGTCAACAGGGTT
CCACTAGAGCTGAATGCAGCACAGGCTCTGGGCATAGAAGTTTTGTGCACCTTCCAGTTA
GTATTTACTGTTTTTTCTGTGGAGGCTCAACGACGGAGGGAAAGTCAAGAACCAGGAAAC
CTCGCCATCGGATTTGCACACACAGCCGGAGTGCTAATAGGGGTACGGTTCTCTGGTGGG
AGTATGAATCCTGCGCGTTCACTGGGTCCAGCTATCATCACAGGCTTCTGGGAAAACCAC
TGGGTGTATTGGATTGGACCAGTGCTCGGTGCGATTCTGGCCGGAGTTTCCCATGAGTTC

TTCTTTTCGCTCAGTGCATCTCGCCAGAAGCTGGTGGCCTGTCTGACCTGTAAGGATATT
GAGATTGTGGAGACAGCCAGCATGACCGGATCATCACTTTCAACTGTCACACAGAACGCC
ATTAGAGCTAAACAGGCCAACAAG---CAAGAAAACAAC
>MN168336
GAGCTTCGGAGTCGGCAGTTCTGGCGTGCCATGGTGGCAGAGCTGCTCGGCACCCTGGTG
TTAGTGAGCACCGTGCTGGGTGCATCTGTGCCGGGCCCTGGAGGGGCCCCCGTGGGACCC
CTGTACCCAGCAGTGGCAGTGGGTGTGGTGATTATTGCACTGGCACACTGTTTTGGAGAA
ATAAGTGGGGCACAGGTGAACCCTGCAGTGACTCTGTCCCTGTTGGCCACTCGGAGGGTG
GATGTTCTCAGGGCCGTTGTTTATATCGCTGCACAGTGTGTGGGGGCCTCTTTAGGAGCC
GGGGCCCTCTACCTGGCCCTGCCACTCAAAACCACCGCAGAACACTTCGTCAACAGGGTT
CCTATAGAGTTGAATGCAGCCCAGGCTCTGGGCATAGAGGTTTTGTGCACCTTCCAGATG
GTCTTC---GTGTTCTCAGTGGAGGATCAGAGACGGAGGGAGAGCCCAGAACCAGGAAAC
CTGGCGATTGGATTAGCACACACTGCTGGAGTGCTAATAGGGGCGCGGTTCTCTGGTGCA
AGCATGAACCCTGCACGCTCTCTGGGTCCAGCCATCATCACTGGCTTCTGGGAAAACCAC
TGGATTTATTGGATCGGACCGGTGCTCGGTGCTATACTGGCTGGAGTGTCCCACGAGTTC
TTCTTTGCGCGCAGTGCCTCTCGCCAGAAGCTGGTGGCATGTCTGACCTGTAAGGATATC
GAGATTGTGGAGACG---AGCATGACCGGATCATCACTGTCCACAGTCACCCAGAACGCC
ATGAGAGCAAAGCAGGCCAACAAA---CAAGAAAACAAC
>MN168337
GAGATACGTAGTCGGCAGTTCTGGCGCGCTATTCTTGCAGAGCTGCTTGGCACCCTGGTC
TTAGTGAGCGCCGTGTTGGGTGCCTCTGTGCCAGGCCCCGGAGAGGCCTCCGTGGGACCC
CTGTACCCAGCGGTGGCAGTGGGTGTTTCGATCATTGCACTGGGACACTGTTTTGGAGAA
ATAAGCGGGGCACAGGTGAACCCTGCTCTGACTCTGGCTCTGTTGGCCACTCGGAAGCTG
GAAGTTCTCAGGGCCGTCGTTTATATGGCTGCTCAGTGTTTGGGGGCCTGTTTAGGAGCC
GGGGCCCTCTACCTGGCTCTGCCAGTCAAAACCACTGCAGAACACTTCGTCAACAAGATT
CCTATAGAGCTGAATGCAGCTCAGGCTCTGGGCATCGAGGTTTTGTGCACCTTCCAGATG
GTCTTCACGGTTTTCTCAGTGGAGGATCAGCGACGGAGGGAAAGCCCAGAACCAGGAAAC
CTGGCCATTGGATTAGCACACACTGCTGGAGTGCTAATAGGGGCGCGGTTCTCTGGTGCT
AGTATGAACCCTGCACGTTCTCTGGGTCCAGCCATCATCACAGGCTTCTGGGAAAACCAC
TGGGTGTATTGGATCGGACCAGTGATCGGTGCTATACTTGCTGGAGTGTCCCATGAGTTC
TTTTTTGCACGCAGCGCCTCTCGCCAGAAGCTGGTGGCATGTCTGACCTGTAGGGACATC
GAGATTGTGGAGACGACCAGCATGACGGGATCATCTCTGTCCACAGTCACACAGAACGCT
ATGAGAGCCAAGCAGGCCAACAAA---CCAGAAAACAAC
>MN168338
GAGATACGTAGTCGGCAGTTCTGGCGCGCTATTCTTGCAGAGCTGCTTGGCACCCTGGTC
TTAGTGAGCGCCGTGTTGGGTGCCTCTGTGCCAGGCCCCGGAGAGGCCTCCGTGGGACCC
CTGTACCCAGCGGTGGCAGTGGGTGTGTCGATCATTGCACTGGGACACTGTTTTGGAGAA
ATAAGCGGGGCACAGGTGAACCCTGCTCTGACTCTGGCTCTGTTGGCCACTCGAAAGCTG
GAAGTTCTCAGGGCCGTCGTTTATATGGCTGCTCAGTGTTTGGGGGCCTGTTTAGGAGCC
GGGGCCCTCTACCTGGCTCTGCCAGTCAAAACCACTGCAGAACACTTCGTCAACAAGATT
CCTATAGAGCTGAATGCAGCTCAGGCTCTGGGCATCGAGGTTTTGTGCACCTTCCAGATG
GTCTTCACGGTTTTCTCAGTGGAGGATCAGCGACGGAGGGAAAGCCCAGAACCAGGAAAC
CTGGCCATTGGATTAGCACACACTGCTGGAGTGCTAATAGGG------------GGTGCT
AGTATGAACCCTGCACGTTCTCTGGGTCCAGCCATCATCACAGGCTTCTGGGAAAACCAC
TGGGTGTATTGGATCGGACCAGTGATCGGTGCTATACTTGCTGGAGTGTCCCATGAGTTC
TTTTTTGCACGCAGCGCCTCTCGCCAGAAGCTGGTGGCATGTCTGACCTGTAGGGACATC
GAGATTGTGGAGACGACCAGCATGACGGGATCATCTCTGTCCACAGTCACACAGAACGCT
ATGAGAGCCAAGCAGGCCAACAAA---CCAGAAAACAAC
>MN168339
GAGATACGTAGTCGGCAGTTCTGGCGCGCTATTCTTGCAGAGCTGCTTGGCACCCTGGTC
TTAGTGAGCGCCGTGTTGGGTGCCTCTGTGCCAGGCCCCGGAGAGGCCTCCGTGGGACCC

CTGTACCCAGCGGTGGCAGTGGGTGTGTCGATCATTGCACTGGGACACTGTTTTGGAGAA
ATAAGCGGGGCACAGGTGAACCCTGCTCTGACTCTGGCTCTGTTGGCCACTCGGAAGCTG
GAAGTTCTCAGGGCCGTCGTTTATATGGCTGCTCAGTGTTTGGGGGCCTGTTTAGGAGCC
GGGGCCCTCTACCTGGCTCTGCCAGTCAAAACCACTGCAGAACACTTCGTCAACAAGATT
CCTATAGAGCTGAATGCAGCTCAGGCTCTGGGCATCGAGGTTTTGTGCACCTTCCAGATG
GTCTTCACGGTTTTCTCAGTGGAGGATCAGCGACGGAGGGAAAGCCCAGAACCAGGAAGC
CTGGCCATTGGATTAGCACACACTGCTGGAGTGCTAATAGGGGCGCGGTTCTCCGGTGCT
AGTATGAACCCTGCACGTTCTCTGGGTCCAGCCATCATCACAGGCTTCTGGGAAAACCAC
TGGGTGTATTGGATCGGACCAGTGATCGGTGCTATACTTGCTGGAGTGTCCCATGAGTTC
TTTTTTGCACGCAGCGCCTCTCGCCAGAAGCTGGTGGCATGTCTGACCTGTAGGGACATC
GAGATTGTGGAGACGACCAGCATGACGGGATCATCTCTGTCCACAGTCACACAGAACGCT
ATGAGAGCCAAGCAGGCCAACAAA---CCAGAAAACAAC
>MN168340
GAGATACGTAGTCGGCAGTTCTGGCGCGCTATTCTTGCAGAGCTGCTTGGCACCCTGGTC
TTAGTGAGCGCCGTGTTGGGTGCCTCTGTGCCAGGCCCTGGAGAGGCCTCCGTGGGACCC
CTGTATCCAGCGGTGGCAGTGGGTGTGTCGATCATTGCACTGGGACACTGTTTTGGAGAA
ATAAGCGGGGCACAGGTGAACCCTGCTTTGACTCTGGCTCTGTTGGCCACTCGGAAGCTG
GAAGTTCTCAGGGCTGTCGTTTATATGGCTGCTCAGTGTTTGGGGGCCTGTTTAGGAGCC
GGGGCCCTCTACCTGGCTCTGCCAGTCAAAACCACTGCAGAACACTTCGTCAACAAGATT
CCTATAGAGCTGAATGCAGCTCAGGCTCTGGGCATCGAGGTTTTGTGCACCTTCCAGATG
GTCTTCACGGTTTTCTCAGTGGAGGATCAGCGACGGAGGGAAAGCCCAGAACCAGGAAAC
CTGGCCATTGGATTAGCACACACTGCTGGAGTGCTAATAGGGGCGCGGTTCTCCGGTGCT
AGTATGAACCCTGCACGTTCTCTGGGTCCAGCCATCATCACAGGCTTCTGGGAAAACCAC
TGGGTGTATTGGATCGGACCAGTGATCGGTGCTATACTTGCTGGAGTGTCCCATGAGTTC
TTTTTTGCACGCAGCGCCTCTCGCCAGAAGCTGGTGGCATGTCTGACCTGTAGGGACATC
GAGATTGTGGAGACGACCAGCATGACGGGATCATCTCTGTCCACAGTCACACAGAACGCT
ATGAGAGCCAAGCAGGCCAACAAA---CCAGAAAACAAC
>MN168341
GAGCTACGTAGTCGGCAGTTCTGGCGCGCCATCCTGGCCGAGCTGCTGGGCACCCTGGTC
TTAGTGAGCGCCATCCTGGGGGCCTCTGTGCCCGGCCCTGGGGGGGCCTCCCCGGGGCCG
TTGTACCCGGCGGTGGCGGTCGGAGTGTCGATCGTTGCGCTGGGACACTGTTTTGGAGAA
ATAAGTGGTGCTCAGGTGAACCCCGCTCTGACTCTGGCTCTGCTGGCCACGCGCCGGCTG
GAGGTCCTCAGGGCCGTCGGCTATCTGGCCGCTCAGTGTTCGGGGGCCTTCTTAGCCTCC
GGGGCCCTTTACCTCGCCCTGCCGCTCAAAACCACCGCGGACCACTTCGTCAGCAGGGTC
CCCCTGGAGGTGAACGCGGCCCAGGCAGTGGGCATCGAGGCCCTGTGCACCTTCCAGATG
GTCTTCACCGTGTTCTCGTCGGAGGAAATGCGACGGAGGGAAAGCCCAGAACCGGGACAC
CTGGCCATTGGCTTGGCACACACTGCCGGAGTGCTGATAGGGGCACGATTCTCAGGTGCC
AGTATGAACCCTGCACGTTCTCTGGGTCCGGCCATCGTCACTGGATTCTGGGAAAACCAC
TGGGTGTACTGGATCGGACCGGTGATGGGCGCCATCCTCGGTGGGGTGTCCCACGAGTTC
CTGTTCGCTCGTAGCGCCTCTCGCCAGAAGCTGGTGGCGTGTCTCACCTGCAAGGATATC
GAGATTGTGGAGACGGCCAGCATGACGGGATCGTCTCTGTCCACGGTGACGCAGAACGCC
---AGGGCCAAGCAGGCCAACAAA---CAGGAGAACAAC
>MN168342
GAGCTACGTAGTCGGCAGTTCTGGCGCGCCGTTCTGGCCGAGCTGCTGGGCACCCTGGTC
CTGGTGAGCGCCATCCTGGGGGCCTCCGTGCCTGGCCCCGGGGAGGCCCCCCCGGGCCCG
CTGTACCCGGCGGTGGCGGTCGGAGTGTCCATCGTTGTGCTGGGACACTGTTTTGGAGAA
ATAAGCGGTGCACAGGTGAACCCGGCTCTCACTCTGGCTCTGCTGGCCACGCGCAGGCTG
GAGGTCCTCAGGGCCGTCGGCTACCTGGCCGCTCAGTGTTCGGGGGCCTTTTTAGCCGCC
GGGGCCCTGTACCTCGCCCTGCCGCTCAAAACCACCGCGGACCACTTCGTCAACAAGGTC
CCTCTGGAGGTGAACGCGGCCCAGGCGGTGGGGATCGAGGTCCTGTGCACCTTCCAGATG
GTCTTCACCGTGTTCTCGGCGGAGGAAATGCGACGGAGGGAAAGCCCAGAACCGGGACAC

CTGGCCATCGGCTTGGCGCACACCGCCGGAGTGCTGATAGGGGCCCGATTCTCAGGCGCT
AGTATGAACCCGGCACGTTCTCTGGGCCCGGCCATCGTCGCAGGATTCTGGGAAAACCAC
TGGGTGTACTGGATCGGACCGGTGATGGGCGCCATCCTTGGTGGAGTGTCCCACGAGTTC
TTGTTCGCGCGCAGCGCCTCTCGCCAGAAGCTGGTGGCATGCCTGACCTGCAAGGACATC
GAGATCGTGGAGGCGGCCAGCATGACGGGGTCGTCTCTGTCCACGGTGACGCAGAACGCG
---AGGGCCAAGCAGGCCGGCAAA---CAGGAGAACAAC
>MN168343
GAGCTACGTAGTCGGCAGTTCTGGCGCGCCGGTCTCGCGGAGCTGCTCGGCACCCTGGTC
CTAGTGAGCGCCATCCTGGGTGCCTGCATGCCCGGCCCCGGAGAGGCCCCCCCGGGACCC
CTGTACCCGGCCGTGGCGGTCGGCGTGTCCATAGTCGCCCTGGCTCACTGTTTCGGAGAA
ATAAGCGGCGCACAGGTGAATCCGGCTCTGACTCTGGCTCTGTTGGCCACGCGGCGCCTG
GAGGTCCTCAGGGCCGTGGTCTATGTCGCTGCACAGTGTTTGGGGGCCTTTCTGGCAGCC
GGGGCCCTGTACCTGGCCCTGCCGATCAAAACCACCGCAGAACACTTCGTCAACAGGGTT
CCCATCGAGGTGAATGCAGCCCAGGCTCTGGGCATCGAGGTTTTGTGCACCTTCCAGATG
GTCTTCACCGTGTTCTCAGTGGAGGAACAGCGGCGGAGGGAAAGCCCAGAACCAGGAATC
CTGGCCATTGGATTAGCACACACCGCCGGAGTGCTAATAGGGGTAAGATTCTCCAGTGCG
AGTATGAACCCTGCACGCTCTCTGGGTCCGGCCATCATCACTGGATTCTGGGAAAACCAC
TGGGTGTATTGGATCGGGCCGGTGGTGGGGGCGATACTGGGCGGAGTCTCCCACGAGTTC
CTCTTTGCTCGCAGCGCCTCTCGCCAGAAGCTGGTGGCATGTCTGACCTGTAAGGACATC
GAGATTGTGGAGACGGCCAGCATGACGGGGTCGTCCCTGTCCACCGTGACGCAGAACGCC
---AGAGCCAAGCAGACCAACAAA---CAGGACAACAAC
>MN168344
GAGCTACGTAGTCGGCAGTTCTGGCGCGCCTTTCTTGCCGAGCTGCTCGGCACCCTGGTC
TTAGTGAGCGCCATACTGGGTGCCTCTATGCCAGGCCCTGGAGAGGCCCCCCCGGGACCC
CTGTACCCGGCAGTGGCAGTAGGTGTGTCGATTGTTGCGCTGGGACACTGTTTTGGAGAA
ATAAGCGGTGCACAGGTGAACCCTGCTTTGACTCTGGCTCTGTTGGCCACGCGGAGGCTG
GAGGTCCTCAGGGCCGTCGTTTATGTCGCTGCACAGTGTTTGGGGGCCTTTTTAGCAGCC
GGGGCCCTTTACCTGGCAATGCCGGTCAAAACCACTGCAGACCACTTCATCAACAGGGTT
CCTATAGAGGTGAATGCAGCCCAGGCTCTGGGCATCGAGGTTTTGTGCACCTACCAGATG
GTCTTCACTGTGTTCTCAGTGGAGGATCAGCGACGGAGGGAAAGCCCAGAGCCAGGAACC
CTGGCCATTGGATTAGCACACACTGCTGGAGTGCTAATAGGGGCACGATTCTCCGGTGCG
AGTATGAACCCTGCACGTTCTCTAGGTCCAGCCATCGTCACTGGATTCTGGGAAAACCAC
TGGGTGTATTGGATCGGACCAGTGATCGGTGCTATACTGGGCGGAGTGTCCCATGAGTTC
TTTTTTGCACGCAGCGCCTCTCGCCAGAAGCTGGTGGCATGTCTGACCTGCAAGGACATC
GAGATTGTGGAGGCGGCCAGCATGACGGGATCATCTCTGTCCACAGTCACACAGAACGCG
---AGAGCCAAGCAGACCAACAAA---CCAGAAAACAAC
>MN168345
GAGCTACGCAGTCGGCAGTTCTGGTGTGCCACTCTTGCAGAGCTGCTCGGCACCCTGGTC
TTAGTGAGCGCCATACTGGGTGCCTCTGTGCCCAGCCCCGGAGAGGCCCCCCCGGGACCC
CTGTACCCGGCAGTGGCAGTAGGAGTGTCGATTGTTGCGCTGGGACACTGTTATGGAGAA
ATAAGCGGTGCACAGGTGAACCCTGCTCTGACTCTGGCTCTGTTGGCCACGCGGAGGCTG
GAGGTTCTCAGGGCCGTTGTTTACATCGCTGCACAGTGTTTGGGGGCCTTTTTAGGAGCC
GGGGCCCTTTACCTGGCCCTGCCGGTCAAAACCACTGCAGACCACTTCGTCAACAGGGTT
CCTATAGAGGTGAATGCGGCCCAGGCTCTGGGCATCGAGGTTCTGTGCACCTTCCAGTTG
GTCTTCACTGTGTTCTCAGTGGAGGATCAGCGACGGAGGGAAAGCCCGGAACCAGGAAAC
CTGGCCATTGGATTAGCACACACTGCTGGAGTGCTAATAGGGGCCCGATTCTCCGGTGCT
AGTATGAACCCTGCACGTTCTCTGGGTCCAGCCATCATCACCGGCTTCTGGGAAAACCAC
TGGGTGTATTGGATCGGACCGGTGATCGGTGCCATACTGGGTGGAGTCTCCCATGAGTTC
CTCTTTGCACGTAGTGCCTCTCGCCACAAGCTGGTGGCATGTCTGACCTGTAAGGACATC
GAGATTGTGGAGACGGCCAGCATGACGGGATCATCTCTGTCCACAGTCACACAGAACGCG
---AGAGCCAAACAGACCACCAAA---CAAGAAAACAAC

>MN168346
GAGCTACTAAGTCGGCAGTTCTGGTTCGCCATCCTTGCTGAGCTGCTCGGCACCATGATC
GTAGTGAGCACTGTGCTGGGGGCCTCTGTGCCGGGCCCGGGAGAGGCCTCCACGGGACCC
CTGTACCCAGCAGTGGCAATAGGGGTGTCAATTATTGCGCTGTCACACTGTTTTGGAGAA
ATAAGCGGGGCACAGGTGAACCCTGCAGTGACTCTGGCTCTGTTGGCCACTCGCAGGCTG
GATGTTCTCAGGGCCGTTGTTTATATCGCTGCACAGTGTTTGGGGGCCACTTTAGGAGCC
GGGGCCCTCTACCTGGCCCTGCCAGTCAAATCCACTGCAGAGATCTTCGTCAACAGGGTT
CCCATAGAGCTGAATGCAGCCCAGGCTCTGGGCATGGAGGTGTTGTGCACCTTCCAGATG
GTGTTCACTGTGTTCTCAGTGGAGGATCAGCGACGGAGGGAAATGACAGAACCAGGAAAC
CTCGCCATAGGATTAGCCCACACTGCTGGAGTGTTAATGGCGGGGCGATTCTCCGGTGCC
AGTATGAACCCTGCACGCGCTCTGGGTCCAGCCATCATCATGGGTCTATGGGAAAACCAC
TGGGTGTATTGGCTTGGCCCGGTGATGGGCTCCATACTGGCCGGAGTGTCCCATGAGTTC
TTCTTTGCACGCAGCGCCTCTCGCCAGAAGTTTATGGCATGTCTGATCTGTAAGGACATC
GAGATTGTTGAGGCGGCCAGCATCACGGGATCGTCTCTGTCCACAGTCACACAGAACGCC
ATCAGAGCCAAACAGGCCAACAAC---------------
>MN168347
GAGCTACGCAGTCGGCAATTCTGGTGCGCCATCCTTGCTGAGCTGCTCGGCACCATGATC
CTAGTGAGCGCTGTGCTGGGGGCCTCTGTGCCGGGCCCGGGAGAGGCCTCCACGGGACCC
CTGTACCCAGCAGTGGCAGTAGGGGTGTCAATTATTGCGCTGATACACTGTTTTGGAGAA
ATAAGCGGGGCACAGGTGAACCCTGCAGTGACTCTGGCTCTGTTGGCCACTCGTAGGCTG
GATGTTCTCAGGGCCGTTGTTTATATCGCTGTACAGTGTTTGGGGGCCACTTTAGGAGCC
GGGGCCCTCTACCTGGCCCTGCCAGTCAAATCCACTGCAGACATCTTCGTCAACAGGGTT
CCCATAGAGCTGAATGCAGCCCAGGCTCTGGGCATGGAGGTGTTGTGCACCTTCCAGATG
GTTTTCACCGTTTTCTCAGTGGAGGATCAGCGACGGAGGGAAAGCACAGAACCAGGAAAC
CTCGCCATAGGATTAGCCCACACTGCTGGAGTGCTAATAGCGGGGCGCTTCTCCGGTGCC
AGTATGAACCCTGCACGCTCTCTGGGTCCAGCCATCATCACGGGTAAATGGGAAAACCAC
TGGGTTTATTGGCTTGGCCCGGTGATGGGCTCCATACTGGCAGGAGTGTCCCATGAGTTC
TTCTTTGCACGCAGCGCCTCTCGCCAGAAGTTTATGGCATGTCTGACCTGTAAGGACATC
GAGATTGTTGAGGCGGCCAGCATCACGGGATCGTCTCTGTCCACAGTCACACAGAACGCC
ATCAGAGCCAAACAGGCCAACAAC---------------
>MN168348
GAGCTACGAAGTCGGCAGTTCTGGCGTGCCATTCTTGCTGAGCTGCTTGGCACCATGTTC
TTAGTGAGCGCCGTCCTGGGTGCCTCTGTGCCAGGCCCTGGAGAGCCCCCCACGGGGCCC
CTGTACCCAGCAGTGGCAGTGGGCGTGTCGATTGTTGCAATGGCACACTGTTTTGGAGAA
ATAAGCGGGGCACAGGTGAACCCTGCAGTGACTCTGGCTCTGTTGGCCACTCGCAGGTTG
GATGTTCTCAGGGCCGTTGTTTATATCGCCGCTCAGTGTTTTGGGGCCTCTTTAGGAGCC
GGGGCCCTCTACCTGGCCCTGCCGCTCAAAACCACTGCAGAAAACTTCGTCAACAGGGTT
CCTATAGAGCTGAATGCAGCCCAGGCTCTGGGCATCGAGGTGTTGTGCACCTTCCAGATG
GTCTTCACTGTGTTCTCAGTTGAGGATCAGCGACGGAGGGAAAGCCCAGAACCAGGAAAC
CTGGCCATTGGATTAGCACACACTGCTGGAGTGCTAATAGGGGGGCGGTTTTCCGGTGCT
AGTATGAACCCTGCACGTTCTCTGGGTCCAGCCATCTTCACTGGCCTCTGGGAAAACCAC
TGGGTGTATTGGATCGGACCGGTG---GGTGCTATACTGGCTGGAGTGTCCCATGAGTTC
TTCTTTACTCGCAGCGCCTCTCGCCATAAGTTAGTGGCATGTCTGACCTGTAAGGACATC
GAGATTGTGGAGACGACCAGCATGACGGGATCCTCTCTGTCCACAGTCACACAGAACGCC
ATCAGAGCCAAGCAGACCAACAAA---CAAGAAAACAAC
>MN168349
GAGCTACGTAGTAAGCAGTTCTGGCGCGCCATTCTTGCAGAGCTGCTTGGCACCCTGGTT
TTAGTGAGCGCCGTACTGGGTGCATCTGTGCCAGGCCCTGGAGAGGCCCCCACGGGACCC
CTGTACCCAGCAGTGGCAGTGGGTGTGACGATTGTCGCACTGGGACACTGTTTTGGAGAA
ATAAGCGGGGCACAGGTGAACCCTGCAGTGACTCTGGCTCTGTTGGCCACTCGGAAGCTG
GAAGTGGTCAGAGCTGTTGTTTACATGGCTGCACAGTGTTTGGGGGCCTCTTTAGGAGCT

GGGGCCCTCTACTTGGCCCTGCCAGTCAAAACCACTGCAGACCACTTTGTCAACAGGGTT
CCTATAGAGTTAAATGCAGCCCAGGCTCTGGGCATGGAGGTTTTGTGCACCTTCCAGATG
GTCTTCACTGTGTTCTCAGTAGAGGATCAGCGGCGGAGGGAAAGCCCAGAACCAGGAAAC
CTGGCCATTGGATTAGCACACACTGCTGGAGTGCTAATAGGGGGGCGGTTCTCTGGTGCT
AGTATGAATCCCGCACGTTCTCTGGGTCCAGCCATCATCACTGGATTCTGGGAAAACCAC
TGGGTGTATTTGATCGGACCAGTGATCGGTGCTATACTGGCTGGAGTGTCCCATGAGTTC
TTCTTTGCACAAAGCGCCTCTCGCCAGAAGCTGGTGGCTTGTCTGACCTGTAAGGACATA
GAGATTGTGGAGACGACCAGCATGACGGGATCATCTCTGTCCACAGTCACACCAAACGCC
ATGAGAGCCAAGCAGGCCAACAAA---CAAGAAAACAAC
>MN168350
GAGCTACGTAGTCGGCAGTTCTGGCGTGCCATGCTGGCAGAGCTGCTCGGCACCCTGGTA
TTAGTAAGCGCCGTGCTGGGTGCCTCTGTGCCAGGCCCCGGCGAGGCCCCCGGGGGACCC
CTCTACCCAGCAGTGGCAGTGGGTGCTGTGATTGTTGTACTGGGTCACTGTTTTGGAGAA
ATAAGTGGGGCACAGGTGAACCCTGCTGTGACTCTGTCCCTGTTGGCCACTCGGAGGCTG
GATGTTCTCAGGGCCCTTGCTTATATCACTGCACAGTGTTTGGGGGCCTCTTTAGGAGCT
GGAGCCCTCTACCTGGCCCTGCCACTCAAAACCACTGCAGAATACTTCGTCAACAGGGTT
CCCATAGAGCTGAATGCAGCCCAGGCTCTGGGCATAGAGGCATTGTGCACCTTCCAGCTG
GTCTTCACTGTGTTCTCAGTGGAGGACCAGCGACGGAGGGAAAGCCCAGAACCAGGAAAC
CTGGCCATTGGATTAGCACACACTGCTGGCGTGCTAATAGGGGCACGGTTCTCTGGTGCA
AGTATGAACCCCGCACGTTCTCTGGGTCCGGCAATCATCACTAGCTTCTGGGAAAACCAC
TGGGTGTATTGGATGGGACCAGTGCTCGGTGCTATCCTGGCGGGAGTGTCCCATGAGTTC
TTCTTTGCACGCAGTGCCTCTCGCCAGAAGCTGGTGGCATGTCTAACCTGTAAGGATATC
GAGATTGTGGAGACGGCCAGCATGACCGGATCATCGCTGTCCACAGTCACGCAGAATGCC
ATGAGAGCCAAGCAGGCCAACAAA---CAGGAAAACAAC
>MN168351
GAGCTGCGTAGTCGACAGTTCTGGCGTGCCGTGCTGGCAGAGCTGCTCGGCACCCTGGTA
TTAGTGAGCGCCGTGCTGGGTGCCTCCATGCCAGGCCCCGGCGAGGCCCCCGGGGGTCCC
CTGTACCCAGCAGTGGCAGTGGGTGCGGTGATTGTCGCAATGGGACACTGTTTTGGAGAA
ATAAGTGGGGCACAGGTGAACCCTGCAGTGACTCTGTCTCTGTTGGCCACTCGGAGGCTG
GATGTTCTCAGGGCCCTTGTTTATATCGCTGCACAGTGTTTGGGGGCCTCTTTAGGAGCC
GGGGCCCTCTACCTGGCCCTGCCGCTGAAAACCACTGCAGAATACTTCGTCAACAGGGTT
CCTATAGAGCTGAATGCAGCCCAGGCTCTGGGCATAGAGGTTTTGTGCACCTTCCAGATG
GTCTTCACGGTGTTCTCAGCGGAGGATCAGCGACGGAGGGAAAGCCCAGAACCAGGAAAC
CTGGCCATTGGATTAGCACACACTGCTGGAGTGCTAGTAGGGGCGCGGTTCTCTGGTGCA
AGTATGAACCCTGCACGTTCTCTGGGTCCAGCCATCATCACTGGCTTCTGGGAAAACCAC
TGGGTTTATTGGATCGGACCAGTGCTCGGTGCTATACTGGCTGGAGTGTCCCATGAGTTC
TTCTTTGCACGCAGCGCCTCTCGCCAGAAGCTGGTTGCATGTCTGACCTGTAAGGATATT
GAGATTGTGGAGACGGCCAGCATGACCGGGTCATCACTGTCAACAGTCACGCAGAACGCC
ATGAGAGCCAAGCAGGCCAACAAA---CAAGAAAACAAC
>MN168352
GAGCTCCGCAGTCGGCAGTTCTGGAGCGCCGCCTTGGCAGAGCTGCTTGGCACCCTGGTG
TTAGTGAGCACCGTGCTGGGCGCCTCTGTTCCAGGGCCCGGCGAGGCCCCCGGGGGGCCC
CTGTATCCAGCTGTGGCAGTGGGTGTGGTCATTGTTGCACTGGCACACTGCTTTGGAGAA
ATAAGTGGAGCGCAGGTGAACCCGGCTGTCACGCTGTCCTTGCTGGCCACCCGGAAGCTG
GACGCTCTGCGGGCGCTCGTTTACCTGGTGGCTCAGTGTTTGGGGGCCTGTTTAGGGACC
TCGGCCCTCTACCTGGCCCTGCCCCTCAAAGCCACAGCAGAGCACTTTGTTAACAGGGTT
CCTGCAGATTTGAATGCAGCGCAGGCTCTGTGCATCGAGGTTCTGTGCACCTTTCAGATG
GTTTTCACCGTCTTCTCAGTGGAGGAGCAGAGACGGAAGGAGAGCGTGGAGCCAGGAAAT
CTGGCCATTGGGTTTTCTCACACTGCTGGAGTGCTAATAGGGGCTCGTTTCTCTGGTGCC
AGCATGAACCCGGCACGCTCCTTGGGTCCAGCCATCATCACTGGATTCTGGGAAAATCAC
TGGGTGTACTGGATCGGTCCCATCATCGGTGCTCTACTGGCAGGAGTGTCCCATGAGTTC

TTCTTCGCCCGCAGCGCCTCTCGCCAGAAGCTGGTGGCCTGTTTGACCTGCAAGGACATT
GAGATCGTTGAGACGGCCAGCATGACCGGCTCGTCTCTGTCCACAGTCACTCAGAACGCC
ATGAGAGCCAAACATGCCAACAAA---CAGGAAAGCAAC
>MN168353
GAGCTCCGCAGTCGGCAGTTCTGGAGCGCCGCCTTGGCAGAGCTGCTTGGCACCCTGGTG
TTAGTGAGCACCGTGCTGGGCGCCTCTGTTCCAGGGCCCGGCGAGGGCCCCGGGGGGCCC
CTGTATCCAGCTGTGGCAGTGGGTGTGGTCATTGTTGCACTGGCACACTGCTTTGGAGAA
ATAAGTGGAGCGCAGGTGAACCCGGCTGTCACGCTGTCCTTGCTGGCCACCCGGAAGCTG
GACGCTCTGCGGGCGCTCGTTTACCTGGTGGCTCAGTGTTTGGGGGCCTGTTTAGGGACC
TCGGCCCTCTACCTGGCCCTGCCCCTCAAAGCCACAGCAGAGCACTTTGTTAACAGGGTT
CCTGCAGATTTGAATGCAGCGCAGGCTCTGTGCATCGAGGTTCTGTGCACCTTTCAGATG
GTTTTCACCGTCTTCTCAGTGGAGGAGCAGAGACGGAAGGAGAGCGTGGAGCCAGGAAAT
CTGGCCATTGGGTTTTCTCACACTGCTGGAGTGCTAATAGGGGCTCGTTTCTCTGGTGCC
AGCATGAACCCGGCACGCTCCTTGGGTCCAGCCATCATCACTGGATTCTGGGAAAATCAC
TGGGTATACTGGATCGGTCCCATCATCGGTGCTCTACTGGCAGGAGTGTCCCATGAGTTC
TTCTTCGCCCGCAGCGCCTCTCGCCAGAAGCTGGTGGCCTGTTTGACCTGCAAGGACATT
GAGATCGTTGAGACGGCCAGCATGACCGGCTCGTCTCTGTCCACAGTCACTCAGAACGCC
ATGAGAGCCAAACATGCCAACAAA---CAGGAAAGCAAC
>MN168354
GAGCTCCGCAGTCGGCAGTTCTGGAGCGCCGCCTTGGCAGAGCTGCTTGGCACCCTGGTG
TTAGTGAGCGCCGTGCTGGGCGCCTCTGTTCCAGGGCCCGGCGAGGCCCCCGGGGGGCCC
CTGTATCCAGCTGTGGCAGTGGGTGTGGTCATTGTTGCACTGGCACACTGCTTTGGAGAA
ATAAGTGGAGCGCAGGTGAACCCGGCTGTCACGCTGTCCTTGCTGGCCACCCGGAAGCTG
GACGCTCTGCGGGCGCTCGTTTACCTGGTGGCTCAGTGTTTGGGGGCCTGTTTAGGGACC
TCGGCCCTCTACCTGGCCCTGCCCCTCAAAGCCACAGCAGAGCACTTTGTTAACAGGGTT
CCTGCAGATTTGAATGCAGCGCAGGCTCTGTGCATCGAGGTTCTGTGCACCTTTCAGATG
GTTTTCACCGTCTTCTCAGTGGAGGAGCAGAGACGGAAGGAGAGCGTGGAGCCAGGAAAT
CTGGCCATTGGGTTTTCTCACACTGCTGGAGTGCTAATAGGGGCTCGTTTCTCTGGTGCC
AGCATGAACCCTGCACGCTCCTTGGGTCCAGCCATCATCACTGGATTCTGGGAAAATCAC
TGGGTATACTGGATCGGTCCCATCATCGGTGCTCTACTGGCAGGAGTGTCCCATGAGTTC
TTCTTCGCCCGCAGCGCCTCTCGCCAGAAGCTGGTGGCCTGTTTGACCTGCAAGGACATT
GAGATCGTTGAGACGGCCAGCATGACCGGCTCGTCTCTGTCCACAGTCACTCAGAACGCC
ATGAGAGCCAAACATGCCAACAAA---CAGGAAAGCAAC
>MN168355
GAGCTCCGCAGTCGGCAGTTCTGGAGCGCCGCCTTAGCAGAGCTGCTTGGCACCCTGGTG
TTAGTGAGCGCCGTGCTGGGCGCCTCTGTTCCAGGGCCCGGCGAGGCCCCCGTGGGGCCC
CTGTATCCAGCAGTGGCAGTGGGTGTGGTCATTGTTGCATTGGCACACTGCTTTGGAGAA
ATAAGTGGAGCGCAGGTGAACCCGGCTGTCACGCTGTCCTTGCTGGCCACCCGGAAGCTG
GACGCTGTGCGGGCTCTCGTTTACCTGGTGGCTCAGTGTTTGGGGGCCTGTTTAGGGGCC
TCGGCCCTCTACCTGGCCCTGCCCCTCAAAGCCACAGCAGAGCACTTTGTTAACAGGGTT
CCTGCAGATTTGAATGCAGCGCAGGCTCTGTGCATCGAGATTCTGTGCACCTTTCAGATG
GTTTTCACCGTCTTCTCAGTGGAGGAGCAGAGACGGAAGGAGAGCGTGGAACCAGGAAAT
CTGGCCATTGGGTTTTCTCACACTGCTGGAGTTCTAATAGGGGCTCGTTTCTCTGGTGCC
AGCATGAACCCGGCACGCTCCTTGGGTCCAGCCATCATCACTGGATTCTGGGAAAATCAC
TGGGTATACTGGATCGGTCCCATCATCGGCGCTCTACTGGCCGGAGTGTCCCATGAGTTC
TTCTTCGCCCGCAGCGCCTCTCGCCAGAAGCTGGTGGCCTGTTTGACCTGCAAGGACATC
GAGATCGTTGAGACGGCCAGCATGACCGGCTCGTCTCTGTCCACAGTCACTCAGAACGCC
ATGAGAGCCAAACATGCCAACAAA---CAGGAAAGCAAC
>MN168356
GAGCTCCGCAGTAGGCAATTCTGGAGTGCCGTCTTAGCAGAGCTGCTTGGCACCCTGGTG
TTAGTGAGCGCTGTGCTGGGTGCCTCTGTTCCAGGGCCCGGCGAGGCCCCAGTGGGGCCC

CTGTATCCAGCTGTGGCAGTGGGTGTGGTCATTATTTCACTGGCACACTGCTTTGGAGAA
ATAAGTGGAGCGCAGGTGAACCCGGCTGTCACACTGTCCTTGCTGGCCACCCGGAAGCTG
GACGCTCTGCGGGGGGTCGTTTACATGGTGGCTCAGTGTTTGGGGGCCTGTGTAGGGGCC
TCGGCCCTCTATCTGGCTCTGCCCCTCAAAGCCACAGCTGAGCACTTTGTTAACAGGGTT
CCTGCAGATTTGAATGCAGCGCAGGCTCTGTGCATCGAGGTTCTGTGCACCTTTCAGATG
GTTTTCACCATCTTCTCAGTGGAGGAGCAGAAACGGAAGGAGAGCGTGGAACCAGGAAAT
CTGGCCATTGGACTTTCTCACACTGCTGGAGTGCTAATAGGGGCCCGTTTCTCTGGTGCC
AGTATGAACCCGGCTCGCTCCTTGGGTCCAGCCATCATCACTGGATTCTGGGAAAATCAC
TGGGTATACTGGATCGGTCCGATCATCGGCGCTCTACTGGCCGGAGTGTCCCATGAGTTC
TTCTTCGCCCGCAGCGCCTCTCGCCAGAAGCTGGTGGCCTGTTTGACCTGCAAGGACATT
GATATTGTTGAGACGGCCAGCATGACCGGTTCGTCTCTGTCCACAGTCACTCAGAACGCC
ATGAGAGCCAAACATGCCAACAAA---CAGGAAAGCAAC
>MN168357
GAGCTCCGCAGTAGGCAATTCTGGAGTGCCGTCTTAGCAGAGCTGCTTGGCACCCTGGTG
TTAGTAAGCGCCGTGCTGGGTGCCTCTGTTCCAGGGCCCGGCGAGGCCCCAGTGGGGCCC
CTGTATCCAGCTGTGGCAGTGGGTGTGGTCATTATTTCACTGGCACACTGCTTTGGAGAA
ATAAGTGGAGCGCAGGTGAACCCGGCTGTCACGCTGTCCTTGCTGGCCACCCGGAAGCTG
GACGCTCTGCGGGGGGTTGTTTACATGGTGGCTCAGTGTTTGGGGGCCTGTGTAGGGGCC
TCGGCCCTCTATCTGGCTCTGCCCCTCAAAGCCACAGCTGAGCACTTTGTTAACAGGGTT
CCTGCAGATTTGAATGCAGCGCAGGCTCTGTGCATCGAGGTTCTGTGCACCTTTCAGATG
GTTTTCACCATCTTCTCAGTGGAGGAGCAGAGACGGAAGGAGAGCGTGGAACCAGGAAAT
CTGGCCATTGGACTTTCTCACACTGCTGGAGTGCTAATAGGGGCCCGTTTCTCTGGTGCC
AGTATGAACCCGGCTCGCTCCTTGGGTCCAGCCATCATCACTGGATTCTGGGAAAATCAC
TGGGTATACTGGATCGGTCCGATCATCGGCGCTCTACTGGCCGGAGTGTCCCATGAGTTC
TTCTTCGCCCGCAGCGCCTCTCGCCAAAAGCTGGTGGCCTGTTTGACCTGCAAGGACATT
GAGATCGTTGAGACGGCCAGCATGACCGGCTCGTCTCTGTCCACAGTCACTCAGAACGCC
ATGAGAGCCAAACATGCCAACAAA---CAGGAAAGCAAC
>MN168358
GAGCTCCGCAGTAGGCAATTCTGGAGTGCCGTCTTAGCAGAGCTGCTTGGCACCCTGGTG
TTAGTGAGCGCTGTGCTGGGCGCCTCTGTTCCAGGGCCCGGCGAGGCCCCAGTGGGGCCC
CTGTATCCAGCTGTGGCAGTGGGTGTAGTCATTATTTCACTGGCACACTGCTTTGGAGAA
ATAAGTGGAGCGCAGGTGAACCCGGCTGTCACGCTGTCCTTGCTGGCCACCCGGAAGCTG
GACGCTCTGCGGGCGGTCGTTTACATGGTGGCTCAGTGTTTGGGGGCCTGTGTAGGGGCC
TCGGCCCTCTATCTGGCTCTGCCCCTCAAAGCCACAGCAGAGCACTTTGTTAACAGGGTT
CCTGCAGATTTGAATGCAGCGCAGGCTCTGTGCATCGAGGTTCTGTGCACCTTTCAGATG
GTTTTCACCATCTTCTCAGTGGAGGAGCAGAGACGGAAGGAGAGCGTGGAACCAGGAAAT
CTGGCCATTGGACTTTCTCACACTGCTGGAGTGCTAATAGGGGCCCGTTTCTCTGGTGCC
AGTATGAACCCGGCACGCTCCTTGGGTCCAGCCATCATCACTGGATTCTGGGAAAATCAC
TGGGTATACTGGATCGGTCCGATCATCGGCGCTCTACTGGCCGGAGTGTCCCATGAGTTC
TTCTTCGCCCGCAGCGCCTCTCGCCAGAAGCTGGTGGCCTGTTTGACCTGCAAGGACATT
GAGATCGTTGAGACGGCCAGCATGACCGGCTCGTCTCTGTCCACAGTCACTCAGAACGCC
ATGAGAGCCAAACATGCCAACAAA---CAGGAAAGCAAC
>MN168359
GAGCTCCGCAGTCGGCAGTTCTGGAGCGCCGTCTTAGCAGAGCTGCTTGGCACCCTGGTG
TTAGTGAGCGCCGTGCTGGGCGCCTCTGTTCCAGGGCCCGGCGAGGTCCCCGGGGGGCCC
CTGTATCCAGCGGTGGCAGTGGGTGCGGTCATTGTTGCACTGGCACACTGCTTTGGAGAC
ATAAGTGGAGCGCAGGTGAACCCGGCTGTCACGCTGTCCTTGCTGGCCACCCGGAAGCTG
GACGCTCTGCGGGCCCTCGTTTACATGGTGGCTCAGTGTTTGGGGGCCTGTTTAGGGGCC
TCGGCCCTCTATCTGGCTCTGCCGCTCAAAGCCACAGCAGAGCACTTTGTTAACAGGGTT
CCTGTAGATTTGAATGCAGCGCAGGCTCTGTGCATCGAGGTTCTGTGCACCTTTCAGATG
GTTTTCACCGTCTTCTCAGTGGAGGAGCAGAGACGGAAGGAGAGCGTGGAACCAGGAAAT

CTGGCCATTGGATTTTCTCACACTGCTGGAGTGTTAATAGGGGCTCGTTTCTCTGGTGCC
AGTATGAACCCGGCACGCTCCTTGGGTCCAGCCATCATCACTGGATTCTGGGAAAATCAC
TGGGTGTACTGGATCGGTCCGATCATCGGCGCTCTACTGGCCGGAGTGTCCCATGAGTTC
TTCTTCGCCCGCAGCGCCTCTCGCCAGAAACTGGTGGCCTGTTTGACCTGCAAGGACATT
GAGATCGTTGAGACGGCCAGCATGACCGGATCGTCTCTGTCCACAGTGACTCAGAACGCC
ATGAGAGCCAAACATGCCAACAAA---CAGGAAAGCAAC
>MN168360
GAGCTACGCAGTCGGCAGTTCTGGAGGGCCATTTTAGCAGAGCTGCTCGGCACCATGGTG
TTAGTGAGCGTCGTCCTGGGGGCCTCTGTCCCAGGGCCCGATGGGGCCCCTGTGGGCCCC
TTGTATCCAGCTGTAGCAGTGGGTGCGGTCATTGTCTCGCTGGCACATTGCTTTGGAGAA
ATAAGCGGAGCACAGGTGAACCCCGCCGTCACGTTGTCCTTGCTGGCCACCCGCAAGCTG
GATGTCCTCCGCGCCCTCGTCTACATGGTGGCGCAGTGTTTGGGGGCCTGTTTGGGAACC
TCGGTCCTCTACCTCGCTCTGCCCGTCAAAGCCACAGCAGGCCACTTTGTTAACAGGGTT
CCCATAGATCTGAATGCGGCGCAGGCTCTGTGCATCGAGGTGCTGTGCACCTTTCAGATG
GTCTTCACCGTCTTCTCAGTGGAGGAGCAGAGACGCAGGGAAAGCGTAGAACCAGGAAAT
CTGGCCATTGGATTTGCTCACACTGCTGGAGTGCTAATAGGGGCACGGTTCTCTGGTGCC
AGTATGAATCCAGCTCGCTCCTTGGGCCCAGCCACCATCACTGGATTCTGGGAAAATCAC
TGGGTATACTGGATCGGTCCAATCTTCGGGGCTCTGCTCGCCGGAGTGTCCCACGAGTTC
TTCTTCGCCCGCAGCGCCTCTCGCCAGAAGCTGGTGGCCTGTTTGACCTGCAAGGACATT
GAGATCATTGAGACGGCCAGCATGACTGGCTCGTCTCTGTCCACGGTTACTCAGAACGCT
ATGAGAGCCAAGCATGCCAACAAG---CAGGAAGGCAAC
>MN168361
GAGCTCCGTAGTCGGCGGTTCTGGAGTGCCACGTTGGCTGAGCTGCTCGGCACCCTGGTG
TTAGTGAGCGCTGTTCTGGGTGCCTCTGTTCCAGGGCCCGACCAGGCCCCCGTGGGACCC
CTATACCCAGCTGTAGCAGTGGGTGTTGTCATCGTCTCACTGGCACACTGCTTTGGAGAA
ATCAGTGGGGCGCAGGTGAATCCAGCTCTGACTCTCTCCTTGCTGGCCACCCGGAAGCTG
GATCTTCTCCATGCCCTCATTTACATGGTGGCTCAGTGTTTGGGGGCCTGTTTGGGAGCC
TCGGCCCTCTACCTCGCTCTACCTCTCAAATCCACAGCAGACCATTTTGTTAACAGGGTT
CCCATAGAATTGAATGCAGCACAGGCTCTGTGGATCGAGGTTCTCTGCACTTTTCAGATG
GTCTTCACCGTCTTCTCTGTGGAGGAGCACAGACGGAAGGAGAGCGTAGAACCAGGAAAT
CTGGCCATTGGATTTGCTCACTCTGCTGGAGTGATGATAGGCGCCCGTTTCTCTGGTGCC
AGTATGAATCCAGCACGCTCCTTGGGTCCAGCTATCATCACTGGATTCTGGGAAAATCAC
TGGGTATTTTGGATTGGTCCAATCATCGGTGCTCTTCTAGCTGGAGTGTCCCACGAGTTC
TTCTTCGCCCGCAGTGCCTCTCGCCAGAAGCTGGTGGCCTGTTTGACCTGCAAGGACATT
GAAATCATTGAGACGGCCAGCATGACTGGGTCATCTCTGTCCACAGTGACTCAGAACGCC
ATGAGAGCCAAGCATGCCAGCAAA---CAGGAGAGCAAC
>MN168362
GAGCTCCGTAGTCGGCGGTTCTGGAGTGCCACTTTGGCTGAGCTGCTCGGCACCCTGGTG
TTAGTGAGCGCTGTTCTGGGTGCCTCTGTTCCAGGGCCTGACCAGGCCCCCGTCGGACCC
CTATACCCAGCTGTAGCAGTGGGTGTCGTCATCGTTTCACTGGCACACTGCTTTGGAGAA
ATCAGTGGGGCGCAGGTGAATCCAGCTCTGACTCTCTCCTTGCTGGCCACCCGGAAGCTG
GATCTTCTCCATGCCCTCATTTACATGGTGGCACAGTGTTTGGGGGCCTGTTTGGGAGCC
TCTGCCCTCTACCTCGCTTTACCTCTCAAATCCACAGCAGACCATTTTGTTAACAGGGTC
CCCATAGAATTGAATGCAGCACAGGCTCTGTGGATCGAGGTTCTCTGCACTTTTCAGATG
GTCTTCACTGTCTTCTCTGTGGAGGAGCACAGACGGAAGGAGAGCGTAGAACCAGGAAAT
CTGGCCATTGGATTTGCTCATTCTGCTGGAGTGATGATAGGCGCCCGTTTCTCTGGTGCC
AGTATGAATCCAGCACGCTCCTTGGGTCCAGCTATCATCACTGGATTCTGGGAAAATCAC
TGGGTATTTTGGATTGGTCCAATCATCGGCGCTCTTCTAGCTGGAGTGTCCCACGAGTTC
TTCTTCGCCCGCAGTGCCTCTCGCCAGAAGCTGGTGGCCTGTTTGACCTGCAAGGACATT
GAAATCATTGAGACGGCCAGCATGACTGGGTCGTCTCTGTCCACAGTGACTCAGAACGCC
ATGAGAACCAAGCATGCCAGCAGA---CAGGAGAGCAAC

>MN168363
GAGCTTCGTAGTCGGCAGTTCTGGAGTGCCATGCTTGCAGAGCTGCTAGGCACCCTGCTG
TTAGTGAGCGCTGTTCTGGGCACCTCGGTTCCAGGTCCTGACGAGGCCCCCGTGGGGCCC
CTGTACCCAGCTGTAGCACTGGGTGCGGTCATCATCGCACTGGGACACTGTTTCGGAGAA
ATAAGTGGAGCACAGGTGAACCCTGCCGTGACTCTCTCTCTGCTGGCCACTCGGAAGCTG
GATGTTCTCCGGGCGCTTGTTTATGTCTCTGCTCAGTGTTTAGGGGCCTGTTTAGGGACC
CTGGCCCTCTACCTTGCCCTGCCTCTCAAAACCACCGCAGACCACTTTGTGAACAAGGTA
CCCATAGAGCTGAATGCAGCACAGGCTCTGGGCATTGAGATGTTGTGCACCTTCGAGATG
GTCTTCACTATCTTCTCAGTGGAGGAGCAGAGACGGAGGGAGAGCCCCGAACCTGGAAAT
CTGGCCATCGGACTGGCGCACACAGCCGGAGTGCTGATAGGGGCGCGGTTCTCTGGTGCC
AGCATGAATCCGGCCCGCTCTCTGGGTCCAGCCATCATCACTGGTTTCTGGGAAAACCAC
TGGGTGTACTGGATCGGACCGGTTCTTGGTGCTCTATTGGCCGGAGTCTCTCATGAGTTC
TTCTTTGCACGCAGCGCTTCTCGCCAGAAGCTGGTGGCCTGTTTGACCTGTAAGGACATT
GAGATTGTTGAAACAGCCAGCATGACCGGGTCGTCCCTCTCCACGGTGACGCAGAACGCC
ATGAAGGCCAAGCAGGCGAACAAA---CAGGACAGCAAC
>MN168364
GAGCTTCGTAGTCGGCAGTTCTGGAGTGCCATGCTTGCAGAGCTGCTAGGCACCCTGCTG
TTAGTGAGTGCTGTTCTGGGCACCTCGGTTCCAGGTCCTGACGAGGCCCCCGCGGGGCCC
CTGTACCCAGCTGTAGCACTGGGAGCGGTCATCATCGCACTGGGACACTGTTTCGGAGAA
ATAAGTGGAGCGCAGGTGAACCCTGCCGTGACTCTCTCTCTGCTGGCCACTCGGAAGCTG
GATGTTCTCCGGGCGTTTGTTTATGTCTCTGCTCAGTGTTTAGGGGCCTGTTTAGGGACC
CTGGCCCTCTACCTTGCCCTGCCTCTCAAAACCACCGCAGACCACTTTGTGAACAAGGTA
CCAATAGAGCTGAACGCAGCACAGGCTCTGGGCATTGAGATGTTGTGCACCTTCGAGATG
GTCGTCACCGTCTTCTCAGTGGAGGAGCAGAGACGGAGGGAGAGCCCCGAACCTGGAAAT
CTGGCCATCGGACTGGCGCACACAGCCGGAGTGCTGATAGGGGCGCGGTTCTCAGGTGCC
AGCATGAATCCGGCCCGCTCTCTGGGTCCAGCCATCGTCACTGGTTTCTGGGAAAACCAC
TGGGTGTACTGGATCGGACCGGTTCTTGGTGCTCTATTGGCCGGAGTCTCCCATGAGTTC
TTCTTTGCACGCAGCGCTTCTCGCCAGAAGCTGGTGGCCTGTTTGACCTGTAAGGACATT
GAGATTGTTGAAACAGCCAGCATGACCGGGTCGTCCCTCTCCACGGTAACGCAGAACGCC
ATGAAGGCCAAGCAGGCGAACAAA---CAGGACAACAAC
>MN168365
GAGCTTCGTAGTCGGCAGTTCTGGAGTGCCATGCTTGCAGAGCTGCTAGGCACCCTGCTG
TTAGTGAGCGCTGTTCTGGGCACCTCGGTTCCAGGTCCTGACGAGGCCCCCGTGGGTCCC
CTGTACCCAGCTGTAGCACTGGGTGCGGTCATCATCGCAGTGGGACACTGTTTCGGAGAA
ATAAGTGGAGCACAGGTGAACCCTGCCGTGACTCTCTCTCTGCTGGCCACTCGGAAGCTG
GATGTTCTCCGGGCGCTTGTTTATGTCTCTGCTCAGTGTTTAGGGGCCTGTTTAGGGACC
CTGGCCCTCTACCTTGCCCTGCCTCTCAAAACCACAGCAGACCACTTTGTGAACAAGGTA
CCCATAGAGCTGAACGCAGCACAGGCTCTGGGCATTGAGATGTTGTGCACCTTTGAGATG
GTCTTCACCATCTTCTCAGTGGAGGAGCAGAGACGGAGGGAGAGCCCCGAACCTGGACAT
CTGGCCATCGGACTGGCGCACACAGCCGGAGTGCTGATAGGGGCGCGGTTCTCTGGTGCC
AGCATGAATCCGGCCCGCTCTCTGGGTCCAGCCATCATCACTGGATTCTGGGAAAACCAC
TGGGTGTACTGGATCGGACCGGTTCTTGGTGCTCTATTGGCCGGAGTCTCTCATGAGTTC
TTCTTTGCACGCAGCGCTTCTCGCCAGAAGCTGGTGGCCTGTTTGACCTGTAAGGACATT
GAGATTGTTGAAACAGCCAGCATGACCGGGTCGTCCCTCTCTACGGTAACGCAGAACGCC
ATGAAGGCCAAGCAGGCGAACAAA---CAGGACAACAAC
>MN168366
GAGCTTCGTAGTCGGCAGTTCTGGACTGCCATGCTTGCAGAGCTGCTTGGCACCCTGCTG
TTAGTGAGTGCTGTTCTGGGCACCTCGGTTCCAGGGCCTGACGAGGCCCCCGTGGGACCC
CTGTACCCAGCTGTAGCACTGGGTGCGGTCATCATCGCACTGGGACACTGTTTCGGAGAA
ATAAGTGGAGCACAGGTGAATCCTGCTGTGACTCTTTCTCTGCTGGCCACCAGGAAGCTG
GATGTCCTCCGGGCCCTCGTGTATGTCTCTGCTCAGTGTCTAGGGGCCTGTTTAGGGGCC

TTGGCCCTCTACCTCGCCCTGCCTCTCAAAACCACTGCAGACCACTTTGTAAACAAGGTG
CCCATAGAGCTGAATGCAGCACAGGCTCTGGGCATTGAGGTGTTGTGCACCTTTGAGATG
GTCTTCACCATCTTCTCAGTGGAGGAGCAGAGACGCAGGGAGAGCCCCGAACCTGGAAAT
CTGGCCATCGGACTGGCGCACACAGCTGGAGTGCTGATAGGGGCACGGTTCTCCGGTGCC
AGCATGAATCCGGCCCGCTCTCTGGGTCCAGCCATCATCACTGGTTTCTGGGAAAACCAC
TGGGTGTACTGGATCGGACCGGTTCTTGGTGCTCTACTGGCAGGAGTCTCTCACGAGTTC
TTCTTTGCACGCAGCGCTTCTCGCCAGAAGCTGGTGGCCTGTTTGACCTGTAAGGACATT
GAGATTGTTGAGACAGCCAGCATGACTGGGTCGTCCCTCTCCACTGTAACGCAGAACGCC
ATGAGGGCCAAGCAGGCCAACAAA---CAGGACAACAAC
>MN168367
GAGCTTCGTAGTCGGCAGTTCTGGAGTGCCATGCTTGCAGAGCTGCTCGGCACCCTGCTG
TTAGTGACTGCTGTTCTGGGCGCCTCTGTTCCGGGCCCTGAGGAGATCCCTGGAGGGCCC
CTGTACCCGGCGGTAGCAGTGGGTGCGGTCATTGTTGCACTGGGACACTGTTTCGGAGAA
ATAAGCGGAGCACAGGTGAACCCTGCTGTGACTCTGTCTCTGTTGGCCACCCGGAGGCTG
GACGTTCTGCGGGCCCTCGTTTATGTCGCCGCTCAGTGTTTGGGGGCCTGTTTAGGAGCT
TCGGCCCTCTACCTCGCCCTGCCTGTTCGGACCACTGCAGACCACTTTGTGAACAGGGTG
CCCGCAGAGTTGAACGCAGGGCAGGCTCTGGGCGTTGAGGTTCTGTGCACCTTTCAGATG
GTCTTCACCGTCTTCTCAGTGGAGGACCAGCGGCGGAGGGAGAGCCCAGAACCAGGAAAT
CTGGCCATCGGGTTGGCGCACACTGCTGGAGTGATGATAGGGGCTCGGTTCTCTGGTGCC
AGCATGAACCCAGCCCGCTCTCTGGGTCCGGCCATCGTCACTGGGTTCTGGGAAAACCAC
TGGGTCTACTGGATCGGTCCAGTCCTCGGTGCCTTACTGGCCGGAGTCTCCCACGAGTTC
TTCTTTGCACGCAGCGCCTCTCGCCACAAGCTGGTGGCCTGCTTGACCTGTAAGGACATC
GACATTGTAGAGACGGCCAGCATGACCGGCTCGTCTCTGTCCACGGTCACGCAGAGCGCC
CTGAGGGCCAAGCAGGCCACCAAG---CAGGAGGGCAAC
>MN168368
GAGCTTCGCAGTCGGCAGTTCTGGAGTGCCATGCTTGCAGAGCTGCTCGGCACCCTGCTG
TTAGTGAGCGCTGTGCTGGGCGCCTCGCTCCCGGGCCCCGACGAGGCCCCGGCGGGCCCC
CTGTACCCAGCGGCAGCGGTGGGGGCGGTGATTGTTGCGCTGGGACACTGCTTCGGAGAA
ATAAGCGGAGCACAGGTGAACCCCGCTGTGACTCTGTCCTTCTTGGCCACGCGGAGGCTG
GACGTCCTCCGGGCCGTCGTTTACGTCACCGCTCAGTGTTTGGGGGCCTGTTTAGGAGCC
TCGGCCCTCTACCTGGCCCTGCCCGTCAAAACCACTGCAGACCACTTTGTGAACAGGGTG
CCCGTAGAGCTGAACGCGGGCCAGGCTCTGGGCGTGGAGGTTTTGTGCACCTTTCAGATG
GTCTTCACCGTCTTCTCAGTGGAGGACCAGCGGCGGAGGGAGAGTCCAGAACCAGGAAAC
CTGGCCATTGGGTTAGCACACACTGCTGGAGTGCTAATAGGGGCCCGGTTCTCTGGTGCC
AGCATGAACCCGGCCCGCTCTCTGGGTCCGGCCATCGTCACGGGGTTCTGGGAAAACCAC
TGGGTGTACTGGATCGGTCCGGTCCTCGGCGCCTTGCTGGCTGGAGTCTCCCACGAGTTC
TTCTTCGCTCGCAGCGCCTCTCGCCACAAGCTGGTGGCCTGCTTGACCTGCAAGGACATC
GACATCGTCGAGACGGCCAGCATGACCGGCTCGTCTCTGTCCACGGTCACGCAGAACGCC
ATGAGAGCCAAGCAGGCGAACAAA---CAGGAGGCCAAC
>MN168369
GAGCTGCGTAGTAGAAAGTTCTGGTGTGCTATTCTGGCAGAGCTGTTAGGTACCCTGGTG
CTGGTGAGCGCCGTGCTGGGCGCCTCTGTTCCGGGCCCTGAAGAAGCCGCCGTGGGACCC
CTGTATCCTGCAGCGGCAGTGGGCGTGGCCATCATTGCACTGAGTCACTGTTTTGGAGAA
ATGAGCGGAGCGCAAGTGAACCCTTCTCTGACTCTGTCTCTCTTGGCCACTCGGAGGCTG
GACGCTCTGCGGGCCTTGGTTTACATCGCCGCCCAATGTTTGGGGGCCTGTTTAGCCGCC
TGGATGCTGTACCTGGCCTTACCCCTCAAGACGACAGCGGAGCACTTCGTTAACAGGGTG
CCCATCGAGTTAAACGCAGCTCAGGCTTTGGGGATGGAGGTTCTGTGTACCTTCCAGATG
GTCTTCACTGTGTACTCGGTGGAGGACCAGCGACGGAGAGACAGCCCAGAACCAGGAAAC
CTGGCCATTGGAGCGGCCCACTCTGCTGGAGTTCTCATAGGGGCTCGCTTTTCTGGTGCC
AGTATGAACCCGGCACGCTCCTTGGGTCCAGCCATCGTCACGGGTTTCTGGGAGAACCAC
TGGATTTATTGGATTGGACCAGTGATGGGTGCGTTGCTGGCTGGAGTGTCCCATGAGTTC

TTTTTCGCACGCAGCGCCTCTCGTCAGAAGCTAGTGGCCTGCTTGACCTGCAAAGACATT
GAGATTGTTGAGACGGCCAGCGTGACCGGATCGTCGCTGTCCACGGTCACGCAGAACGCC
GCCAGAAGCAAACAGGCCAACAAA---CCGGAGAACAAC
>MN168370
GAGCTGCGTAGTAGAAAGTTCTGGAGTGCAATTCTGGCAGAGCTGTTAGGCACTCTGGTG
CTGGTGAGCGCCGTGCTGGGCGCCTCTGTTCCGGGCCCTGAGGAAGCCGCCGTGGGACCC
CTGTATCCCGCAGCGGCAGTGGGTGTGGCCATCGTTGCACTGTGTCACTGCTTTGGAGAA
ATGAGCGGAGCGCAAGTGAATCCTTCTCTGACTCTGTCTCTCTTGGCCACTCGGAGGCTG
GACGCTCTGCGGGCCTTGGTTTACATCGCCGCCCAATGTTTGGGGGCCTGTTTAGCCGCC
TGGATGCTGTACCTGGCCTTACCCCTCAAAACGACAGCGGAGCACTTCGTTAACAGGGTG
CCCATTGAGTTGAATGCAGCTCAGGCTTTGGGCGTGGAGGTTCTGTGTACCTTTCAGATG
GTCTTCACTGTGTACTCAGTGGAGGATCAGAGACGGAGAGACAGTCCAGAACCAGGAAAC
CTGGCTATTGGAGCGGCACACTCTGCTGGGGTTCTTATAGGGGCTCGGTTTTCTGGTGCC
AGTATGAACCCCGCACGCTCCTTGGGTCCAGCCATCGTCACGGGTTTCTGGGAGAATCAC
TGGATTTATTGGATTGGACCAGTGATGGGTGCGCTGCTGGCCGGAGTGTCCCATGAGTTC
TTTTTCGCACGCAGCGCCTCCCGTCAGAAGCTAGTGGCCTGCTTGACCTGCAAAGACATT
GAGATTGTTGAGACGGCCAGCGTGACCGGATCGTCGCTGTCCACGGTCACGCAGAACGCC
GCCAGAAGCAAACAGACCAACAAA---CCGGAGAACAAC
>MN168371
GACCTGCGTAGTAGAAAGTTCTGGTGTGCGATTCTAGCCGAGCTGCTAGGCACCCTGGTG
TTGGTGAGTGCCGTGCTGGGTGCCTCCGTTCCGGGCCCTGAAGAAGCCGCCGTGGGACCC
ATGTATCCTGCAGTGGCAGTGGGTGTGGCCATAATTGCACTGAGTCACTGTTTTGGAGAA
ATAAGCGGAGCACAAGTGAACCCTAGTCTGACTCTGTCTCTCTTGGCCACTCGGAGGCTG
GATGTTCTGCGGGCCCTGGTTTACATTGCTGCCCAGTGTTTTGGGGCCTGTTTAGCCGCC
TGGTTTCTGTACCTGGCCTTACCCTTCAAAACAACAGCGGAGCACTTCGTTAACAGGGTG
CCCATTGAGTTAAATGCAGCTCAGGCTTTGGGCATAGAGGTTTTGTGCACCTTTCAGATG
GTCTTCACTGTCTATTCAGTGGAGGATCAGAGACGGAGAGACAGCCCAGAACCAGGAAAC
TTGGCCATTGGAGTGGCACACTCTGCTGGAGTACTTATAGGGGCTCGGTTTTCTGGTGCC
AGCATGAACCCGGCGCGCTCCTTGGGTCCAGCCATCGTCACAGGTTTCTGGGAGAACCAC
TGGATTTATTGGATTGGACCAGTGATGGGTGCCTTGCTGGCTGGAGTGTCCCATGAGTTC
CTCTTTGCACGCAGCGCCTCTCGTCAGAAGCTAGTGGCCTGTTTGACCTGCAAAGATATT
GAGATTGTTGAGACGACCAGCATGACCGGGTCATCGCTGTCCACAGTCACGCAGAACGCC
GCCAGGAGCAAACAGGCCAACAAA---CCGGAGAACAAC
>MN168372
GAGCTACGTAGTCGGCAATTCTGGCACGCCATGCTGGCAGAGCTCCTCGGCACCCTGGTG
TTGGTGAGTGCTGTGCTGGGTGCCTCTGTGCCAGGCCCTGGAGATGCCCCTGGAGGACCC
CTGTATCCAGCGGTGGCAGTGGGTGTGGTGATTGTAGCACTGGGACACTGTTTTGGAGAA
ATAAGTGGGGCACAGGTGAACCCTGCTGTGACTCTGGCTCTGTTGGCCACACGGCGGCTT
GATGTTCTCCGGGCCCTCGTTTATATCATCGCTCAGTGTTTGGGGGCCTCCTTAGGAGCT
GGGGCCCTGTACCTGGCCCTGCCGTTTAAAACCACAGCAGAGCACTTTGTCAACAGGGTA
CCTTTAGAGCTGAATGCAGCCAAGGCTCTGGGCATTGAGGTCTTGTGCACCTTCCAGATG
GTCTTCACCGTCTTCTCGGTGGAGGACCAGCGAAGGAGGGAGAGCCCAGAACCAGGAAAC
CTGGCCATTGGACTAGCACACACTGCTGGTGTGCTGATAGGGGCTCGGTTCTCTGGCGCG
AGTATGAATCCCGCCCGCTCGCTGGGTCCGGCCATCATCACAGGCTTCTGGGAAGACCAC
TGGGTTTACTGGATCGGGCCGGTGCTCGGCGCCATACTGGCAGGGATGTCCCATGACTTC
TTCTTTGCACGCAGCGCCTCTCGTCAGAAGCTGGTGGCCTGTTTGACCTGTAAGGACATC
GAGATCGTGGAGACGGCAAGCATGACCGGATCGTCGCTGTCTACAGTCACGCAGAATGCC
ATGAGAGCCAAGCAGGCCAGCAAG---CAGGAAAACAAC
>MN168373
GAGCTACGTAGTCGGCAATTCTGGCACGCCATGCTGGCAGAGCTCCTCGGCACCCTGGTG
TTGGTGAGCGCTGTGCTGGGTGCCTCTGTGCCAGGCCCTGGAGATGCCCCTGGAGGACCC

CTGTATCCAGCGGTGGCAGTGGGTGTGGTGATTGTAGCACTGGGACACTGTTTTGGAGAA
ATAAGTGGGGCACAGGTGAACCCTGCTGTGACTCTGGCTCTGTTGGCCACACGGCGGCTT
GATGTTCTCCGGGCCCTCGTTTATATCATCGCTCAGTGTTTGGGGGCCTCCTTAGGAGCT
GGGGCCCTGTACCTGGCCTTGCCGTTTAAAACCACAGCAGAGCACTTTGTCAACAGGGTA
CCTTTAGAGCTGAATGCAGCCAAGGCTCTGGGCATTGAGGTCTTGTGCACCTTCCAGATG
GTCTTCACCGTCTTCTCGGTGGAGGACCAGCGAAGGAGGGAGAGCCCAGAACCAGGAAAC
CTGGCCATCGGACTAGCACACACTGCTGGTGTGCTGATAGGGGCTCGGTTCTCTGGCGCG
AGTATGAATCCCGCCCGCTCGCTGGGTCCGGCCATCATCACAGGCTTCTGGGAAGACCAC
TGGGTTTACTGGATCGGGCCGGTGCTCGGCGCCATACTGGCAGGGATGTCCCATGACTTC
TTCTTTGCACGCAGCGCCTCTCGTCAGAAGCTGGTGGCCTGTTTGACCTGTAAGGACATC
GAGATCGTGGAGACGGCAAGCATGACCGGATCGTCGCTGTCTACAGTCACGCAGAATGCC
ATGAGAGCCAAGCAGGCCAGCAAG---CAGGAAAACAAC
>MN168374
GAGCTACGTAGTCGGCAATTCTGGCACGCCATGCTGGCAGAGCTCCTCGGCACCCTGGTG
TTGGTGAGCGCTGTGCTGGGTGCCTCTGTGCCAGGCCCTGGAGATGCCCCTGGAGGACCC
CTGTATCCAGCGGTGGCAGTGGGTGTGGTGATTGTAGCACTGGGACACTGTTTTGGAGAA
ATAAGTGGGGCTCAGGTGAACCCTGCTGTGACTCTGGCTCTGTTGGCCACACGGCGGCTT
GATGTTCTCCGGGCCCTCGTTTATATCATCGCTCAGTGTTTGGGGGCCTCCTTAGGAGCT
GGGGCCCTGTACCTGGCCCTGCCGTTTAAAACCACAGCAGAGCACTTTGTCAACAGGGTA
CCTTTAGAGCTGAATGCAGCCAAGGCTCTGGGCATTGAGGTCTTGTGCACCTTCCAGATG
GTCTTCACCGTCTTCTCGGTGGAGGACCAGCGAAGGAGGGAGAGCCCAGAACCAGGAAAC
CTGGCCATTGGACTAGCACACACTGCTGGTGTGCTGATAGGGGCTCGGTTCTCTGGCGCG
AGTATGAATCCCGCCCGCTCGCTGGGTCCGGCCATCATCACAGGCTTCTGGGAAGACCAC
TGGGTTTACTGGATCGGGCCGGTGCTCGGCGCCATACTGGCAGGGATGTCCCATGACTTC
TTCTTTGCACGCAGCGCCTCTCGTCAGAAGCTGGTGGCCTGTTTGACCTGTAAGGACATC
GAGATCGTGGAGACGGCAAGCATGACCGGATCGTCGCTGTCTACAGTCACGCAGAATGCC
ATGAGAGCCAAGCAGGCCAGCAAG---CAGGAAAACAAC
>MN168375
GAGCTACGTAGTCGGCAATTCTGGCATGCCATGCTGGCAGAGCTCCTCGGCACCCTGGTG
TTGGTGAGCGCTGTGCTGGGTGCCTCTGTGCCGGGCCCTGGAGATGCCTCTGGAGGACCC
CTGTATCCAGCGGTGGCAGTGGGTGTGGTGATTGTAGCACTGGGACACTGTTTTGGAGAA
ATAAGTGGGGCACAGGTGAACCCTGCTGTGACTCTGGCTCTGTTGGCCACACGGCGGCTT
GAGGTTCTCCGGGCCCTCGTTTATATCATCGCTCAGTGTTTGGGGGCCTCCTTAGGAGCT
GGGGCCCTGTACCTGGCCCTGCCGTTTAAAACCACAGCAGAGCACTTTGTCAACAGGGTA
CCTTTAGAGCTGAATGCAGCCAAGGCTCTGGGCATCGAGGTCTTGTGCACCTTCCAGATG
GTCTTCACCATCTTCTCAGTGGAGGACCAGCGAAGAAGGGAGAGCCCAGAACCAGGAAAC
CTGGCCATTGGACTAGCACACACTGCTGGTGTGCTGATAGGGGCTCGGTTCTCTGGCGCG
AGTATGAATCCTGCCCGCTCGCTGGGTCCAGCCATCATCACAGGCTTCTGGGAAGACCAC
TGGGTTTACTGGATCGGGCCGGTGCTCGGCGCCATACTGGCAGGGATGTCCCATGACTTC
TTCTTTGCACGCAGCGCCTCTCGTCAGAAGCTGGTGGCCTGTTTGACCTGTAAGGACATC
GAGATCGTGGAGACGACAAGCATGACCGGGTCGTCGCTGTCTACAGTCACGCAGAATGCC
ATGAGAGCCAAGCAGGCCAGCAAG---CAGGAAAACAAC
>MN168376
GAGCTACGTAGTCGGCAATTCTGGCACGCCATGCTGGCAGAGCTCCTCGGCACCCTGGTG
TTGGTGAGCGCTGTGCTGGGTGCCTCTGTGCCAGGCCCTGGAGATGCCCCTGGAGGACCC
CTGTATCCAGCGGTGGCAGTGGGTGTGGTGATTGTAGCACTGGGACACTGTTTTGGAGAA
ATAAGTGGGGCACAGGTGAACCCTGCTGTGACTCTGGCTCTGTTGGCCACACGGCGGCTG
GATGTTCTCCGGGCCCTCGTTTATATCATCGCTCAGTGTTTGGGGGCCTCCTTAGGAGCT
GGGGCCCTGTACCTGGCCCTGCCGTTTAAAACCACAGCAGAGCACTTTGTCAACAGGGTA
CCTTTAGAGCTGAATGCAGCCAAGGCTCTGGGCATCGAGGTCTTGTGCACCTTCCAGATG
GTCTTCACCGTCTTCTCAGTAGAGGACCAGCGAAGGAGGGAAAGCCCAGAACCAGGAAAC

CTGGCCATTGGACTAGCACACACTGCTGGTGTGCTGATAGGGGCTCGGTTCTCTGGCGCG
AGTATGAATCCCGCCCGCTCGCTGGGTCCAGCCATCATCACAGGCTTCTGGGAAGACCAC
TGGGTTTACTGGATCGGGCCGGTGCTCGGGGCTATACTGGCAGGGATGTCCCATGAGTTC
TTCTTTGCACGCAGTGCCTCTCGTCAGAAGCTGGTGGCCTGTTTGACCTGCAAGGACATC
GAGATCGTGGAGACGGCAAGCATGACCGGATCGTCGCTGTCTACAGTCACACAGAACGCC
ATGAGAGCCAAGCAGGCCAGCAAG---CAGGAAAACAAC
>MN168377
GAGCTACGTAGTCGGCAGTTCTGGCGTGCCATGGTGGCAGAGCTACTTGGCACCCTGGTG
TTAGTGAGCGCTGTGCTGGGTGCCTCTGTGCCAGGCCCTGGAGAGGCCCCTGGAGGACCC
CTCTATCCAGCGGTGGCAGTGGGTGTGGTGATTGTAGCACTGGGACACTGTTTTGGAGAA
ATAAGTGGGGCACAGGTGAACCCAGCTTTGACTCTGGCTCTGTTGGCCACACGGCGGCTG
GATGTTCTCCGGGCCCTTGTTTATATCATTGCTCAGTGTTTGGGGGCCTCCTTAGGAGCC
GGGGCCCTGTACTTGGCCCTGCCTTTTAAAACCACTGCAGACTACTTTGTCAACAGGGTA
CCTTTAGAGTTAAATGCAGCCAAGGCTCTGTGCATTGAGGTTTTGTGCACCTTCCAGATG
GTCTTCACTGTCTTCTCAGTGGAGGACCAGCGAAGGAGGGAGAGCCCAGAACCAGGAAAC
CTGGCCATTGGATTAGCACATACTGCTGGTGTACTGATAGGGGCGCGGTTCTCTGGTGCG
AGTATGAATCCTGCCCGCTCTTTTGGTCCAGCCATCATCACTGGTTTCTGGGAAGATCAC
TGGGTGTACTGGATCGGGCCAGTGCTCGGTGCCATACTGGCGGGGGTCTCCCATGAGTTC
TTCTTTGCACGCAGCGCCTCTCGTCAGAAGCTGGTGGCCTGTTTGACCTGTAAGGACATC
GAGATCGTGGAGACGGCAAGCATGACCGGATCATCGCTGTCCACAGTTACCCAGAATGCC
ACAAGAGCCAAGCAGGCCAACAAA---CAGGAGAACAAC
>MN168378
GAGCTGCGTAGTCGGCAGTTCTGGCGTGCCATGGTGGCAGAGCTACTTGGCACCCTGGTG
TTAGTGAGCGCTGTGCTGGGTGCCTCTGTGCCAGGCCCTGGAGAGGCCCCTGGAGGACCC
CTCTATCCAGCGGTGGCAGTGGGTGTGGTGATTGTAGCACTGGGACACTGTTTTGGAGAA
ATAAGTGGGGCACAGGTGAACCCAGCTTTGACTCTGGCTCTGTTGGCCACACGGCGGCTG
GATGTTCTCCGGGCCCTTGTTTATATCATTGCTCAGTGTTTGGGGGCCTCCTTAGGAGCC
GGGGCCCTGTACTTGGCCCTGCCTTTTAAAACCACTGCAGACTACTTTGTCAACAGGGTA
CCTTTAGAGTTAAATGCAGCCAAGGCTCTGTGCATTGAGGTTTTGTGCACCTTCCAGATG
GTCTTCACTGTCTTCTCAGTGGAGGACCAGCGAAGGAGGGAGAGCCCAGAACCAGGAAAC
CTGGCCATTGGATTAGCACATACTGCTGGTGTACTGATAGGGGCGCGGTTCTCTGGTGCG
AGTATGAATCCTGCCCGCTCTTTTGGTCCAGCCATCATCACTGGTTTCTGGGAAGATCAC
TGGGTGTACTGGATCGGGCCAGTGCTCGGTGCCATACTGGCGGGGGTCTCCCATGAGTTC
TTCTTTGCACGCAGCGCCTCTCGTCAGAAGCTGGTGGCCTGTTTGACCTGTAAGGACATC
GAGATCGTGGAGACGGCAAGCATGACCGGATCATCGCTGTCCACAGTTACCCAGAATGCC
ACAAGAGCCAAGCAGGCCAACAAA---CAGGAGAACAAC
>MN168379
GAGCTTCGTAGTCGGCAGTTCTGGCGTGCCATGCTCGCAGAGCTGCTCGGCACCCTGGTG
TTAGTGAGCTGCGTGCTGGGTTCCTCCATGCCAGGACCTGGAGAGGCCCCGGGGGGGCCC
CTGTATCCAGCAGTGGCTGTGGGCGTGGTGATTGTCGCACTGGGACACTGTTTTGGAGAA
ATAAGCGGGGCACAGGTGAACCCTGCTGTGACTCTGTCTCTGTTGGCCACGCGGAGGCTG
GATGTTCTCCGGGCCCTTCTTTACATTGCTATTCAGTGTCTGGGGGCCTCTTTAGCAGCC
GGGGCCTTGTACCTGGCCCTGCCCATCAAAACCACTGCAGACCACTTTGTCAACAAGGTG
CCGTTAGATTTGAATGCAGGCCAGGCTCTGGGCATTGAGATTTTGTGCACCTTCCAGATG
GTCTTCACCATCTTCTCAGTGGAGGACCAGCGTCGGAGGGAGTGTACAGAACCCGGAAAC
CTGGCCATTGGATTAGCACACACTGCTGGAGTGCTAATAGGGGCACGGTTCTCTGGTGCA
TGTATGAATCCTGCACGTGCTCTGGGTCCAGCCATCATCACGGGTTTCTGGGAAAACCAC
TGGGTTTACTGGCTCGGACCGGTTCTTGGAGGTATCCTGGCCGCGGTATCCCATGACTTC
TTCTTTGCACGCAGCGCCTCTCGTCAGAAGTTGGTGGCTTGTTTGACCTGTAAGGACATC
GAGATCGTGGAGACAGCCAGCATGACGGGGTCCTCGCTGTCCACAGTCACACAAAACGCC
ACGAGGGCCAAGCAGGCCAACAAG---CAGGAGAACAAT

>MN168380
GAGCTCCGGAGTCGGCAGTTCTGGCGTGCCATGATGGCAGAGCTGCTCGGCACCCTGGTG
TTAGTGAGCGCTGTGCTGGGTGCCTCGGTGCCAGGCCCTGGAGAGGCCCCCGGGGGACCC
CTGTACCCAGCCGTGGCAGTAGGTGTGGCGATTGTCGCAATGGCACACTGTTTTGGGGAA
ATAAGCGGAGCTCAGGTGAACCCTGCTTTGACTCTGTCTTTCATGACCACACGGAGGCTG
GATGTTCTCCGGGGCCTCGTTTATATCACTGCCCAATGTTTAGGGGCCTCTTTAGGAGCC
GGGGCCCTTTATTTGGCTCTGCCAATCAAAACCACTGCAGACCATTTTGTCAACAAGGTG
CCCATAGAGTTAAATGCAGCTCAGGCTCTCGGTATCGAGGTTCTGTGCACCTTCCAGATG
GTCTTTACTGTCTTCTCAGTGGAGGACCAGCGGCGGAGGGAGAGTCCAGAACCAGGAAAC
CTGGCCATTGGATTAGCACATAGTGCTGGAGTGCTCATAGGGGCTCGGTTCTCTGGTGCG
AGTATGAATCCTGCACGATCTCTGGGTCCAGCCATCATCACTGGCTTCTGGGAAAACCAC
TGGGTTTATTGGATCGGACCGGTGCTCGGTGCTGTGCTGGCCGGAGTGTCCCACGAATTC
TTCTTTGCACGCAGCGCCTCTCGTCAGAAGCTGGTGGCCTGTTTGACCTGTAAAGACATT
GAAATCGTAGAGACGACCAGCATGACGGGATCGTCTCTGTCCACGGTCACGCAGAACGCA
---CGAGTGAAGCAGGCCAACAAA---CAGGAGAACAAC
>MN168381
GAGCTCCGGAGTCGGCAGTTCTGGCGTGCCATGATGGCAGAGCTGCTCGGCACCCTGGTG
TTAGTGAGCGCTGTGCTGGGTGCCTCGGTGCCAGGCCCTGGAGAGGCCCCCGGGGGACCC
CTGTACCCAGCCATGGCAGTAGGTGTGGCAATTGTCGCAATGGCACACTGTTTTGGGGAA
ATAAGCGGAGCTCAGGTGAACCCTGCTTTGACTCTGTCTTTCATGACCACACGGAGGCTG
GATGTTCTCCGGGGCCTCGTTTATATCACTGCCCAGTGTTTAGGGGCCTCTTTAGGAGCC
GGGGCCCTTTATTTGGCTCTGCCAGTCAAAACCACTGCAGACCATTTTGTCAACAAGGTG
CCCATAGAGTTAAATGCAGCTCAGGCTCTCGGTATCGAGGTTCTGTGCACCTTCCAGATG
GTCTTTACTGTCTTCTCAGTGGAGGACCAGCGGCGGAGGGAGAGTCCAGAACCAGGAAAC
CTGGCCATTGGATTAGCACATAGTGCTGGAGTGCTCATAGGGGCTCGGTTCTCTGGTGCG
AGTATGAATCCTGCACGATCTCTGGGTCCAGCCATCATCACTGGCTTCTGGGAAAACCAC
TGGGTATATTGGATCGGACCGGTGCTCGGTGCTGTGCTGGCCGGAGTGTCCCACGAATTC
TTCTTTGCACGCAGCGCCTCTCGTCAGAAGCTGGTGGCCTGTTTGACCTGTAAAGACATT
GAAATCGTAGAGACGACCAGCATGACGGGATCGTCTCTGTCCACGGTCACGCAGAACGCA
---CGAGTGAAGCAGGCCAACAAA---CAGGAGAACAAC
>MN168382
GAGCTACGTAGTCGGCAGTTCTGGCGTGCCATGATGGCAGAGCTGCTCGGCACCCTGGTG
TTAGTGAGCGCTGTGCTGGGTGCCTCAGTGCCAGGCCCTGGAGAGGCCCCCGGGGGACCC
CTGTACCCAGCTGTGGCAGTAGGTGTGGCGATTGTCGCAATGGCACACTGTTTTGGGGAA
ATAAGCGGAGCTCAGGTGAACCCTGCTTTGACTCTGTCTTTCATGACCACACGGAGGCTG
GATGTTCTCCGGGGCCTTGTTTATATCACTGCCCAGTGTTTGGGGGCCTCTTTAGGAGCC
GGGGCCCTTTATTTGGCTCTGCCAGTCAAAACCACTGCAGACCATTTTGTCAACAAGGTG
CCCATAGAGTTGAATGCAGCTCAGGCTCTCGGTATCGAGGTTCTGTGCACCTTCCAGATG
GTCTTTACTGTCTTCTCAGTGGAGGACCAGCGGCGGAGGGAGAGTCCAGAACCAGGAAAC
CTGGCCATTGGATTAGCACATAGTGCTGGAGTGCTCATAGGGGCTCGGTTCTCTGGTGCG
AGTATGAATCCTGCGCGATCTCTGGGTCCAGCCATCATCACTGGCTTCTGGGAAAACCAC
TGGGTTTATTGGATCGGACCGGTGCTCGGTGCCGTGCTGGCCGGAGTGTCCCACGAATTC
TTCTTTGCACGCAGTGCCTCTCGTCAGAAGCTGGTGGCCTGTTTGACCTGTAAAGACATT
GAAATCGTAGAGACGACCAGCATGACGGGATCGTCTCTGTCCACGGTCACGCAAAATGCA
---CGAGTGAAGCAGGCCAACAAA---CAGGAGAACAAC
>MN168383
GAGCTACGTAGTCGGCAGTTCTGGCGTGCCATGATGGCAGAGCTGCTCGGCACCCTGGTG
TTAGTGAGCGCTGTGCTGGGTGCCTCGGTGCCAGGCCCTGGAGAAGCCCCCGGGGGACCC
CTGTACCCAGCTGTGGCAGTAGGTGTGGCGATTGTCGCAATGGCACACTGTTTTGGGGAA
ATAAGCGGAGCTCAGGTGAATCCTGCTTTGACTTTGTCCTTCATGACCACACGGAGGCTG
GATGTTCTCCGGGGCCTCGTTTATATCACTGCTCAGTGTTTGGGGGCCTCTTTAGGAGCC

GGGGCCCTTTATCTGGCTCTGCCAGTCAAAACCACTGCAGACCATTTTGTCAACAAGGTG
CCCATAGAGTTACATGCAGCTCAGGCTCTCGGTATCGAGGTTCTGTGCACCTTCCAGATG
GTCTTTACTGTCTTCTCCGTGGAGGACCAGCGGCGGAGGGAGAGTCCAGAACCAGGAAAC
CTGGCCATTGGATTAGCACATAGTGCTGGAGTGCTCATAGGGGCTCGGTTCTCTGGTGCG
AGTATGAATCCTGCCCGATCTCTGGGTCCAGCCATCATCACTGGCTTCTGGGAAAACCAC
TGGGTATATTGGATTGGACCGGTGCTCGGTGCCGTACTGGCCGGAGTGTCCCACGAATTC
TTCTTTGCACACAGCGCCTCCCGTCAGAAGCTGGTGGCCTGTTTGACCTGTAAAGACATT
GAGATCGTAGAGACGACCAGCATGACGGGGTCGTCTCTGTCCACGGTGACGCAGAACGCA
---CGAGTGAAGCAGGCCAACAAA---CAGGAGAACAAC
>MN168384
GAGCTACGTAGTCGGCAGTTCTGGCGTGCCATGATGGCAGAGCTGCTCGGCACCCTGGTG
TTAGTGAGCGCTGTGCTGGGTGCCTCGGTGCCAGGCCCTGGAGAGGCCCCCGGGGGACCC
CTGTACCCAGCTGTGGCAGTGGGTGTGGCGATTGTGGCAATAGCGCACTGTTTTGGAGAA
ATAAGCGGAGCACAGGTGAACCCTGCTTTGACTCTGTCTTTCATGACCACACGGAGGCTG
GATGTTCTTCGGGGCCTTGTTTATATGACTGCTCAGTGTTTGGGGGCCTCTTTAGGAGCC
GGAGCCCTCTACCTGGCTCTGCCTGTCAAAACCACTGCAGACCATTTTGTCAACAAGGTG
CCCATTGAGTTAAATGCAGCTCAGGCTCTTGGCATTGAGGTTCTGTGCACCTTCCAGTTG
GTCTTCACTGTCTTCTCAGTGGAGGACCAGCGACGGAGGGAGAGTCCAGAACCAGGAAAC
CTGGCCATTGGATTAGCACACAGCGCTGGAGTGCTAATAGGGGCTCGGTTCTCAGGTGCG
AGTATGAATCCTGCGCGCTCTCTGGGTCCAGCCATCATCACCGGCTTCTGGGAAAACCAC
TGGGTTTACTGGATCGGACCGGTGCTCGGTGCTGTTCTGGCCGGAGTGTCCCATGAGTTC
TTCTTTGCACGCAGCGCCTCTCGTCAGAAGCTGGTGGCCTGTTTGACCTGTAAAGACATT
GAGATCGTAGAGACGACCAGCATGACGGGATCATCTCTGTCCACCGTCACACAGAACGCA
---AGAGCCAAGCAGGCCAACAAA---CAGGAGAACAAC
>MN168385
GAGCTTCGTAGTCGGCAGTTCTGGCGTGCCATGCTGGCAGAGCTGCTTGGCACCCTGGTG
TTAGTGAGCGCCGTGCTGGGTGCCTCCTTGCCAGGCCCGGGAGAGGCCCCTGGGGGGCCC
CTGTACCCAGCTGTGGCCGTGGGTGTGGTAATCGTTGCACTAGGACACTGTTTTGGAGAG
ATAAGCGGGGCACAGGTCAACCCTGCTGTGACTCTGTCTCTGTTGGCCACGAGGAAAGTG
GATGTTTTGCGGGCTCTTGTTTTTATCGCTGCTCAGTGTTTGGGAGCCTCGTTGGGAGCC
GGGGCGCTCTACCTGGCCCTGCCGGTCAGGACTTTGGCCGACCACTTTGTCAACAAGGTG
CCCATAGATTTGAATGCAGGTCAGGCTCTGGGTATCGAGGTTCTGTGCACCTTCCAGATG
GTCTTCACCGTCTTCTCAGTGGACGACCAGCGGCGAAGGGAAAGCACAGAGCCAGGAAAT
CTGGCCATTGGATTAGCACACACTGCAGGAGTGATGATAGGGGCCCGGTTTTCTGGTGCG
AGTATGAATCCTGCACGAACTCTCGGTCCTGCGATCATCACCGGCTTCTGGGAAAATCAC
TGGGTTTACTGGATCGGACCGGTGTTTGGCGCCGTGCTGGCCGGAGTGTCCCACGAGTTC
TTCTTTGGCCGCAGCGCCTCTCGACAGAAGCTGGTGGCCTGTTTGACCTGCAAAGACATC
GAGATCGTGGAGACGGCCAGCATGACCGGGTCATCTCTATCCACGGTGACGCAGAGCGCC
ATGAGAGCCAAACAGGCCAACAAG---CAAGAGTCCAAC
>MN168386
GAGCTTCGTAGTCGGCAGTTCTGGCGTGCCATGCTGGCAGAGCTGCTTGGCACCCTGGTG
TTAGTGAGCGCCGTGCTGGGTGCCTCCTTGCCAGGCCCGGGAGAGGCCCCTGGGGGGCCC
CTGTACCCAGCTGTGGCCGTGGGTGTGGTGATCGTTGCATTGGGACACTGTTTTGGAGAG
ATAAGCGGGGCACAGGTCAACCCTGCTGTGACTCTGTCTCTGTTGGCCACGAGGAAAGTG
GATGTTTTGCGGGCTCTTGTTTTTATCGCTGCTCAGTGTTTGGGAGCCTCGCTGGGAGCC
GGGGCGCTCTACCTGGCCCTGCCAGTCAGGACTTTGGCGGACCACTTTGTCAACAAGGTG
CCCATTGAGTTGAATGCAGGTCAGGCTCTGGGTATCGAGGTTCTGTGCACTTTCCAGATG
GTCTTTACCATCTTCTCAGTGGATGACCAGCGGCGAAGGGAAAGCACAGAGCCAGGAAAT
CTGGCCATTGGATTAGCACACACTGCTGGAGTGTTGATTGGGGCCCGGTTTTCTGGTGCG
AGTATGAATCCTGCACGAACTCTCGGTCCAGCGATCATCACCGGCTTCTGGGAAAATCAC
TGGGTGTACTGGATGGGACCGGTGTTCGGTGCCATGCTGGCCGGAGTTTCCCACGAGTTC

TTCTTTGCCCGCAGCGCCTCTCGACAGAAGCTGGTGGCCTGCTTGACCTGCAAAGACATC
GAGATCGTGGAGACAGCCAGCATGACCGGCTCGTCTCTATCCACGGTGACGCAGAGCGCC
ATGAGAGCCAAACAGGCCAACAAG---CAGGATACAAAC
>MN168387
GAGCTTCGCAGTCGGCAGTTCTGGCGTGCCATGCTGGCAGAGCTGCTCGGCACCCTGGTG
TTAGTGAGCGCCGTGCTGGGTGCCTCTCTGCCGGGCCCAGGAGAGGCCCCTGGGGGGCCC
CTGTACCCAGCGGTGGCAGTGGGTGTGGCGGTCGTTGCACTGGGACACTGTTTTGGAGAA
ATAAGCGGGGCACAGGTCAACCCCGCTGTGACTCTGTCTCTGTTGGCCACGAGGAAGGTG
GATGTGTTGCGGGCGCTGGTTTTAATGGCGGCTCAGTGTTTGGGAGCCTCTTTAGGGGCG
GGGGCGCTCTACCTGGCCCTGCCGGTCAAGACGCTGGCGGACCACTTTGTCAACAAGGTG
CCCATAGAGCTGAACGCAGCTCAGGCTCTGGGCATCGAGGTTCTGTGCACCTTCCAGATG
GTCTTCACCATCTTCTCAGTGGACGACCAGCGGCGAAGGGACAGCCCAGAGCCGGGAAAC
CTGGCCATCGGATTAGCACACACTGCTGGAGTGTTAATAGGGGCCCGGTTTTCTGGAGCG
AGTATGAATCCTGCTCGATCTCTGGGTCCTGCAATCATCACCGGCTTTTGGGAAAATCAC
TGGGTGTACTGGATCGGACCGGTGCTCGGGGCCGTGCTGGCTGGCGTGTCCCACGAGTTC
TTCTTCGCTCGCAGCGCCTCCCGACAGAAGCTGGTGGCCTGCCTGACCTGCAAGGACATC
GAGATCGTGGAGACGGCCAGCATGACCGGATCGTCTCTGTCCACCGTCACGCAGAGCGCC
ATGAGGGCCAAACAGGCCAACAAA---CAGGAGAACAAC
>MN168388
GAGCTGAGGACTCGGCAGTTCTGGCGTGCCATCCTGGCAGAGCTGCTGGGCACCCTGGTG
TTAGTGAGCACCGTGCTGGGTGCCTCTGTGCCAGGCACCGGAGAGGCCCCCGGGGGGCCC
CTGTACCCAGCAGTGGCCGTGGGTGTGGTGATTGTTGCACTGGGACACTGTTTTGGAGAA
ATAAGTGGGGCACAGGTGAATCCAGCTGTTACTCTGTCTCTTTTGGCCACTCGTAAAATG
GATGTGCTGCGAGCCTTTGTTTATATCACTGCTCAGTGTTTGGGGGCCACTTTAGCAGCT
GGGGCCCTTTTCCTGGCCCTGCCACTAAAACCCACTGCAGAACATTTCATCAACAAGGTT
CCTATGGAGGTAAATGCAGCTCAGGCTCTGGGCATTGAGGTTCTGTGCACCTTTCAGATG
GTTTTCACCATCTTCTCAGTGGATGAACAGCGACGAAGGGATAGTCCAGAGCCAGGAAAC
CTAGCCATTGGGTTGGCACACACTGCTGGAGTGCTGTTAGGGGCTCGGTTTTCAGGTGCA
TGTATGAACCCTGGGCGTGCTCTGGGTCCAGCTATCATCATTGGCTTCTGGGAAAACCAT
TGGGTTTATTGGCTGGGGCCGGTGCTTGGGGCCGTCCTGGCCGGGGTGTCCCACGAGTTC
TTCTTTGCTCGCAGTGCCTCTCGTCAGAAGCTGGTGGCCTGTTTGACCTGCAGGGACATT
GAGATTGTGGAGACGGCGAGCATGACGGGATCATCCTTGTCCACGGTCACGCAGAGCGCC
ATGAGGGCGAAGCAGGCCAACAAA---CAGGAGAACAAC
>MN168389
GAGCTACGGAGTCGGCAGTTTTGGCGAGCCATCCTGGCAGAGCTGCTCGGCACCCTGGTG
TTAGTGAGTGCCGTGCTGGGCGCCTCTGTGCCGGGCCCAGGAGAGCTCCCTGGGGGACCC
CTGTACCCAGCTGTGGCAGTGGGGGTGGTGATTGTTGCACTGGCCCACTGTTTTGGAGAA
ATAAGCGGAGCACAGGTGAACCCGGCGGTGACTCTGTCTCTGTTGGCCACTCGGAGGGTG
GACGTCCACAGGGCCCTTTTCTACATTGCTGTGCAGTGTCTGGGGGCCTCTCTGGGGGCC
GGGGCCCTCTACCTGGCCCTGCCCATGAAAACCACGGCGGAACACTTCGTCAACAGGGTC
CCTCTGGAGATGAACGCAGGCCAGGCGCTGGGTATTGAGGTTCTGTGCACCTTCCAGATG
GTCTTCACTATTTTCTCAGTTGAGGACCAGCGGCGGAGGGAGAGTCCAGAACCAGGGAAC
TTAGCCATCGGATTGGCCCACACAGCCGGAGTGTTAATAGGGGTACGGTTCTCTGGTGCG
TGTATGAATCCTGCTCGTGCTCTGGGTCCAGCCATCATCGTCGGATTCTGGGAAAATCAC
TGGGTGTACTGGATCGGCCCGGTGCTCGGTGCTGTGCTGGGCGGAGTGTCCCACGAGTTC
CTCTTCGCCCGCAGCGCCTCTCGTCAGAAGCTGGTGGCGTGTCTGACCTGTAAGGACATC
GACATCGTGGAGACGGCCAGTATGACCGGATCGTCCCTGTCCACGGTCACGCAGAACGCC
ATGAGAGCCAAGCAGGCCAACAAA---CAAGACGGCAAC
>MN168390
GAGCTACGGAGTCGGCAGTTTTGGCGAGCCATTCTGGCAGAGCTGCTCGGCACCCTGGTG
TTAGTGAGTGCCGTGCTGGGCGCCTCTGTGCCGGGCCCAGGAGAGCTCCCTGGGGGACCC

CTGTACCCAGCTGTGGCAGTGGGTGTGGTGATTGTTTCACTGGGCCACTGTTTTGGAGAA
ATAAGTGGAGCTCAGGTGAACCCGGCAGTGACTCTGTCTCTGTTGGCCACTCGGAGGGTG
GACGTCCACAGGGCCCTTTTCTACATCGCTGTGCAGTGTCTGGGGGCCTCTCTGGGGGCC
GGGGCCCTCTACCTGGCCCTGCCGATGAAAACCACGGCGGAACACTTCGTCAACAGGGTG
CCTCTGGAGATGAACGCAGGCCAGGCGCTGGGTATTGAGGTTCTGTGCACCTTCCAGATG
GTCTTCACTATTTTCTCAGTGGAGGACCAGCGGCGGAGGGAGAGTCCAGAACCAGGGAAC
TTAGCCATCGGATTGGCCCACACAGCTGGAGTGTTAATAGGGGGACGGTTCTCTGGTGCG
TGTATGAATCCTGCTCGTGCTCTGGGTCCAGCCATCATCGTCGGGTTCTGGGAAAATCAC
TGGGTTTACTGGATCGGCCCGGTGCTCGGTGCTGTGCTGGGCGGAGTGTCCCACGAGTTC
CTCTTCGCACGCAGTGCCTCTCGCCAGAAGCTGGTGGCGTGTCTGACCTGTAAGGACATC
GACATCGTGGAGACAGCCAGTATGACCGGATCGTCCCTGTCCACGGTCACGCAGAACGCC
ATGAGAGCCAAGCAGGCCAACAAA---CAAGACGGCAAC
>MN168391
GAGCTACGGAGTCGGCAGTTCTGGCGAGCCATGCTGGCAGAGCTGCTCGGCACCCTGGTG
TTAGTGAGTGCCGTGCTGGGCGCCTCTGTGCCGGGCCCAGGAGAGGCCCCTGGGGGACCC
CTGTACCCAGCTGTGGCCGTGGGTGTGGTGATTGTTGCACTGGGACACTGTTTTGGAGAA
ATAAGTGGTGCACAGGTGAACCCGGCAGTGACTCTGTCTCTGTTGGCCACTCGGAGGGTG
GACGTCCTCAGGGCCCTTTTCTACATCGCAGTGCAGTGTCTGGGGGCCTCTCTGGGGGCC
GGGGCCCTCTACCTGGCCCTGCCGCTGAAAACCACCGCGGAACACTTTGTCAACAGGGTG
CCTCTGGAGTTGACCGCAGCCCAGGCGCTGGGTATTGAGGTTCTGTGCACCTTCCAGATG
GTCTTCACTATTTTCTCAGTGGAGGACCAGCGGCGGAGGGAGAGTCCAGAACCAGGAAAC
TTAGCCATCGGATTAGCTCACACAGCTGGAGTGTTAATAGGGGCCCGGTTCTCTGGTGCG
TGTATGAATCCGGCTCGTGCTCTGGGTCCAGCCATCATCGTCGGGTTCTGGGAAAATCAC
TGGGTTTACTGGATAGGCCCGGTGCTCGGTGCTTTGCTAGGTGGAGTGTCCCATGAGTTC
CTCTTCGCACGCAGCGCCTCTCGCCAGAAGCTGGTGGCATGTCTGACCTGTAAGGACATC
GAGATCGTGGAGACAGCCAGTATGACTGGATCATCCTTGTCCACGGTCACACAGAACGCC
ATGAGAGCCAAGCAGGCCAACAAA---CAAGACAACAAT
>MN168392
GAGCTGCGGAGTCGGCAGTTCTGGCGAGCCGTGCTGGCAGAGCTGCTCGGCACCCTGGTG
TTAGTGAGCGCCGTGCTGGGTGCGTCCGTGCCAGGGCCCGGAGAGGCCCCCGGGGGCCCC
CTGTACCCAGCGGTGGCGCTGGGCGCGGTGATCGTTGCGCTGGGACACTGTTTTGGAGAA
ATCAGCGGCGCGCAGGTGAACCCGGCGGTGACTCTGTCCCTGTTGGCCACTCGGAGGCTG
GACGTCCTCAGGGCCGTTCTCTACATCGCTGTTCAGTGTCTGGGGGCCTCTCTGGGAGCC
GGGGCCCTTTACCTGGCCCTGCCCCTGAAGACCACCGCCGACCACTTCGTCAACAAGGTG
CCGATGGAGCTGAACGCGGCTCAGGCGCTGGGCGTCGAGCTGCTCTGCACCTTCCAGATG
GTTTTCACCATCTTCTCGGTGGAGGACCAGCGACGGAGGGAGAGTCCCGAACCGGGGAAC
TTGGCTATTGGACTGGCGCACACAGCTGGAGTGATGATCGGGGTA---------------

---GTTTACTGGATCGGCCCGGTGCTCGGCGCCGTGCTGGCCGGAGTGTCGCACGAGTTC
TTCTTCGCGCGCGGCGCCTCGCGCCACAAGCTGCTGGCGTGTCTGACGTGCCGCGACATC
GAGATCGTGGAGGCGACCAGCGTGACGGGCTCCTCGCTGTCCACGGTCACGCAGAGCGCC
ATGAGGGCCAAGCAGGCCAACAAG---CAGGACAACAAC
>MN168393
GAGCTACGGAGTCGGCAGTTCTGGAGAGCCACGTTAGCAGAGCTGCTTGGCACCCTGGTG
TTAGTAAGCACCGTGCTGGGTGCCTCTGTGCCAGGTCCGGGAGAGACCCTTGGGGGACCT
CTGTACCCAGCTGTTGCAGTGGGGGTGGTGATTATTGCACTGGCACACTGTTTTGGAGAA
ATAAGCGGGGCACAGGTGAACCCAGCAGTGACTCTGTCTCTGTTGGCCACTCGGAGGGTG
GACGTTGTCAGGGCCCTTGTTTACATTGCTGCACAGTGTTTGGGGGCTTCTTTAGGAACT
GGAGCCCTCTACCTGGCCCTGCCGCTGAAAACCACAGCAGAGCACTTCGTCAACAGGGTG
CCTATAGAACTGAATGCAGCTCAGGCTCTGGGCATAGAGGTTTTGTGCACCTTCCAGATG
GTGTTCACTGTGTTCTCAGTGGAGGACCAGCGACGGAGGGACTGCCCAGAACCAGGAAAC

TTAGCCATTGGATTAGCACACACCGCTGGAGTGTTAATAGGGGCAAGGTTCTCTGGTGCG
TGTATGAATCCTGCACGTGCTCTGGGTCCCGCGATCATCACTGGTTTCTGGGAAAATCAC
TGGGTGTACTGGATCGGACCAGTGCTCGGCTCTGTACTGGGTGGAGTGTCCCATGAGTTC
TTCTTTGCACGGAGTGCCTCTCGCCAGAAGCTGGTGGCTTGTCTGACCTGTAAGGATATT
GAGATCGTGGAGACAGCCAGTATGACTGGATCATCACTGTCCACGGTCACACAGAATGCC
ATGCGAGCCAAACAGGCCAACAAA---CAGGATAATAAC
>MN168394
GAGCTACGGAGTCGGCAGTTCTGGCGAGCCATGCTGGCAGAGCTGCTCGGCACCCTGGTG
TTAGTGAGTGCCGTGCTGGGTGCCTCTGTGCCAGGCCCTGGTGAGGCCCCTGTGGGACCC
CTGTACCCAGCTGTAGCAGTGGGTGTGGTGATTGTCGCGCTGGGGCACTGTTTTGGAGAA
ATAAGTGGGGCACAGGTGAACCCTGCAGTGACTCTGTCTCTGTTGGCCACTCGGAGGCTG
GATGTTCTCAGGGCCCTTGTTTATATTGCTGCACAGTGTCTGGGGGCCTCTTTAGGAGCC
GGGGCCCTCTACCTGGCCCTGCCGCTGAAAACCACTGCAGACCACTTCATCAACAGGGTC
CCCATAGAGTTGAATGCAGGCCAGGCGCTGGGCATAGAGGTTTTGTGCACCTTCCAGATG
GTGTTCACTATCTTCTCAGTGGAGGACCAGCGACGGAGGGAGAGCCCAGAACCAGGAAAC
TTAGCCATTGGATTAGCCCACACTGCTGGAGTGTTAATAGGGGCACGATTCTCTGGTGCG
AGTATGAATCCTGCACGTTCCCTGGGCCCAGCCATCATCACTGGCTTATGGGAAAACCAC
TGGGTGTATTGGATTGGACCAGTGCTTGGTGCTGTGTTGGCCGGAGTGTCCCACGAGTTC
TTCTTTGCACGCAGCGCCTCTCGCCAGAAGCTGGTGGCGTGTCTGACCTGTAAGGACATT
GAGATTGTGGAGACGGCCAGTATGACCGGATCATCGCTGTCCACTGTCACACAGAACGCC
ATGAGAGCTAAACAGGCCAACAAA---CAAGAGAACAAC
>MN168395
GAGCTACGGAGTCGGCAGTTCTGGCGAGCCATGCTGGCAGAGCTGCTCGGCACCCTGGTG
TTAGTGAGCGCCGTGCTGGGTGCCTCTGTGCCAGGCCCTGGTGAGGCCCCTGTGGGACCC
CTGTACCCAGCTGTAGCAGTGGGTGTGGTGATTGTTGCGCTGGGGCACTGTTTTGGAGAA
ATAAGTGGGGCACAGGTGAACCCTGCAGTGACTCTGTCTCTGTTGGCCACTCGGAGGCTG
GATGTTCTCAGGGCCCTTGTTTATATTGCTGCACAGTGTCTGGGGGCCTCTTTAGGAGCC
GGGGCCCTCTACCTGGCCCTGCCGCTGAAAACCACTGCAGACCACTTCATCAACAGGGTA
CCCATAGAGTTGAACGCAGGCCAGGCGCTGGGCATAGAGGTGTTGTGCACCTTCCAGATG
GTGTTCACTATCTTCTCAGTGGAGGACCAGCGACGGAGGGAGAGCCCAGAACCAGGAAAC
TTAGCCATTGGATTAGCCCACACTGCTGGAGTGTTAATAGGGGCACGATTCTCTGGTGCG
AGTATGAATCCTGCACGTTCCCTGGGCCCAGCCATCATCACTGGCTTATGGGAAAACCAC
TGGGTGTATTGGATTGGACCGGTGCTTGGTGCTGTGTTGGCCGGAGTGTCCCACGAGTTC
TTCTTTGCACGCAGCGCCTCTCGCCAGAAGCTGGTGGCGTGTCTGACCTGTAAGGACATC
GAGATTGTGGAGACGGCCAGTATGACCGGATCATCGCTGTCCACTGTCACACAGAACGCC
ATGAGAGCTAAACAGGCCAACAAA---CAAGAGAACAAC
>MN168396
GAGCTACGGAGTCGGCAGTTCTGGCGAGCCATGCTGGCAGAGCTGCTCGGCACCCTGGTG
TTAGTGAGCGCCGTGCTGGGTGCCTCCGTGCCAGGCCCTGGTGAGGCCCCTGGGGGACCA
CTGTACCCAGCTGTAGCAGTGGGTGTGGTGATTGTTGCGCTGGGGCACTGTTTTGGAGAA
ATAAGTGGGGCACAGGTGAACCCTGCAGTGACTCTGTCTCTGTTGGCCACTCGGAGGCTG
GATGTTCTCAGGGCCCTTGTTTATATTGCTGCACAGTGTCTGGGGGCCTCTTTAGGAGCT
GGGGCCCTCTACCTGGCCCTGCCATTGAAAACCACTGCAGACTACTTCATCAACAGGGTG
CCTATAGAGTTAAATGCTGCCCAGGCGCTGGGCATAGAGGTTTTGTGCACCTTCCAGATG
GTGTTCACTGTGTTTTCAGTGGAGGACCAGCGACGGAGGGAGAGCACAGAACCAGGGAAT
TTAGCCATTGGATTAGCCCACACTGCTGGAGTGTTAATAGGGGCACGATTCTCTGGTGCG
AGTATGAATCCTGCACGTTCTCTGGGCCCAGCCATCATCACTGGCTTCTGGGAAAACCAC
TGGGTGTATTGGATCGGACCAGTGCTTGGTGCCGTGCTGGCCGGAGTGTCCCACGAGTTT
TTCTTCGCTCGCAGCGCCTCTCGTCAGAAGCTGGTGGCGTGTCTGACCTGTAAGGACATC
GAGATTGTGGAGACGGCCAGTATGACCGGATCATCACTGTCCACAGTCACACAGAACGCC
ATGAGAGCCAAACAGGCCAACAAA---CAAGAGAACAAC

>MN168397
GAGCTGCGGAGTCGGCAGTTCTGGCGAGCAATGCTGGCAGAGCTGCTCGGCACCCTGGTG
TTAGTGAGTGCCGTGCTGGGTGCCTCTGTGCCAGGCCCTGGCGAGGCCCCTGGGGGACCC
CTGTACCCAGCTGTAGCTGTGGGTGTGATGATTGTTGCACTGGGGCACTGTTTTGGAGAA
ATAAGTGGGGCACAGGTGAACCCTGCAGTGACTCTTTCTCTGCTGGCCACTCGGAGGCTC
GATGTTCTCAGGGCTCTTGTTTATATTGCTGCCCAGTGTCTGGGGGCCTCCCTTGGAGCC
GGGGCCCTCTACCTGGCCCTGCCACGGAAATCTACTGCAGAACACTTTGTCAACAAGGTT
CCTATAGAGTTGTATGCTGCACAGGCGCTGGGCATCGAGGTTCTGTGCACCTTCCAGATG
GTCCTCACGGTCTTTTCGGTGGATGAACAGCGACGGAGGGAGAGCCCAGAACCAGGAAAC
TTAGCCATCGGATTAGCTCACTCTGCTGGAGTGTTTATAGGGGGGCGATTCTCTGGTGCA
AGTATGAATCCTGCTCGTTCTCTGGGCCCAGCTATCATCACCGGTTTCTGGGAAAACCAC
TGGGTGTATTGGATCGGACCAGTGCTTGGTGCCATGCTGGCTGGAGTCTCCCATGATTTC
TTCTTTGCACGCAGCGCCTCTCGTCAGAAGCTGGTGGCGTGTCTGACCTGTAGGGACATT
GAGATTGTGGAGACAGCCAGTATGACCGGATCATCACTGTCCACAGTCACGCAGAATGCC
ATTAGAGCCAAACAGGCCAACAAG---CAGGAGAACAAC
>MN168398
GAGCTACGTAGCCGGCAGTTCTGGCGTGCCATGCTGGCAGAGCTGCTCGGGACCCTGATA
TTGGTGAGCGCCGTGCTGGGTGCCTCTGTGCCGGGCCCTGGAGACGTCACTGGAGGACCC
ATGTACCCAGCGGTGGCTGTGGGTGTGGTGGTTGTTGCACTAGGACACTGTTTTGGAGAA
ATAAGCGGAGCACAGATGAACCCTGCAGTGACTCTGTCTCTGTTGGCCACACGGCGGCTG
GACGTTCTCAGGGCGCTTGTTTATATGGCTGCACAGTGTGTGGGGGCCTCTTTAGGAGCC
GGGGCCCTTTACCTGGCCCTACCGCTGAAAACCACTGCAGACCACTTTGTCAACAGGGTG
CCTATAGAGTTGAACGCAGCCCAGGCTCTGGGCACAGAGGTTTTGTGCACCTTCCAGTTG
GTCTTAACTGTGTTCTCAGTGGAGGACCAGCGTCGGAGGGAGAGCCCAGAACCAGGAAAC
CTCGCTATTGGATTAGCACACACTGCTGGAGTACTAATAGGGGCAAGGTTCTCTGGTGCG
AGTATGAACCCTGCACGCTCTCTGGGTCCAGCCATCATCACTGGCTTCTGGGAAAATCAC
TGGCTTTATTGGGTTGGACCTGTTCTTGGTGGGGTACTAGCCGGAGTGTCCCATGAGTTC
TTCTTTGCACGCAGCGCCTCTCGCCAGAAGCTGGTGGCGTGTCTGACCTGTAAAGATATC
GAGATCGTGGAGACAGCCAGTATGACGGGGTCGTCACTGTCCACGGTCACGCAGAACGCC
ATGAGAGCCAAGCAGGCCAACAAA---CAAGAGAACAAC
>MN168399
GAGCTGCGTAACCGGCAGTTCTGGCGTGCCATGCTGGCAGAGCTGCTTGGCACCCTCATA
TTGGTGAGTGCTGTCCTGGGTGCCTCTGTGCCAGGACCTGGAGAGGTCACAGGGGGACCC
CTGTATCCAGCAGTGGCTGTGGGAGTGGTGGTTGTTGCACTAGGACACTGTTTTGGAGAA
ATAAGCGGAGCACAGATGAACCCTGCAGTGACTCTGTCTCTGTTGGCCACACGGAGGCTG
GACATTGTCAAGGCGCTTGTTTACATGACTGCGCAGTGTTTGGGGGCCTGTTTAGGAGCA
GGGACCCTTTACCTGGCCCTACCACTGAAAAGCACTGCAGACTACTTTGTTAACAGGGTG
CCCATAGAGTTGAATGCAGCCCAGGCTCTGGGCATAGAGGTTTTGTGCACCTTCCAGCTG
GTCTTAACCGTGTTTTCAGTGGAGGACCAGCGACGGAGGGAGAGCCCAGAGCCAGGAAAC
CTGGCCATTGGTTTAGCACACACTGCTGGAGTACTAATAGGAGGAAGGTTTTCTGGTGCC
AGTATGAATCCTGCACGTTCTCTGGGTCCAGCTATCATCACTGGCTTCTGGGAAAACCAC
TGGGTGTACTGGATTGGGCCGGTGCTTGGTGCTGTAATAGCCGGAGTGTCCCACGAGTTC
TTCTTTGCCCGCAGTGCCTCACGCCAGAAGCTGGTGGCATGTCTGACCTGTAAGGATATT
GAGATTGTGGAGACAGCCAGTATGACTGGATCCTCGCTGTCCACAGTTACACAGAATGCC
ACGAGAGCCAAGCAGGCCAACAAA---CAAGAGAACAAC
>MN168400
GAGCTACGCAGTCGGCAGTTTTGGCGCGCCATGCTGGCGGAGCTGCTGGGCACCCTGGTG
TTGGTGAGCGCCGTGCTGGGAGCCTGTGTGCCAGGCCACGGGGAGGTCACGGGGGGACCC
TTGTACCCCGCGGTGGCCGTGGGTGTGGTAGTTGTTGCAATGGCACACTGTTTTGGGGAC
GTGAGCGGGGCACAGATGAACCCTGCTGTGACTCTGTCTCTGTTGGCCACGCGGAGGCTG
GACGTTCTCAGGGCGCTGGTTTATGTGGCTGCACAGTGTCTGGGGGCCTCTCTGGGGGCC

GGGGCCCTCTACTTGGCCCTGCCAGTGAAGGCTACTGCAGATTACTTCGTTAACAGGGTC
CCCTTAGAGTTGAATGCGGCCCAGGCTCTGGGGGTAGAGATGTTGTGCACCTTTCAGTTG
GTCTTAACCGTGTTCTCAGTGGAGGAGCAACGACGCAGGGAGAGTCCAGAACCAGGGAAC
TTGGCCATCGGATTAGCTCACACCGCTGGAGTGCTAATAGGGGCCAGGTTTTCTGGTGCA
AGTATGAATCCCGCACGCTCCTTGGGTCCAGCCATCGTCACTGGCTTCTGGGAAAATCAC
TGGGTGTACTGGATCGGACCGGTGCTTGGTGCCGTTCTAGCTGGAGTGTCCCATGAGTTC
TTCTTCGCACGCAGTGCCTCTCGCCAGAAGCTGGTGGCCTGTCTGACCTGCAAGGATATT
GAGATTGTGGAGACGGCCAGTATGACAGGGTCCTCGCTGTCCACCGTCACACAGAATGCC
CTGAGAGCCAAACAGGCCAACAAA---CAGGAAAACAAC
>MN168401
GAGCTACGTAGCCGGCAGTTCTGGCGTGCCATGCTGGCAGAGGTGCTAGGCACCCTGGTT
TTGGTGAGCACTGTGCTGGGTGCCTCAGTGCCAGGCCCTGCAGATGCCCCCAGGGGACCC
CTGTACCCAGCTGTGGCCGTGGGTGCGGTGATTGTTGCACTGGGACACTGCTTTGGAGAA
ATAAGTGGGGCACAGGTGAACCCTGCAGTGACTCTGTCTCTGTTGGCCACTCGGAGGTTG
AATGTTCTCAGGGCTCTTGTTTATATCACTGCGCAGTGTTTGGGGGCCTCTTTAGGAGCC
GGGGCCCTCTACCTGGCCCTGCCACCGAAGACCACTGCAGAGTTCTTTGTCAACAAGGTG
CCTGTAGACTTGCACGCAGCCCAGGCTCTGGGTATAGAGATTTTGTGCACCTTCCAGATG
GTCTTGACTGTGTTCTCAGTGGAGGACCAGAGACGAAGGGAGAGTCCAGAACCAGGAAAC
TTAGCCATTGGATTAGCACACACTGCTGGAGTGCTAATAGGGGCGCGGTTCTCTGGTGCG
AGTATGAATCCTGCACGTTCTCTGGGTCCAGCCCTCATCACTGGCATCTGGGAAAATCAC
TGGGTGTATTGGATCGGACCGGTGCTCGGTGCTGTGCTGGCTGGAGTGTCCCATGAGTTC
TTCTTTGCACGGAGCGCCTCCCGCCACAAACTGGTGGCGTGTCTGACCTGCAAGGATATT
GACATTGTGGAGACGGCCAGTATGACCGGATCATCGCTTTCCACGGTCACGCAAAACGCC
ATGAGAGCCAAGCAGGCCAACAAA---CAAGAGAACAAC
>MN168402
GAGCTACGTAGTCGGCAGTTCTGGCGTGCCATACTGGCAGAACTAATAGGCACCCTGGTT
TTGGTGAGCGCCGTGCTGGGTGCCTCTGTTCCAGGCCCTGGAGAGGTCCCTGGGGGACCC
CTGTACCCGGCGGTGGCAGTGGGGGTGGTGATTGTTGCACTGGGACACTGTTTTGGAGAA
ATAAGTGGGGCACAGGTGAACCCTGCAGTGACTCTGTCTCTGTTGGCCACTCGGAGGCTG
GATGTTCTCAGGGCTCTTGTTTATATTGTGGCGCAGTGTTTGGGAGCTTCTTTAGGGGCC
GGGGCCCTCTACCTGGCTCTGCCACTGAAAACCACCGCAGACTTCTTCGTCAATAGGGTA
CCTATAGAGTTGCATGCAGCTCAGGCTCTGGGCATAGAGTTTTTGTGCACCTTCCAGATG
GTCTTAACTGTGTTCTCAGTGGAGGACCAACGACGGAGGGAGAGTCCAGAACCAGGAAAC
TTGGCCATTGGGCTTGCACACACTGCAGGCGTACTGATAGGGGCGCGGTTCTCTGGAGCG
AGTATGAATCCTGCACGTTCATTGGGTCCAGCCATTATCACTGGCATCTGGGAATATCAC
TGGGTGTATTGGATCGGACCGGTGCTTGGTGCTGTACTGGCTGGCGTGTCTCATGAGTTC
TTCTTTGCACGCAGCGCCTCTCGCCAGAAGCTGGTGGCGTGTCTGACCTGTAAGGATATT
GAGATTGTAGAGACGGCCAGTATGACCGGATCGTCTCTTTCCACGGTGACACAGAACGCA
ATGAGAGCCAAGCAGAACAACAAG---CAAGAGAACAAC
>MN168403
GAGCTTCGTAGCCGGCAGTTCTGGCGAGCCATGATGGCGGAGCTGATCGGCACGCTGGTG
CTGGTGAGCGCCGCGCTGGGTGCGTCCGTACCGGTCCGTGGGGAGGCCCCGGAGGGATCC
TTGTCCCCTGCGGTAGCAGTCGGTGCGGTGGTTGTCGTACTGGGGCACTGTTTTGGGGAA
ATAAGCGGGGCGCAGGTCAACCCTGCAGTCACTTTGTCCCTCCTGGCCACGCGGAGGCTG
GACGTCCTGAGGGCTCTCATTTATATTGCTGCCCAGTGTTTGGGGGCTTTATTGGGGGCC
GGGGCCCTCTACTTGGCTTTGCCCCTCAAAAGCACTGCAGACAACTTTGTCACCAGGGTG
CACATTGAGTTGAATGCAGCGCAGGCCCTTGGCATTGAGGTCTTATGCACCTTCCAGTTG
GTTTTCACAATTTTCTCAGTTGAGGCCCAGCGACGAAGGGACAGCCCGGAACCAGGAAAC
CTTGCTATTGGACTGGCACACACTGCTGGAGTGATGATAGGGGCTCGGTTCTCTGGTGCT
AGTATGAACCCTGCTCGCTCTCTTGGTCCTGCTATCATCACAGGATTTTGGGAAAACCAT
TGGGTGTACTGGATCGGACCGGTCTTCGGGGCAGTACTGGCCGGTGTGTCGCATGAATTC

TTCTTTGCACAAAGCGCCTCGCGCCAAAAGCTGGTGGCATGCCTGACGTGCAAAGACATT
GAGATCGTGGAGACCACCAGCATTACCGGCTCGTCCCTGTCCACCGTCACTCAGAACGCT
GCCAGAGCCAAACAGGCCCACAAA---CACGACAACAAC
>MN168404
GAGCTCCGTAGCCGTCAGTTCTGGCAAGCCATGATGGCGGAGCTGATCGGCACGCTGGTG
CTGGTGAGCGCTGTGCTGGGTGCTTCCGTACCGGACCGTGACGAGACCCCGGTGGGACCC
TTGTACCCAGCAGTGGCAGTCGGCGCAGTGATTGTCGTGCTGGGACACTGTTTTGGGGAA
ATAAGCGGAGCACAGGTCAACCCTGCAGTGACTTTATCTCTCCTGGCCACACGAAGGCTG
GATGTCCTAAGGGCCCTCATTTATATTGCCGCCCAGTGTTTGGGGGCTTTATTGGGGGCC
GGGGCCCTCTACTTGGCTTTGCCGCGCAAAATCACTGCAGGCTACTTCGTCACCCGGGTG
CACGATGAGTTGAATGCAGCGCAGGCCCTTGGCATCGAGGTCTTATGCACCTTCCAGTTG
GTTTTCACCGTTTTCTCAGTTGAGGCCCAGCGACGAAGGGAAAGCCCAGAACCAGGAAAT
TTGGCTATTGGACTGGCGCACACTGCAGGAGTCATGATAGGGGCTCGGTTCTCTGGTGCC
AGTATGAACCCTGCTCGCTCTCTTGGTCCGGCCATCATCACGGGATTTTGGGAAAACCAT
TGGGTGTATTGGATCGGACCGGTCATCGGAGCAGTACTTGCCGGTGTGTCCCATGAATTC
CTCTTTGCACAAAGCGCCTCTCGCCAGAAGTTGGTTGCGTGTCTGATGTGCAAAGACATT
GAAATCGTGGAGACGACCAGCATGACTGGCTCGTCCCTGTCTACCGTCACTCAGAATGCT
GCCAGAGCCAAACAGGCCCACAAA---CAAGACAAC---
>MN168405
GAGCTTCGAAGTCGTCAGTTCTGGCGTTCCATACTGGCAGAGCTGCTTGGCACCATGGTG
TTGGTGAGTGCCGTGCTGGGTGCGTCTGTGCCAGGCCCTGGAGAGGCCCCCGGGGGACCC
CTGTACCCAGCAGTGGCAGTGGGTGTAGTGATTGTTTCACTGGCCCACTGTTTTGGAGAG
ATTAGTGGGGCACAGGTGAACCCTGCTGTGACTCTGGCTCTGCTGGCCACACGGAGGCTT
GAGGTCCTCAGGGCCCTTGTTTATATCGTTGCACAGTGTTTGGGGGCTTCTTTAGGGGCC
GGGGCCCTCTACCTGGCCCTGCCGCTCAAAACCACTGCAGACTACTTTGTCAACAAGGTC
CCAATAGAGTTGTATGCAGCCCAGGCTCTGGGCATAGAGGTTTTGTGCACCTTCCAGATG
GTCTTCACTGTGTTCTCAGTGGAGGACCAGCGACGGAGGGAGAGCCCAGAACCAGGAAAT
CTGGCCATTGGATTAGCACACTCTGCTGGAGTGCTAATAGGGGCGCGGTTCTCTGGTGCA
AGTATGAACCCTGCACGTTCTCTGGGTCCAGCCATCATCACTGGCTTCTGGGAAAACCAC
TGGGTGTACTGGATGGGACCGGTGATCGGTGCTATACTGGCCGGCGTGTGCCACGAGTTC
TTCTTTGCACGCAGCGCCTCTCGCCAGAAGCTGGTGGCATGCCTGACCTGTAAGGACATC
GAGATCGTGGAGACGGCCAGCATGACCGGATCATCGCTGTCCACGGTCACACAGAACGCC
ATGAGAGCCAAGCAGGCCAACAAA---CAAGAGAACAGA
>MN168406
GAGCTTCGAAGTCGTCAGTTCTGGCGTTCCATACTGGCAGAGCTGCTTGGCACCATGGTG
TTGGTGAGCGCTGTGCTGGGTGCGTCTGTGCCAGGCCCTGGAGAGGCCCCCGGGGGACCC
CTGTACCCAGCAGTGGCAGTGGGTGTAGTGATTGTTTCACTGGCCCACTGTTTTGGAGAG
ATTAGTGGGGCACAGGTGAACCCTGCTGTGACTCTGGCTCTGCTGGCCACACGGAGGCTA
GAGGTCCTCAGGGCCCTTGTTTATATCGTTGCACAGTGTTTAGGGGCTTCTTTAGGGGCC
GGGGCCCTCTACCTGGCCCTGCCGCTCAAAACCACTGCAGACTACTTTGTCAACAAGGTG
CCAATAGAGTTGTATGCAGCCCAGGCTCTGGGCATAGAGGTTTTGTGCACCTTCCAGATG
GTCTTCACTGTGTTCTCAGTGGAGGACCAGCGACGGAGGGAGAGCCCAGAACCAGGAAAT
CTGGCCATTGGATTAGCACACTCTGCTGGAGTGCTAATAGGGGCGCGGTTCTCTGGTGCG
AGTATGAACCCTGCACGTTCTCTGGGTCCAGCCATCATCACTGGCTTCTGGGAAAACCAC
TGGGTGTACTGGATGGGACCGGTGATCGGTGCTATACTGGCCGGCGTGTGCCACGAGTTC
TTCTTTGCACGCAGCGCCTCTCGCCAGAAGCTGGTGGCATGTCTGACCTGTAAGGACATC
GAGATCGTGGAGACGGCCAGCATGACCGGATCATCACTGTCCACGGTCACACAGAACGCC
ATGAGAGCCAAGCAGGCCAACAAA---CAAGAGAACAGA
>MN168407
GAGCTTCGAAGTCGTCAGTTCTGGCGTTCCATACTGGCAGAGCTGCTTGGCACCATGGTG
TTGGTGAGCGCCGTGCTGGGTGCGTCTGTGCCAGGCCCTGGAGAGGCCCCCGGGGGACCC

CTGTACCCAGCAGTGGCAGTGGGTGTAGTGATTGTTTCACTGGCCCACTGTTTTGGAGAG
ATTAGTGGGGCACAGGTGAACCCTGCTGTGACTCTGGCTCTGCTGGCCACACGGAGGCTT
GAGGTCCTCAGGGCCCTTGTT---------GCACAGTGTTTGGGGGCTTCTTTAGGGGCC
GGGGCCCTCTACCTGGCCCTGCCGCTCAAAACCACTGCAGACTACTTTGTCAACAAGGTG
CCAATAGAGTTGTATGCAGCCCAGGCTCTGGGCATAGAGGTTTTGTGCACCTTCCAGATG
GTCTTCACTGTGTTCTCAGTGGAGGACCAGCGACGGAGGGAGAGCCCAGAACCAGGAAAT
CTGGCCATTGGATTAGCACACTCTGCTGGAGTGCTAATAGGGGCGCGGTTCTCTGGTGCG
AGTATGAACCCTGCACGTTCTCTGGGTCCAGCCATCATCACTGGCTTCTGGGAAAACCAC
TGGGTGTACTGGATGGGACCGGTGATCGGTGCTATACTGGCCGGCGTGTGCCACGAGTTC
TTCTTTGCACGCAGCGCCTCTCGCCAGAAGCTGGTGGCATGTCTGACCTGTAAGGACATC
GAGATCGTGGAGACGGCCAGCATGACCGGATCATCGCTGTCCACGGTCACACAGAACGCC
ATGAGAGCCAAGCAGGCCAACAAA---CAAGAGAACAGA
>MN168408
GAGCTCCGCAGTCGGCAGTTTTGGCGTGCGATGCTCGCAGAGCTGCTCGGCACCCTGGTG
TTCGTCAGCACCGTGCTGGGTGCTTCTATTCCAGGTTCCGGTGAGGTCCCCGGGGGCCCC
ATGTACCCAGCCGTGGCAGCGGGGGTTGCGATTGTTGCTGTGGCTCACTGTTTTGGAGAA
ATAAGTGGTGCACAGGTCAACCCTGCGGTCACTCTGTCTCTGCTGGCGACTCGGAGGGTG
GATGTACTCAGGGCCCTTGTTTACGTCGCGGCGCAGTGCTTGGGGGCCTCTTTAGGAGCC
GGGGCCCTCTATCTGGCCCTGCCACTCAAACGCACAGCTGACTACTTTGTCAACAGGGTG
CCCATAGAGCTGAATGCAGCCCAGGCTCTGGGGATAGAGGTTTTGTGCACGTTCCAGCTG
GTTTTCACCGTATTCTCAGTCGAGGAGCAGCGGCGGAAAGACGGCGCAGAACCTGGAAAC
CTGGCTATTGGACTTGCACACACCACTGGAGTGCTAATTGGAGCGCGCTTCTCTGGTGCG
AGTATGAACCCTGCACGTTCTCTGGGCCCGGCCATCATCACTGGATTCTGGGAAAACCAC
TGGGTGTACTGGATCGGACCACTGGTTGGCGCTGTGTTGGCTGGCATGTCTCATGACTTC
CTCTTCGTACGCAGCGCCTCTCGGCAGAAGTTGGTGGCTTGCCTCACCTGTAAGGATATA
GAAATCGTGGAGACGGCCAGTATGACAGGATCATCAATGTCCACGGTCACACAGAACGCC
ACGAGGGCCAAACAGGCCAACAAA---CAAGAGAGCAAC
>MN168409
GAGCTTCGTAGTCGTCAGTTTTGGCGTGCGATGCTCGCAGAGCTGCTCGGCACCCTGGTG
TTTGTGAGCACCATGCTGGGTGCCTCTATTCCAGGTCCCGGTGAGGTCCCGGGGGGGCCG
CTGTACCCAGCCGTGGCAGCGGGTGCCGTGATTGTTGCGCTGGCTCACTGTTTTGGAGAA
ATAAGCGGCGCACAGGTGAACCCTGCAGTCACTCTGTCTCTGCTGGCGACCCGGAGGGTG
GATGCACTCAGGGCCCTTGTTTATGTAGCTGCACAGTGCTTGGGGGCCTCTTTAGGAGCT
GGGGCCCTCTACCTGGCCCTGCCACTCAAAGGCACAGCAGACTACTTTGTCAACAAGGTG
CCCATAGAGCTGAATGCAGCCCAGGCTCTGGGGATCGAGGTTTTGTGCACATTCCAGCTG
GTCTTCACCATATTCTCAGTGGAGGAGCAGCGACGGAGGGAGAGCACAGAGCCGGGAAAC
CTGGCTATTGGATTTGCACACACAGCTGGAGTGCTGATTGGAGCGCGCTTTTCTGGTGCG
AGTATGAACCCTGCGCGTTCTCTGGGCCCGGCCATCATCACTGGATTCTGGGAAAACCAC
TGGGTGTACTGGATTGGACCTGTGCTCGGTGCTGTGCTGGCTGGTATGTCTCATGATTTC
CTCTTCGTGCGCAGCGCCTCTCGGCAGAAGTTGGTGGCTTGCCTCACCTGTAAGGATATT
GAAATCGTGGAGACGGCCAGTATGACGGGATCATCACTGTCCACTGTCACGCAGAACGCC
ATGAGGACCAAACAGGCCAACAAA---CAAGAGAACAAC
>MN168410
GAGCTGCGCAGTCGGCAATTCTGGCGTGCCATGCTAGCAGAGGTTCTTGGCACCCTGGTG
TTAGTGAGCACCTTGCTGGGTTCCTCTCTTCCAGGCCCCGGAGAGGCCCCTGGGGGCCCC
TTATACCCAGCGGTGGCTGTAGGTGTGGTGATTGTTGCACTGGGACACTGTTTTGGAGAA
ATTAGCGGTGCACAGGTGAACCCTTCAGTGACTCTGTCTCTGCTGGCCACTCGGAGGATA
GACGTTCTCAGAGCCCTTCTTTACATCATAGCACAGTGTCTGGGGTCCTCTTTAGGTGCT
GGGGCCCTCTACCTGGCCCTGCCACTCAAATCCACTGCAGAGAACTTTGCTAACAGGGTG
CCTTTGGAGGTGAATGCAGGACAGGCTCTGGGGATAGAAGTTCTGTGCACCTTCCAGATG
GTTTTCACCATATTCTCAGTGGAAGACCAGCGACGAAGGGAGAGCACCGAACCTGCAAAC

CTCGCCATTGGACTTGCACATACCGCTGGGGTGCTCATCGGTGCTCGGTTCTCTGGTGCA
TGTATGAATCCAGCTCGAGCTCTGGGTCCAGCCATCATCACTGGATTCTGGGAAAATCAC
TGGGTATTCTGGATAGGACCGGTGCTCGGGGCTGTCCTGGCCGGCGTCTCCCATGAGTTC
TTCTTTGCACCAACTGCCTCCCGTCACAAGCTCGTAGCCTGTCTGACCTGCAAAGATATC
GAGATTGTGGAGACAGCGAGCATGACCGGATCGTCTCTGTCCACCGTTACGCAGAACGCC
ATGAGGGCCAAGCAGGCCAACAAA---CAGGAAAACAAC
>MN168411
GAGCTCCACAGTCGTCCGTTCTGGCGTGCCGTGTTGGCAGAGCTGCTGGGCACCCTGGTG
TTAGTGAGTGCTGTTCTGGGCTCGTTGGTGCCGGGGCCCGGGGAGGCCTCTGGGGGCCCT
CTGTACCCGGCATTGGCAGTGGGCGTGGCCATAGTGGCAGTGGCGCACTGTTTTGGAGAG
ATCAGCGGTGCTCAGGTAAACCCTGCAGTGACTCTCGCCCTCCTGGCCACTCGCCGCTTG
GACATGCTCAGAGCTGTGTTCTTCATCTGTGCTCAGTGTGTGGGGGCCTGTCTCGGCACC
GGGGCCCTGTACTTGGCTCTTCCACTCAAAACCACAGCAGACCACTTTATCAATAAGGTC
CCTCTGGAGCTCAACGCCGCTCAGGCTCTGTGTGTGGAGATGCTGTGCACGTTTCAGATG
GTCTTCACAGTGTTCTCTGCAGAGGACCAGCGCCGCAGAGACAGTCCTGAGCCGGGGAAC
CTCGCTGTGGGGTTCTCTCACACCGCGGGGGTCCTGATCGGGGCGCGATTCTCTGGAGCC
AGCATGAACCCAGCGAGGACTCTGGGCCCTGCCATCATTACTGGCTTCTGGGAAAATCAC
TGGGTATACTGGATCGGGCCGGTGCTTGGGGCTGTGCTCGCGGGCGTCTCTCATGAGTTT
TTCTTCGCCCCCTCTGCGTCTCGTCAGAAGCTGGTGGCGTGTTTGACATGTAAAGACATC
GAGATTGTAGAGACGGCGAGTATGACCGGCTCTTCTTTGTCCACTGTCACGCAGAACGCC
TCCCGAGCTAAACACAGCAACAAG---AACGACAAC---
>MN168412
GAGCTCCACAGTCGTCCGTTCTGGCGTGCCGTGTTGGCAGAGCTGCTGGGCACCCTGGTG
TTAGTGAGTGCCGTTCTGGGCTCATTGGTGCCGGGGCCCGGGGAGGCCTCTGGGGGCCCT
CTGTACCCGGCACTGGCAGTGGGCGTGGCCATAGTGGCGGTGGCGCACTGTTTTGGAGAG
ATCAGTGGTGCTCAGGTAAACCCTGCAGTGACTCTAGCCCTCCTGGCCACTCGCCGCATG
GACATACTCAGAGCTGTTTTCTTCATCTGTGCTCAGTGTGTAGGGGCCTGTCTCGGCACC
GGGGCCCTGTACTTGGCTCTTCCACTCAAAACCACAGCAGACCACTTTATCAATAAGGTA
CCTCTGGAGCTGAACGCGGCTCAGGCTCTGTGTGTGGAGATGCTGTGCACATTTCAGATG
GTCTTCACAGTGTTCTCTGCAGAGGACCAGCGCCGCAGGGACAGTCCTGAGCCAGGAAAC
CTTGCTGTGGGGTTTTCTCACACCGCGGGGGTCCTAATCGGGGCACGATTCTCTGGAGCC
AGCATGAACCCAGCAAGGACTCTGGGCCCTGCCATCATCACTGGCTTCTGGGAAAATCAC
TGGGTATACTGGATCGGGCCAGTGCTAGGGGCCGTGCTTGCTGGGGTCTCTCATGAATTC
TTCTTCGCCCCCTCTGCATCTCGTCAGAAGCTGGTAGCGTGTTTGACATGTAAGGACATC
GAGATTGTAGAGACAGCGAGTATGACCGGATCTTCTCTGTCCACCGTCACACAGAATGCT
GCCCGAGCTAAACAAAGCAACAAG---ACTGAGAAC---
>MN168413
GAGCTCCACAGTCGTCCGTTCTGGCGTGCCGTGTTGGCAGAGCTGTTGGGCACCCTGGTG
TTAGTGAGTGCCGTTCTGGGCTCGTTGGTGCCGGGGCCCGGGGAGGCCTCTGGGGGCCCT
CTGTACCCGGCACTGGCAGTGGGCGTGGCCATAGTGGCGATGGCACACTGTTTTGGAGAG
ATCAGCGGTGCTCAGGTGAACCCAGCGGTGACTCTAGCTCTCCTGGCCACTCGCCGCATG
GACTCGCTCAGAGCCGTGTTCTTCATCTGTGCTCAGTGTGTGGGCGCCTGTCTCGGCACC
GGAGTCCTGTACTTGGCTCTTCCTCTCAAAACTACAGCAGACCACTTTGTCAATAAGGTG
CCTCTGGAGCTGAACGCAGCTCAGGCTCTGGGTGTGGAGATCCTCTGCACGTTTCAGATG
GTCTTCACAGTGTTCTCTGCAGAGGACCAGCGACGCAGGGACAGTCCAGAACCAGGGAAC
CTCGCTGTGGGGTTTGCCCACACCGCCGGGGTACTGATAGGGGCACGCTTCTCTGGAGCC
AGCATGAACCCGGCAAGGACTCTGGGCCCTGCCATCATCACTGGCTTCTGGGAGAATCAC
TGGGTGTACTGGATCGGGCCAGTGCTAGGGGCCGTGCTCGCGGGGGTCTCTCATGAGTTC
TTCTTTGCCCCCTCGGCGTCTCGTCAGAAGCTGGTGGCGTGTTTGACGTGTAAAGACATA
GAGATCGTAGAGACGGCGAGTATGACCGGCTCCTCACTGTCCACTGTCACACAAAATGCC
ATCCGAGCCAAACAAGGTAACAAG---ACCGACAAC---

>MN168414
GAGCTCCACAGTCGTCCGTTCTGGCGTGCCGTGTTGGCAGAGCTGTTGGGCACCCTGGTG
TTAGTGAGTGCTGTTCTGGGCTCGTTGATGCCGGGGCCCGGGGAGGCCTCTGGGGGCCCT
CTGTACCCGGCACTGGCAGTGGGCGTGGCCATAGTGGCGGTGGCTCACTGTTTTGGAGAG
ATCAGCGGTGCTCAGGTGAACCCAGCGGTGACCCTTGCCCTCCTGGCCACTCGCCGCGTG
GACTCTCTCAGAGCTGTGTTCTTCATCTGTGCTCAGTGTGTGGGCGCCTGTCTAGGCACC
GGAGCCCTGTACTTGGCTCTTCCACTCAAAACTACAGCAGACCACTTTGTCAATAAGGTG
CCTCTGGAGCTGAACGCGGCTCAGGCTCTGGGTGTGGAGATCCTCTGCACGTTTCAGATG
GTCTTCACAGTGTTCTCTGCAGAGGACCAGCGCCGCAGGGACAGTCCAGAACCAGGGAAC
CTCGCTGTGGGGTTTGCCCACACTGCAGGCGTGCTGATAGGGGCACGTTTCTCTGGAGCC
AGCATGAACCCAGCAAGGACTCTGGGCCCTGCCATCATCACTGGCTTCTGGGAGAATCAC
TGGGTGTACTGGATCGGGCCAATTCTAGGGGCCGTGCTCGCAGGGGTCTCTCATGATTTC
TTCTTTGCGCCCTCGGCATCTCGTCAGAAGTTGGTGGCGTGTTTGACATGTAAAGACATC
GAGATCGTAGAAACAGCGAGTATGACCGGCTCCTCACTGTCCACGGTCACACAGAACGCT
ATCCGAGCCAAACAAGGTAACAAG---ACGGACAAC---
>MN168415
GAGCTTCGTAGTCGGCAGTTCTGGCGTGCCATCCTGGCAGAATTGCTTGGCACCCTGCTG
CTGGTGAGTGCCGTGCTGGGGGCCTCTGCGCCAGGCCCAGGAGAAACCCCTGGAGGGCCC
CTGTACCCAGCCGTGGCGGTGGGGACGGTGATAGTGTCGCTGTGCCATTGTTTTGGAGAA
ATCAGCGGGGCACAGGTGAACCCGTCTGTGACCTTGTCAATGATGGTGACGCGGAGGATA
GATGTTCTCAGGGCGCTTCTCTACGTGGCTGCACAGTGTGCAGGGTCCTTCCTGGCGGCA
GGGATCTTCTACTTGGCCCTGCCACGCAAAGCCATGTCTGACTTCTTGGTCAATAAGGTG
CCTTTGGAGCTGAACGCGGCCCAGGCTCTGGGGATGGAGGCACTGTGCACTTTCCAGATG
GTCTTCACCGTGTTCTCAGTGGAGGATCAGCGACGCAGGGAGAGCCCAGAACCGGCGAAC
CTAGCAATTGGATTCGCTCACACATCTGCCATTCTAATTGCCGCTCGCTTCTCTGGTGCC
TGCATGAATCCTGCTCGAGCTCTTGGTCCAGCCATCATCACTAACTTCTGGGAGCATCAC
TGGGTGTTCTGGATTGGACCACTGGTGGGTGGGGTCATGGCCGGCCTCTCTTACGAGTTC
CTGTTTGCACGCAGCGCCTCCCGCCAGAAGCTGGTGGCCTGTCTGACCTGTAAGGACATT
GAGATTGTGGAGATGGCCAGTGTGTCCCGATCATCACTGTCCACAGTCACGCAGAACGCT
TCACGCTCCAAGCAGGGCAACAAA---CAGGAGAACAAC
>MN168416
GAGCTCCGTAGTCGGCAGTTCTGGCGTGCCATCCTGGCAGAATTACTTGGCACCCTGCTG
TTGGTGAGTGCCGTGCTGGGGGCCTCCGTGCCAGGCCCTGGAGAGGCCCCGGGAGGACCT
CTGTACCCAGCGGTGGCCGTGGGCGCGGTGATTGTCGCGCTGGGCCACTGTTTCGGAGAG
ATCAGCGGGGCGCAGGTGAACCCCTCGGTCACTCTGTCTCTGATGGCCACTCGGAGGATA
GACGTTCTCAGGGCCCTGCTCTATGTGGCTGCCCAGTGCGTGGGGGCCTTCCTGGCAGCA
GGGATCTTCTACCTGGCTCTGCCGCACAAAGGCCTGGCAGACTACTTGGTCAACAAGGTT
CCTATAGAGCTGAATGCCGCCCAGGCCCTGGGGATGGAGGCTCTGTGTACCTTCCAGATG
GTCTTCACCGTGTTCTCCGTGCAGGACCAGCAGCGCAGGGAGAGCACCGAACCGGCGAAC
CTGGCCCTCGGGTTTGCTCACGTTTCAGCCATCCTAATAGCGGCTCGGTTCTCTGGTGCC
TGCATGAATCCTGCTCGGGCTCTGGGTCCGGCCATCATCACCAACTTCTGGGAAAACCAC
TGGGTGTTCTGGATTGGGCCCGTGATGGGTGGAGTTCTGGCTGGAATCTCCTACGAGTTC
CTGTTTGCACGTTCCGCTTCCCGTCAGAAGTTGGTGGCGTGTCTGACCTGTAAAGACATC
GAGATCGTGGAGACGGCCAGCGTGTCTCGGTCATCGCTGTCCACGGTCACACAGAACGCT
GCTCGAGTCAAACAGGCAAACAAG---CAGGAGAACAAC
>MN168417
GAGCTTCGTAGTCGGCAGTTCTGGCGTGCCATCCTGGCAGAATTGCTTGGCACCCTGCTG
CTGGTGAGTGCCGTGCTGGGTTCCACTGTGCCAGGCCCTGGAGATGCCCCAGGAGGGCCC
TTGTACCCAGCCGTGGCAGTGGGTGCGGTGATTGTCGCACTGTGCCACTGTTTTGGAGAG
ATCAGCGGGGCACAGGTGAACCCATCAGTGACCCTGTCTCTGATGGCCGCACGAAGGATA
GATGTTCTCAGGGCCCTTTTTTATGTGGCTGCACAGTGTGTGGGGGCCTTCCTGGCAGCT

GGGATCTTCTATCTGGCTGTGCCACACAAAGCCATTGCAAACTCCTTTGTCAACAAGGTG
CCTATAGAGCTGAATGCAGCCCAGGCTCTGGGGATGGAGGCTTTGTGCACTTTCCAAATG
GTCTTCACTGTGTTCTCTGTGGAGGACCAGCGGCGCAGGGAGAGCCCAGAACCAGCAAAC
CTGGCCATTGGATTTGCTCACGTCTCTGCAGTGCTGATAGCGGCCCGGTTCTCTGGTGCG
TGTATGAATCCTGCTCGTGCTCTGGGTCCAGCCATCATCACTAACTTCTGGGAAAATCAC
TGGGTGTTCTGGATCGGGCCGGTGACGGGTGGGGTTCTGGCTGGCCTCTCTTACGAGTTC
TTGTTCGCACGCAGCGCCTCCCGCCAGAAGCTGGTGGCGTGTCTGACCTGTAAGGACATT
GAGATCTTGGAGACGGCCAGCGTGTCCCGATCGTCGCTGTCCACGGTCACGCAGAACGCC
AACAGAGCCAAGCAGGCAAACAAG---CAGGAGAGCAAC
>MN168418
GAGCTGCGCAGTCGGCAGTTCTGGCGTGCCATGCTGGCAGAGCTGCTCGGCACCCTGGTG
CTCGTGAGCGCCGTGCTGGGGGCCTCTGTGCCAGGCCCTGGAGAGGCCCCCGGGGGACCG
CTGTACCCGGCCGTGGCAGTGGGTGTGGTGATTGTCGCACTGGGCCACTGTTTTGGAGAG
ATCAGTGGGGCACAGGTGAACCCGGCAGTCACTCTAACTATGTTGGCGACACGGAAGCTG
GATGTCCTCAGGGCGCTTGTATACATGGGTGCCCAGTGTCTGGGGGCCTCTTTAGGGGCT
GGGGCCCTCTACTTGGCCCTGCCACTCAAATCCACCGCAGACTACTTCGTCAACAGGGTG
CCTATAGAGCTGAATGCAGCCCAGGCTCTGGGGATCGAGGTCCTGTGCACCTTCCAGATG
GTCTTCACCGTGTTCTCAGTGGAGGAGCAGCGACGGCGGGAGAGCACGGAGCCGGGAAAC
CTTGCCATCGGACTGGCCCACACTGCTGGAGTCCTAATAGGGGCACGGTTCTCTGGTGCA
AGTATGAACCCTGCTCGCTCTCTGGGTCCAGCCATCATCACCGGCTTCTGGGAAAACCAC
TGGGTATACTGGATAGGGCCTGTGGCAGGTGCAATGCTGGCCGGCATGTCCCACGAGTTC
GTCTTCGCACGCAGCGCGTCCCGGCAGAAGCTGGTGGCGTGTCTGACCTGCAAGGATATC
GAGATCGTGGAGACGGCCAGCGTGTCCCGATCATCGATCTCCACCGTCACTCAGAACGCC
ACGAGGGCCAAGCCGGGCAGCAAA---CAGGAGAACAAC
>MN168419

------------CAGGTGAACCCGGCAGTCACTCTAACTATGTTGGCAACACGGAAGCTG
GATGTTCTCAGGGCGCTTGTATATGTGGGTGCCCAGTGTCTGGGGGCCTCTTTAGGGGCT
GGGGCCCTCTACTTGGCCCTGCCACTCAAATCCACCGCAGACTACTTCGTCAACAGGGTG
CCTATAGAGCTGAATGCAGCCCAGGCTCTGGGGATCGAGGTTTTGTGCACCTTCCAGATG
GTCTTCACTGTGTTCTCAGTGGAGGAACAGCGACGGCGGGAGAGCACAGAGCCGGGAAAC
CTCGCCATCGGACTGGCCCACACTGCTGGAGTCCTAATAGGGGCACGGTTCTCTGGTGCG
AGTATGAACCCTGCTCGCTCTCTGGGTCCAGCCATCATCACCGGCTTCTGGGAAAACCAC
TGGGTATACTGGATAGGGCCCGTGGCAGGTGCAATGCTGGCTGGCATGTCCCATGAGTTC
GTCTTTGCACGCAGCGCATCACGGCAGAAACTGGTGGCGTGTTTGACCTGCAAGGATATC
GAGATCGTGGAGACAGCCAGCGTGTCCCGATCATCAATCTCCACCGTCACTCAGAACGCC
ACGAGAGCCAAGCCGGCCAGCAAA---CAGGAGAACAAC
>MN168420
GAGCTACGCAGTCGGCAGTTCTGGCGTGCCATGCTGGCAGAACTGCTCGGCACCCTGGTG
TTTGTGAGCGCCGTGCTGGGGGCCTCTGTGCCAGGCCCCGGTGAGGCCCCTGGGGGACCC
CTGTACCCAGCCGTGGCAGTGGGTGCGGTGATTGTTTCACTGGGCCACTGTTTTGGAGTG
ATCAGCGGGGCACAGGTGAACCCAGCAGTCACTCTGACTATGTTGGCTACACGCAAGCTG
GATGTTCTCAGGGCACTTGCGTATGTTGGTGCCCAGTGTCTGGGGGCCTCTCTAGGGGCC
GGGGCCCTCTACCTGGCCCTGCCGCTGAAATCCACCGCAGACTACTTTGTCAACAAGGTG
CCTATAGAGCTGAACGCGGCCCAGGCTCTGGGGATCGAGGTTTTGTGCACCTTCCAGATG
GTCTTCACTGTGTTTTCAGTGGAGGAGCAGCGGCGGAGGGAGAGCACAGAGCCAGGAAAC
CTAGCCATTGGATTCGCCCACATTGCTGGTGTCCTAATAGGGGCACGGTTCTCTGGTGCA
AGTATGAACCCTGCACGCTCTCTGGGTCCAGCCATCATCACTGGCTTCTGGGAAAACCAT
TGGGTATACTGGATAGGACCCGTGACAGGTGCCATGCTGGCCGGCATGTCCCATGAGTTC

ATCTTTGCGCGCAGTGCGTCCCGGCAGAAGCTGGTGGCGTGCCTGACCTGTAAGGATATT
GAGATCGTAGAGACAGCCAGTGTGTCCCGATCGTCGATCTCCACCGTCACTCAGAACGCC
ACGAGAGCCAAGCAGGCCAACAAA---CAGGAGAACAAC
>MN168421
GAGCTACGTAGTCGGCAGTTCTGGCGTGCCGTTCTGGCAGAAGTGATCGGCACCCTGGTG
TTTGTGAGTACCGTGCTGGGGGCCTCTGTGCCTGGCCCAGGAGAGGTCCCTGGGGGACCC
CTGTACACAGCCCTGGCAGCGGGTGTGGTGATTGTTGCACTTTGCCACTGTTTTGGAGAG
ATCAGCGGGGCACAGGTGAACCCTGCTGTGACTCTGGCTCTGTTGGCCACACGGAAGATG
GATGTTCTCAGAGCCCTTGTTTATATAGGCGCACAGTGTCTGGGGGCCACTCTAGGGGCT
GGGGCCCTCTACCTGGCCCTGCCACTCAAACCCACTGCAGACTGCTTCGTCACCAGGGTG
CCTATAGAGATTAATGCAGCCCAGGCTCTGGGGATGGAGGTGTTGTGCACCTTCCAGATG
ACCTTCACTGTGTTCTCAGTGGAGGACCAGCGAAGGAGGGAGTGCACAGAACCAGGAAAC
CTGGCCATTGGATTTGCCCACACTACTGGAGTCCTAATAGCGGGACGGTTCTCTGGTGCA
TGTATGAACCCTGCACGCTCTCTGGGTCCAGCCATCATCACTGGTTTCTGGGAAAACCAC
TGGGTATATTGGATAGGGCCAGTGCTAGGTGCAATGCTGGCTGGCGTGTCCCATGAATTC
TTCTTTGCACGCAGCGCATCTCGCCAGAAGCTGGTGGCATGTCTGACCTGTAAGGATATT
GAGATAGTGGAGACAGCCAGTGTATCCCGATCATCACTCTCCACTGTCACTCAGAACGCC
ATGAGAGCCAAGCAGGCTAACAAA---CAGGAGAACAAC
>MN168422
GAGCTACGTACTCGGCAGTTCTGGCGTGCCGCTCTGGCAGAACTGCTCGGCACCCTGGTG
TTTGTGAGTGCCGTGCTGGGGGCCTCTGTGCCTGGCCCGGGAGGGGTCCCTGGGGGACCC
CTGTACACAGCCCTGGCAGCGGGTGTGGCGATTGTTGCACTGTGCCACTGTTTTGGGGAG
ATCAGCGGGGCACAGGTGAACCCTGCAGTGACTCTGACTATGTTGGCCACACGGAAGATG
GATGTTCTCAGAGCCCTTGTTTACATAGGCGCACAGTGTCTGGGGGCCTTTCTAGGTACT
GGGGCCCTCTACCTGGCCCTGCCACTCAAACCCACCGCAGACTGCTTCGTCACCAGGGTG
CCTATAGAGATGAACGCAGCCCAGGCTCTGGGGATGGAGGTGTTGTGCACCTTCCAGATG
GTCTTCACTGTGTTCTCAGTTGGGGACCAGCGGAGGAGGGAGTGCACAGAACCAGGAAAC
CTGGCCATTGGATTTGCCCACACTTCTGGAGTCCTAATAGCGGGACGGTTCTCTGGTGGA
TGTATGAACCCTGCACGCTCTCTGGGTCCAGCCATCATCACTGGTTTCTGGGAAAACCAC
TGGGTATATTGGATAGGGCCAGTGCTAGGTGCAATGCTGGCTGGCATGTCCCATGAATTC
TTCTTTGCACACAGCGCATCTCGCCAGAAGATGGTGGCGTGTCTGACCTGTAAGGATATT
GAAATAGTGGAGACAGCCAGTTTATCCCGATCGTCACTCTCCACTGTCACTCAGAACGCC
ACGAGAGCCAAGCAGGCTAACAAA---CAGGAGAACAAC
>MN168423
GAGCTACGTAGTCGGCAGTTCTGGCGTGCCGTGTTGGCAGAACTGCTCGGCACCCTGGTG
TTAGTGAGCGCCGTGCTGGGTGCCTCTGTGCCCGGCCCTGGAGAGGCCTCTGGGGGACCC
CTGTACCCTGCCCTGGCAATCGGTGTGGTGATTGTCGCTCTGTGCCACTGTTTTGGAGAG
ATCAGTGGGGCGCAGGTGAACCCTGCGGTGACTCTGACTCTGCTGGCCACACGGAGGGTG
GATGTTCTCAGAGCCCTTTTTTATATTGCTGCGCAGTGTGTGGGGGCCTTTCTAGGGACC
GGGGCCCTCTACCTGGCCCTGCCGCTCAAACGCACTGCAGACTCCTTCCTCAACAAGGTG
CCTCTTGACATGAATGCAGCCCAGGCTCTGGGGATGGAGATGCTGTGCACCTTCCAGATG
GTCTTCACTGTGTTCTCGGTGGAGGACCAGCGACGGAGGGAGTGCACAGAACCAGCAAAC
CTGGCTATTGGATTCGCCCACATTGCTGGAGTCCTAATAGCGGGACGGTTCTCTGGAGCG
AGTATGAACCCTGCACGCTCTCTGGCTCCAGCCATCATCATTGGTTTCTGGGAAAACCAC
TGGGTATATTGGATAGGGCCGGTGCTAGGTGCAGTACTGGCAGGCGTGTCCCATGAATTC
TTTTTTGCACGCAGCGCATCCCGCCAGAAGCTTGTGGCTTGTCTGACCTGTAAGGATATT
GAGATAGTGGAGACGGCCAGTGTGTCCCGGTCATCGCTCTCCACTGTCACTCAGAACGCC
ATGAGAGCCAAGCAGGCTAACAAA---CAGGAGAACAAC
>MN168424
GAGCTGCGTAGTCGGCAATTCTGGCGTGCCATACTGGCAGAAGTGCTCGGCACCCTGGTG
TTTGTGAGTGCCGTGCTGGGGGCCTCTGTGCCAGGCCCTGGAGAGGCCCCTGGGGGACCC

CTGTACCCAGCCCTGGCAGCGGGTGTGGTGATTGTTGCACTGGGCCACTGTTTTGGAGAG
ATCAGTGGGGCACAGGTGAACCCTGCAGTGACTCTGTCCCTGTTGGCCACACGGAGGCTG
GATGTTCTCAGGGCCCTTGTTTATGTTGGTGCACAATGTCTGGGTGCCTCTCTAGGGGCT
GGAGCCCTCTACCTGGCCCTGCCACTCAAAGCCACTGCAAACTTCTTTGTCAACAGGGTG
CCTATAGAGGTGAATGCAGCCCAGGCTCTGGGGATGGAGGTTTTGTGCACCTTCCAGTTG
GTCTTCACTATATTCTCAGTGGAGGACCAGCGACGGAGGGAGAGCACAGAACCAGGAAAC
CTGGCCATTGGATTTGCCCACAGTGCTGGAGTTCTGATAGGGGCACGGTTCTCTGGTGCA
AGTATGAACCCTGCACGCTCTCTGGGTCCAGCCATCATCACTGGCTTCTGGGAAAACCAC
TGGGTATATTGGATAGGACCAGTGCTAGGTGCAATGCTGGCTGGTGTTTCCCATGAGTTC
TTCTTTGCACGCAGCGCATCTCGCCAGAAGCTGGTGGCATGTCTGACCTGTAAGGATATT
GAGATAGTGGAGACAGCCAGTGTATCCCGATCATCACTCTCCACTGTCACGCAGAACGCC
ATGAGAGCCAAACAGGAGAACAAC---------------
>MN168425
GAGCTACGCAGTCGGCAGTTCTGGCGGGCCATGCTGGCAGAGCTGGTGGGCACCCTGCTG
TTTGTGAGCGCCATACTTGGGGCCTCCATGCCCGGCCCTGGAGAGGCCCCTGGGGGACCC
ATCTATCCAGCAATTGCTGCAGGTGTGACCATTGTTGCTCTGTGCCATTGTTTCGGAGAG
ATCAGCGGCACACAGGTGAACCCTGCAGTGACTCTGTCATTCCTGGCTACACGGAAGCTG
GATGCCCTTAGGGCCCTGGTGTATGTGGGTGCACAGTGTCTGGGGGCCTCTCTGGGGGCC
GGGGCCCTCTACCTGGCACTACCGGTCCAAACCTCCCCAGAGCACTTCGTAACCAGAGTT
CCTATGGAGGTGAATGTGGCCCAGGCTCTGGGGATGGAGGTTCTGTGTACCTTCCAGATG
ACCTTCACCGTATATTCGGTGGAGGAGCAGCGACGCAGGGAGGGCACCGAGCCGGGGAAC
CTAGCCATCGGGTTCGCCCACACCGCCGGAGGACTTATGGCGGCGCGGTTCTCTGGAGCA
TGTATGAACCCAGCACGCTCTCTGGGCCCAGCCATCATCACTGGCTACTGGGAAAACCAC
TGGGTATACTGGATTGGGCCGGTGCTGGGTGCCCTGCTTGCCGGAGTCTCACATGAGTTC
TTCTTTGCCAACAGCGCATCTCGCCAGAAGCTGGTGGCTTGTCTGACCTGCAAGGATATC
GAGATTGTGGAGACGGCCAGTGTGTCCCGCTCATCACTGTCAACTGTCACGCAGAATGCC
ATGAGAGCCAAACAGGCTAGCAAG---CAGGACAACAAT
>MN168426
GAGCTACGCAGTCGGCAATTCTGGCGCGCCATGCTGGCAGAACTGCTGGGCACCCTGGTG
TTTGTGAGTGCCATACTTGGGGCCTCTGTGCCCGGCCCCACAGAGGCCCCCGGAGGACCT
ATCTATCCAGCCCTGGCTGCTGGTGTGACCATCGTTGCCGTGTGCCATTGTTTTGGAGAG
ATCAGCGGTGCACAGGTGAACCCTGCAGTAACTCTGTCATTCCTGGCCACACGGAAGCTG
GATGCCCTCAGGGCCCTGGTGTATGTGGGTGCCCAATGTCTGGGGGCCTCTCTGGGGGCC
GGGGCCCTCTACGTGGCACTACCAGTCAAAACCACCGCAGAGCACTTCATAACCAGAGTT
CCTATGGAGGTGAACGCGGCCCAGGCTCTGGGGATGGAGATCCTGTGTACCTTCCAGATG
GCCTTCACTGTGTATTCGGTGGAGGACCAGCGACGCAAGGAGGGCACCGAGCCGGGCAAC
CTGGCCATCGGGTTCGCCCACACCGCCGGAGTACTGATGGCGGCACGGTTCTCTGGAGGA
TGTATGAACCCTGCACGCTCTCTGGGCCCAGCCATCATCACTGGCTTCTGGGAAAACCAC
TGGGTA--

>MN168427
GAGCTCCGTAGCCGGCAGTTCTGGCGTGCCATGCTTGCAGAACTGCTGGGCACCCTGCTG
TTAGTGAGTACCGTTTTAGGGGCCTCTGTGCCAGGCCCTGAAGAGGCCCCTGTGGGACCC
CTGTACCCGGCCCTGGCAGCAGGGGTGGTCATAGTTGCACTGTGCCACTGTTTTGGAGAG
ATCAGCGGGACTCAGGTGAACCCTGCTGTGACTCTGACCATGATGGCTACAAGGAGAATA
GACGTGCTCCGGGGTGTGGCCTACGTTAGCGCACAGTGTATGGGAGGCTGTCTGGGAGCC
GGGGCCCTCTACCTGGCCCTGCCACTCAAATCCACCGCAGACCACTTTGTTAACAAGGTG
CCTCTGGAGGTGAACGCAGCCCAGGCTCTGGGCATGGAGGTGCTGTGCACCTTCCAGTTG
GTCTTCACTGTGTACGCGGTGGAGGACCAGAGGCGGAGGGGGAGCACAGACACAGGGAAC

CTGGCCATTGGATTCGCTCACACCACTGGAGTTCTGATGGCGGCAAGATTCTCTGGGGGA
TGTATGAATCCTGCACGTGCTCTGGGTCCAGCCGTCATCGTTGGCTTCTGGGAAAACCAC
TGGGTATTTTGGATCGGCCCGGTGATCGGCGCCATGCTGGCCGGCGTCTCCCACGAGTTC
TTCTTTGCAGCGGCGGCGTCTCGCCACAAGCTGGTGGCCTGTCTGACCTGCAAGGACATC
GAGATGGTGGAGACGGCCAGCGTGTCGCACTCCTCACTGTCCACGGCCACCCAGAATGCC
ATGAGAGCCAAGCAGGCTAACAAG---CAGGACAACAAC
>MN168428
GAGCTCCGCAGTCGACAGTTTTGGCGTGCCGTGCTGGCAGAGGTGCTGGGCACCCTGGTG
CTCGTGAGCACCATGCTCGGGGCCTCTGTGCCGGGTCCAGGAGAGGCCCCCGGGGGCCCA
CTGTACCCAGCCCTGGCAATCGCTGCCGTGATCACCTCGCTGGGTCACTGTTTCGGAGAG
ATCAGCGGGGCGCAGGTGAACCCTTCAGTGACTCTCTCATTTTTGGCCGCAAAGAAGATA
GACCTGGTCAGGGCCCTTGTTTATGTGGCCGCACAGTGCCTGGGGGCCTTTCTGGCCAGC
GGGGCCCTCTACCTGGCCCTCCCGCACAAATCCACTGCGGAGGTGTTCGTCAATCGGGTC
GGCCCCGACGTCAACGCGGCACAGGCGGTGGGCGTGGAGGTGCTCTGCACCTTCTCCATG
GTCTTCGCCATCTTCTCTGTGGAGGAGCAGAGGCGGAGAGAGAGCACCGAGCCCAGCCAC
CTGGGCATCGGACTGGCCCACACGGCCGCAGTGTTTATAGGGGCGAGGTTTTCTGGTGCA
GGAATGAATCCGGCTCGTGCTCTGGGTCCAGCTCTCATCACCGGCTACTGGGAGAACCAC
TGGGTATACTGGATCGGGCCGGTGACGGGCGGTGTGCTGGCCGGCGTGTCACACGAGTTT
TTCTTCGCCCACAGCGCCTCCCGCCTGAAGCTGGTGGCGTGTCTGACCTGTAAGGACATC
GACATGGTGGAGACTGCCAGCATGTCCCGCTCCTCCCTCTCCACCGTCACGCAGAACGCC
AACCGGGCCAAGCAGGCCAACAAG---GGCGAGGGCACC
>MN168429
---------AGTCGACAGTTTTGGCGTGCCGTGCTGGCAGAGGTGCTGGGCACCCTGGTG
CTCGTGAGCACCATGCTCGGGGCCTCTGTGCCGGGTCCGGGAGAGGCCCCCGGGGGCCCC
CTGTACCCAGCCCTGGCAATCGCTGCCGTGATCACCTCGCTGGGTCACTGTTTTGGAGAG
ATCAGCGGGGCACAGGTGAACCCTTCAGTGACTCTGTCATTTTTGGCCGCAAAGAAGATA
GACCTGGTCAGGGCCCTTGCTTATGTGGCCGCACAGTGCCTGGGGGCCTTTCTGGCCAGC
GGGGCCCTCTACCTGGCCCTCCCGCACAAATCCACTGCGGAGGTGTTCGTCAATCGGGTC
GGCCCCGACGTCAACGCGGCACAGGCGGTGGGCGTGGAGGTGCTCTGCACCTTCTCCATG
GTCTTCGCCATCTTCTCTGTGGAGGAGCAGAGGCGGAGAGAGAGCACCGAGCCCAGCCAC
CTGGGCATCGGACTGGCCCACACGGCCGCAGTGTTTATAGGGGCGAGGTTTTCTGGTGCA
GGAATGAATCCGGCTCGTGCTCTGGGTCCAGCTCTCATCACCGGCTACTGGGAGAACCAC
TGGGTATACTGGATCGGGCCGGTGACGGGCGGCGTGCTGGCCGGCGTGTCACACGAGTTC
TTCTTCGCCCACAGCGCCTCCCGCCTGAAGCTGGTGGCGTGTCTGACCTGTAAGGACATC
GACATGGTGGAGACGGCCAGCATGTCCCGCTCCTCCCTCTCCACCGTCACGCAGAACGCC
AACCGGGCCAAGCAGGCCAACAAG---GCCGAGGGCAGC
>MN168430
------CGCAGTCGACAGTTTTGGCGTGCGGTGCTGGCAGAGGTGCTGGGCACCCTGGTG
CTCGTGAGCACCATGCTCGGGGCCTCTGTGCCGGGTCCGGGAGAGGCCCCCGGGGGCCCG
CTGTACCCAGCCCTGGCAATCGCTGCCGTGATCACCTCGCTGGGCCACTGTTTCGGAGAG
ATCAGCGGGGCACAGGTGAACCCTTCAGTGACTCTGTCATTTTTGGCCGCAAAGAAGATA
GACCTGGTCAGGGCCCTTGCTTATGTGGCCGCACAGTGCCTGGGGGCCTTTCTGGCCAGC
GGGGCCCTCTACCTGGCCCTCCCGCACAAATCCACTGCGGAGGTGTTCGTCAATCGGGTC
GGCCCCGACGTCAACGCGGCACAGGCGGTGGGCGTGGAGGTGCTCTGCACCTTCTCCATG
GTCTTCGCCATCTTCTCTGTGGAGGAGCAGAGGCGGAGAGAGAGCACCGAGCCCAGCCAC
CTGGGCATCGGACTGGCCCACACGGCCGCAGTGTTTATAGGGGCGAGGTTTTCTGGTGCA
GGAATGAATCCGGCTCGTGCTCTGGGTCCAGCTCTCATCACCGGCTACTGGGAGAACCAC
TGGGTATACTGGATCGGGCCGGTGACGGGCGGCGTGCTGGCCGGCGTGTCACACGAGTTC
TTCTTCGCCCACAGCGCCTCCCGCCAGAAGCTGGTGGCGTGTCTGACCTGTAAGGACATC
GACATGGTGGAGACGGCCAGCGTGTCCCGCTCCTCCCTCTCCACCGTCACGCAGAACGCC
AACCGGGCCAAGCAGGCCAACAAG---GCCGAGGGCAGC

>MN168431
---CTCCGCAGTCGACAGTTTTGGCGTGCGGTGCTGGCAGAGGTGCTGGGCACCCTGGTG
CTCGTGAGCACCATGCTCGGGGCCTCTGTGCCGGGTCCGGGAGAGGCCCCCGGGGGCCCG
CTGTACCCAGCCCTGGCAATCGCTGCCGTGATCACCTCGCTGGGTCACTGTTTTGGAGAA
ATCAGCGGGGCACAGGTGAACCCTTCGGTGACTCTGTCATTCTTGGCCGCAAAGAAGATA
GACCTGGTCAGGGCCCTTGTTTATGTGGCCGCACAGTGCCTGGGGGCCTTTCTCGCCAGC
GGGGCCCTCTACCTGGCCCTCCCGCACAAATCCACTGCGGAAGTGTTTGTCAATCGGGTC
GGCCCCGACGTCAACGCGGCACAGGCGGTGGGCATGGAGGTGCTCTGCACCTTCTCCATG
GTCTTCGCCATCTTCTCTGTGGAGGAGCAGAGGCGGAGAGAGAGCACAGAGCCCAGCCAC
CTGGGCATCGGACTGGCCCACACGGCCGCAGTGTTTATAGGGGCGAGGTTTTCGGGTGCA
GGAATGAATCCGGCCCGTGCTCTGGGTCCAGCTCTCATCACCGGCTACTGGGAGAACCAC
TGGGTATACTGGATCGGGCCGGTGACGGGCGGCGTGCTGGCCGGCGTGTCACACGAGTTC
TTCTTCGCCCACAGCGCCTCCCGCCAGAAGCTGGTGGCGTGTCTGACCTGTAAGGACATC
GATATGGTGGAGACGGCCAGTATGTCCCGCTCCTCCCTCTCCACCGTCACGCAGAACGCC
AACCGGGCCAAGCAGGCCAACAAG---GGCGAGGGCAAC
>MN168432
GAGCTCCGCAGTCGACAGTTTTGGCGTGCGGTGCTGGCAGAGGTGCTGGGCACCCTGGTG
CTCGTGAGCACCATGCTCGGGGCCTCTGTGCCGGGTCCGGGAGAGGCCCCCGGGGGCCCG
CTGTACCCAGCCCTGGCAATCGCTGCCGTGATCACCTCGCTGGGTCACTGTTTTGGAGAG
ATCAGCGGGGCACAGGTGAACCCTTCTGTGACTCTGTCATTCTTGGCCGCAAAGAAGATA
GACCTGGTCAGGGCCCTTGTGTATGTGGCCGCACAGTGCCTGGGGGCCTTCCTCGCCAGC
GGGGCCCTCTACCTGGCCCTCCCGCACAAATCCACTGCGGAAGTGTTTGTCAATCGGGTC
GGCCCCGACGTCAACGCGGCACAGGCGGTGGGCATGGAGGTGCTCTGCACCTTCTCCATG
GTCTTCGCCATCTTCTCTGTGGAGGAGCAGAGGCGGAGGGAGAGCACAGAGCCCAGCCAC
CTGGGCATCGGACTGGCCCACACGGCCGCCGTGTTTATAGGGGCGAGGTTTTCTGGTGCA
GGAATGAACCCGGCTCGTGCTCTGGGTCCAGCTCTCATCACCGGCTACTGGGAGAACCAC
TGGGTATACTGGATCGGGCCGGTGACGGGCGGCGTGCTGGCCGGCGTGTCACACGAGTTC
TTCTTCGCCCACAGCGCCTCCCGCCAGAAGCTGGTGGCGTGCCTGACCTGTAAGGACATC
GACATGGTGGAGACGGCCAGTATGTCCCGCTCCTCCCTCTCCACCGTCACGCAGAATGCC
AACCGGGCCAAGCAGGCCAACAAG---GGCGAGGGCAAC
>MN168433
---CTCCGCAGTCGACAGTTTTGGCGTGCGGTGCTGGCGGAGGTGCTGGGCACCCTGGTG
CTCGTGAGCACAATGCTCGGGGCCTCTGTGCCGGGTCCGGGAGAGGCCCCAGGGGGCCCG
CTGTACCCAGCCCTGGCAATCGCTGCCGTGATCACCTCGCTGGGTCACTGTTTTGGAGAG
ATCAGCGGGGCACAGGTGAACCCTTCAGTGACTCTGTCATTTTTGGCCGCAAAGAAGATA
GACCTGGTCAGGGCCCTTATGTATGTGGCCGCGCAGTGCCTGGGGGCCTTTCTCGCCAGC
GGGGCCCTCTACCTGGCCCTCCCGCACAAGTCCACTGCGGAAGTGTTTGTCAATCGGGTC
GGCCCCGACGTCAACGCGGCGCAGGCGGTGGGCATGGAGGTGCTCTGCACCTTCTCTATG
GTCTTCGCCATCTTCTCTGTGGAGGAGCAGAGGCGGAGAGACAGCACAGAGCCCAGTCAC
CTGGGCATCGGGCTGGCCCACACGGCCGCAGTGTTTATAGGGGCGAGGTTTACTGGTGCA
GGAATGAATCCGGCTCGTGCTCTGGGTCCAGCTCTCATCACCGGCTACTGGGAGAACCAC
TGGGTATACTGGATCGGGCCGGTGACGGGCGGCGTGCTGGCCGCCGTGTCACACGAGTTC
TTCTTCGCCCACAGCGCCTCCCGCCAGAAGCTGGTGGCGTGTCTGACCTGTAAGGACATC
GACATGGTGGAGACGGCCAGCATGTCGCGCTCCTCCCTCTCCACCGTCACGCAGAACGCC
AACCGGGCCAAGCAGGCCAACAAG---GGCGAGGGCAAC
>MN168434
GAGCTCCGCAGTCGACAGTTTTGGCGTGCGGTGCTGGCAGAGGTGCTGGGCACCCTGGTG
CTCGTGAGCACCATGCTCGGGGCCTCTGTGCCGGGTCCGGGAGAGGCCCCAGGGGGCCCA
TTGTACCCAGCCCTGGCAATAGCTGCCGTGATCACTTCGCTGGGTCACTGTTTTGGAGAG
ATCAGCGGGGCACAGGTGAACCCTTCAGTGACTCTGTCATTTTTGGCCGCAAAGAAGATA
GACCTGGTCAGGGCCCTTGTGTACGTGGCCGCACAGTGCCTGGGGGCCTTTCTCGCCACC

GGGGCCCTCTACCTGGCCCTCCCGCACAAATCCACTGCCGAAGTGTTTGTCAATAGGGTC
GGCCCCAATGTCAACGCAGCACAGGCGGTGGGCATGGAGGTGCTCTGCACCTTCTCCATG
GTCTTTGCCATCTTCTCTGTGGAGGAACAGCGGCGGCGTGAGAGCACGGAGCCCAGCCAC
CTGGGCATCGGATTGGCCCACACGGCTGCAGTGTTTATAGGGGCGAGGTTTTCTGGTGCA
GGAATGAATCCGGCTCGTGCTCTGGGTCCAGCGCTCATT---------------------
---GTGTACTGGATCGGGCCGGTGACGGGCGGCGTGCTGGCCGGCGTGTCCCACGAGTTC
TTCTTTGCCCACAGCGCCTCCCGCCAGAAGCTGGTGGCGTGTCTGACCTGTAAGGACATC
GACATGGTGGAGACGGCCAGCATGTCCCGCTCCTCCCTCTCCACCGTCACGCAGAATGCC
AACCGGGCCAAGCAGGCCAATAAG---GGAGAGGGCAAC
>MN168435

------------------------------ATCACGTCGCTGGGTCACTGTTTTGGAGAG
ATCAGCGGGGCACAGGTGAACCCTTCGGTGACTCTGTCGTTTTTGGCCGCAAAGAAGATC
GACCTGGTCAGGGCCCTTGTGTACGTTGCCGCCCAGTGCCTGGGGGCCTTTCTCGCCACC
GGGGCCCTCTACCTGGCCCTCCCGCACAAATCCACCGCCGATGTCTTTGTCAATAGGGTG
GGCCCTGGTGTCAACGCAGCGCAGGCGGTGGGCATGGAGGTGCTCTGCACCTTCTCCATG
GTCTTCGCCATCTTCTCCGTGGAGGAGCAGCGGCGGAGGGAGAGCACGGAGCCCAGCCAC
CTGGGCATCGGACTGGCCCACACAGCCGCAGTGTTTATAGGGGCGAGGTTTTCTGGTGCA
GGAATGAATCCTGCTCGCGCCTTGGGTCCAGCTCTAATCACCGGC---------------
---GTGTACTGGATCGGGCCGGTGACGGGCGGCGTGCTGGCCGGCGTGTCCCACGAGTTC
TTCTTCGCCCACAGCGCATCGCGCCAGAAGCTGGTGGCGTGTCTGACCTGCAAGGACATC
GACATGGTGGAGACGGCCAGCATGTCCCGCTCCTCCCTCTCCACCGTCACGCAGAACGCC
AACCGGGCCAAGCAGGCCAACAAG---GGCGAGGGCAAC
>MN168436
GAGCTACGCAGTCGACAGTTTTGGCGTGCAGTGCTGGCAGAGGTGCTGGGCACCCTGGTG
CTCGTGAGTACCATGCTTGGGGCCTCTGTGCCTGGTCCGGGAGAGGCCCCTGGGGGCCCC
TTGTACCCAGCCCTGGCAATAGCTGCCGTGATCGTTTCACTGGGTCACTGTTTTGGAGAG
ATCAGCGGGGCACAGGTGAACCCTTCAGTGACTCTGTCACTGTTGGCCACAAAGAAGATA
GACCTTGTCAGGGCCCTTGTGTATGTAGCCGCACAGTGCCTGGGGGCCTTTCTCGCCACC
GGGGCCCTCTACCTGGCCCTCCCGCGGAAATCCACTGCGGAAGTCTTTGTCAATAGGGTG
GCCCCAGACGTCAACGCAGCGCAGGCGGTGGGCATGGAGGTGCTCTGCACCTTCTCCATG
GTCTTCACCATCTTCTCAGTGGAGGAGCAGAGGCGGAAAGAGAGCACAGAGCCTGGCAAC
CTGGGCATCGGATTGGCCCACACGGCTGGAGTGTTTATAGCGGCAAGGTTTTCTGGTGCA
GGAATGAATCCGGCTCGTGCTCTGGGTCCAGCCATCATCACTGGCTTCTGGGAGAACCAC
TGGGTATACTGGATCGGACCGGTGACGGGCGGCGTGCTGGCCGGCGTCTCCCACGAGTTC
TTCTTCGCCCACAGCGCCTCGCGCCAGAAGCTGGTGGCGTGTCTGACCTGTAAGGACATA
GACATGGTGGAGACGGCCAGCATGTCCCGCTCCTCCCTCTCCACCGTCACCCAGAACGCC
AACCGGGCCAAGCAGGCCAACAAG---GGGGAGGGCAAC
>MN168437
GAGCTACGCACTCGACAGTTTTGGCGTGCAGTGTTGGCAGAGGTGCTGGGCACCCTGGTG
CTCGTGAGTACCATGCTCGGGGCCTCTGTGCCTGGTCCGGGAGAGGCCCCTGGGGGCCCC
TTGTACCCAGCCCTGGCAATAGCTGCCGTGATCGTTTCGCTGGGTCACTGTTTTGGAGAG
ATCAGCGGGGCACAGGTGAACCCTTCGGTGACTCTGTCACTGTTGGCCACAAAGAAGATA
GACCTTGTCAGGGCCCTTGTGTATGTAGCCGCACAGTGCCTGGGGGCCTTTCTCGCCAGC
GGGGCCCTCTACCTGGCCCTCCCGCGGAAATCCACTGCGGAAGTCTTTGTCAATAGGGTG
CCCCCAGACGTCAACGCAGCGCAGGCGGTGGGCATGGAGGTGCTCTGCACCTTCTCCATG
GTCTTCACCATCTTCTCTGTGGAGGAACAGAGGCGGAGAGAGAGCACAGAGCCCGGCAAC
CTGGGCATCGGATTGGCCCACACGGCTGCAGTGTTTATAGCGGCGAGGTTTTCTGGTGCA
GGAATGAATCCGGCTCGTGCTCTGGGTCCTGCCATCATCACTGGCTTCTGGGAGAACCAC
TGGGTGTACTGGATCGGGCCGGTGACGGGCGGCGTGCTGGCCGGCGTCTCCCACGAGTTC

TTCTTCGCCCACAGCGCCTCGCGCCAGAAGCTGGTGGCGTGTCTGACCTGTAAGGACATA
GACATGGTGGAGACGGCCAGCATGTCCCGCTCCTCGCTCTCCACCGTCACCCAGAACGCC
AACCGGGCCAAGCAGGCCAACAAG---GGGGAGGGCAAC
>MN168438
GAGCTACGCAGTCGACAGTTTTGGCGTGCAATGTTAGCAGAGCTGCTGGGCACCCTGGTG
CTCGTGAGTACCATGCTCGGGGCCTCTGTGCCTGGTCCGGGAGAGGCCCCTGGGGGCCCC
CTGTACCCAGCCCTGGCAATAGCTGCCGTGATCGTTTCGCTGGGTCACTGTTTTGGAGAG
ATCAGCGGGGCACAGGTGAACCCTGCAGTGACTCTATCACTGTTGGCCACAAAGAAGATA
GACCTTGTCAGGGCCCTTGTGTATATAGCCGCACAGTGCCTGGGGGCCTTTCTCGCCACC
GGGGCCCTCTACCTGGCCCTCCCGCGGAAATCCACTGCGGAAGTCTTTGTTAATAGGGTG
CCCCCAGACGTCAACGCAGCGCAGGCGGTGGGCATGGAGGTGCTCTGCACCTTCTCCATG
GTCTTCACCATCTTCTCTGTGGAGGAACAGAGGCGGAGAGAGAGTACAGAGCCTGGCAAC
CTGGGCATTGGATTGGCCCACACGGCTGCAGTGTTTATAGCGGCGAGGTTTTCTGGTGCA
GGAATGAATCCGGCTCGTGCTCTGGGTCCAGCCATCATCACTGGCTTCTGGGAGAACCAC
TGGGTATACTGGATCGGGCCGGTGACGGGCGGCGTGCTGGCCGGCGTCTCCCACGAGTTC
TTCTTCGCCCACAGCGCCTCGCGCCAGAAGCTGGTGGCGTGTCTGACCTGTAAGGACATA
GACATGGTGGAGACGGCCAGCATGTCCCGCTCCTCCCTCTCCACCGTCACCCAGAACGCC
AACCGGTCCAAGCAGGCCAACAAG---GGGGAGGGCAAC
>MN168439
GAGCTACGCAGTCGACAGTTTTGGCGTGCAATTTTGGCAGAACTGCTGGGCACCCTGGTG
CTCGTGAGCACCATGCTCGGGGCCTCTGTGCCGGGTCCGGGAGAGCCCCCAGGGGGCCCC
CTGTACCCAGCGCTGGCAATAGCTGCCGTGATCGTTACGCTGGGTCACTGTTTTGGAGAG
ATCAGCGGGGCACAGGTGAATCCTGCGGTGACTCTGTCATTCTTGGCCACAAAGAAGATG
GACCTCGTCAAGGCCCTGGTGTATGTAAGCGCACAGTGCCTGGGCGCCTTTCTCGCCACC
GGGGCCCTGTACCTGGCCCTCCCGCGGAAATCCACTGCGGAAGTCTTTGTCAATAGGGTG
CCCGCGGACGGCAACGCTGCGCAGGCCGTGGGCATGGAGGTGTTGTGCACCTTCTCCATG
GTCTTCACCATCTTCTCCGTGGAGGAACAGAGGCGGAGAGAGAGCACAGAGCCTGGCAAC
CTGGGCATCGGATTGGCCCACACGGCTTCAGTGATGATAGCGGCGAGGTTTTCTGGTGCG
GGAATGAATCCGGCTCGTGCTCTGGGTCCCGCCATCATCACTGGCTTCTGGGAGAACCAC
TGGGTATACTGGATCGGGCCGGTGACAGGCGGCGTGCTGGCTGGCGTCTCCCACGAGTTC
TTCTTCGCCCACAGCGCCTCACGCCAGAAGCTGGTGGCCTGTCTGACCTGCAAGGACATC
GACATGGTGGAGACCGCCAGCATGTCCCGTTCCTCCCTCTCCACCGTCACCCAGAACGCC
ACCAGGGCCAAGCAGGCCAACAAG---GGGGAGAGCAGC
>MN168440
GAGCTACGCAGTCGACAGTTTTGGCGTGCAATGTTGGCAGAACTGCTGGGCACCCTGGTG
CTCGTGAGCACCATGCTCGGGGCCTCTGTGCCTGGTCCGGGAGAGGCCCCTGGGGGGCCC
CTGTACCCAGCCATTGCAATAGCTGCCGTGATCGTTACGCTGGGTCACTGTTTTGGAGAG
ATCAGCGGGGCACAGGTGAACCCTGCGGTGACTCTGTCATTTTTGGCCACAAAGAAGATA
GACCTTGTCAGGGCCCTGGTGTATGTAGGCGCACAGTGCCTGGGGGCCTTTCTCGCCACC
GGGGCCCTTTACCTGGCCCTCCCGCGGAAATCCACTGCGGAAGTCTTTGTCAATAGGGTG
CCTGTGGACGGCAACGCAGCGCAGGCGGTGGGCATGGAGATGCTGTGCACCTTCTCCATG
GTCTTCACCATCTTCTCCGTGGAGGAACAGAGGCGGAGAGAGAGCACCGAGCCTGGCAAC
CTGGGCATTGGATTGGCCCACACGGCTTCAGTGATGATAGCGGCGAGGTTTTCTGGTGCG
GGAATGAATCCAGCTCGTGCTCTGGGTCCAGCCATCATCACTGGCTTCTGGGAGAACCAC
TGGGTATACTGGATCGGGCCGGTGACGGGCGGTGTGCTGGCCGGCGTCTCCCACGAGTTC
TTCTTCGCCCACAGCGCCTCACGCCAGAAGCTGGTGGCCTGTCTGACCTGTAAGGACATA
GACATGGTGGAGACGGCCAGCATGTCCCGCTCTTCTCTCTCCACTGTCACACAGAACGCC
ACCAGGGCCAAACAGGCCAACAAG---GGGGAGAGCAAC
>MN168441
GAGCTACGCAGTCGACAGTTTTGGCGTGCAATGTTGGCAGAACTGCTGGGCACCCTGGTG
CTCGTGAGCACCATGCTCGGGGCCTCTGTGCCTGGTCCGGGAGAGGCCCCTGGGGGGCCC

CTGTACCCAGCCCTGGCAATAGCTGCCGTGATCGTTACGCTGGGTCACTGTTTTGGAGAG
ATCAGCGGGGCACAGGTGAACCCTGCGGTGACTCTGTCATTTTTGGCCACAAAGAAGATA
GACCTCGTCAGGGCCCTGGTGTATGTAGGCGCACAGTGCCTGGGGGCCTTTCTCGCCACC
GGGGCCCTCTACCTGGCCCTCCCGCGGAAATCCACTGCGGAAGTCTTTGTCAATAGGGTG
CCTGTGGATGGCAACGCAGCGCAGGCGGTGGGCATGGAGATGCTGTGCACCTTCTCCATG
GTCTTCACCATCTTCTCCGTGGAGGAACAGAGGCGGAGAGAGAGCACCGAGCCTGGCAAC
CTGGGCATTGGATTGGCCCACACGGCTTCAGTGATGATAGCGGCGAGGTTTTCTGGTGCG
GGAATGAATCCAGCTCGTGCTCTGGGTCCAGCCATCATCACTGGCTTCTGGGAGAACCAC
TGGGTATACTGGATCGGGCCGGTGACGGGCGGTGTGCTGGCCGGCGTCTCCCACGAGTTC
TTCTTCGCCCACAGCGCCTCGCGCCAGAAGCTGGTGGCCTGTCTGACCTGTAAGGACATA
GACATGGTGGAGACGGCCAGCATGTCCCGCTCTTCTCTCTCCACGGTCACGCAGAACGCC
ACCAGGGCCAAACAGACCAACAAG---GGGGAGAGCAAC
>MN168442
GAGCTACGGAGTCGACAGTTCTGGCGCGCCGTGTTGGCAGAACTGCTGGGCACCCTGGTG
CTCGTGAGCACCATGCTGGGGGCCTCCGTGCCCGGTCCGGGGGAGCCCCCGGGGGCCCG
CTGTACCCCGGTCTGGCGATATGCGCCGTGATCGTGACCCTCGGCCACTGCTTCGGCGAG
ATTAGCGGGGCGCAGGTGAACCCTTCGGTGACCCTGTCCATGCTGGCCACCAGGAAGGTG
GACCTGGTGCGCGCCCTGGTGTACGTCGGGGCCCAGTGTCTGGGGGCCTTCCTGGCCACC
GGCGCCCTGTACCTCGCCCTCCCGCGCAAATCCACCGCCGAAGTCTTTGTCAATCGGGTG

--GCGAGGTTCTCTGGCGCG
GGAATGAATCCAGCCCGTGCCCTGGGCCCAGCCATCATCACTGGCTTCTGGGAGAACCAC
TGGGTATACTGGATCGGCCCGGTGACGGGTGGCGTGCTCGCCGGCGTCTCCCACGAGTTC
CTCTTCGCCCACAGCGCCTCGCGCCAGAAGCTGGTGGCCTGCCTGACCTGCAAGGACGTC
GACATGGTGGAGACGGCCAGCGTGTCGCGCTCCTCACTGTCCACCGTCACGCAGAACGCC
GGCCGCGCCAAGCAGGCCAACAAG---GCCGACGGAAAC
>MN168443
GAGCTCCGGAGTCGGCAGTTTTGGCGTGCGGTGTTGGCAGAACTGCTGGGCACCCTGGTG
CTCGTGAGCACCATGCTGGGGGCCTCCGTGCCGGGTCCGGGGGAGCCCCCTGGGGGCCCG
CTGTACCCGGGCCTGGCCATCTTCGCCGTGATCGTTACGCTGGGACACTGCTTCGGAGAG
ATCAGCGGTGCTCAGGTGAACCCCTCGGTGACTGTGTCCATGTTGGCCGCCAGGAAGCTG
GACCTGGTGAGGGGCCTGGTGTACATGGGGGCCCAGTGTTTGGGGGCCTTCCTGGCCACT
GGGGCCCTGTACCTCGCCCTGCCGCGGAAATCCACTGCCGAAGTCTTCGTCAATAGGGTG
CCGGAGGACGGCAACGCGGCGCAGGCGGTGGGCATGGAGGTCCTGTGCACCTTCTCCATG
GTCTTCACCATCCACGCCGTGCAGGAGCAGCGGCAACGGGACTGCACCGAGCCGGGCCAC
CTGGGCATCGGCCTCGCCCACACCGCCTCCGTGATGATGGCGTCGAGGTTCTCTGGCGCG
GGGATGAATCCAGCTCGGGCTCTGGGTCCGGCCATCATCACTGGCTTCTGGGAGAACCAC
TGGGTATACTGGATCGGTCCCTTGTCGGGCGGGGTGCTCGCCGGCGTGTCCCACGAGTTC
TTGTTCGCTCCCAGCGCGTCGCGCCAGCGGCTGGTGGCGTGTCTCACCTGTAAAGACGTG
GACATGGTGGAGACGGCCAGCGTGTCTCGCTCCTCGCTGTCCACCGTCACGCAGAACGCC
GGCCGAGCCAAGCCGGCCAACAAG---GCCGACGCCAAC
>MN168444
GAGCTCCGGAGTCGGCAGTTTTGGCGTGCGGTGTTGGCAGAACTGCTGGGCACCCTGGTG
CTCGTCAGCACCATGCTGGGGGCCTCCGTGCCGGGTCCCGGGGAGCCCCCTGGGGGCCCG
CTGTACCCTGGGCTGGCGATATGCGCCGTGATCGTTACGCTGGGACACTGCTTCGGAGAG
ATCAGCGGGGCTCAGGTGAACCCCTCGGTGACTCTGTCCCTGTTGGCCGCCAGGAAGTTG
GATCTTGTGAGGGCCCTGGTGTATGTGGGGGCCCAGTGTTTGGGGGCCTTCCTGGCCACC
GGGGCCCTGTACCTCGCCCTGCCGCGGAAATCCACCGCCGAAGTCTTCGTCAATAGGGTC
---GCGGACGGCAACGCGGCGCAGGCGGTGGGCATGGAGGTCCTGTGCACCTTCTCCATG
GTCTTCACGATCTTCGCCGTCGAGGAGCAGCGACGGCGGGACTGCACCGAGCCGGGCAAC

CTGGGCATCGGCCTGGCC---ACGGCCTCCGTGATGATGGTGGCGAGGTTCTCTGGCGCG
GGA------------CGTGCTCTGGGTCCAGCCATCATCACCGGCTTCTGGGAGAACCAC
TGGGTGTACTGGATCGGGCCGGTGTCAGGCGGGGTCCTCGCCGGCATCTCCCACGAGTTC
TTCTTCGCCCACAGCGCCTCGCGCCAGCGGCTGGTGGCGTGTCTGACCTGCAAGGACGTG
GACATGGTGGAGACGGCCAGCGTGTCCCGGTCCTCGCTGTCCACCGTCACCCAGAATGCC
GGCCGCGCCAAACAGGCCAACAAG---GGCGACGGGAAC
>MN168445
GAGCTACGCAGTCGGCAGTTTTGGCGTGCAATGTTGGCAGAACTGCTGGGCACCCTGGTG
CTCGTGAGTACCATGCTCGGGGCCTCTGTGCCTGGTCCGGGAGAGGCCCCTGGGGGCCCC
CTGTACCCAGCGCTGGCAATATGCGCCGTGATCGTTTCCCTGGGTCACTGTTTTGGAGAG
ATCAGCGGGGCACAGGTGAACCCTGCCGTGACTCTGTCGCTGTTGGCTACAAGGAAAGTG
GACCTTGTGAGGGCTCTGGTGTATGTCGGTGCCCAGTGCTTAGGGGCCTTCCTCGCCACC
GGGGCCCTGTACCTAGCCCTCCCGCGGAAATCCACCGCGGAAGTCTTTGTCAATAGGGTC
CCGGTAGATGGCAACGCAGCGCAGGCGGTGGGCATGGAGGTGCTGTGCACCTTCTCGATG
GTCTTCACCATCTTCTCCGTGGAGGAACAGAGGCGGAGAGATAGCACAGAGCCGGGCAAC
CTGGGCATCGGATTGGCCCACACGGCCGCAGTGATGATAGGGGCAAGGTTTTCTGGTGCG
GGAATGAATCCAGCTCGTGCTCTGGGTCCAGCCATCATCACTGGCTTCTGGGAGAACCAC
TGGGTATACTGGATCGGCCCGGTGACGGGCGGCGTGCTGGCCGGCGTCTCCCACGAGTTC
TTCTTCGCCCACAGCGCCTCGCGCCAGAAGCTGGTGGCCTGTCTGACCTGTAAGGACATA
GACATGGTGGAGACGGCCAGCATGTCTCGCTCCTCGCTCTCCACCGTCACCCAGAATGCC
ACCAGGGCCAAGCAGGGCAACAAG---GGGGAGAGCAAC
>MN168446
GAGCTGCGCAGTCGACAGTTCTGGCGTGCGATGCTGGCAGAACTGCTCGGCACCCTGGTG
CTCGTGAGTACCATGCTCGGGGCCTCTGTGCCTGGTCCGGGAGAGCCCCCCGTGGGACCC
CTGTACCCAGCACTGGCAATATGTGCCGTGATCGTTACGCTGGGTCACTGTTTTGGAGAG
ATCAGCGGTGCACAGGTGAACCCTGCAGTGACTCTGTCACTGTTGGCCGCCAAGAAGATC
GACCTCGTCCGGGCCCTGGTGTTCGTCAGCGCGCAGTGCGTGGGGGCCTTCCTGGCCACC
GGGGCCCTGTACCTGGCCCTCCCACACAAATCCACTGCAGAAGTCTTTGTCAATAGGGTG
CCCGTAGAGGGCAACGCGGCGCAGGCGGTGGGCATGGAGATGCTGTGTACCTTCGCCATG
GTCTTCACCATCTTCTCCGTGGAGGAACAGAGGCGGAGAGAGAGCACCGAGCCTGGCAAC
CTGGGCATCGGATTGGCCCACACGGCTTCAGTGATGTTAGCGGCGAGGTTTTCTGGTGCG
GGGATGAACCCAGCGCGCGCTCTGGGTCCAGCGATCATCACCGGATTCTGGGAGAACCAC
TGGGTATACTGGATCGGCCCGGTGTCAGGCGGCGTGATGGCGGGCATCTCCCACGAGTTC
TTCTTCGCCCACAGCGCCTCGCGCCAGAAGCTGGTGGCCTGTCTGACCTGTAAGGACATA
GACATGGTGGAGACGGCCAGCATGTCCCGCTCCTCTCTCTCCACCGTCACCCAGAACGCC
TCCAGGGCCAAGCAGGCCAACAAG---GGGGAGGGCAAC
>MN168447
GAGCTACGCAGTCGGCAGTTTTGGCGTGCCATTTTGGCAGAACTGCTGGGCACCCTGGTG
CTCGTGAGTACCATGCTAGGGGCCTCTGTACCCGGTCCTGGAGAGGCCCCAGGGGGGCCC
CTGTACCCAGCCCTGGCAATAGCTGCTGTGATCGTTTCGCTGGGTCACTGTTTTGGGGAG
ATCAGCGGGGCACAGGTGAACCCTGCGGTGACTCTGTCACTGTTGGCCACCAAGAAGATA
GACCTGGTCAGGGCCCTGGTGTACGTAGGCGCACAGTGCCTGGGAGCCTTCCTGGCCACC
GGGGCCCTCTACCTGGCCCTCCCGCGCAAATCCACTGCAGAAGTGTTTGCCAACAGGGTG
CCTGTAGACGGCAACGCAGCACAGGCGCTGGGCATGGAGGTGCTGTGCACCTTCTCCATG
GTCTTCACCATCTTCTCTGTGGAGGAGCAGAGGCGGAGAGAGAGCACCGAGCCTGGCAAC
CTGGGCATTGGACTGGCCCACACGGCTGGAGTGATGATAGCGGCCAGGTTTTCTGGAGCG
GGAATGAATCCAGCTCGTGCTCTGGGTCCAGCCATCATCACTGGCTTCTGGGAGAACCAC
TGGGTATACTGGATCGGCCCGGTGACGGGCGGCGTCCTGGCCGGCGTGTCCCACGAGTTC
TTCTTCGCCCACAGCGCCTCGCGCCAGAAGCTGGTGGCCTGTCTGACCTGTAAGGACATC
GACATGGTGGAGACCGCCAGCATGTCACGCTCCTCCCTCTCCACTGTGACCCAGAACGCC
ATCAGGGCCAAGCAGGCCAACAAG---GCAGAGAGCAAC

>MN168448
GAGCTACGCAGTCGGCAGTTTTGGCGTGCCATTTTGGCAGAACTGCTGGGCACCCTGGTG
CTCGTGAGTACCATGCTAGGGGCCTCTGTACCCGGTCCTGGAGAGGCCCCAGGAGGGCCC
CTGTACCCAGCCCTGGCAATAGCTGCTGTGATCGTTTCGCTGGGTCACTGTTTTGGGGAG
ATCAGCGGGGCACAGGTGAACCCTGCGGTGACTCTGTCACTGTTGGCCACCAAGAAGATA
GACCTGGTCAGGGCCCTGGTGTACGTAGGCGCACAGTGCCTGGGAGCCTTCCTGGCCACC
GGGGCCCTCTACCTGGCCCTCCCGCGCAAATCCACTGCAGAAGTGTTTGCCAACAGGGTG
CCTGTAGACGGCAACGCAGCACAGGCGCTGGGCATGGAGGTGCTGTGCACCTTCTCCATG
GTCTTCACCATCTTCTCTGTGGAGGAGCAGAGGCGGAGAGAGAGCACCGAGCCTGGCAAC
CTGGGCATTGGACTGGCCCACACGGCTGGAGTGATGATAGCGGCCAGGTTTTCTGGAGCG
GGAATGAATCCAGCTCGTGCTCTGGGTCCAGCCATCATCACTGGCTTCTGGGAGAACCAC
TGGGTATACTGGATCGGCCCGGTGACGGGCGGCGTGCTGGCCGGCGTGTCCCACGAGTTC
TTCTTCGCCCACAGCGCCTCGCGCCAGAAGCTGGTGGCCTGTCTGACCTGTAAGGACATC
GACATGGTGGAGACCGCCAGCATGTCACGCTCCTCCCTCTCCACCGTCACCCAGAACGCC
ATCAGGGCCAAGCAGGCCAACAAG---GCAGAGAGCAAC
>MN168449
GAGCTACGCAGTCGGCAGTTTTGGCGTGCCATTTTGGCAGAACTGCTGGGCACCCTGGTG
CTCGTGAGTACCATGCTAGGGGCCTCTGTACCCGGTCCTGGAGAGGCCCCAGGGGGGCCC
CTGTACCCAGCCCTGGCAATAGCTGCTGTGATCGTTTCGCTGGGTCACTGTTTTGGGGAG
ATCAGCGGGGCACAGGTGAACCCTGCGGTGACTTTGTCACTGTTGGCCACCAAGAAGATA
GACCTGGTCAGGGCCCTGGTGTACGTAGGCGCGCAGTGCCTGGGAGCCTTCCTGGCCACC
GGGGCCCTCTACCTGGCCCTCCCGCGCAAATCCACTGCAGAAGTGTTTGCCAATAGGGTG
CCTGTAGACGGCAACGCAGCACAGGCGCTGGGCATGGAGGTGCTGTGCACCTTCTCCATG
GTCTTCACCATCTTCTCCGTGGAGGAGCAGAGGCGGAGAGAGAGCACCGAGCCTGGAAAC
CTGGGCATTGGATTGGCCCACACGGCTGGAGTGATGATAGCGGCCAGGTTTTCTGGAGCG
GGAATGAATCCAGCTCGTGCTCTGGGTCCAGCCATCATCACTGGCTTCTGGGAGAACCAC
TGGGTATACTGGATCGGCCCGGTGACGGGCGGCGTGCTGGCCGGCGTGTCCCACGAGTTC
TTCTTCGCCCACAGCGCCTCGCGCCAGAAGCTGGTGGCCTGTCTGACCTGTAAGGACATC
GACATGGTGGAGACCGCCAGCATGTCACGCTCCTCCCTCTCCACCGTCACCCAGAACGCC
ATCAGGGCCAAGCAGGCCAACAAG---GCAGAGAGCAAC
>MN168450
GAGCTACACAGTCGACAGTTTTGGCGTGCGATGCTGGCAGAACTCGTCGGCACCCTGGTG
CTTGTGAGCACCATGCTCGGGGCTTCCATGCCTGGTCCCGGAGAGGCCCCTGGGGGACCC
CTGTACCCGGCCCTGGCAATAGCTGCTGTGGTCGTTGCACTGCGTCACTGTTTTGGAGAG
ATCAGTGGGGCACAGGTGAACCCTGCGGTGACTTTGTCGCTATTGGCCACAAGAAAGATA
GACCTTCTCAGGGCCTTGGTTTACATAGGCGCACAGTGCCTAGGGGCCTTTCTGGCAGCT
GGGGCCCTCTATCTGGCCCTACCACGGAAATCCACTGCAGACGTCTTTGTCAATAGGGTG
CCCATAGACGTTAACGCAGCACAGGCACTCAGCATGGAGGTTCTGTGTACCTTCCAGATG
GTCTTCACCATCTTCTCTGTGGAAGAGCAGAGACGGAACGAGAGCACCGAACCAGGCAAT
TTAGGCATCGGATTGTCCCACACCGCTGGAGTTATGATAGGGGCAAGGTTCTCCGGTGCA
GGAATGAATCCAGCTCGTGCTCTGGGTCCTGCCATTATCACTGGCTTTTGGGAAAACCAC
TGGGTATACTGGATTGGACCTGTGGCTGGTGGGGTGCTGGCTGGGGTTTCCCACAAGTTC
TTCTTCGCACACAGCGCCTCTCGCCAGAAGCTGGTGGCCTGTGTGACCTGTAAGGACATT
GAGATCGTGGAGACAGCCAGCGTGTCCCGATCCTCACTGTCCACCGTCACCCAGAACGCC
ACCAGGGTCAAGCAGGCTAACAAG---GGAGAGAATAAC
>MN168451
GAGCTACGCAGTCGGCAGTTTTGGCGTGCCGTGCTGGCAGAAGTGCTTGGCACCCTGGTG
CTTGTGAGCACCATGCTCGGGGCCTCTGTGCCTGGCCCTGGAGAGGCCCCTGGGGGACCC
CTGTACCCTGCGCTGGCGATAGGTGTGGTGATTGTCGCACTGAGTCACTGTTTCGGAGAG
ATCAGCGGGGCACAGGTGAACCCTGCTGTGACTCTGTCACTCTTGGCGACAAAGAAGATG
GATCTTTTAAGGGCCCTTCTTTACATTGCCGCCCAGTGCCTCGGGGCATCTCTGGGGGCC

GGCGCCCTCTACTTGGCCCTACCGCGTAAAACCACCGCAGACTACTTCCTCAACAGGGTG
CCCATGGAGGTGAACGCGGCCCAGGCTCTGGGGATGGAGGTCCTGTGCACCTTCCAGATG
GTCTTCACCGTGTTTTCAGTGGAGGACCAGCGGCGCAGGGAGTGCCCTGAACCGGGGAAT
ATTGCCATCGGGTTTGCCCACACCGCCGGGGTTCTGATCGGGGCAAGGTTCTCGGGCGCG
AGCATGAACCCGGCGCGTGCTCTGGGTCCGGCTATCATCGTCGGCTTCTGGGAAAGCCAC
TGGGTATACTGGATCGGACCGGTGCTCGGTGCGGTGCTGGCCGGGGTCTCCCACGAGTTT
TTGTTCGCGCCCAGCGCGTCTCGCCAGAAGCTGGTGGCCTGTCTCACCTGCAAGGATATC
GAGATCGTGGAGACGGCCAGCGTGTCTCGGTCGTCGCTCTCCACCGTCACGCAGAACGCC
ATGAGAGCCAAGCAGCCAAACAAG---CAGGAGAACAAC
>MN168452
---CTACGCAGTCGGCAGTTTTGGCGCGCCATGCTGGCAGAAGTGCTTGGCACCCTGGTG
CTCGTGAGCACCATGCTCGGGGCCTCTGTGCCTGGCCCCGGAGAGGCCCCTGGGGGACCC
CTGTACCCAGCGCTGGCGATAGGTGTGGTGATTGTCGCACTGGGGCACTGTTTTGGAGAG
ATCAGCGGGGCACAGGTGAACCCTGCTGTGACTCTGTCGCTCTTGGCGACAAGGAAGATG
GATCTTTTAAGGGCCCTTCTTTACATAGCCGCCCAGTGCCTCGGGGCCTCTCTGGGAGCC
GGGGCCCTCTACTTGGCCCTACCACGTAAACCCACCGCAGACTACTTCCTCAACAGGGTG
CCCATGGAGGTGAACGCAGCCCAGGCTCTGGGGATGGAGGTCCTGTGCACCTTCCAGATG
GTCTTCACCGTGTTCTCAGTAGAGGACCAGCGGCGCAGGGAGTGCCCTGAACCGGGGAAC
ATTGCCATCGGGTTTGCCCACACCGCGGGGGTTCTGATCGGGGCAAGGTTCTCAGGTGCG
AGCATGAACCCTGCGCGTACTCTGGGTCCAGCTATCATCATCGGCTTCTGGGAAAACCAC
TGGGTATACTGGATCGGACCGGTGCTCGGTGCGGTGCTGGCCGGGGTCTCCCACGAGTTC
TTCTTCGTGCCCAGTGCCTCCCGCCAGAAGCTGGTGGCTTGTCTGACCTGCAAGGATATC
GAGATTGTGGAAACAGCCAGCGTGTCCCGGTCGTCGCTCTCCACTGTCACGCAGAACGCC
ATGAGACCCAAGCAGCCCAACAAG---CAGGAGAACAAC
>MN168453
GAGATACGCAGTCGGCAGTTCTGGCGTGCAGTGCTAGCAGAACTGCTGGGCACCCTGGTG
TTTGTGAGTACCATGCTTGGGGCCTCTGTTCCAGGCTCTGAAAATGCCCCTGGGGGACCC
CTGTACCCAGCCATGGCAGCAGGTGTGGCCATTGTTGCAGTAGCCCACTGTTTTGGAGAG
ATCAGTGGGGCACAGATTAATCCGGCTGTGACTCTGTCCATGATGGCTACACGGAAGTTA
GATGTTCTCAGGGGTCTTGTGTATATCGTTGCACAGTGTGTGGGTGCCTTCCTTGCAGCC
GGTGGCCTCTACCTGGCCCTTCCACTCAAATCCACTTCAGAATTCTTCGTCAACAAGGTT
CCCATGGAGGTGAATGCACTTCAGGCATTGGGAATGGAGGTCTTGTGCACCTTCCAGATG
GCCTTTACAGTGTACTCAGTGGAAGACCAACGAAAAAGAGAGAGCACGGAACCAGGGAAC
CTAGGAATTGGATTGGCCCACACTGCAGGAGTTCTGATAGCAGCACGGTTCTCTGGAGCC
TGTATGAACCCTGCTCGTGCTCTGGGTCCAGCAATCATCACTGGCTTCTGGGAGAGCCAC
TGGGTATATTGGATTGGGCCGGTTGTGGGGGCCATGATGGCAGGCATGGCCCACGAGTTC
TTCTTCGCTCCCAGTGCATCTCGCCAGAAGCTGGTGGCCTGCATGACCTGTAAGGACATA
GATATGGTGGAGACGGCCAGCGTGTCCCGGTCCTCTCTCTCCACGGTCACGCAGAACGCC
TTGAGAGCCAAGCAGGCTAGCAAA---CAGGAAAACAAC
>MN168454
GAGCTACGCAGTCGGCAGTTCTGGCGTGCCGTGCTAGCAGAACTGCTTGGCACCCTGGTG
TTTGTGAGTGCCATGCTTGGGGCTTCTGTTCCAGGCCCTGAAAAGGCCCCTGGGGGACCC
CTGTACCCAGCCATGGCAGCAGGTGTGGCGATTGTTGCAGTAGCCCACTGTTTTGGAGAG
ATCAGCGGGGCACAGATTAACCCTGCTGTGACTCTGTCCATGATGGCTACACGGAAGGTA
GATGTTCTCAGGGGTCTTGTGTATATCGTTGCACAGTGTGTGGGGGGCCTTCCTTGCAGCT
GGTGCCCTCTACCTGGCCCTTCCACTCAAATCCACCGCAGAATTCTTCGTCAACAGGGTG
CCTATGGAGGTTAATGCAGCCCAGGCTTTGGGGATGGAGGTGTTATGCACCTTCCAGATG
GCCTTCACAGTGTACTCAGTGGAAGACCAACGAAGAAGAGAGAGCACAGAACCAGGGAAC
CTAGGAATTGGATTGGCGCACACTGCAGGAGTTCTGATAGCAGCACGGTTCTCTGGAGCC
TGTATGAACCCTGCACGTGCTCTGGGTCCAGCCATCATCACTGGCTTCTGGGAAAACCAC
TGGGTATACTGGATCGGGCCGGTTCTGGGCGCCATGCTGGCCGGCATGTCCCACGAGTTC

TTCTTCGTGCACAGTGCGTCTCGCCAGAAGCTGGTGGCCTGTCTGACCTGTAAGGACATT
GAGATGGTGGAGATGGCCAGTGTGTCACGCTCCTCTCTCCACGGTCACGCAGAATGCC
TTGAGAGGCAAGCAGGCTAACAAA---CAGGAAAACAAC
>MN168455
GAGTTACGCAGCCGGCAATTCTGGCGGGCCGTGCTGGCAGAGCTGCTGGGCACCCTGGTG
TTTGTGAGTGCAGTGCTGGGGGCCTGTGTTCCAGGGCCTGGAGGCGTCTCTGGGGGGCCC
CTGTACCCAGCCCTATCTGTGGGCATGGTGGTTGTTGCCCTGAGCCACTGTTTTGGAGAG
ATCAGTGGGGCACAGGTGAACCCTGCAGTTACTGTGGCCATGTTGGCTACACGGAAGCTG
GATGTTCTGAGGGCCCTGGTCTACTTAGCAGCACAATGTGCTGGGGGAAGTCTAGGGGCT
GGGGCCCTCTACCTGGCTCTGCCCCTCAAAACCCCTGCAGACTGCTTTGTCAATAAGGTG
CCCATAGATGTGAATGCAGCTCAGGCTCTGGGTATAGAGATCCTGTGCACCTTCCAGATG
GTCTTCACCGTGTTCGCAGTGGGGGACCAGAGACGAAGGGAGAGCACTGAACCAGGGAAC
TTGGCCATTGGAGCTGCCCACACTGCTGGAGTTTTTATAGGGGCCCGGTTCTCTGGTGGG
AGTATGAACCCCGCTCGCTCCTTCGCTCCAGCTGTCATCACTGGCTTCTGGGAGAACCAC
TGGGTATACTGGATCGGGCCAGTGCTGGGTGGAGTGTTGGCCGGGGTGTCCCACGAGTTC
TTCTTTGCACCCAGTGCGTCTCGCCAGAAGCTGGTGGCCTGTATGACCTGTAAAGACATT
GAGATCGTGGAGACGGCCAGCGTGTCTCGGTCGTCTCTCTCCACTGTCACACAGAACGCC
GTGAGAGCCAAGCAGGCAAACAAA---CAGGACAACAAC
>MN168456
GAGCTGTGTAGTCGTCAGTTCTGGCGTGCCATTCTGGCAGAACTACTTGGCACCCTGGCA
TTTGTGAGTGCAGTTCTGGGGGCCTCGGTGCCAGGTCCCGGAGAGGCCTCTAGGGGACCC
CTGTACCCTGCTCTGGCAGCAGGCACGGTAGCTGTAGCCCTGGGTCACTGCTTTGGAGAG
ATTAGTGGTGCACAGGTGAAT---------------------------------------

>MN168457
GAGTTGCGCAGTCGTCAGTTTTGGCGAGCCATGCTTGCGGAGCTGCTGGGCTCCCTGGTG
TTTGTGAGTGCCGTGTTGGGCTCCTCGGTGCCCGGCTCTGGAGAGGCCCCACCAGGCCCC
CTCTACCCAGCCCTGGCTGCTGGCATGGTGGCTGTTGCCTTGGGTCACTGCTTTGGAGAG
ATCAGTGGGGCGCAGGTAAATCCTGCGGTGACCTTGTCCCTGCTGGCTACACGGAAGCTA
GACCTGCTCAGGGCACTGGTCTACGTCGGGGCCCAATGTCTGGGGGCCTCGCTAGGGGCT
GGAGCCCTCTACCTCGCCCTGCCACTAAAATCCACTGCAGAATGCTTTGTCAGCAAGGTA
CCTATGGAGTTGAACGCTGCCCAGGCTCTGGGGATGGAGGTCCTGTCGACGTTCCAGCTA
GTCTTCACAGTGTTCTCTGTGGAGGATCAGAGGAGGCGGGAGAACGTGGAGCCAGGGAAC
CTGGCCATTGGGTTCTCTGTCAGTGCTGGGGTCCTCATGGCGGGTCGGTTCTCCGGTGGC
AGTATGAACCCAGCCCGCTCCCTAGGCCCAGCCATCATCACAGGCTTCTGGGAGAACCAC
TGGGTATACTGGATCGGGCCAGTGCTTGGGGCAGTTCTGGCTGCGTGGTCCCATGAGTTC
TTCTTTGCGCCCGGTGCATCACGCCAGAAGCTGGTGGCATGTCTGACCTGTAAGGACATC
GAGATGGTGGAGACGGCCAGCGTGTCTCGCTCCTCCCTGTCCACCGTCACACAGACCGCC
ATGAGAGCCAAGCAGACCAACAAG---CAGGAGCACAAC
>MN168458
GAGTTGCGCAGTCGTCAGTTTTGGCGAGCCATGCTTGCGGAGCTGCTGGGCTCCCTGCTG
TTTGTGAGTGCCGTCTTGGGCTCCTCGGTGCCCGGCTCTGGAGAGGCCCCACCAGGCCCC

CTCTACCCAGCCCTGGCTGCTGGCATGGTGGCTGTTGCCCTGGGTCACTGCTTTGGAGAG
ATCAGTGGGGCACAGGTCAATCCTGCCGTGACCTTGTCCCTGTTGGCCACACGGAAGCTA
GACCTGCTCAGGGCTCTGGTCTACATTGGAGCCCAATGTCTGGGGGCCTCGCTAGGGGCT
GGAGCCCTCTATCTTGCCCTCCCACTAAAATCCACTGCTGAATGCTTTGTCAGCAAGGTA
CCTATGGAGCTGAATGCTGCCCAGGCTCTGGGGATGGAGGTTCTGTCGACGTTCCAGCTG
GTCTTCACAGTGTTCTCTGTGGAGGACCAGAAGAGGCGGGAGAACATGGAGCCAGGGAAC
CTGGCTGTTGGGTTCTCCATCAGCGCTGGGGTCCTCATGGCGGGTCGGTTCTCTGGTGGC
AGTATGAACCCAGCCCGCTCCCTGGGCCCAGCCATCATCACAGGCTTCTGGGAAAACCAC
TGGCTGTACTGGATCGGACCCGTCCTTGGGGCAGTTCTGGCTGCCTGGTCCCACGAGTTC
TTCTTTGCACCCGGGGCTTCACGCCAGAAGCTGGTGGCATGTCTGACCTGTAAGGACATT
GAGATGGTGGAGACGGCCAGTGTGTCTCGCTCCTCCCTGTCCACCGTCACCCAGACCGCC
ATGAGAGCCAAGCAGACCAACAAG---CAGGAGCACAAC
>MN168459
GAGTTGCGTAGTCGTGAGTTCTGGCGTGCCATGCTGGCCGAGCTGCTGGGTTCCCTGGTG
TTTGTGAGTGCTGTTCTGGGGGCCTCTGTGCCGGGCCCTGGAGAGGCCTCCACGGGGCCC
CTCTACCCAGCCCTGGCTGCAGGCATGGTGGCTGTTGCCCTGGGACACTGTTTTGGAGAG
ATCAGCGGAGCACAGGTGAACCCAGCAGTTACCCTGTCTCTGTTGGCCACGCGGAAGCTG
GACCTGGTGAGGGCCCTGGTGTACGTGGGAGCCCAGTGTCTGGGGGCCTCCCTGGGGGCC
GGGGCTCTCTACTTCGCCCTGCCCCTCAAAACCACCGCAGACTGCTTCGTCAGCAGGGTT
CCTCTCGAGGTGAACGCGGCCCAGGCTCTAGGGATGGAGCTTCTGTCAACGTTCCAGCTG
GTCTTCACTGTGTTCTCCGTGGAGGACCACCGTCGGAGGGAGTACACAGAACCAGGGAAC
CTGGCCATCGGCTTCTCTCTCAGCGCAGGCGTGCTCATCGGGGGTCGGTTCTCTGGAGGC
AGTATGAACCCGGCACGCTCCCTGGGTCCAGCCATCATCACTGGCTTCTGGGAAAACCAC
TGGGTATACTGGATTGGACCGGTGCTGGGTGCAGTGCTGGCCGGGGTGTCCCATGAGTTC
TTCTTTGCCCCCAGTGCATCGAGGCAGAAGCTGGTTGCGTGTCTGACCTGTAAGGACATA
GAGATGGTGGAGGCAACCAGCGTATCCCGGTCCTCCCTGTCCACCGTCACCCAGACCGCC
ATGAGGGCTAAGCAGGTCAACAAA---CAGGAGCACAAC
>MN168460
GAGTTGCGTAGTCGTGAGTTCTGGCGTGCCATGCTGGCCGAGCTGCTGGGTTCCCTGGTG
TTTGTGAGTGCTGTTCTGGGGGCCTCTGTGCCGGGCCCTGGAGAGGCCTCCACGGGGCCC
CTCTACCCAGCCCTGGCTGCAGGCATGGTGGCTGTTGCCCTGGGACACTGTTTTGGAGAG
ATCAGCGGAGCACAGGTGAACCCAGCAGTTACCCTGTCTCTGTTGGCCACGCGGAAGCTG
GACCTGGTGAGGGCCCTGGTGTACGTG---------------GGGGCCTCCCTGGGGGCC
GGGGCTCTCTACTTCGCCCTGCCCCTCAAAACCACCGCAGACTGCTTCGTCAGCAGGGTT
CCTCTCGAGGTGAACGCGGCCCAGGCTCTAGGGATGGAGCTTCTGTCAACGTTCCAGCTG
GTCTTCACTGTGTTCTCCGTGGAGGACCACCGTCGGAGGGAGTACACAGAACCAGGGAAC
CTGGCCATCGGCTTCTCTCTCAGCGCAGGCGTGCTCATCGGGGGTCGGTTCTCTGGAGGC
AGTATGAACCCGGCACGTTCCCTGGGTCCAGCCATCATCACTGGCTTCTGGGAAAACCAC
TGGGTGTACTGGATTGGACCGGTGCTGGGTGCAGTGCTGGCCGGGGTGTCCCATGAGTTC
TTCTTTGCCCCCAGTGCATCGAGGCAGAAGCTGGTTGCGTGTCTGACCTGTAAGGACATA
GAGATGGTGGAGGCAACCAGCGTATCCCGGTCCTCCCTGTCCACCGTCACCCAGACCGCC
ATGAGGGCTAAGCAGGTCAACAAA---CAGGAGCAAAAC
>MN168461
GAGTTGCGTAGTCGTGAGTTCTGGCGTGCCATGCTGGCCGAGCTGCTGGGTTCCCTGGTG
TTTGTGAGTGCTGTTCTGGGGGCCTCTGTGCCGGGCCCTGGAGAGGCCTCCACGGGGCCC
CTCTACCCAGCCCTGGCTGCAGGCATGGTGGCTGTTGCCCTGGGACACTGTTTTGGAGAG
ATCAGCGGAGCACAGGTGAACCCAGCAGTAACCCTGTCTCTGTTGGCCACGCGGAGGCTG
GACCTGGTGAGGGCCCTGGTATACGTGGGAGCCCAGTGTCTGGGGGCCTCCCTGGGGGCC
GGGGCTCTCTACTTCGCCCTGCCCCTCAAAACCACCGCAGACTGCTTCGTCAGCAAGGTT
CCTCTCGAGGTGAACGCGGCCCAGGCTCTAGGGATGGAGCTTCTGTCAACGTTCCAGCTG
GTCTTCACTGTGTTCTCCGTGGAGGACCACCGTCGGAGGGAGTACACAGAACCAGGGAAC

CTGGCCATCGGCTTCTCTCTCAGCGCAGGCGTGCTCATCGGGGTCAGATTCTCTGGAGGC
AGTATGAACCCGGCACGTTCCCTGGGTCCAGCCATCATCACTGGCTTCTGGGAAAACCAC
TGGGTATACTGGATTGGACCGGTGCTGGGTGCAGTGCTGGCCGGGGTGTCCCATGAGTTC
TTCTTTGCCCCCAGTGCATCGAGGCAGAAGCTGGTTGCGTGTCTGACCTGTAAGGACATA
GAGATGGTGGAGGCAACCAGCGTATCCCGGTCCTCCCTGTCCACCGTCACCCAGACCGCC
ATGAGGGCTAAGCAGGTCAACAAA---CAGGAGCAAAAC
>MN168462
GAGTTGCGTAGTCGTGAGTTCTGGCGTGCCATGCTGGCCGAGCTGCTGGGTTCCCTGGTG
TTTGTGAGTGCTGTTCTGGGGGCCTCTGTGCCGGGCCCTGGAGAGGCCTCCACGGGGCCC
CTCTACCCAGCCCTGGCTGCAGGCATGGTGGCTGTTGCCCTGGGACACTGTTTTGGAGAG
ATCAGCGGAGCCCAGGTGAACCCAGCAGTTACCCTGTCTCTGTTGGCCACGCGGAGGCTG
GACCTGGTGAGGGCCCTGGTGTACGTGGGAGCCCAGTGTCTGGGGGCTTCCCTGGGGGCC
GGGGCTCTCTATTTCGCCCTGCCCCTCAAAACCAACGCAGACTGCTTCGTCAGCAGGGTT
CCTCTCGAGGTGAACGCGGCCCAGGCTCTAGGGATGGAGCTTCTGGCAACGTTCCAGCTG
GTCTTCACTGTGTTCTCTGTGGAGGACCACCGTCGGAGGGAGAACACAGAACCAGGGAAC
CTGGCCATCGGCTTCTCTCTCAGCGCAGGCGTGCTCATCGGGGGTCGGTTCTCTGGAGGC
AGTATGAACCCGGCACGTTCCCTGGGTCCAGCCATCATCACTGGCTTCTGGGAAAACCAC
TGGGTGTACTGGATTGGACCGGTGCTGGGTGCAGTGCTGGCCGGGGTGTCCCATGAGTTC
TTCTTTGCCCCCAGTGCATCGAGGCAGAAGCTGGTTGCGTGTCTGACCTGTAAGGACATA
GAGATGGTGGAGGCAACCAGCGTATCCCGGTCCTCCCTGTCCACCGTCACCCAGACCGCC
ATGAGGGCTAAGCAGGTCAACAAA---CAGGAGCAAAAC
>MN168463
GAGTTGCGCAGTCGTGAGTTCTGGCGTGCCATGCTGGCCGAGCTGCTGGGTTCCCTGGTG
TTTGTGAGTGCTGTTCTGGGGGCCTCTGTGCCGGGCCCTGGAGAGACCTCCACGGGGCCC
CTCTACCCAGCCCTGGCTGCAGGCATGGTGGCTGTTGCCCTGGGACACTGTTTTGGAGAG
ATCAGCGGAGCACAGGTGAACCCAGCAGTTACCCTGTCTCTGTTGGCCACGCGGAGGCTG
GACCTGATGAGGGCCCTGGTGTACGTGGGAGCCCAGTGTCTGGGGGCTTCCCTGGGGGCC
GGGGCTCTCTACTTCGCCCTGCCCCTCAAAACCACCGCAGACTGCTTCGTCAGCAGGGTT
CCTCTCGAGGTGAATGCAGCCCAGGCTCTAGGGATGGAGCTTCTGTCAACGTTCCAGCTG
GTCTTCACTGTGTTCTCCGTGGAGGACCACCGTCGGAGGGAGAATACAGAACCAGGGAAC
CTGGCCATCGGCTTCTCTCTCAGCGCAGGCGTGCTCATCGGGGGTCGGTTCTCTGGAGGC
AGTATGAACCCGGCACGTTCCCTGGGTCCAGCCATCATCACTGGCTTCTGGGAAAACCAC
TGGGTATACTGGATTGGACCGGTGCTGGGTGCAGTGCTGGCCGGGGTGTCCCATGAGTTC
TTCTTTGCCCCCAGTGCATCGAGGCAGAAGCTGGTTGCGTGTCTGACCTGTAAGGACATA
GAGATGGTGGAGGCAACCAGCGTATCCCGGTCCTCCCTGTCCACCGTCACCCAGACCGCC
ATGAGGGCTAAGCAGGTCAACAAA---CAGGAGCAAAAC
>MN168464
GAGTTGCGTAGTCGTGAGTTCTGGCGTGCCATGCTGGCCGAGCTGCTGGGTTCCCTGGTG
TTTGTGAGTGCTGTTCTGGGGGCCTCTGTGCCGGGCCCTGGAGAGACCTCCACGGGGCCC
CTCTACCCAGCCCTGGCTGCAGGCATGGTGGCTGTTGCCCTGGGACACTGTTTTGGAGAG
ATCAGCGGAGCACAGGTGAACCCAGCAGTTACCCTGTCTCTGTTGGCCACGCGGAGGCTG
GACCTGATGAGGGCCCTGGTGTACGTGGGAGCCCAGTGTCTGGGGGCTTCCCTGGGGGCC
GGGGCTCTCTACTTCGCCCTGCCCCTCAAAACCACTGCAGACTGCTTCGTTAGCAGGGTT
CCTCTCGAGGTGAATGCAGCCCAGGCTCTAGGGATGGAGCTTCTGTCAACGTTCCAGCTG
GTCTTCACTGTGTTCTCCGTGGAGGACCACCGTCGGAGGGAGAACACAGAACCAGGGAAC
CTGGCCATCGGCTTCTCTCTCAGCGCAGGCGTGCTCATCGGGGGTCGGTTCTCTGGAGGC
AGTATGAACCCGGCACGTTCCCTGGGTCCAGCCATCATCACTGGCTTCTGGGAAAACCAC
TGGGTATACTGGATTGGACCGGTGCTGGGTGCAGTGCTGGCCGGGGTGTCCCATGAGTTC
TTCTTTGCCCCCAGTGCATCGAGGCAGAAGCTGGTTGCGTGTCTGACCTGTAAGGACATA
GAGATGGTGGAGGCAACCAGCGTATCCCGGTCCTCCCTGTCCACCGTCACCCAGACCGCC
ATGAGGGCTAAGCAGGTCAACAAA---CAGGAGCAAAAC

>MN168465
GAGTTGCGTAGTCGTGAGTTCTGGCGTGCCATGCTGGCCGAGCTGCTGGGTTCCCTGGTG
TTTGTGAGTGCTGTTCTGGGGGCCTCTGTGCCAGGCCCAGGAGAGGCCTCCACGGGGCCC
CTCTACCCAGCCCTGGCTGCAGGCATGGTGGCTGTTGCCCTGGGACACTGTTTTGGAGAG
ATCAGCGGAGCACAGGTGAACCCAGCAGTTACCCTGTCTCTGTTGGCCACGCGGAGGCTG
GACCTGGTGAGGGCCCTGGTGTACGTGGGAGCCCAGTGTCTGGGGGCTTCCCTGGGGGCC
GGGGCTCTCTACTTCGCCCTGCCCCTCAAAACCACCGCAGACTGCTTCGTCAGCAGGGTT
CCTCTCGAGGTGAATGCGGCCCAGGCTCTAGGGATGGAGCTTCTGTCAACGTTCCAGCTG
GTCTTCACTGTGTTCTCCGTGGAGGACCAGCGTCGGAGGGAGAACACAGAACCAGGGAAC
CTGGCCATCGGCTTCTCTCTCAGCGCAGGCGTGCTCATCGGGGGTCGGTTCTCTGGAGGC
AGTATGAACCCGGCACGTTCCCTGGGTCCAGCCATCATCACTGGCTTCTGGGAAAACCAC
TGGGTATACTGGATTGGACCGGTGCTGGGTGCAGTGCTGGCCGGGGTGTCCCATGAGTTC
TTCTTTGCCCCCAGTGCGTCGAGGCAGAAACTGGTGGCGTGTCTGACCTGTAAGGACATA
GAGATGGTGGAGGCAACCAGCGTATCCCGGTCCTCCCTGTCCACCGTCACCCAGACCGCC
ATGAGGGCTAAGCAGGGCAACAAA---CAGGAGCAAAAC
>MN168466
GAGTTGCGTAGTCGTGAGTTCTGGCGTGCCATGCTGGCCGAGCTGCTGGGTTCCCTGGTG
TTTGTGAGTGCTGTTCTGGGGGCCTCTGTTCCGGGTCCTGGAGAGGCCTCCACGGGGCCT
CTCTACCCAGCCCTGGCTGCAGGCATGGTGGCTGTTGCCCTGGGACACTGCTTTGGAGAG
ATCAGCGGAGCACAGGTAAACCCAGCAGTTACCCTGTCTCTGTTGGCCACACGGAGGCTG
GACCTGGTGAGGGCTCTAGTGTACGTGGGAGCCCAGTGTCTGGGGGCCTCCCTAGGGGCC
GGGGCTCTCTACCTCGCCCTGCCCCTCAAAACCACTGCAGACTGCTTTGTCAGCAGGGTT
CCTCTGGAGGTGAATGCGGCCCAGGCTCTAGGGATGGAGCTTCTGTCAACGTTCCAGCTG
GTCTTCACTGTGTTCTCCGTGGAGGACCACCGTCGGAGGGAGAACACAGAGCCAGGGAAC
CTGGCCATCGGCTTCTCTCTCAGCGCAGGCGTGCTCATCGGGGGTCGGTTCTCTGGAGGC
AGTATGAACCCGGCACGTTCCCTGGGTCCAGCCATCATCACTGGCTTCTGGGAAAACCAC
TGGGTGTACTGGATTGGGCCGGTGCTGGGTGCAGTGCTGGCCGGGGTGTCCCATGAGTTC
TTCTTTGCCCCCAGTGCGTCGAGGCAGAAGCTGGTTGCGTGTCTGACTTGTAAGGACATC
GAGATGGTGGAGGCAACAAGCGTGTCCCGGTCCTCCCTGTCCACCGTCACCCAGACCGCC
ATGAGGTCTAAGCAGGTCAACAAA---CAGGAGCAAAAC
>MN168467
GAGTTGCGTAGTTGTGAGTTCTGGCGTGCCATGTTGGCCGAGTTGCTGGGCTCCCTGGTG
TTTGTGAGTGCTGTGCTGGGGGCCTCTGTGCCGGGCCCTGGAGAGGCCTCCATGGGGCTC
CTCTACTTAGCCCTGGCCGCAGGCATGGTGGCTGTCACCCTGGGACACTGCTTTGTAGAG
ATCAGCTGGGCACAGGTGAACCCA---------TTGTCTATTTTTATCTCTCAAAGGCTG
GACTTGCTGAGGGCACTGGTGTATGTGGTGGCTCATTGTGTGGGGGCCTCCCTGGGGGCT
GGGGCTCTCTACCTCTCC---CCCCTAAAAACCACAGCAGACTGCTTCATCAGCAGGCTC

>MN168468
GAGTTGCGTAGTTGTGAGTTCTGGCGTGCCATGTTGGCTGAGTTGCTGGGCTCCCTGGTG
TTTGTGAGTGCTGTGCTGGGGGCCTCTGTGCCGGGCCCTGGAGAGGCCTCCATGGGGCTC
CTCTACCCAGCCCTGGCCGCAGGCATGGTGGCTGTCACCCTGGGACACTGCTTTATAGAG
ATCAGCTGGGCACAGGTGAACCCA------------------------------------

---------------------CCCCTAAAAACCACAGCAGACTGCTTCATCAGCAGGCTC

>MN168469
GAGTTGCGTAGTCGTGAGTTCTGGCGTGCCATGCTGGCCGAGTTGCTGGGTTCTCTGGTG
TTTGTGAGTGCTGTGCTGGGGGCCTCTGTGCCGGGCCCTGGAGAGGCCTCCATGGGGCTC
CTCTACCCAGCCCTGGCCGCAGGCATGGTGGCTGTCACCCTGGGACACTGCTTTGGAGAG
ATCAGCTGGGCACAGGTGAACCCC------------------------------------

------------------------CTAAAAACCACAGCAGACTGCTTCGTCAGCAGGCTC

>MN168470

------------CAGGTGAACCCAGCAGTGACCCTGTCTCTGTTGGCCAAGCAAAGGCTG
GATCTGCTGAGGGCACTGGTGTATGTGGGGGCCCAGTGT---------------------
---------------------CCCCTAAAAACCACAGCAGACTGCTTCGTCAGCAGGCTC

>MN168471
GAGCTGCGTAGTCGTGAGTTCTGGCGGGCTGTGCTGGCCGAGCTGATGGGTTCCTTGGTG
TTTGTCAGTGCGGTCCTGGGGGCCTCTGTGCCGGGCCCTGGAGGACCCTCAACTGGGCCA
CTCTACCCTGCCCTGGCCGCAGGCATGGTGGCTGTGGCCCTGGGACACTGCTTCGGTGAG
ATCAGCGGGGCACAGGTGAACCCAGCAGTGACCCTGTCTCTGTTGGCCACGCGGAGGATG
GACACGCTGAAGGCTCTGGCGTATGTGGTGGCCCAGTGTCTTGGGGCCATAATGGCGGCC
TCTGCTCTCTACCTTGCCCTGCCTCTCAGAACCACTGCCGACTGCTTCATCAGCATGGTT
CCCCTGGAGGTGAATGTGGTACAGGCTCTAGGGATGGAGGCTCTCTCAACGTTCCAGCTG
GTCTTCACTGTGTTTTCTGTGGAGGACCAGCGACGGAGGGAGAACCCAGAACCAGGCAAC
CTGGCCATCGGCTTCTCTATCAGTGCAGGCGTGCTGACCGCGGGTCAGTACTCTGGTGGC
AGTATGAATCCGGCACGTTCCCTGGGTCCTGCCATCATCACTGGCTTCTGGGAAAACCAC
TGGGTATACTGGATCGGGCCGGTGTTGGGAGCAGTGCTTGCCGGGATGTCCCATGACTTT

TTCTTTGCTCCTAGTGCGTCAAGGCAGAAGCTGGTTGCGTGTCTGACCTGTAAGGACATA
GAGATGGTGGAAACAGCCAGCGTGTCCCGGTCCTCCCTGTCTACAGTTACCCAGACCGCC
ATGAGGGCTAAGCAGGTCAACAAA---CAGGAGCAAAAC
>MN168472
GAGCTGAGGAGCCGTCAGTTCTGGCAGGGGATTCTGGCAGAAACTCTTGGCTCTCTGGTC
TTTGTATCTGCTGTGTTGGGCTCTTTAGTGCCGGGGCCGGACGGGGCCTCCCCGGGGCCC
ATTTACCCCGCACTGGCTGCTGGTATGGCAACTGTGGTTCTGGGATATTGCTTTGGTGAA
ATCAGTGGGGCTCAGGTGAATCCTGCGGTGACTGTGGCCCTGTTGGCCATGCGTAAGGTG
GATGTGTTGAGGGCTGTGGTTTATCTGCTCGCTCAGTGTTTGGGTGGGATCCTCGCAGCT
GGCCTCATGTACCTCTCTCTGCCCCTGAAGTCCACAGCACAGAACTTCATCAACAAGGTC
CCAGTGGATATGAACGCAGGTCAAGCTCTTGGGATGGAGATGCTTGCCACGTTTCTCCTG
GGTTTCACTGTATTTTCTGTGGAAGATCAACGCAGGAGAGAAATAAATGAACCAGGGAAT
TTGGCCATTGGATTTGCTGTGACCACTGCCATCTTTATTGCTGGGAGGTTTTCTGGTGCC
AGCCTGAACCCAGCTCGCTCTCTTGGTCCCGCTATAATACTTGGATATTGGGAACATCAC
TGGGTCTACTGGATTGGTCCAATATTAGGTGCAGTCCTGGCTGGAGTGTCTCATGAGTTC
ATCTTTGCCCCCAGTGCGTCCAGACAGAAGCTGGTGGCATGTTTGACCTGCAAGGACATT
GAGATTGTGGAGACGGCCAGCGTGTCTCGATCGTCCCTGTCCACTGTCACACAGAGCGCC
ATGAGAAACAAACAAAGCAACAAA---CTGGAGCACAGC
>MN168473
GAGCTGAGGAGCCGTCAGTTCTGGCAGGGGATTCTGGCAGAAACTCTTGGCTCTCTGGTC
TTTGTATCTGCTGTGTTGGGCTCTTTAGTGCCGGGGCCGGACGGGGCCTCCCCGGGGCCC
ATTTACCCCGCACTGGCTGCTGGTATGGCAACTGTGGTTCTGGGATATTGCTTTGGTGAA
ATCAGTGGGGCTCAGGTGAATCCTGCGGTGACTGTGGCCCTGTTGGCCATGCGTAAGGTG
GATGTGTTGAGGGCTGTGGTTTATCTGCTCGCTCAGTGTTTGGGTGGGATCCTCGCAGCT
GGCCTCATGTACCTCTCTCTGCCCCTGAAGTCCACAGCACAGAACTTCATCAACAAGGTC
CCAGTGGATATGAACGCAGGTCAAGCTCTTGGGATGGAGATGCTTGCCACGTTTCTCCTG
GGTTTCACTGTATTTTCTGTGGAAGATCAACGCAGGAGAGAAATAAATGAACCAGGGAAT
TTGGCCATTGGATTTGCTGTGACCACTGCCATCTTTATTGCTGGGAGGTTTTCTGGTGCC
AGCCTGAACCCAGCTCGCTCTCTTGGTCCCGCTATAATACTTGGATATTGGGAACATCAC
TGGGTCTACTGGATTGGTCCAATATTAGGTGCAGTCCTGGCTGGAGTGTCTCATGAGTTC
ATCTTTGCCCCCAGTGCGTCCAGACAGAAGCTGGTGGCATGTTTGACCTGCAAGGACATT
GAGATTGTGGAGACGGCCAGCGTGTCTCGATCGTCCCTGTCCACTGTCACACAGAGCGCC
ATGAGAAACAAACAAAGCAACAAA---CTGGAGCACAGC
>MN168474
GAGCTGAGGAGCCGTCAGTTCTGGCAGGGGATTCTGGCAGAAACTCTTGGCTCTCTGGTC
TTTGTATCTGCTGTGTTGGGCTCTTTAGTGCCGGGGCCGGACGGGGCCTCCCCGGGGCCC
ATTTACCCCGCACTGGGTGCTGGTATGGCAACTGTGGTTCTGGGATATTGCTTTGGTGAA
ATCAGTGGGGCTCAGGTGAATCCTGCGGTGACTGTGGCCCTGTTGGCCATGCGTAAGGTG
GATGTGTTGAGGGCTGTGGTTTATCTGCTCGCTCAGTGTTTGGGTGGGATCCTCGCAGCT
GGCCTCATGTACCTCTCTCTGCCCCTGAAGTCCACAGCACAGAACTTCATCAACAAGGTC
CCAGTGGATATGAACGCAGGTCAAGCTCTTGGGATGGAGATGCTTGCCACGTTTCTCCTG
GGGTTCACTGTATTTTCTGTGGAAGATCAACGCAGGAGAGAAATAAATGAACCAGGGAAT
TTGGCCATTGGATTTGCTGTGACCACTGCCATCTTTATCGCTGGGAGGTTTTCTGGTGCC
AGCCTGAACCCAGCTCGCTCTCTTGGTCCCGCTATAATCCTTGGATATTGGGAACATCAC
TGGGTATACTGGATTGGTCCAATATTAGGTGCAGTCCTGGCTGGAGTGTCTCATGAGTTC
ATCTTTGCCCCCAGTGCGTCCAGACAGAAGCTGGTGGCATGTTTGACCTGCAAGGACATT
GAGATTGTGGAGACGGCCAGCGTGTCTCGATCGTCCCTGTCCACTGTCACACAGAGCGCC
ATGAGAAACAAACAAAGCAACAAA---CTGGAGCACAGC
>MN168475
---CTGAGGAGCCGTCAGTTCTGGCAGGGGATTCTGGCAGAAACTCTTGGCTCTCTGGTC
TTTGTATCTGCTGTGTTGGGCTCTTTAGTGCCGGGGCCGGACGGGGCCTCCCCGGGGCCC

ATTTACCCCGCACTGGCTGCTGGTATGGCAACTGTGGTTCTGGGATATTGCTTTGGTGAA
ATCAGTGGGGCTCAGGTGAATCCTGCGGTGACTGTGGCCCTGTTGGCCATGCGTAAGGTG
GATGTGTTGAGGGCTGTGGTTTATCTGCTCGCTCAGTGTTTGGGTGGGATCCTCGCAGCT
GGCCTCATGTACCTCTCTCTGCCCCTGAAGTCCACAGCACAGAACTTCATCAACAAGGTC
CCAGTGGATATGAACGCAGGTCAAGCTCTTGGGATGGAGATGCTTGCCACGTTTCTCCTG
GGGTTCACTGTATTTTCTGTGGAAGATCAACGCAGGAGAGAAATAAATGAACCAGGGAAT
TTGGCCATTGGATTTGCTGTGACCACTGCCATCTTTATCGCTGGGAGGTTTTCTGGTGCC
AGCCTGAACCCAGCTCGCTCTCTTGGTCCCGCTATAATACTTGGATATTGGGAACATCAC
TGGGTCTACTGGATTGGTCCAATATTAGGTGCAGTCCTGGCTGGAGTGTCTCATGAGTTC
ATCTTTGCCCCCAGTGCGTCCAGACAGAAGCTGGTGGCATGTTTGACCTGCAAGGACATT
GAGATTGTGGAGACGGCCAGCGTGTCTCGATCGTCCCTGTCCACTGTCACACAGAGCGCC
ATGAGAAACAAACAAAGCAACAAA---CTGGAGCACAGC
>MN168476
GAGCTGAGGAGCCGTCAGTTCTGGCAGGGGATTCTGGCAGAAACTCTTGGTTCTCTGGTC
TTTGTATCTGCTGTGTTGGGCTCTTTAGTGCCGGGGCCGGACGGGGCCTCCCCGGGGCCC
ATTTACCCCGCACTGGCTGCTGGTATGGCAACTGTGGTTCTGGGATATTGCTTTGGTGAA
ATCAGTGGGGCTCAGGTGAATCCCGCGGTGACTGTGGCCCTGTTGGCCATGCGTAAGGTG
GATGTGTTGAGGGCTGTGGTTTATCTGCTCGCTCAGTGTTTGGGTGGGATCCTCGCAGCT
GGCCTCATGTACCTCTCTCTGCCCTTGAAGTCCACAGCACAGAACTTCATCAACAAGGTT

--GGGAGATTTTCTGGTGCC
AGCCTGAACCCAGCTCGCTCTCTTGGTCCCGCTATAATACTTGGATATTGGGAACATCAC
TGGGTATACTGGATTGGTCCAATATTAGGTGCAGTCCTGGCTGGAGTGTCTCATGAGTTC
ATCTTTGCCCCCAGTGCGTCCAGACAGAAGCTGGTGGCTTGTTTGACCTGCAAGGACATT
GAGATTGTGGAGACGGCCAGCGTGTCTCGATCGTCCCTCTCCACTGTCACACAGAGCGCC
ATGAGAAACAAACAAAGCAACAAA---CTGGAGCACAGC
>MN168477
GAGCTGAGGAGCCGTCAGTTCTGGCAGGGGATTCTGGCAGAAACTCTTGGTTCTCTGATC
TTTGTATCTGCTGTGTTGGGCTCTTTAGTGCCGGGGCCAGACGGGGCCTCCCCGGGGCCC
ATTTACCCCGCATTGGCTGCTGGTATGGCAACTGTGGTTCTGGGATATTGCTTTGGTGAA
ATCAGTGGGGCTCAGGTGAATCCTGCGGTGACTGTGGCCCTGTTGGCCATGCGTAAGGTG
GATGTGTTGAGGGCTGTGGTGTATCTGCTCGCTCAGTGTTTGGGTGGGATCCTCGCAGCT
GGCCTCATGTACCTCTCTCTGCCCCTGAAGTCCACGGCACAGAACTTCATCAACAAGGTC
CCAGTGGATATGAACGCAGGTCAAGCTCTGGGGATGGAGATGCTTGCCACGTTTCTCCTG
AGTTTCACCGTGTTTTCAGTGGAAGATCAGCGCAGGAGAGATATAAATGAACCAGGGAAT
TTGGCCATTGGGTTTGCTGTGACCACGGCCATCTTTATCGCTGGGAGGTTTTCTGGTGCC
AGCCTGAACCCAGCTCGCTCTCTTGGTCCCGCTATAATACTTGGATATTGGGAACATCAC
TGGGTATACTGGATCGGTCCAATCTTAGGTGCAGTGCTGGCTGGAGTGTCTCATGAGTTC
ATTTTTGCTCCCAGTGCGTCCAGACAGAAGCTGGTGGCTTGTTTGACCTGTAAGGACATT
GAGATTGTGGAGACGGCCAGCGTGTCTCGATCGTCCCTGTCCACTGTCACACAGAGCGCC
ATGAGAAACAAACAAAGCAACAAG---CTGGAGCACAGC
>MN168478
GAGCTGAGGAGCCGTCAGTTCTGGCAGGGGATTCTGGCAGAAACTCTTGGTTCTCTGATC
TTTGTATCTGCTGTGTTGGGCTCTTTAGTGCCGGGGCCGGACGGGGCCTCCCCGGGGCCC
ATTTACCCCGCACTGGCTGCTGGTATGGCGACTGTGGTTCTGGGATATTGCTTTGGTGAA
ATCAGTGGGGCTCAGGTGAATCCTGCGGTGACTGTGGCCCTGTTGGCCATGCGTAAGGTG
GATGTGTTGAGGGCTGTGGTGTATCTGCTCGCCCAGTGTTTGGGTGGGATCCTCGCGGCT
GGCCTCATGTACCTCTCTCTGCCCCTGAAGACCACGGCACAGAACTTCATCAACAAGGTC
CCAGTGGATATGAACGCAGGTCAAGCTCTCGGGATGGAGATGCTTGCCACGTTTCTCCTG
GGTTTCACCGTATTTTCAGTGGAAGATCAACGCAGGAGAGAAGTAAATGAACCAGGGAAT

CTGGCCATTGGATTTGCTGTGACCACTGCCATCTTCATCGCTGGGAGGTTTTCTGGAGCC
AGCCTGAACCCTGCTCGCTCTCTTGGTCCCGCTATAATACTTGGATATTGGGAACATCAC
TGGGTCTACTGGATCGGTCCAATCCTAGGTGCAGTGCTGGCTGGAGTGTGTCACGAGTTC
ATTTTTGCCCCCAGTGCGTCCAGGCAGAAGCTGGTGGCTTGTTTGACCTGTAAGGACATT
GAGATTGTGGAGATGGCCAGCGTGTCTCGATCATCCCTGTCCACTGTCACACAGAGCGCC
ATGAGAAACAAACAAAGCAACAAA---CTGGAGCACAGC
>MN168479
GAGCTGAGGAGCCGTCAGTTCTGGCAGGGGATTCTGGCAGAGACTCTTGGTTCCTTGGTC
TTTGTATCTGCTGTGCTGGGCTCTTTAGTGCCGGGGCCGGACGGGGCCTCCCCGGGGCCC
ATTTACCCCGCACTGGCTGCTGGTATGGCGACTGTGGTTCTGGGATATTGCTTTGGTGAA
ATCAGTGGGGCTCAGGTGAATCCTGCGGTGACTATGGCTCTCTTGGCCACGCGTAAGGTG
GATGTGTTGAGGGCTGTGGTGTATCTGCTCGCTCAGTGTTTGGGTGGGATCCTCGCGGCT
GGCCTCATGTACCTCTCGCTGCCCCTGAAGTCCACGGCACAGAACTACATCAACAAGGTC
CCTGTGGAAATGAACGCAGGTCAAGCTCTTGGGATGGAGATGCTTGCCACGTTTCTCCTG
GGTTTCACTGTATTTTCTGTGGAAGATCAACGCAGGAGAGAAATAAACGAACCAGGGAAT
TTAGCCATTGGGTTTGCTGTGACCACTGCCATCTTTATTGCTGGGAGGTTTTCTGGTGCC
AGTCTGAACCCAGCTCGCTCTCTTGGTCCTGCTATAATACTTGGATATTGGGAACATCAC
TGGGTCTACTGGATCGGTCCAATCTTAGGTGCAGTGCTGGCTGGAGTGTCTCATGAGTTC
ATCTTTGCCCCCAGTGCGTGCAGACAGAAGCTGGTGGCTTGTTTGACCTGTAAGGACATT
GAGATTGTGGAGACGGCCAGCGTGTCTCGATCGTCCCTGTCCACTGTCACACAGAGCGCC
ATGAGAAACAAACAAAGCAACAAA---CTGGAGCACAGC
>MN168480
GAGCTGAGGAGCCGTCAGTTCTGGCAGGGGATTCTGGCAGAGACTCTTGGTTCCCTGGTC
TTTGTATCTGCTGTGCTGGGCTCTTTAGTGCCGGGGCCGGACGGGGCCTCCCCGGGGCCC

---------------GTGAATCCTGCGGTGACTGTGGCTCTCTTGGCCACGCGTAAGGTG
GATGTGTTGAGGGCCGTGGTGTATCTGCTAGCTCAGTGTTTGGGTGGGATCCTCGCGGCT
GGCCTCATGTACCTCTCGCTGCCCCTGAAGTCCACGGCACAGAACTACATCAACAAGGTC
CCAGTGGAAATGAACACAGGTCAAGCTCTTGGGATGGAGATGCTTGCCACGTTTCTCCTG
GGTTTCACTGTATTTTCTGTGGAAGATCAACGCAGGAGAGAAATAAATGAACCAGGGAAT
TTAGCCATTGGGTTTGCTGTGACCACTGCCATCTTTATTGCTGGGAGGTTTTCTGGTGCC
AGTCTGAACCCAGCTCGCTCTCTTGGTCCCGCTATAATACTTGGATATTGGGAACATCAC
TGGGTATACTGGATCGGTCCAATCTTAGGTGCAGTGCTGGCTGGAGTGTCTCATGAGTTC
ATCTTTGCCCCCAGTGCGTCCAGACAGAAGCTGGTGGCTTGTTTGACCTGTAAGGACATT
GAGATTGTGGAGACGGCCAGCGTGTCTCGATCGTCCCTGTCCACTGTCACACAGAGCGCC
ATGAGAAACAAACAAAGCAACAAA---CTGGAGCACAGC
>MN168481
GAGCTGAGGAGCCGTCAGTTCTGGCAGGGGATTCTGGCAGAGACTCTTGGTTCCCTGGTC
TTTGTATCTGCTGTGCTGGGCTCTTTAGTGCCGGGGCCGGACGGGGCCTCTCCGGGGCCC
ATTTACCCCGCACTGGCTGCTGGTATGGCGACTGTGGTTCTGGGATATTGCTTTGGTGAA
ATCAGTGGGGCTCAGGTGAATCCTGCGGTGACTGTGGCTCTCTTGGCCACGCGTAAGGTG
GATGTGTTGAGGGCTGTGGTGTATCTGCTCGCTCAGTGTTTGGGTGGGATCCTCGCGGCT
GGCCTCATGTACCTCTCGCTGCCCCTGAAGTCCACAGCACAGAACTACATCAACAAGGTC
CCAGTGGAAATGAACGCAGGTCAAGCTCTTGGGATGGAGATGCTTGCCACGTTTCTCCTG
GGTTTCACTGTATTTTCTGTGGAAGATCAACGCAGGAGAGAAATAAATGAACCAGGGAAT
TTAGCCATTGGGTTTGCTGTGACCACTGCCATCTTTATTGCTGGCAGGTTTTCTGGTGCC
AGTCTGAACCCAGCTCGCTCTCTTGGTCCCGCTATAATACTTGGATATTGGGAACATCAC
TGGGTATACTGGATCGGTCCAATCTTAGGTGCAGTGCTGGCTGGAGTGTCTCATGAGTTC
ATCTTTGCCCCCAGTGCGTCCAGACAGAAGCTGGTGGCTTGTTTGACCTGTAAGGACATT
GAGATTGTGGAGACGGCCAGCGTGTCTCGATCGTCCCTGTCCACTGTCACACAAAGCGCC
ATGAGAAACAAACAAGGCAACAAA---CTGGAGCACAGC

>MN168482
GAGCTGAGGAGCCGTCAGTTCTGGCAGGGGATTCTGGCAGAAACTCTTGGTTCTCTGATC
TTTGTATCTGCTGTGTTGGGCTCTTTAGTGCCGGGGCCGGACGGGGCCTCCCCGGGGCCC
ATTTACCCCGCATTGGCTGCTGGTATGGCAACTGTGGTTCTGGGATATTGCTTTGGTGAA
ATCAGTGGGGCTCAGGTGAATCCTGCGGTGACTGTGGCTCTCTTGGCCACTCGTAAGGTG
GATGTGTTGAGGGCTGTGGTGTATCTGCTCGCTCAGTGTTTGGGTGGGATCCTCGCAGCT
GGCCTCATGTACCTCTCGCTGCCCCTGAAGTCCACAGCACAGAACTACATCAACAAGGTC
CCAGTGGAAATGAACGCAGGTCAAGCTCTTGGGATGGAGATGCTTGCCACGTTTCTCCTG
GGTTTCACCGTATTTTCTGTGGAAGATCAACGCAGGAGAGAAATAAATGAACCAGGGAAT
TTAGCCATTGGGTTCGCTGTGACCACTGCCATCTTTATCGCTGGAAGGTTTTCTGGTGCC
AGCTTGAACCCAGCTCGCTCTCTTGGTCCCGCTATAATACTTGGATATTGGGAACATCAC
TGGGTATACTGGATCGGTCCAATCTTCGGTGCAGTGCTGGCTGGAGTGTCTCATGAGTTC
ATTTTTGCCCCCAGTGCATCCAGACAGAAGCTGATGGCTTGTTTGACCTGTAAGGACATT
GAGATTGTGGAGATGGCCAGCGTGTCTCGATCGTCCCTGTCCACTGTCACACAGAGCGCC
ATGAGAAACAAACAAAGCAACAAA---CTGGAGCACAGC
>MN168483
GAGCTGAGGAGCCGTCAGTTTTGGCAGGGGATTCTGGCAGAGCTTCTTGGTTCCCTGATC
TTTGTATCTGCTGTGTTGGGCTCTTTAGTGCCGGGGCCGGACGGGGCCTCCCCGGGGCCC
ATCTACCCCGCACTGGCTGCCGGTATGGCAACTGTGGTTCTGGGATATTGCTTTGGTGAA
ATCAGTGGGGCTCAGGTAAATCCTGCAGTGACTGTGGCGCTCTTGGCCACGCGTAAGGTG
GATGTGTTCAGGGCGCTGGTGTATCTGGTGGCCCAGTGTTTGGGGGGGATCCTCGCCACG
GGCCTCATGTACCTGTCGCTGCCCCTGAAGTCCACCGCACAGAACTACATCAACAAGGTC
CCAGTGGACATGAACGCAGGTCAAGCTCTCGTGATGGAGATGCTTGCCACGTTTGTCCTG
GGCTTCACCGTATTTTCTGTGGAAGATCAACGCAGGAGAGAAATAAACGAACCTGGAAAC
TTAGCCATTGGGTTTGCTGTAACCACTGCAATCTTTATTGCTGGGAGGTTTTCTGGTGCC
AGCTTGAATCCGGCTCGCTCCCTTGGTCCCGCTATAATACTTGGATATTGGGAACACCAC
TGGGTATACTGGATCGGGCCAATATTAGGTGCAGTCCTGGCTGGACTATCCCACGAGTTT
ATTTTCGCCCCCAGTGCGTCCAGACAGAAGCTGGTGGCTTGCTTGACCTGTAAGGACATT
GAGATCGTGGAGACGGCCAGCGTGTCTCGATCATCCCTGTCCACCGTCACACAGAGCGCC
ATGAGAAACAAGCAAAGCAATAAA---CTGGAGCACAGC
>MN168484
GAGCTGAGGAGCCGTCAGTTTTGGCAGGGGATTCTGGCAGAGCTTCTTGGTTCCCTGATC
TTTGTATCTGCTGTGTTGGGTTCTTTGGTGCCGGGGCCGGACGGGGCCTCCCCGGGGCCC
ATCTACCCTGCACTGGCTGCCGGTATGGCAACTGTGGTCCTGGGATATTGCTTTGGTGAA
ATCAGTGGGGCTCAGGTCAATCCTGCAGTGACTGTGGCGCTCTTGGCCACGCGTAAGGTG
GATGTGTTCAGGGCACTGGTGTATCTGCTGGCCCAGTGTTTGGGGGGGATCCTCGCCACT
GGCCTCATGTACCTGTCGCTGCCCCTGAAGTCCACCGCACAGAACTACATCAACAAGGTC
CCGGTGGACATGAACGCAGGCCAAGCTCTTGTGATGGAGATGCTTGCCACGTTTGTCCTG
GGCTTCACCGTATTTTCTGTGGAAGATCAACGCAGGAGAGAAATAAACGAACCTGGGAAC
TTAGCCATTGGGTTTGCTGTAATCACTGCAATCTTCATTGCTGGGAGGTTTTCTGGTGCC
AGCTTGAATCCGGCTCGCTCCCTCGGCCCCGCTATAATCCTTGGCTATTGGGAACATCAC
TGGGTATACTGGATCGGGCCAATATTAGGTGCAGTCCTGGCTGGACTGTCCCACGAGTTT
ATTTTTGCCCCCAGTGCGTCCAGACAGAAATTGGTGGCTTGTTTGACCTGTAAGGACATT
GAGATTGTGGAGACGGCCAGCGTGTCTCGATCGTCCCTGTCCACCGTCACACAGAGCGCC
ATGAGAAACAAGCAAAGCAACAAA---CTGGAGCACAGC
>MN168485
GAGCTGAGGAGCCGTCAGTTTTGGCAGGGGATTTTGGCAGAGGTTCTTGGTTCCTTGGTC
TTTGTATCCGCTGTGTTGGGCTCTTTAGTGCCGGGGCCGGATGGGGCCTCCCCGGGGCCC
ATCTACCCCGCACTGGCTGCTGGTATGGCAACTGTGGTTCTGGGATATTGCTTTGGTGAA
ATCAGTGGGGCTCAGGTGAATCCTGCAGTGACTGTGGCGCTCTTGGCCACGCGTAAAGTG
GATGTGTTCAGGGCGCTGGTTTATCTGGTTGCCCAGTGTTTGGGGGGGATCCTCGCCACT

GGCCTTATGTACCTGACGCTGCCCCTAAAGTCCACCGCACAGAACTACATCAACAAGGTC
CCAGTGGAAATGAACGCAGGTCAAGCTCTTGTGATGGAGATGCTTGCCACGTTTGTCCTG
GGGTTCACTGTATTTTCTGTGGAAGATCAACGCAGGAGAGAAATAAATGAACCTGGAAAC
TTAGCCATTGGGTTTGCTGTAACCACTGCAATCTTTATCGCTGGGAGATTTTCTGGTGCC
AGCTTGAATCCAGCTCGCTCCCTTGGTCCCGCTATAATACTTGGATATTGGGAACACCAC
TGGGTATACTGGATTGGGCCAATATTAGGTGCAGTCCTGGCTGGAGTATCCCATGAGTTT
ATTTTTGCCCCCAGTGCATCCAGACAGAAGCTAGTGGCTTGTCTGACCTGTAAGGACATT
GAGATTGTGGAGACGGCCAGCGTGTCTCGATCGTCCCTGTCCACTGTCACACAGAGCGCC
ATGAGAAACAAACAAAGCAACAAA---CTGGAGCACAGC
>MN168486
GAGCTGCGGAGCCGTCAGTTTTGGCAGGGGATCCTGGCAGAGGTTCTTGGTTCTCTGGTG
TTTGTATCTGCTGTTTTGGGCTCTTTAGTGCCGGGGCCGGATGGGGTGTCCCCGGGACCC
ATCTACCCTGCACTGGCTGCTGGTATGGCAACTGTGGTTCTGGGATATTGCTTTGGTGAA
ATCAGCGGGGCTCAGGTGAATCCCGCAGTGACGGTGGCTCTCTTGGCCACCCGTAAGGTG
GATGTGTTGAGGGCTGTGGTGTATCTGGTGGCTCAGTGTTTGGGTGGGATCCTCGCAACT
GGCCTCATGTACCTCTCACTGCCTCTGAAGTCAACCGCACAGAACTACATCAACAAGGTC
CCAGTGGAGATGAACGCAGGTCAAGCTCTTGGAATGGAGATGCTTGCTACGTTTCTTCTG
GGTTTCACTGTGTTTTCTGTGGAAGATCAACGCAGGAGAGAAATAAATGAACCTGGAAAC
TTAGCCATTGGCCTTGCTGTAACCACTGCGATCTTTATTGCTGGGAGATTTTCTGGTGCC
AGCTTGAATCCCGCTCGCTCCCTTGGTCCTGCTATAATACTGGGATATTGGGAACATCAC
TGGGTGTACTGGATCGGGCCAATATTAGGTGCAGTCCTAGCTGGAGTCTCTCATGAGTTT
ATTTTCGCACCGAGCGCATCCAGACAGAAGCTCGTGGCCTGCATGACCTGTAAGGACATT
GAGATAGTGGAAACGGCCAGTGTGTCTCGATCGTCACTGTCCACAGTTACACAGAGCGCC
ATGAGACACAAACAAAACAACAAG---CTGGAGCACAGC
>MN168487
GAGCTGAGGAGCCGTCAGTTTTGGCAGGGGATTCTGGCAGAGACTCTTGGTTCTCTGGTC
TTTGTATCTGCTGTGTTGGGCTCTTTAGTGCCGGGGCCAGACGGGGCCTCCCCCGGGCCC
ATTTACCCCGCACTGGCTGCTGGTATGGCAACTGTGGTTCTGGGATATTGCTTTGGTGAA
ATCAGTGGCGCTCAGGTGAATCCTGCGGTGACTGTGGCTCTCTTGGCCACACGTAAGGTG
GATGTGTTGAGGGCCGTGGTGTATCTGGTTGCTCAGTGTTTGGGTGGGATCCTCGCAGCT
GGCCTCATGTACCTCTCACTGCCCCTGAAGTCCACAGCACAAAACTACATCAACAAGGTC
CCAGTGGAAATGAATGCAGGTCAAGCTCTGGGGATGGAGATGCTTGCCACATTTCTCCTG
GGTTTCACTGTATTTTCTGTGGAAGATCAACGCAGGAGAGAAATAAATGAACCAGGGAAT
TTAGCCATTGGGTTTGCTGTGACCACTGCCATCTTTATTGCTGGGAGATTTTCTGGTGCC
AGTCTGAACCCGGCTCGCTCCCTTGGTCCTGCTATAATACTTGGATATTGGGAACATCAC
TGGGTATACTGGATTGGTCCAATATTAGGTGCAGTTCTGGCTGGAGTGTCTCATGAGTTC
ATTTTTGCCCCCAGTGCATCCAGACAGAAGCTGGTGGCTTGTTTGACCTGTAAGGACATT
GAGATTGTGGAGACGGCCAGCGTGTCTCGATCATCCCTGTCCACAGTCACACAGAGCGCC
ATGAGAAACAAACAAAACAACAAA---CTGGAGCACAGC
>MN168488
GAGGTGAAAAACCCTAAGTTTTGGCGGGGCATACTGGCCGAGATTATAGGCTCTCTGGTC
TTTGTATCAGTTGTATTGGGCTCTTCACTATCAGGGCTCGAGGGTGTCTCTTCTGGACCT
TTGTATCCAGCACTGGCAGCAGGTATGGCAGCAGTTGGCCTAGGACATTGCTTTAGAAAG
ACCAGTGGGGCTCAGGTGAACCCTGCTTTAACATTGGCCTTATTGGCTACACGGAAACTG
GATGCTTTAAAGGCCATGGTCTATGTGTTTGCCCAGTGTTTAGGAGCCACTGTAGGAGCT
GGGATTCTCTACTTGGTCCTACCCTTAAAATCCACAGCTAAGATCTATGTCAATAAGGTA
CCAATGGAAGGAAATGCAGGGCAGGCTCTGGGGATGGAGGTACTTGTCACATTCCAATTG
GTCTTTACCATCTTCTCAGTGGAGGCTCAGCGCAAGAGAGAGGAATGTGAACCTGGCAAC
CTGGCCATTGGCTGCTCCCTTAGTGCAGGGATTTTTACTGCAGGTGGAATTTCTGGAGGC
AGCATGAACCCTGCGAGATCTCTTGGACCTGCAATTATAGTTGGATACTGGGAACACCAC
TGGGTTTATTGGATTGGACCAGTGTTGGGTGCTGTGTTAGCTGGTGTGGCTCATGAATTC

TTTTTCGCTGCAAGTGCCTCAAGGCAGAAGCTGGTGTCCTGTCTCACCTGTAAGGACATT
GAGATATTCGAGACAGCCAGTATGTCCCGTTCATCTCTGTCAACCATCACACAGACAGCT
GTGAGGACCAAGCAGAGTGACAAA---CTCGACCACAGC
>MN168489
GAGGTGAAAAATCCTAAATTTTGGCAGGGCATACTGGCCGAGATGATAGGTTCTTTGATC
TTTGTATCAGTTGTATTGGGCTCTTCACTGTCAGAGCTCGAAGATATGTGTTCTGGACCT
TTGTACCCAGCACTGGCAGCAGGTATGGCAGCAGTTGGCCTAGGACATTGCTTTAGAAAT
ATCAGTGGTGCTCAGGTGAACCCTGCATTAACTTTGGCCTTATTAGCTACCCGGAAATTG
GACACTTTAAAGGCCATGGTCTATGTGTTTGCCCAGTGTTTAGGAGCCACTGTAGGAGCT
TGGATTCTCTACTTGGTCCTACCCATAAAATCCACAGCCAATATTTATGTCAACAAGGTA
TCAGTGGAGGGAAATGCAGGGCAGGCTCTGGGGATGGAGGTGCTTGTCACATTTCAGTTG
GTCTTTACCATTTTTTCAGTTGAGGATCAACGCAAGAGAGAGGAATGTGAACCTGGCAAC
TTGGCCATTGGCTGTTCCCTTAGTGCAGGGATTTTTACTGCAGGTAGGATTTCCGGAGGC
AGTATGAACCCTGCTAGGTCTCTTGGACCGGCAATTATAGTTGGATACTGGGAACACCAC
TGGGTGTATTGGATTGGGCCAGTGTTGGGTGCTGTGTTAGCTGGCATGGCTCATGAATTC
TTTTTCGCTCCAAGTGCCTCAAGGCAGAAGCTGGTGTCCTGTCTCACCTGTAAGGACATT
GAGATATTAGAGACAGCCAGTGTGTCCCGTTCATCTCTGTCAACCATCACACAGACAGCT
GTGAGGGCCAAACAGAGTGACAAA---CTTGACCAAAGC
>MN168490
GAGGTGAGAAACCCAAAGTTTTGGAGGGGCATACTGGCAGAGATGATGGGCTCTCTAGTC
TTTGTATCAGTTGTATTAGGCTCTTCACTATCAGGGCATGAGGGTGTCTCTTCTGGACCT
TTGTACCCAGCACTGGCAGCAGGTATGGTAGCGGTTGGTCTAGGACATTGCTTTAGAAAG
ATCAGTGGGGCACAGGTGAACCCTGCATTAACTTTGGCCTTATTAGCTACACGGAAACTG
GACGCTTTAAAAGCCGTGGTCTATGTATTTGCACAGTGTTTAGGAGCCACTGTAGGAGCT
GGGATTCTCTACATGGTCCTACCCTTAAAATCCACAGCTAAAATCTATGTCAACAAGGTA
CCAATGGAGGGTAATGCAGGGCAGGCTCTGGGGATGGAGATACTTGTAACCTTTCAACTG
GTCTTTACCATCTTCTCAGTGGAGGATCAGAGAAAGAGCGAGGAATGTGAACCTGGAAAT
TTGGCAATTGGCTGCTCCCTTAGTGCAGGAATTTTCACTGCAGGTAGAATTTCTGGAGGC
AGCATGAACCCTGCTAGGTCTCTTGGACCTGCAATTATAGTTGGATACTGGGAACACCAC
TGGGTATACTGGATTGGGCCAGTGTTGGGTGCTGTGTTCGCTGCCATGGCTCATGAATTC
TTTTTCGCTTCAAGTGCCTCAAGGCAGAAGTTAGTGTCCTGTCTCACCTGTAAGGACATT
GAGATATTAGAGACAGCCAGCGTGTCCCATTCATCTTTGTCGACCATCACACAGACAGCT
GGGAGGGCCAAGCAAAGTGACAAA---CTTGACCACAGC
>MN168491
GAGATGAGAAACCCTAAGTTTTGGCGGGGAATACTGGCTGAGATAATAGGCTCTCTGGTT
TTTGTATCAGTTGTATTGGGCTCATCACTATCAGGGCTTGAGGGTGTCTCTTCTGGACCT
TTGTACCCAGCGCTGGCAGCAGGTATGGCTGCGGTCTGCCTAGGACATTCTTTTAAAAAG
ATCAGTGGCGCTCAGGTGAACCCTGCTTTAACTTTGGCTTTATTAGCTACACGGAAACTG
GATGCTTTAAAGGCGATGGTCTATGTGTTTGCCCAGTGTTTAGGAGCGACTGTAGGAGCT
GGGATTCTGTACTTGGTCTTACCGTTAAAATCCACAGCTAATATTTTTGTCAACAAGGTA
CCAATGGAGGGAAATGCAGGGCAGGCTCTGGGGATGGAGGTACTTGTTACCTTCCAATTG
GTCTTTACCATCTTCTCAGTAGAGGATCAACGCCAGAGAGAGGAATGTGAAACTGCAAAC
TTGGCCATTGGCTGCTCCATTAGTGCAGGGATTTTTACTGCAGGTAAAATCTCTGGAGGC
AGCATGAACCCTGCTAGGTCTCTTGGACCTGCAATCATAGTTGGATACTGGGAGCACCAT
TGGGTGTACTGGATTGGGCCAGTGTTGGGTGCTGTGTTAGCTGGCATGGCTCATGAATTC
TTTTTCGCTCCAAGTGCCTCAAGGCAGAAGCTGGTATCATGTATCACCTGTAAAGACATT
GAGATGTTAGACACAGCTAGCATGTCCCATTCATCTCTGTCAACCATCACACAGACAGCT
GTGAGAGCCAAGCAGAGCGGCAAA---CTTGACCACAGC
>MN168492
GAGGTAAGAAACCCTAAGTTTTGGAGGGGCATACTGGCCGAGATGATAGGCTCTCTGGTC
TTTGTATCAGTTGTATTGGGCTCTTCACTATCAGGGCTCGAGGGCGTCTCTTCTGGACCT

TTGTACCCAGCACTGGCAGCAGGTATGGCAGCAGTTGGCCTAGGACATTGCTTTAGAAAA
ATCAGTGGGGCTCAGGTGAATCCTGCTTTAACTTTGGCCTTATTAGCTACACGGAAACTG
GATGCTTTAAAGGCCATGGTCTATGTGTTTGCCCAGTGTTTAGGAGCCACTATAGGAGCT
GGAATTCTCTACTTGGTCCTACCCTTAAAATCCACAGCTAAGATCTATGTCAACAAGGTA
CCAATGGAGGGAAATGCAGGGCAGGCTCTGGGGATGGAGGTACTCGTCACCTTCCAGTTG
GTCTTTACCATCTTCTCAGTGGAGGATCAGCGCAAGAGAGAGGACTATGAACCTGGCAAC
TTGGCCATTGGCTGTTCCCTTAGTGCAGGGATTTTTACTGCAGGTAGAATTTCTGGAGGC
AGCATGAACCCTGCTAGGTCTCTTGGACCTGCAATTATAGTTGGATACTGGGAACACCAC
TGGGTGTATTGGATTGGGCCAGTGTTGGGTGCTGTGTTAGCTGGCGTGGCTCATGAATTC
TTTTTCGCTCCAAGTTCCTCAAGGCAGAAGCTGGTGTCCTGTCTCACCTGTAAGGACATT
GAGATATTAGAGACAGCCAGCATGTCCCGTTCATCTCTGTCGACCATCACGCAGACAGCT
GTGAGAGCCAAGCAGAGTGACAAA---CTCGACCACAGC
>MN168493
GAGGTCAGAAGCCGTCAGTTTTGGCGGGGCATGCTGGCTGAGATCCTAGGCTCTCTGATG
TTTGTGTCAGCTGTGTTGGGCTCGTCAATACCAGGTCCTGAGGGTACTTCCTCTGGCCCT
CTGTACCCAGCACTAGCTGCAGGCATGGCAGCTGTGGGCCTGGGACATTGTTTTGGAGAG
ATCAGTGGAGCTCAGGTGAACCCTGCTGTAACATTGGCTTTATTGGCTACAAGGAGACTG
GACACATTAAGGGCTGTGTTCTATGTCACTGCCCAGTGCTTAGGGGCCACTTTAGGGGCT
GGGATCCTCTACTTGGTTCTACCCCTGAAATCCACAGCTGAGATCTATGTCAACAAGGTA
CCCACAGAGGTGAACGCAGGGCAGGCTCTGGGGACAGAGATACTGGCCACCTTCCAGTTG
GTCTTCACCATCTTCTCTGTGGAGGATTATCGAAGGAGAGAGGGGAGTGAACCTGGCAAC
CTGGCTATTGGCTGCTCCCTATCTGCTGGCATTTTCACAGCTGGTAGAATTTCTGGAGGT
AGCATGAACCCTGCTCGTTCTCTCGGACCTGCCATTATAGTTGGGTTCTGGGAACATCAC
TGGGTGTACTGGATAGGTCCAGTCCTGGGTGCAGTTCTGGCTGGTGTGGCTCACGAATTC
TTCTTTGCTCCAACTGCCTCAAGACAGAAGTTGGTGGCATGTCTCACATGTAAAGACATT
GAAATAGTGGAGACTGCCAGTGTGTCTCGGTCCTCTCTGTCCACTGTCACACAAACAGCT
ATGAGGGTCAAGCAGACCAACAAA---CTCGAACACAGC
>MN168494
GAGGTCAGAAGCCGTCAGTTTTGGCGGGGCATGCTGGCTGAGATCCTAGGCTCTCTGGTC
TTCGTGTCAGCTGTGTTGGGCTCTTCAGTGCCAGGTCCTGGGAGCAATTCCTCCGGGCCC
CTTTACCCGGCACTAGCTGCAGGCATGGCAGCAGTGGGCCTGGGACACTGTTTTGGAGAG
ATCAGCGGGGCTCAGGTGAACCCTGCTGTAACTTTGGCTTTATTGGCTACAAGGAGACTG
GACACATTAAGGGCTGTGGTCTATGTCATTGCACAATGCTTAGGAGCCACTCTAGGGGCT
GGGATGCTCTACTTGGTCCTGCCCCTGAAATCCACAGCTGAGGTCTTTGTCAACAAGGTA
CCCATAGAGGTGAACGCAGGGCAGGCTCTGGGGACAGAGATGCTGGCCACCTTCCAGCTG
GTCTTCACCATCTTCTCTGTGGAAGATCAGCGGAGGCGAGAGGGGAGTGAACCCGGCAAC
CTGGCTATTGGCTGCTCCCTCTCTGCTGGCATTTTTACAGCTGGTAGAATTTCTGGAGGC
AGCATGAACCCTGCTCGTTCTCTTGGACCTGCCATTATAGTTGGGTTCTGGGAACATCAC
TGGGTGTACTGGATAGGGCCGGTGCTGGGTGCAGTTCTGGCAGGTGTGTCTCATGATTTC
TTTTTTGCTCCAACCACCTCAAGGCAGAAGCTGGTGGCATGTCTCACTTGTAAAGACATC
GAAATAGTGGAGACTGCCAGTGTGTCTCGCTCCTCTCTGTCCACTGTCACACAGACAGCT
ATGAGGGCCAAGCAGACCAACAAA---CTTGAACACAAC
>MN168495
GAGGTAAGGAGCCGTCAGTTTTGGCGGGCCATGCTGGCTGAGATATTAGGCTCTTTGATC
TTCATATCGGCTGTGTTGGGCTCTTCACTGCGAGGGCCTGATGATGCTTCCTCTGGGCCT
CTGTACCCAGCACTGGCTGCAGGCATGGCAGCAGTTGGTCTGGGACACTGCTTTGGAGAA
ATCAGTGGGGCGCAGGTGAACCCTGCTGTAACTCTTGCTTTGTTGACTACACGAAGACTG
GATGTGCTAAGGGCGGTGGTCTATGTCTTTGCCCAGTGCTTAGGGGCCACTCTAGGGGCT
GGAATCCTCTACTTGGTCCTACCTTTGAAATCTACAGCAGAGATCTATGTCAACAAGGTA
CCCATGGAGGTGAACGCAGGGCAGGCTTTGGGGATAGAGATGCTAGCTACCTTTCAGCTG
GTATTCACCATCTTTTCTGTGGAGGACCTGCGCAGGAGAGAGGGGAGTGAGCCTGGCAAC

CTGGCCATCGGCTGTTCTGTCAGTGTTGGCATCTTTACTGCCGGAAGAATCTCTGGTGGC
AGTATGAACCCTGCACGCTCTCTTGGACCTGCCATTGTAGTTGGATACTGGGAACATCAC
TGGGTATACTGGATTGGGCCAGTGTTGGGCGCAGTGTTGGCCGGGGTGTCTCATGAGTTC
TTTTTTGCCCCGAGTGCTTCAAGGCAAAAGCTGCTGGCATGTCTCACCTGTAAGGACATT
GAGATCATGGAGACAGCCAGTGTGTCCCGCTCATCTCTGTCTACTGTCACACAGACAGCA
ATGAGAGCCAAGCAGACGAATAAA---CTTGAGCACAAC
>MN168496
GAGGTGAGGAGTCGGCAGTTTTGGCGAAGCATGCTGGCTGAGATACTGGCCTCCTTGGTC
TTTGTATCGGCTGTCTTAGGCTCATCACTGCCAGGATCTGAAGGTCCCTCCCTTGGGCCT
ATCTACCCAGCACTCGCAGCAGGAATGGCAACGGTTGGTCTGGGACATTGCTTCGGAGAA
ATCAGTGGCGCCCAGGTAAACCCTGCTGTTACTCTGGCTTTACTGGCCACAAGGAGACTG
GATGTGCTGAGGGCAGTGGTGTATGTTGTTGCCCAGTGCTTGGGGGCGATTTTGGGGGCT
GGAATATTTTACCTGGTTCTACCATTAAAATTCACAGCTGAGATCTATGTTAACAAGGTT
CCTATGGAGGTGAATGCAGGGCAAGCCTTGGGGGCTGAGATGCTTGCCACCTTCCAGCTG
GCATTCACTGTCTTTTCTGTAGAGGATCAGCGCAGGAGAGAGGGGAGTGAACCTGGAAAC
ATAGCCATTGGCTTCTCTGTTAGTGCTGGAATTTTTATTGCTGGAAAAATCTCTGGAGGC
TGCCTGAACCCTGCTCGCTCTTTAGGACCTGCAATCATAGCTGGGTACTGGGAACACCAC
TGGGTGTTCTGGATTGGGCCAGTGTTGGGGGCAGTGTTGGCCGGTGTGTCTCACGAGTTC
TTTTTCGCTCCGAGTGCATCCAGACAGAAGCTAGTGGCGTGTCTCACCTGTAAAGACATT
GAGATTGTGGAGACGGCCAGTGTGTCTCGCTCATCTCTGTCCACTGTAACACAGACCGCC
ATGAGGGCCAAGCAGACCAACAAA---CTCGAGCACAAC
>MN168497
GAGCTGCGGAGTCGTCAGTTTTGGCGTGGGATGCTGGCAGAGCTCCTGGGCTCTCTGGTG
TTTGTGTCTGCGGTGTTGGGCTCCTCTCTGCCGGGGCCTGACGGGGCCTCAGGAGGAACC
CTCTATCCAGCACTGGCTGCAGGAATGGCGGCTGTTGGATTGGGGCACTGTTTTGGAGAG
ATCAGTGGGGCTCAGGTCAATCCAGCAGTGACTCTGGCTCTCCTGATCACACGCCGGCTG
GACGTGTTGAGGGCAGCGGTCTATATCTTTGCGCAGTGCTTGGGTGCCATGCTGGGTTCA
GGCATCCTGTACCTGGTCCTCCCACTCACCTCTGGCGCTGAGATCTATGTTAATAAGGTT
CCTCTAGAGGTGAACGCAGGCCAAGCACTGGGGATGGAGATCCTGGCAACCTTCCAGCTT
GTCTTCACCATTTTCTCTGTAGATGACCAACGGCGGAGAGAGAGCAATGAACCAGGCAAC
CTGGCCATCGGCTTCTCACTTAGCGCAGGCATTTTTACAGCTGGGCGCGTCTCTGGGGGC
AGCATGAACCCTGCTCGCTCCCTCGGTCCTGCAATTATAGTGGGTTTCTGGGAACACCAC
TGGGTATACTGGATTGGTCCAGTGTTGGGGGCAGTTCTGGCTGCGGTTTCTCATGAGTTT
TTCTTTGCTCCCAGTGCGTCTAGGCAGAAGCTGGTGGCTTGTCTCACCTGTAAAGACATT
GAGATCATAGAAACAGCCAGTGTGTCTCGTTCTTCTCTGTCCACCGTCACTCAGACAGCC
ATGAGAGCTAAACAAGCCAATAAA---CTAGAGCATAAC
>MN168498
GAGCTGCGGAGTCGTCAATTCTGGCGGGCCACAGTCGCGGAGGTGCTGGGCACGCTGGTG
TTTGTGTCGGCAGTGCTTGGCTCCTCTATGCCAGGGCCAGAGGGCAGTGACGTGGGGCCT
CTGTACCCTGCGCTGGCAGCGGGCATGGTGGCGGTAGGCCTGGGACACTGCTTCGGGGAG
ATCAGTGGAGCCCAGGTAAACCCTGCGGTCACTCTGGCTTTCCTGGCCACCAGGCGCCTG
GAGGCTCTGAGAGCACTGGTGTACGTAGCGGCCCAATGCCTGGGAGCCACTCTGGGCACC
GGGATCCTCTACCTGGCCCTGCCGCAGAAGTCCACCGCAGACTCCTTCGCCAACAAGGTG
CCCATGGAGGTGAACGCAGGCCAGGCGCTGGGGATGGAGATGCTGGCCACCTTCCAGCTG
GTCTTCACCATCTTCTCCGTGGAGGACCAGCGGCGGCAGGAGGGGGCTGAGCCAGGGAAC
CTGGCCATCGGCTTCTCACTGAGCGCAGGGATCTTTACAGCTGGTCGCCTCTCAGGGGGC
AGCATGAATCCTGCCAGGACGCTGGGCCCCGCCATCATCACTGGAGTCTGGGAGCACCAC
TGGGTGTACTGGATTGGGCCGGTGTTAGGCGCCATCCTGGCTGGCGTGTCCCACGAGTTC
TTCTTTGCTCAAAGCGCGTCCAGACAGAAGCTGGTGGCGTGTCTGACATGCAAGGACATC
GAGATGGTGGAGACCGCCAGCGTCTCCCGCTCCTCCCTCTCGACTGTCACGCAGACGGCC
ATGAGGGCCAAGCAGGCCCACAAG---TCTGAGCACAGC

>MN168499
GAGCTGCGGAGTCGTCAGTTCTGGCGGGCCACGACTGCCGAGGTCCTAGGCACTCTTGTG
TTTGTGTCAGCAGTGCTGGGCTCCTCTATGCCAGGGCCGGAGGGCAGTGGAGGTGGGCCC
CTGTACCCTGCACTGGCAGCTGGCATGGTGGCAATAGCCCTGGGACACTGCTTTGGAGAA
ATCAGTGGAGCTCAGGTGAACCCTGCGGTGACTTTGGCTTTCTTGGCCACCCGGCGCCTG
GATGCCATGAGGGCGTTGGTGTATGTGGCGGCCCAGTGCCTGGGAGCCACACTGGGCACA
GGGATCCTCTATCTGGCCCTCCCTCAGAAGTCCACAGCTGACTTCTTTGCCAACAAGGTC
CCCGTGGAGGTGAATGCAGGCCAGGCATTGGGGATGGAGATTCTGGCCACCTTCCAGCTG
GTCTTCACCATCTTCTCTGTGGAGGACCAGCGGCGGAGAGAGGGGGCTGAACCAGGCAAC
CTGGCCATCGGCTTCTCATTAAGCGCAGGGATCTTTACAGCAGGTCGTCTCTCAGGAGGC
AGTATGAATCCTGCCAGAACTCTGGGCCCTGCCATCATCACTGGGATGTGGGAGCACCAC
TGGGTGTACTGGATTGGGCCAGTGCTGGGTGCAGTTTTAGCAGGTGTGTCCCATGAGTTC
TTCTTTGCTCCCAGTGCGTCCAGACAGAAACTGGTTGCGTGTCTAACCTGCAAGGACATC
GAAATAGTGGAGACGGCCAGTGTGTCCCGATCTTCGCTCTCCACCGTCACTCAGACTGCC
ATGAGGGCTAAGCAAGCCCACAAG---TCTGAGCACAGC
>MN168500
GAGCTGCGGAGTCGTCAGTTCTGGCGGGCCGCCGCTGCTGAGCTGCTGGGCACGCTGGTG
TTTGTGTCGGCAGTGCTGGGTTCCTCCATACCAGGGCTGGAGGGCGGCGCTGGTGGGCCT
CTGTACCCTGCACTGGCAGGGGGCATGGTGGCCGTGAGCCTGGGACACTGCTTTGGGGAA
ATCAGTGGGGCTCAGGTAAACCCTGCGGTGACTCTGGCTTTCCTGGCCACCCGGCGTCTG
GACGCTCTGAGGGCGTTGGTGTATGTGGCGGCCCAGTGCCTTGGAGCCACTCTGGGCACA
GGGATCCTCTACTTGGCCCTCCCTCTGAATTCCACAGCAGAGAGCTTCGCCAACAAGGTC
CCCATGGAGGTGAATGCAGGCCAGGCTCTGGGGATGGAGTTTCTGGTCACCTTCCAGCTG
GTCTTTACCATCTTCTCTGTGGAGGAACAGCGGCGGCGGGAGGGGGCTGAACCAGGGAAC
CTGGCCATCGGCTTTTCACTGAGCGCAGGGATCTTTACAGCAGGTCGTCTCTCTGGGGGC
AGTATGAATCCTGCCAGAACTCTGGGCCCAGCCATCATCACTGGGATCTGGGAGCACCAC
TGGGTGTACTGGATTGGGCCAGTGTTAGGTGCGATCCTGGCTGCCGTGTCCCATGAGTTC
TTCTTCGCTCCCAGTGCATCCAGACAGAAGCTGGTGGCATGTCTGACCTGCAAGGACATT
GAGATGGTAGAGACGGCCAGCGTCTCCCGCTCCTCGCTCTCCACCATCACTCAGACGGCC
ATGAGGGCCAAGCAGGCCAACAAG---TCTGAGCACAGC
>MN168501
GAGCTGCGGAGCCGTCAGTTCTGGCGGGCCACCGCGGCCGAGGTGCTGGGCACGCTGGTG
TTTGTGTCGGCGGTGCTGGGCTCCTCCATTCCAGGGCCAGAGGGTAGCACTGGGGGGCCC
CTGTACCCTGCACTGGCAGCCGGCATGGTGGCGGTGGCTTTGGGGCACTGCTTTGGGGAA
ATAAGTGGAGCTCAGGTGAACCCTGCGGTGACCCTGGCGTTCTTGGCCACGCGGCGCCTG
GATGGCCTGAGGGCCCTGGTGTACGTGGCTGCCCAGTGCCTGGGGGCCACTCTGGGCACT
GGGGTCCTCTACCTCTCCCTGCCTCTCAAGGCCACCGCAGAGACATTCGCCAACAAGGTG
CCCATGGAGGTGAATGCGGGACAGGCTCTGGGGATGGAGATCCTGGCCACCTTCCAGTTG
GTCTTCACCATCTTCTCCGTGGAGGACCAGCGGCGGCGGGAGGGGGCTGAACCAGGAAAC
CTGGCCATCGGCTTCTCACTGAGTGCAGGGATCTTTACCGCAGGCCGGCTCTCTGGGGGC
AGTATGAATCCTGCCAGAACACTGGGTCCTGCCATCATCACTGGGATCTGGGAGCACCAC
TGGGTGTACTGGATTGGGCCTGTCTTAGGTGCCGTGCTGGCTGGCATCTCCCACGAGTTC
TTCTTCGCTCCAAGCGCGTCCAGACAGAAGCTGGTGGCCTGTCTGACCTGCAAGGACATC
GAGATGGTGGAGACGGCCAGCGTATCGCGCTCCTCACTCTCCACCGTCACCCAGACAGCC
ATGAGGGCCAAGCAGGCCAACAAG---TCCGAGCACGGC
>MN168502
GAGCTGAGGAGCCGGCAGTTTTGGCAAGCTGTGGCTGCAGAGGTATTGGGCACCCTGGTT
TTCGTATCTGCAGTACTGGGCTCCACATTGCCAGGGTCGGAGGGTCTCTCTGGGGGGACA
ATCTACCCAGCCCTGGCGGCTGGCACAGTGGCTGTGGCGCTAGGACACTGTTTCGGGGAG
ATCAGTGGAGCACAGGTGAACCCTGCGGTGACTCTGGCACTCTTGGCCACTCGGAAGCTG
GATGTACTCAGGGCATTGCTATACATAGTTGCCCAGTGCCTTGGGGCCACCCTTAGCACA

GGCCTCTTCTACCTGGCTTTACCTCAGAAATCCACCGCAGAGACATTTGCTAATAAAGTA
CCCATGGAGGTGAATGCAGGCCAGGCTTTGGGGATGGAGATCCTGGCCACCTTTCAGTTG
GTCTTCACCATCTTCTCAGTGGAAGACCAGCGAAGGAGAGATGGGGCTGAACCAGGGAAC
CTGGCCATTGGGTTCTCAGTCAGTGCAGGGATTTTCACTGCGGGTCGAATTTCAGGGGGA
AGCATGAATCCTGCCCGAACTTTTGGCCCTGCCATCATTACTGGGATCTGGGAGCATCAC
TGGGTATACTGGATAGGACCAGTGCTGGGAGCTATCCTAGCCGGGGTGTCTCATGAGTTC
TTCTTCGTGGCTAGTGCATCAAGGCAGAAGCTTGTGGCATGTCTGACTTGCAAGGACATT
GAGATTGTGGAGACAGCCAGTGTCTCACGGTCCTCTCTGTCCACCATCACTCAGACAGCG
ATGAGGGCCAAACAGGCCAACAAA---CCCGAGCAGAGC
>MN168503
GAGCTGCGAAGTCGCCAGTTTTGGCGTGCCTTGCTGGCAGAGGTTCTGGGTTCCCTCATC
TTTGTGTCTGCAGTGCTGGGGTCTTCAGTGCCAGGACCTGCAGACATGGCTCAGGGTCCC
CTGTACCCAGCTCTGGCCGCTGGCATGGTGGCCGTGGCGCTGAGCCATTGCTTTGGCGAG
ATCAGCGGCGCACAGGTGAACCCAGCCGTAACTCTGGCCTTTCTGGCCACACGACGGCTG
GACCTGCTGAGGGCTGCTGTCTACATATTGGGCCAATGCCTGGGCGCTACACTGGGGGCT
GCTCTCCTCTACCTGCTCCTACCTCTGAAATCCGCTGCAGAAGTGTATCCCAGCAAGGTG
TCCTCAGAAGGTAATGCAGGCCAGGCTTTGGGCATGGAGGTACTGGCTACTTTCCAGCTG
GTCTTCACCATCTTCTCTGTAGAGGATCAGCGGCGGCGGGAGGGAAGCGAGCCGGGAAAT
CTGGCCATCGGCTTCTCCTTGAGCGCCGGCGTACTGACCGCGGGTCGATTTTCTGGAGGC
AGCATGAATCCTGCTCGTTCTTTGGGTCCCGCTATTATCACAGGTTTTTGGGAACATCAC
TGGGTGTACTGGATAGGCCCAGTGTTGGGCTCCGTGCTGGCTGGTGTGTCCCATGAGTTC
TTTTTTGTGGCCAGTGCCTCTCGCCAGAAGCTGGTAGCCTGTCTCACCTGCAAGGACATC
GAGATCATGGAGGCCGCCAGCGTGTCCCGCTCCTCCTTGTCCACTGTCACACAGGCAGCC
ATGAGGGCCAAACAGGCTAACAAG---CAAGACCACAAC
>MN168504
GAGCTGCGGAGCCGCCAGTTTTGGCGGGCCTTGCTGGCAGAAGTTCTGGGTTCCCTCATC
TTCGTGTCTGTGGTCCTGGGGGCTTCAGTGCCTGGGCCCGGACATATGGCTCAAGGACCT
CTGTATCCAGCCCTAGCTGCTGGCATGGCTGCCGTGGCGCTGAGCCATTGCTTTGGGGAA
ATCAGCGGCGCACAGGTGAACCCTGCTGTCACATTGGCATTTCTGGCAACCCGGCGACTG
GATCTACTGAGGGCTACTGTCTACATAGTGGGCCAGTGCCTGGGAGCCACACTGGGGTCT
GGCCTCCTCTACCTGCTCCTACCTCTGAAATCTGCTGCTCAGGTGCTCCCCAGCAAGGTC
TCCCCAGATGGAAATGCAGGCCAAGCCTTGGCTGGTGAATTGCTGGCTACCTTCCAGCTG
GTCTTCACCATCTTCTCAGTAGAAGAGCAGCGAAAGAGGGAGGGCAGCGAGCCGGGAAAT
CTGGCTATTGGCTTCTCCTTGAGTGCTGGAGTGCTGGCTGCGGGCCGATTCTCTGGAGGC
AGCATGAATCCAGCTCGCTCTTTGGGTCCAGCTATTATAACAGGATTTTGGGACCATCAC
TGGGTGTACTGGATCGGCCCAGCGCTGGGCTCCGTGCTGGCAGGCATGTCCCATGAATTC
TTTTTTGTCGCCAGTGCCTCTCGCCAGAAGCTGGTAGCCTGCATTACCTGCAAGGACATT
GAGATCATGGAGGCTGCTAGCGTGTCCCGTTCCTCCTTGTCCACTGTAACGCAGGCAGCC
ATGAGGGCCAAGCAAACCACCAAG---CAGGACCACAAC
>MN168505
GAGCTACGAAGTCGTCAGTTCTGGCGGGCATTGCTGGCAGAGATTCTGGGTTCCCTGGTC
TTTGTGTCGGCAGTGCTGGGTTCCTCTGTGCCTGGGCCTGGCGATGTTGCTCCAGGACCA
CTGTACCCTGCCCTGGCAGGAGGCATGGTGGCTGTTGGACTTGCTCACTGTTTTGGAGAA
ATCAGCGGGGCACAGGTTAATCCCGCTGTCACTCTGGCATTCTTGGCAACACGTCGGCTG
GACCTGCTAAGGGCCGTCGCCTACATCCTCAGCCAGTGCCTGGGCGCCACGGTAGGGGCT
GCTATCCTCTACGTGCTTCTGCCCCTGGAGTCTGCAGCGGAATGCTACCCCAGCAAGGTA
TCTTCAGATGGAAACGCAGGCCAGGCTCTGGGGATGGAAGTCCTGGCCACGTTCCAGCTG
GTCTTCACCATCTTCTCTGTGGAGGACCAGCGGAGACGGGAGGGGAGTGAGCCCGGGAAC
CTGGCCATTGGCTTCTCCCTCAGTGCCGGTGTCCTTACTGCGGGCCGTTTTTCTGGAGGT
AGCATGAATCCAGCCAGGTCTTTGGGTCCCGCCATCATCACAGGATTCTGGGAGCATCAC
TGGGTGTACTGGATTGGTCCAGCTCTTGGGTCGGTGCTGGCCGGCGTGTCCCACGAGTTC

CTTTTCACACCCAGCGCCTCCCGTCAGAAGCTGGTCGCTTGCCTGACCTGCAAAGACATT
GAGATCATGGAGGCGGCCAGTGTATCCCGCTCGTCCTTGTCCACCGTCACCCAGGCGGCC
ATGAGGGCCAAGCAGGCCAATAAG---CACGACCACAGC
>MN168506
GAGCTGCAGAGACGACAGTTCTGGCGTGGGGTGCTGGCAGAGACCCTCGGTTCACTCATT
TTCGTGTCGGCGGTGTTGGGCGCTTCGGTACCGGGCCCCGGCGATGTTGCTCAAGGGCCC
CTGTACCCCGCCCTGGCTGCAGGGATGGTGGCAGTGAGCCTGGGGCACTGTTTTGGGGAG
ATGAGTGGAGCCCAGGTGAACCCTGCTGTTACACTGGCCTTCCTGGCCACCCGCCGGCTG
GACCTGCTGAGGGCCGTAGCCTACATCCTGGGCCAGTGTCTGGGGGCCACCCTGGGGGCT
GGAATCCTCTACCTGGCCCTACCACTCAAATCTGCTGCCAAATGCTACGTCAGCAAGGTG
TCCTCGGAGGGTAATGCTGGCCAGGCTCTTGGCATGGAGGTGCTGGCCACCTTCCAGCTG
GTCTTCACCATTTTCTCTGTGGAGGAGCAGCGGCGGCGGGAGGGGTCTGAGCCCGGTAAC
CTGGCCATCGGGTTCTCCCTGAGCGCCGGCATTCTAACCGCGGGCCTTTTCTCTGGGGGC
AGCCTGAATCCTGCTCGTTCCCTGGGTCCCGCCATCATCACAGGAATCTGGGAGCACCAC
TGGGTGTACTGGATTGGGCCGGTGCTGGGGGCCATCCTTGCCGGGGTGTCCCACGAGTTC
TTCTTCGCGGCCAGTGCCTCTCGGCAGAAGCTTGTGGCCTGCCTTACCTGCAAGGACATA
GAGATCATCGAGACGGCCAGCGTCTCCCGATCCTCACTGTCCACCGTCACTCAGACTGCC
ATGAGGGCCAAACAGGCCCACAAA---CACGACCACAAC
>MN168507
GAGCTGCAGAGACGACAGTTCTGGCGTGCGGTGCTGGCAGAGACCCTCGGTTCACTCATT
TTCGTGTCGGCGGTGTTGGGTGCTTCGGTACCGGGCCCCGGCGATGTTGCTCAAGGGCCC
CTGTACCCCGCCCTGGCTGCAGGGATGGTGGCAGTGAGCCTGGGGCACTGTTTTGGGGAG
ATGAGTGGAGCCCAGGTGAACCCTGCTGTTACACTGGCCTTCCTGGCCACCCGCCGGCTG
GACCTGCTGAGGGCCGTAGCCTACATCCTGGGCCAGTGTCTGGGGGCCACCCTGGGGGCT
GGAATCCTCTACCTGGCCCTACCACTCAAATCTGCTGCCAAATGCTACGTCAGCAAGGTG
TCCTCGGAGGGTAATGCTGGCCAGGCTCTTGGCATGGAGGTGCTGGCCACCTTCCAGCTG
GTCTTCACCATTTTCTCTGTGGAGGAGCAGCGGCGGCGGGAGGGGTCTGAGCCCGGTAAC
CTGGCCATCGGGTTCTCCCTGAGCGCCGGCATTCTAACCGCGGGCCTTTTCTCTGGGGGC
AGCCTGAATCCTGCTCGTTCCCTGGGTCCCGCCATCATCACAGGAATCTGGGAGCACCAC
TGGGTGTACTGGATTGGGCCGGTGCTGGGGGCCATCCTTGCCGGGGTGTCCCACGAGTTC
TTCTTCACGGCCAGTGCCTCTCGGCAGAAGCTTGTGGCCTGCCTTACCTGCAAGGACATA
GAGATCATCGAGACGGCCAGCGTCTCCCGATCCTCACTGTCCACCGTCACTCAGACTGCC
ATGAGGGCCAAACAGGCCCACAAA---CACGACCACAAC
>MN168508
GAGCTGCAGAGACGACAGTTCTGGCGTGCGGTGCTGGCAGAGACCCTCGGTTCGCTGATT
TTTGTGTCGGCGGTGTTGGGCTCTTCGGTACCGGGCCCCGGCGATGTTGCTCACGGGCCG
CTGTACCCCGCCCTGGCTGCAGGGATGGTGGCAGTGAGCCTGGGGCACTGTTTTGGGGAG
ATGAGTGGAGCCCAGGTGAACCCTGCTGTTACACTGGCCTTCCTGGCCACCCGCCAGCTG
GACCTGCTGAGGGCCGTAGCCTACATCCTGGGCCAGTGTCTGGGGGCCACCCTGGGGGCT
GGAATCCTCTACCTGGCCCTACCACTCAAATCTGCTGCCAAATGCTACGTCAACAAGGTG
TCCTCGGAGGGTAATGCTGGCCAGGCTCTTGGCATGGAGGTGCTGGCCACCTTCCAGCTG
GTCTTCACCATTTTCTCTGTGGAGGAGCAGCGGCGGCGGGAGGGGTCTGAGCCCGGTAAC
CTGGCCATCGGGTTCTCCCTGAGCGCCGGCATTCTAACTGCGGGCCTTTTCTCTGGGGGC
AGCATGAACCCTGCTCGTTCCCTGGGTCCCGCCATCATCACAGGAATCTGGGAGCACCAC
TGGGTGTACTGGATTGGGCCGGTGCTGGGGGCCATCCTTGCCGGGGTGTCCCATGAGTTC
TTCTTCGCGGCCAGTGCCTCTCGGCAGAAGCTTGTGGCCTGCCTTACCTGCAAGGACATC
GAGATCATTGAGACGGCCAGCGTCTCCCGATCCTCACTGTCCACCGTCACCCAGACTGCC
ATGAGGGCCAAACAGGCCCACAAA---CACGACAACAAC
>MN168509
GAGCTGCGGAGCCGGCAGTTCTGGCGCGCGGTGCTGGCGGAGGTCCTGGGGTCCCTGATC
TTCGTGTCGGCTGTGCTGGGCTCCTCGGTGCCGGGCCCCGGCGAAGTG------GGGCCC

ATCCAGCCGGCCCTCGCGGCCGGATTTGTGGCCATGGGGCTGGGCCAGTGTTTCGGGGAC
ATCAGCGGGGCTCAGGTGAACCCTGCCGTCACGCTGGCCTTCCTGGCCACCCGCAAGCTC
GACCTGCTGAGAGCGGCCGGCTACGTCCTGGGCCAGTGTGTGGGGGCCACGCTGGGGGCT
GGCGTCCTCTTCCTGGCCCTTCCTGTGAAGTCTGCAGCGGAGTGCTACGTCAGCAAGGTG
TCCACTGAGGCCAATGCAGGCCAGGCACTGGCCATGGAGGTCCTGGCCACCTTCCAGCTG
GTCTTCACCATCTTCTCAGCCGAGGAGAGGCGGCAGCGGGACGGGGGCGACCCGGGCAGC
TGGGCCGTGGGGTTCTCGCTGAGCGCCGGCGTGCTGACCGCGGCCCGTTTCTCTGGTGGC
AGTATGAACCCTGCTCGTTCCCTGGGGCCAGCCATCATCACCGGGATCTGGGAGCATCAC
TGGGTGTACTGGATTGGGCCGATGATGGGTTCGGTTCTGGCGGGGGTGTCCCATGAGTTC
TTCTTTGCAGCCAGCGCCTCTCGACAGAAGCTGATCGCTTGCCTGACGTGCAAGGACATC
GAGATCGTGGAAACAGCCAGCCTGTCGCGCTCTTCGCTGTCCACCGTCACCCAGACCGCC
ATGAGGGCCAAACAGGCCAACAAG---CATGACCACAAC
>MN168510
GAGTTGAAGAGCCGCCGATTCTGGCGCGCAGTCCTGGCTGAGACCCTCGGCTCTCTGATC
TTTGTGATGGCAGCGCTGGGCTCCTCGCTGCCCAGTCCAGGGCAGGGCTGCTCGGTTCCC
CTGCAGCCGGCTCTGGCGGTCGGGTTCACAGTGGTGGGGCTGGGTCACTGCTTCGGGGAG
ATCAGTGGAGCCCAGATGAACCCTGCAGTCACCCTGGCCTTCCTTGCCACCCGCAAACTA
GACATTCTACGAACAGCCTGCTACATCCTGGGCCAGTGTCTGGGAGCCACGCTGGGGGCT
GGGATCCTGTACCTCACCCTGCCTCTGAAGTCTGCAGCAGAGTGCTATGTGAACAAGGTG
AACTCAGAGGGCAATGCTGGCCAGGCTCTGGGGATGGAAGTCCTTGTCACCTTCCAGCTG
GTCTTCACCATCTTCTCTGTGGAGGATCACAGGAGGAGGGAGGTGGGGGAGCCAGGGAAC
CTGGCCATCGGATTGTCAGTGAGCGCCGGCGTGCTCACGGCGGGTCGTTTCTCCGGAGGC
AGCATGAATCCCGCTCGATCCCTGGGCCCTGCGATAATCACAGGGATCTGGGAGCATCAC
TGGGTGTACTGGATCGGTCCCATGCTGGGTGCAGTCCTGGCTGGACTCTCTCATGAGTTC
TTCTTCGCAGCCAGCGCTTCTCGCCAGAAGCTGATCGCCTGCCTCACGTGTAAAGACATC
GAGATCATGGAGACGGCTAGCGCGTCACGGTCCTCACTGTCCACCGTCACACAGTCCGCC
ATGCGGGCCAAACACACCAACAAG---AATGACCACAAC
>MN168511
GAGTTGAAGAGCCGCCGATTCTGGCGCGCAGTCCTGGCCGAGACCCTCGGCTCTCTGATC
TTTGTGATGGCAGCGCTGGGCTCCTCGCTGCCCAGTCCAGGACAGGGCTGCTCGGTTCCC
CTGCAGCCAGCTCTGGCGGTCGGGTTCACAGTGGTGGGGCTGGGTCACTGCTTCGGGGAG
ATCAGTGGAGCCCAGATGAACCCTGCAGTCACCCTGGCCTTCCTTGCCACCCGCAAACTA
GACATTCTCCGAACAGCCTGCTACATCCTGGGCCAGTGTCTGGGAGCCACGCTGGGGGCT
GGGATCCTGTACCTCACCCTGCCTCTGAAGTCTGCAGCAGAGTGCTATGTGAACAAGGTG
AACTCAGAGGGCAATGCTGGCCAGGCTCTGGGGATGGAAGTCCTTGTCACCTTCCAGCTG
GTCTTCACCATCTTCTCTGTGGAGGATCACAGGAGGAGGGAGGTGGGGGAGCCAGGGAAC
CTGGCCATCGGATTGTCAGTGAGCGCCGGCGTGCTCACGGCGGGTCGTTTCTCCGGAGGC
AGCATGAATCCCGCTCGATCCCTGGGCCCTGCGATAATCACCGGGATCTGGGAGCATCAC
TGGGTATACTGGATCGGTCCCATGCTGGGTGCAGTCCTGGCTGGACTCTCTCATGAGTTC
TTCTTCGCAGCCAGCGCTTCTCGTCAGAAGCTGATCGCCTGTCTCACGTGTAAAGACATC
GAGATCATGGAGACGGCTAGCGCGTCACGGTCCTCACTGTCCACCGTCACACAGTCCGCC
ATGCGGGCCAAACACACCAACAAG---AATGACCACAAC
>MN168512
GAGCTGAAGAGCCGCAGATTCTGGCGTGCAGTTCTGGCAGAGATACTAGGCTCACTGATA
TTCGTTTCAGCAGTGTTAGGTTCCTCAATACCAAATCAAGCACAGGATTGCTCGATTCTT
CTGCAGCCTGCTCTGGCTGCTGGATTCACAGCTGTGGGACTTGGCCACTGTTTTGGAGAC
ATTAGTGGAGCGCAAGTGAATCCTGCAGTCACCCTGGCTTTTCTTGCCACACGGAAGTTG
GACATTCTGCGAACGGCTTCATACATAATTGGTCAATGTCTGGGGGCTACAATGGCAGCT
GGTATCCTCTACCTTACAATTCCTCTGAAATCAGCAGCCCGGTGCTATGTAAACATGGTT
AGTCCTGAAAGCAATGCAGGCCAAGCTCTGGGAATGGAGGTCCTTGCCACTTTCCAGCTT
GTTTTCACCATCTTCTCAGTAGAGGACCACAGGCGACGAGAGGTGGGAGAACCAGGAAAC

CTTGCTATTGGATTTTCTTTGAGTGCCGGAATACTCACTGCGGGCCACATTTCAGGGGGG
AGCATGAATCCTGCCCGCTCACTTGGGCCAGCAATAATAACAGGCTACTGGGAACACCAC
TGGGTATACTGGATTGGTCCGATGCTTGGCGCACTAATGGCTGGTTTTTCACACGAGTTC
TTCTTTGCTGCTACAGCCTCTCGTCAGAAACTTATCAACTGCATAACATGTAAAGACATT
GAGATAATTGAAACAGGCAGCGTATCCAGATCCTCATTATCCACAGTTACCCAGTCAGCA
ATGCGAGCAAAACAGTCCAATAAA---CACGACCATAGC
>MN168513
GAATTGAGGAGTCGCAGGTTCTGGCGTTCAGTTTTAGCAGAGCTGGTTGGTTCTCTGGTC
TTGGTTGCAGTGATTTTGGGTGCCTCTGCCCCTGGACAGGAGGACGAGGTGCCAGCCTTG
ATGCAGGTGGCTGTAGCTGCGGGGTTTTCTGCTGTCAGCCTGATCCACTGTTTCGGAGAG
ATTAGCGGTGCTCAGGTAAACCCAGCTGTCACACTCGCGTTCTTGTGCACCAGGAAACTG
GATTTCCTTCAGGTTGTGTCCTACCTTTTGGCTCAATGTCTTGGAGCTGTGATTGGATCA
GGGATAATCTACATGTCACTGCCAGTCAAGTCAGCATCAAGGCATTTAGTTAACATGGTA
AGTAAGGATGGAAATGCTGGCCAAGCACTTGCAATGGAGATTTTTGCCACATTCCAATTA
GTTTTCACGATATTTGCTGTGGATGATCATCGGCGCAGGGAAGTGAGAGAACCTGGCAGT
TTGGCAATAGCTCTCAGTCTGACAGCAGGAATCATGGCA---GGTAAGTTTTCAGGAGGC
AGCCTGAATCCTGCAAGATCTCTTGGACCAGCGATAATCACCGGATTCTGGGAGCATCAC
TGGGTATACTGGATTGGTCCTATCCTTGGTGCTGTCCTTGGTGGCATTTCTTATGAGTTC
TTTTTTGCCTCCAGTGCCTCTCAAGAAAAGCTCATTGCCTGTATCACATGCAAAGATATC
GAGATTGTGGAAACTACCAGTGTGTCCCGTTCATCATTACTGACAGTCACACAATCTGCA
ATGCGAGCAAAACAGACTGCAAAAGTGCAGGATCACAGT
>MN168514
GAGTTGAGGAGTCGCAGGTTCTGGCGTTCAGTTTTGGCAGAGCTGGTTGGCTCCCTGGTC
TTGGTTGCAGTGATTTTGGGTGCCTCTGCCCCTGGACAGGAGGACGAGGTGCCTGCCTTG
ATGCAGGTGGCTGTAGCTGCGGGGTTTTCTGCTGTCAGCCTGATCCACTGTTTCGGAGAG
ATTAGCGGTGCTCAGGTAAACCCAGCTGTCACACTCGCGTTCTTGTGCACCAGGAAACTG
GATTTCCTTCAGGTTGTGTCCTACCTTTTGGCTCAATGTCTTGGAGCTGTGATTGGATCA
GGGATAATCTACATGTCACTGCCAGTCAAGTCAGCATCAAGGCATTTAGTTAACATGGTA
AGTAAGGATGGAAATGCTGGCCAAGCGCTTGCAATGGAGATTTTTGCCACATTCCAATTA
GTTTTCACGATATTTGCTGTGGATGATCACCGGCGCAGGGAAGTGGGAGAACCTGGCAGT
TTGGCAATAGCTCTCAGTCTGACAGCAGGAATCATGGCA---GGTAAGATTTCAGGAGGC
AGCCTGAATCCTGCAAGATCTCTTGGACCAGCGATAATCACCGGATTCTGGGAGCATCAC
TGGGTATACTGGATTGGTCCTATCCTTGGTGCTGTCCTTGGTGGCATTTCTTATGAGTTC
TTTTTTGCCTCCAGTGCCTCTCAAGAAAAGCTCATTGCCTGTATCACATGCAAAGATATC
GAGATTGTGGAAACCACCAGTGTGTCCCGTTCATCATTACTGACAGTCACACAATCTGCA
ATGCGAGCAAAACAGACTGCAAAAGTGCAGGATCACAGT
>MN168515
GAGTTGAGGAGTCGCAGGTTCTGGCGTTCAGTTTTAGCAGAGCTGGTTGGTTCCCTGGTC
TTGGTCACAGTTATTTTGGGTGCCTCTGCTCCTGGACAGGAAGATGGACTTCCAGCGTTG
ATTCAGGTGGCTGTAGCTGCGGGGTTTTCTGCTGTCAGCCTGATCCACTGTTTTGGAGAG
ATTAGCGGTGCTCAGGTAAACCCAGCTGTCACAGTCGCATTCTTGTGCACCAGGAAACTG
GATTTCCTTCAGTTTGGGTCCTACCTTCTGGCTCAATGTCTTGGAGCTGTGATTGGATCA
GGGATAATCTACATGTCACTGCCAGTCAAGTCAGCATCAAGGCACTTAGTTAACATGGTA
AGTCCAGATGGAAATGCTGGCCAAGCACTTGCCATGGAGATTTTTGCCACATTCCAGCTG
GTTTTCACTATATTTGCAGTGGATGATCACCGGCATAGGGAAGTGGGAGAACCTGGCAGT
TTGGCAATAGCGCTCAGTCTGACAGCAGGAATCCTGGCA---GGTAAGTTTTCAGGAGGC
AGCCTGAATCCTGCAAGATCCCTTGGACCAGCGATAATCACCGGATTCTGGGAGCATCAC
TGGGTATACTGGATTGGTCCTATCCTTGGTGCTGTCCTTGGTGGCATTTCTTATGAGTTC
TTTTTTGCCTCCAGTGCCTCTCAAGAAAAGCTCATTGCATGTATCACCTGCAAAGATATC
GAGATTGTGGAAACCGCCAGTGTGTCCCGTTCATCATTACTGACGGTCACACAGTCTGCA
ATGAGAGCAAAACAGACTGCAAAAGTGCAGGATCACAGT

>MN168516
GAGTTGAGAAGTCGCAGGTTCTGGCGTTCAGTCTTAGCAGAGTTGGTTGGTTCCCTGGTC
TTGGTCTCCGTTATTCTGGGGGCCTCTGCCCCCGACCAGGAAGGTGGAGTTCCAGCGCTG
ATGCAGGTGGCTGTAGCTGCTGGATTTTCTGCTGTCAGCTTGATCCACTGCTTTGGAGAG
ATTAGCGGTGCCCAGGTAAACCCAGCTGTCACCCTCGCATTCTTGTGCACCAGGAAACTG
GATTTTCTCCAGTTTGTGTCCTATCTTTTGGCTCAGTGTCTTGGAGCTGTGATTGGATCA
GGGATAATCTACATGTCATTGCCAAACAAGTCAACATCAAGGTATTTAGTCAACATGGTG
AGTCCAGATGGAAATGCTGGCCAAGCACTTGCAATGGAGATTTTTGCCACATTCCAGCTG
GTTTTTACTATATTTGCCATGGGGGATCATCGCCGTAGGGAAGTGGGAGAATCTGGTAGC
CTGGCAATTGCGTTCAGTCTGACAGCAGGAATCCTGGCA---GGTAAGTTTTCAGGAGGA
AGCCTGAATCCTGCAAGATCTCTTGGACCAGCAATAATCACCGGATTCTGGGAGCACCAT
TGGGTGTACTGGATTGGCCCTATCCTTGGTGCTGTTCTTGGTGGTATTTCTTACGAGTTC
TTTTTCGCCTCCAGCGCCTCCCAGGAAAAGCTCATTGCCTGTATCACCTGTAAAGATATT
GAGATTGTGGAAACCGCCAGTGTGTCCCGTTCATCATTACTGACAGTCACGCAGTCTGCA
ATGCGAGCAAAGCAGACTGCAAAAGTGCAGGACCACAGT
>MN168517
GAGTTGAGGAGCCGCAGGTTCTGGCGTTCAGTCTTAGCAGAGCTGGTTGGTTCCCTGGTC
TTGGTCTCAGTTATTTTGGGTGCCTCTGTCCCTGGACAGGAAGATGGAGTTCCAGTACTG
ATGCAGGTGGCTGTAGCTGCTGGATTTTCTGCTGTCAGCCTGATCCACTGTTTTGGAGAG
ATTAGTGGTGCCCAGGTAAACCCAGCTGTCACACTCGCATTCTTGTGCACCAGGAAACTG
GATTTCCTCCAGTTTGTGTCCTATCTTTTGGCTCAGTGTCTTGGAGCTGTGATTGGATCA
GGGATAATCTACATGTCATTGCCAATCAAGTCAACATCAAGGTATTTAGTCAACATGGTG
AGTCCAGATGGAAATGCTGGCCAAGCACTTGCAATGGAGATTTTTGCCACATTCCAGCTG
GTTTTCACTATATTTGCCGTGGATGATCATCGGCGTAGGGAAGTGGGAGAATCTGGTAGT
CTGGCAATAGCGTTCAGTCTGACAGCAGGAATCCTGGCA---GGTAAGTTTTCAGGAGGC
AGCCTGAATCCCGCAAGATCTCTTGGACCAGCGATAATCACCGGATTCTGGGAGCATCAC
TGGGTATACTGGATTGGCCCTATCCTTGGTGCTGTCCTTGGTGGTATTTCTTATGAGTTC
TTTTTTGCCTCCAGCGCCTCCCAAGAAAAGCTCATTGCCTGTATCACCTGCAAAGATATC
GAGATTGTGGAAACCGCCAGTGTGTCCCGTTCATCATTACTGACGGTCACGCAGTCTGCG
ATGCGAGCAAAACAGACTGCAAAAGTGCAGGACCACAGT
>MN168518
GAGTTGAAGAGTCGCAGGTTCTGGTGTTCAGTCTTAGCAGAGCTGGTTGGTTCCCTAGTC
TTGGTCTCAGTTATTTTGGGAGCCTCTGCCCCTGGACAAGAAGATGGAGGTCCAGTGCTG
ATGCAGGTGGCT---------------------------CTGATCCACTGTTTTGGAGAG
ATTAGTGGTGCCCAGGTAAACCCAGCTGTCACAATTGCATTCTTGTGCACAAGGAAACTG
GATTTCCTCCAGTTTGTGTCCTATCTTTTGGCTCAGTGTCTTGGAGCTGTGATTGGATCA
GGGATAATCTACATGTCACTGCCAATCAAGTCAACATCAAGGCATTTAGTCAACATGATC
AACAAAGATGGAAATGCTGGCCAAGCACTTGCAATGGAGATTTTTGCCACATTCCAGCTG
GTTTTCACTATATTTGCTGTGGATGATCATCGACGTAGGGAAGTGGGAGAACCTGGCAGT
CTGGCAATAGCCTTCAGTCTGACAGCAGGAATCCTGGCA---GGTAAGTTTTCAGGAGGC
AGCCTGAATCCCGCAAGATCTCTTGGACCAGCGGTAATCACTGGATTCTGGGAGCACCAC
TGGGTA--

>MN168519
GAGCTGCGGAGCCGCAGGTTCTGGCGTTCGGTCCTAGCGGAGCTGGTTGGTTCCCTGATC
TTGGTCACGGTTATCCTGGGAGCCTCGGCCCCTGGGCAGGAGGATGAAGCTCCGGTCCTG
ATGCAGGTGGCTGTAGCCGCCGGCTTCTCGGCGCTCAGCCTGATGCACTGCTTCGGAGAA
ATCAGCGGCGCGCAGATGAACCCGGCTGTCACCCTGGCCTTGCTCTGCACCAGGAAGCTG
GACGGCCTCCAGTCTGTGTTCTACCTTGTGGCCCAGTGCCTTGGCGCTGTGCTTGGAACA

GGAATAATCTACATGTCACTCCCGGTCAAGGCAACATCAAGGCTTCTAGTCAACATGGTG
AGTACAGATGGGAATGCCGGCCAGGCCTTGGCAATGGAGATTTTTGCCACATTCCAGCTG
GTTTTCACCATCTTTGCCGTGGACGACCATCGGCGCAGGGAGGTGGGAGAACCGGGAAAC
CTGGCCGTTGCCCTGAGCCTGGCGACAGGAGTCCTCCAG---GGAAAGTTCTCGGGAGGC
AGCCTGAATCCTGCACGATCGCTTGGACCAGCTGTATTCACCGGAGTCTGGGAGCATCAC
TGGGTGTACTGGATTGGCCCCATCCTTGGTGCTGTCCTTGGGGGTGTTTCTTACGAGTTC
TTTTTTGCCTCCAGTGCCTCCCAGGAGAAGCTGATTGCCTGCATCACCTGCAAAGATATT
GAGATTGTGGAAACGGCCAGTGTGTCTCGGTCTTCGTTACTGACGGTCACCCAGTCCGCG
ATGAGGGTGAAACCCACTGCAAAAGTCCAGGACCAGTTA
>MN168520
GAGCTGCGGAGCCGCAGGTTCTGGCGTTCGGTCCTAGCGGAGCTGGTTGGTTCCCTGATC
TTGGTCACGGTTATCCTGGGAGCCTCGGCCCCTGGGCAGGAGGATGGAGCTCCGGTCCTG
ATGCAGGTGGCTTTAGCCGCTGGCTTCTCGGCGCTCAGCCTGATGCACTGCTTCGGAGAA
ATCAGCGGCGCGCAGATGAACCCAGCCGTCACCCTGGCCTTGCTCTGCACCAGGAAGCTG
GATGGCCTCCAGTTTGTGTTCTACCTTGTGGCCCAGTGCCTCGGCGCTGTGCTTGGAACA
GGGATAATCTACATGTCACTCCCGGTCAAGGCAACATCAAGGCTTCTAGTCAACATGGTG
AGTACAGACGGGAATGCCGGCCAGGCCTTGGCAATGGAGATTTTTGCCACATTCCAGCTG
GTTTTCACCATCTTTGCCGTGGACGACCATCGGCGCAGGGAGGTGGGAGAACCGGGAAAC
CTGGCCGTAGCCCTGAGCCTGGCGACAGGAGTCCTCCAG---GGAAAGTTCTCGGGAGGC
AGCCTGAATCCTGCACGATCGCTCGGACCAGCTGTATTCACCGGAGTCTGGGAGCATCAC
TGGGTATACTGGATTGGCCCCATCCTTGGTGCTGTCCTTGGAGGTGTTTCTTACGAGTTC
TTTTTTGCCTCCAGTGCCTCCCAGGAGAAGCTGATTGCCTGCATCACCTGCAAAGATATT
GAGATTGTGGAAACGGCCAGTGTGTCTCGGTCTTCGTTACTGACGGTCACCCAGTCCGCT
ATGAGGGTGAAACCCACTGCGAAAGTGCAGGACCAGTTA
>MN168521
GATTTGAGGAGCCAGCGTTTCTGGCGCTGTGTGTTAGCTGAGCTGTTGGCCTCCATCATC
CTGGTGTGGGTGATCCTGGGCAGCTCCGTGCAGGGGCAGGGGGTGGGGGTCTCGGGGCTG
GTGCAGGTGGCCGTGGCCGGGGGCTTCTCGGTGGTCAGCCTGGTGCAGTGTTTCGGGGAG
ATCAGTGGGGCCCATGTTAACCCTGCAGTGACGGTGGCTTTCCTCTGCACCAGGAAGCTG
GATGTGCTGTGGTCTGTGTCCTACATCTTGGCTCAGTGTCTCGGAGCCATCCTGGGCTCG
GGGATGGTCTACCTGTCGCTGCCCATCACCTCGTCGCCAGCCCAGCTGGTCAGTGTGATC
AGCGAGGACGGTAATGCAGGCCAGGCGTTGGGGATGGAGGTCTTGGCCACATTCCAGTTG
GTTTTCACCATCTTCGCAGTGAACGAGCGTCGCAGGAGCAAAGGTGCGGAGCCTGGCGGC
TTAGCCATCGGCCTCAGCCTGACCGCCGGCATCTTGGCCTCG------------------

>MN168522
GAGATCCGCAGCGCGTCCCTCTGGCGCGCCGCGCTCGCCGAGGCCGTGGGCACTTTCCTC
CTCGTCCTCGCCGTGCTCGGCGCGGCCTGGCCCGGCACGGGAGAAGGGCCCGCGGCCTCC
CTGCCCGTGGCTCTGAGCGCCGCCTTCTCGTGCGCGGCGCTGTCCCACAGCCTGGGACCG
ACAAGCGGCGCGCAGTTCAACCCCGCCGTCACAGTGGCCCTGCTGTGCACGCGCCGCCTC
GGCGCTCTGCCCTCGGTGGCCTACGTGTTGGCACAGTGCCTGGGCGCGGTCGTGGCGGCC
GGAGTCGTTGCCCTGCTGCTGCCGGAACGGTCCGGCAGAAAGTACTTGGTCACGTCGATT
GGCAGCGAGGGCAACGCGGGGCAGGCGCTGGCGGCCGAGCTCCTCTGCAGCTTCCAGCTG
GTGCTGAGCGCCCTGGCTGGGGAGGAGCACCGCGTGCGCCGCTCCGGGGAGGCGGAGCCG
CTGCCGGTCGGGCTGGCACTGGGCGCCGGCATCCTCGCCTCGGAGAAGTTCTCTGGGGGC
AGCCTCAATCCAGCACGGTCGTTTGGACCTGCACTCGTGTGCGGATTATGGAAACACCAC
TGGGTA---------GGCCCCTGCTGGGCGGCCTCCTGGCCGCCGCCGCTCACGACCTC

GTCTTCTCGTCGGCCGCGAACCGCGACCGCCTCGTCGCGTGCGCCACGTGCCGCGACATC
GAGATCACGGAGGCGCGCAGCGCGTCCCGCTCGTCGCTGGCGCCGCTGCCCGGCGTGGCC
GCCGCGCCCGCCCTCCCCAAGCGCCACTCCAAAGTGTCC
>MN168523
GAGATCCGCAGCGCGTCCCTCTGGCGCGCCGCGTTCGCCGAGGCCGTGGGCACTTTCCTC
CTCGTCCTCGCCGTGCTCGGCGCGGCCTGGCCCGGCACGGGAGAAGGGCCCGCGGCCTCC
CTGCCCGTGGCGCTGAGCGCCGCCTTCTCGTGCGCGGCGCTGTCCCACAGCCTGGGACCG
TCGAGCGGCGCGCAGCTCAACCCCGCCGTCACGGTGGCCCTGCTGTGCACGCGCCGCCTT
GGGGCCCTGCCCTCGGTGGCCTACGTGTTGGCGCAGTGCCTGGGCGCGGTCCTGGCGGCC
GGGATCGTTGCCCTGCTGCTGCCGGAACGTTCCGGCAGAAAGTACTTGGTCACGTCGATT
GGCAGCGAGGGCAACGCGGGGCAGGCGCTGGCTGCCGAGCTCCTCTGCAGCTTCCAGCTG
GTGCTGAGCGCGCTCGCCGGGGAGGAGCACCGCGTGCGCCGCTCCGGGGAAGCGGGGCCG
CTGCCCGTCGGGCTGGCACTGGGCGCCGGAATCCTCGCCTCGGAGAAGTTCTCGGGAGGC
AGCCTCAATCCAGCGCGGTCGTTTGGACCAGCACTCGTGTGCGGATTATGGAAACACCAC
TGGGTCTACTGGATGGGGCCTCTGCTGGGCGGCCTCCTGGCTGCCGCCGCTCACGACCTC
GTCTTCTCGTCGGCCGCGAACCGCGACCGCCTCGTCGCGTGCGCCACGTGCCGCGACATC
GAGATCACGGAGGCGCGCAGCGCGTCCCGCTCGTCGCTGGCGCCGCTGCCCGGCGTGGCC
GCCGCGCCCGCGATCCCCAAGCGCCACTCCAAAGTGTCC
