# Supplementary material for: The vertebrate Aqp14 water channel is a neuropeptide-regulated polytransporter
Source: Commun Biol. 2019 Dec 11;2:462. doi: 10.1038/s42003-019-0713-y (PMC6906440; doi:10.1038/s42003-019-0713-y)
Supplement: Supplementary file 2 — Description of Additional Supplementary Files [file 42003_2019_713_MOESM2_ESM.pdf]

Description of Additional Supplementary Files

File Name: **Supplementary Data 1**
Description: Source data
